# Discovery of 2-(3-Benzamidopropanamido)thiazole-5-carboxylate Inhibitors of the Kinesin HSET (KIFC1) and the Development of Cellular Target Engagement Probes

**DOI:** 10.1021/acs.jmedchem.2c01591

**Published:** 2023-02-07

**Authors:** François Saint-Dizier, Thomas P. Matthews, Aaron M. Gregson, Hugues Prevet, Tatiana McHardy, Giampiero Colombano, Harry Saville, Martin Rowlands, Caroline Ewens, P. Craig McAndrew, Kathy Tomlin, Delphine Guillotin, Grace Wing-Yan Mak, Konstantinos Drosopoulos, Ioannis Poursaitidis, Rosemary Burke, Rob van Montfort, Spiros Linardopoulos, Ian Collins

**Affiliations:** † Centre for Cancer Drug Discovery, Division of Cancer Therapeutics, The Institute of Cancer Research, London SW7 3RP, U.K.; ‡ Division of Structural Biology, The Institute of Cancer Research, London SW7 3RP, U.K.; § Breast Cancer Now Research Centre, The Institute of Cancer Research, London SW7 3RP, U.K.

## Abstract

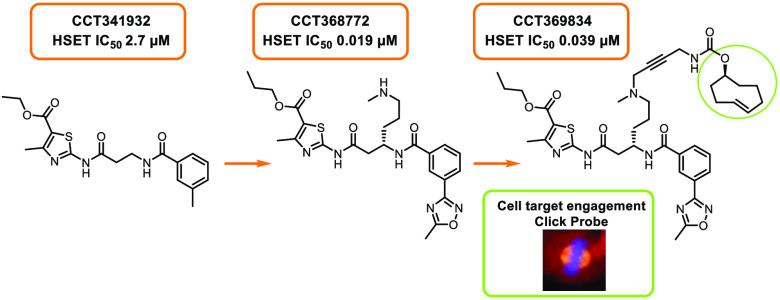

The existence of multiple centrosomes in some cancer cells can lead to cell death through the formation of multipolar mitotic spindles and consequent aberrant cell division. Many cancer cells rely on HSET (KIFC1) to cluster the extra centrosomes into two groups to mimic the bipolar spindle formation of non-centrosome-amplified cells and ensure their survival. Here, we report the discovery of a novel 2-(3-benzamidopropanamido)thiazole-5-carboxylate with micromolar in vitro inhibition of HSET (KIFC1) through high-throughput screening and its progression to ATP-competitive compounds with nanomolar biochemical potency and high selectivity against the opposing mitotic kinesin Eg5. Induction of the multipolar phenotype was shown in centrosome-amplified human cancer cells treated with these inhibitors. In addition, a suitable linker position was identified to allow the synthesis of both fluorescent- and *trans*-cyclooctene (TCO)-tagged probes, which demonstrated direct compound binding to the HSET protein and confirmed target engagement in cells, through a click-chemistry approach.

## Introduction

The human spleen, embryo, and testes protein (HSET), also known as KIFC1 or kinesin-14a, is a member of the kinesin-14 motor protein family.^1^ Like other kinesin motor proteins, HSET consists of a microtubule binding domain (MBD), a coiled-coil stalk, and a motor domain at the C-terminus.^2^ HSET forms homodimers with an antiparallel orientation of the individual proteins so that the MBDs are located at either end of the dimer. In turn, HSET dimers cross-link parallel and antiparallel microtubules (MTs) in the cellular mitotic spindle. The opposing orientations of the MBDs enable the kinesin motor to slide antiparallel MTs relative to the parallel MTs due to the directional movement of each individual HSET protein toward the minus ends of the MTs in an ATP hydrolysis-dependent manner.^2,3^

During mitosis, the assembly of a bipolar mitotic spindle is required to allow an equal partition of the replicated chromosomes into the daughter cells and avoid deleterious and typically lethal multipolar cell division.^4^ In some cancer cells, multiple microtubule organizing centers (MTOCs) are present due to the existence of multiple centrosomes, which can impair the bipolar spindle assembly and thus lead to uneven separation of the chromosomes, multipolar divisions, mitotic catastrophe, and eventually cell death.^5,6^ To overcome this vulnerability, many cancer cells rely on HSET to cluster the extra centrosomes into two groups to mimic the bipolar spindle formation of non-centrosome-amplified cells and ensure their survival.^7,8^ In contrast, as previous studies have found, HSET is not required for the assembly of the correct spindle architecture during the mitosis of non-centrosome-amplified cells. Other mitotic kinesins, notably Eg5, act to separate MTOCs and therefore oppose the clustering action of HSET.^3^

Inhibiting HSET could provide a treatment to target tumors with a high content of cells with amplified centrosomes without affecting normal tissue.^3^ In particular, HSET may be a potential target in breast cancers with high centrosome amplification.^9−11^ In addition to its important role in maintaining mitotic spindle integrity in centrosome-amplified cells, HSET is involved in spermatogenesis in mammalian species.^12^ HSET has also been shown to be expressed in nondividing human neurons, where it is implicated in maintaining MT-dependent axon structures.^13^

There is a need to develop and comprehensively characterize potent HSET inhibitors from diverse scaffolds to provide tools for therapeutic research. A small number of HSET inhibitors with cellular activity have been reported.^14−17^ Of these, AZ82^18^ (**1**, Tables 1 and 2, Figure S4), is a potent, reversible inhibitor of the HSET motor domain in biochemical assays, while others have been characterized primarily by their potent cellular activities.^16,17,19^ Kinesin motor proteins present several opportunities for inhibition with small molecules, either through direct or allosteric competition for ATP substrate binding or through interference with microtubule binding.^14^ In view of the multiple ways in which a HSET inhibitory phenotype could be reached in cells, it is desirable to link biochemical and cellular activities through assays demonstrating direct target engagement in both cellular and cell-free systems.^20^ In this work, we describe the discovery of a new HSET inhibitor series from high-throughput biochemical screening and its initial medicinal chemistry optimization to potent cell-permeable inhibitors. In parallel, we show how the compounds were engineered to provide probe molecules to demonstrate HSET binding in vitro and cellular target engagement through the observation of the colocalization of a *trans*-cyclooctene (TCO)-tagged inhibitor and HSET in human cancer cells.

**Table 1:**
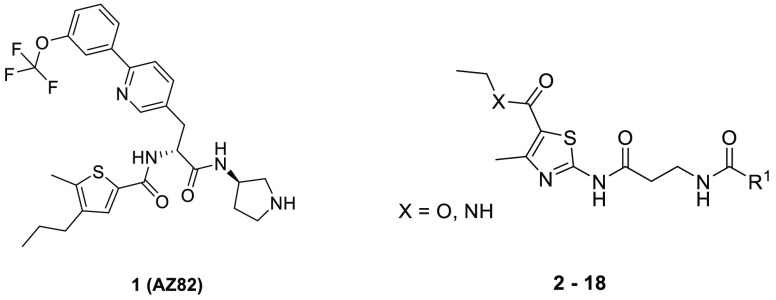

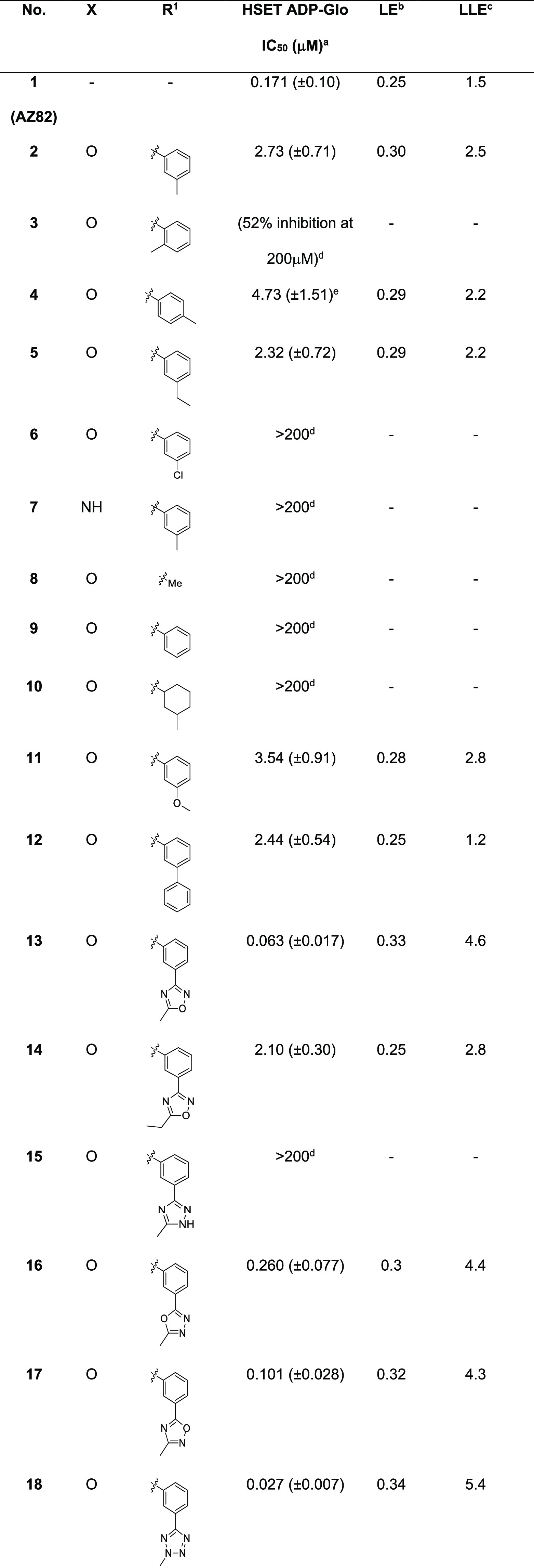
In Vitro Inhibition of HSET and Ligand Efficiencies of **1**–**18**

a Inhibition of recombinant full-length HSET with preformed microtubules and 3 μM ATP measured in ADP-Glo format, mean (±SD) for *n* ≥ 3.

b Ligand Efficacy was calculated using LE = −1.4Log(IC_50_ [M])/number of non-hydrogen atoms.

c Lipophilic Ligand Efficacy was determined using the equation LLE = −Log(IC_50_ [M]) – cLogP, where cLogP was calculated using MoKa from Molecular Discovery.

d From a single determination.

e Inhibition plateaued between 47 and 61%. The mean concentration observed at 50% inhibition was 13 μM.

**Table 2:**
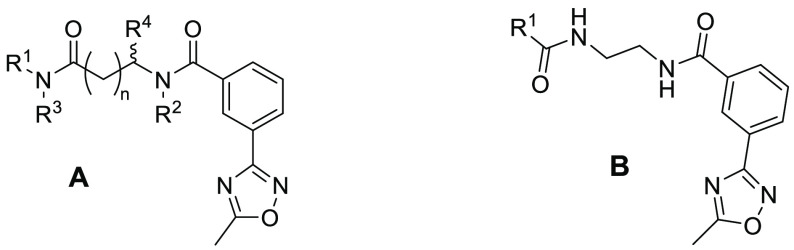

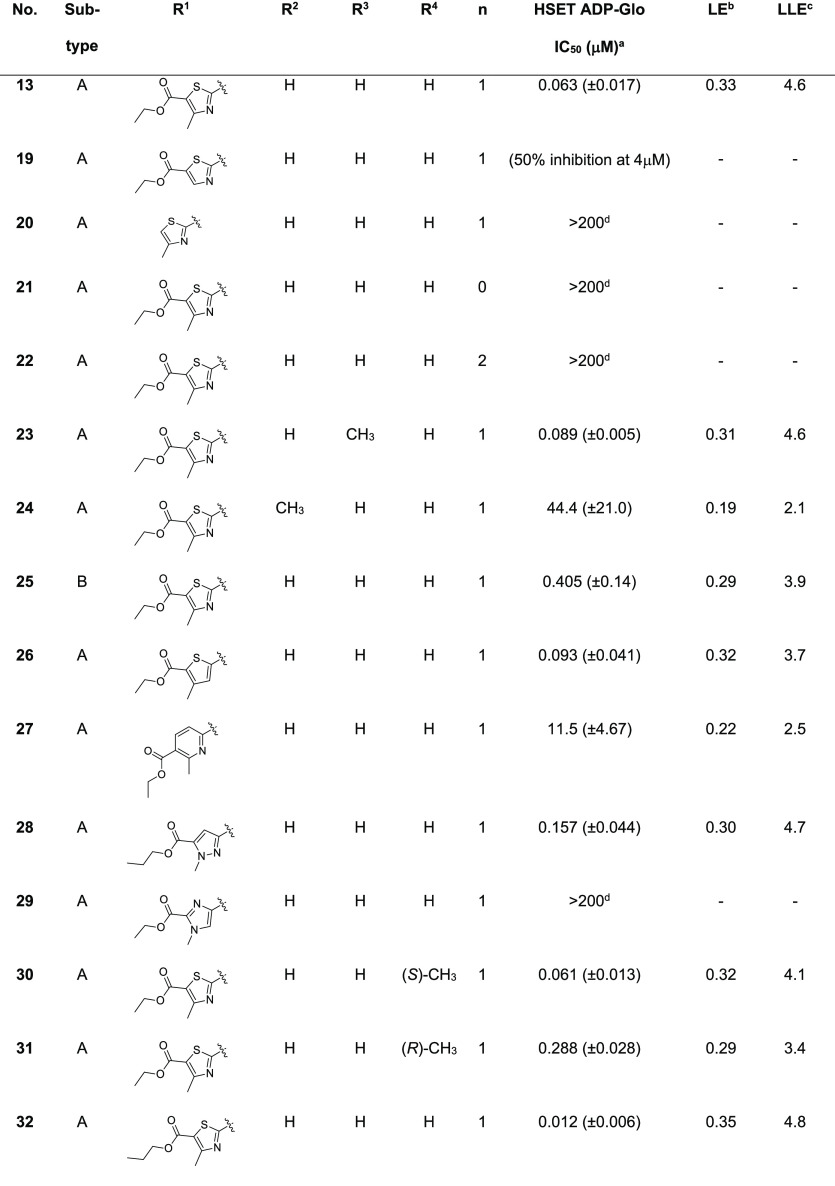
In Vitro Inhibition of HSET and Ligand Efficiencies of **13** and **19**–**32**

a Inhibition of recombinant full-length HSET with preformed microtubules and 3 μM ATP measured in ADP-Glo format, mean (±SD) for *n* ≥ 3 atoms.

b Ligand efficacy was calculated using LE = −1.4Log(IC_50_ [M])/number of non-hydrogen atoms.

c Lipophilic ligand efficacy was determined using the equation LLE = −Log(IC_50_ [M]) – cLogP, where cLogP was calculated using MoKa from Molecular Discovery.

d From a single determination.

## Results and Discussion

A high-throughput screen was carried out using the ADP-Glo format to detect the inhibition of microtubule-stimulated HSET ATPase activity,^21^ from which the commercial compound **2** (CCT341932) (Table 1) was identified as one hit of interest. Both newly purchased and resynthesized batches of **2** confirmed the activity, with a HSET IC_50_ of 2.7 μM. The screening conditions for the ADP-Glo assay used an ATP concentration of 3 μM. When the assay was conducted with a higher ATP concentration of 150 μM, a reduced potency for **2** was observed (HSET IC_50_ of 7.1 μM), suggesting the compound is competitive with ATP. We also examined the potential for **2** to inhibit Eg5, a plus-end-directed mitotic kinesin. Counter-screening against Eg5 is important, as this kinesin opposes the action of HSET in clustering centrosomes and the off-target inhibition of Eg5 may confound the interpretation of cellular effects.^3^ We therefore established an equivalent ADP-Glo assay for microtubule-stimulated Eg5 motor domain activity and demonstrated only 10% inhibition at a top concentration of 200 μM **2**, indicating significant biochemical selectivity for HSET over Eg5. Based on these promising biochemical data and the acceptable ligand efficiency (LE = 0.30) and lipophilic ligand efficiency (LLE = 2.5) of **2**, the compound was taken forward to investigate structure–activity relationships (SARs).

Although crystal structures of the HSET motor domain containing adenosine diphosphate (ADP) are available,^18,22^ none containing a bound HSET inhibitor have been reported. The full HSET protein folded conformation has also been predicted by AlphaFold.^23,24^ Significant conformational flexibility is anticipated in the HSET motor domain by analogy to the known conformational repertoires of other kinesins, for example, Eg5.^25^ Independent docking studies of the previously reported inhibitor AZ82 have suggested two different potential binding poses.^18,22^ Attempts to generate a crystal structure of **2** or close analogues bound to HSET were unsuccessful, and in the absence of a robust structure-based approach, we built a SAR through iterative cycles of design, synthesis, and testing.

Several close analogues (**3**–**6** and **8**–**13**, Table 1) with altered benzamide motifs were quickly synthesized from an advanced intermediate or purchased (**3** and **8**). Although positioning the methyl substituent *para* to the amide linkage in **4** retained activity, the *ortho*-substituted analogue **3** had a seventy-fold reduced potency against HSET. Replacing the 3-methylbenzamide with simple acetamide (**8**), benzamide (**9**), 3-chlorobenzamide (**6**), or the saturated cyclohexane carboxamide (**10**) ablated the activity. We also investigated the replacement of the ethyl ester substituent in **2** by an isosteric amide (**7**) but observed no activity. When the 3-methyl substituent was changed to ethyl (**5**), methoxy (**11**), or phenyl (**12**), similar biochemical activity to the hit **2** was retained, giving confidence that further exploration along this vector was possible.

A scan of more elaborate analogues prepared from commercially available *meta*-substituted benzoic acids pleasingly provided the first submicromolar inhibitor **13**, which displayed improvements in both LE and LLE (Table 1) and enhanced potency over the reference compound **1**. Extending out further from the oxadiazole methyl group proved problematic, with even the ethyl analogue **14** having 33-fold reduced HSET inhibitory activity. Focusing on other five-membered heterocyclic 3-substituents showed that the presence of a hydrogen-bond donor (HBD) in the ring eliminated potency, for example, **15**. The oxadiazole regioisomers **16** and **17** retained similar activity to **13**, and an improvement in activity was noted for the 2-methyl tetrazol-5-yl substituent **18** with a HSET IC_50_ of 27 nM, LE = 0.34, and much increased LLE = 5.4. Subsequent screening of **18** at a higher ATP concentration in the HSET ADP-Glo assays showed a drop in inhibitory activity, suggesting an ATP-competitive mode of action like that of **2** (Table 4). Counter-screening of **13** and **18** confirmed that the gains in HSET activity had not compromised the selectivity against Eg5 (Table 4). While there were some benefits of the 2-methyl tetrazol-5-yl substituent **18**, due to the high hydrogen-bond acceptor (HBA) count, we reverted to the oxadiazole analogues to investigate SARs in other parts of the scaffold.

We investigated the contributions of the thiazole substituents and the flexible alkyl linker to the HSET activity. Removing either the methyl group (**19**) (Table 2) or the ethyl ester (**20**) on the thiazole caused a 65-fold or 3000-fold reduction in potency, respectively. Likewise, shortening (**21**) or lengthening (**22**) the alkyl chain between the amides abolished the HSET activity. The HBD on the amide adjacent to the thiazole **23** could be masked with a methyl group without effecting the potency, while this was not the case for the benzamide **24**, where methylation gave a 700-fold reduction in activity. Here either the HBD appeared important for binding or the *N*-substitution may have a detrimental effect on the ligand conformation. Analogues where the connectivity of this amide bond was reversed also displayed no activity against HSET (compounds not shown). In contrast, the reversal of the amide connectivity adjacent to the thiazole ring **25** was tolerated, although with a six-fold drop in potency. Attempts to replace the thiazole ring with other heteroaromatic scaffolds showed that only small changes were tolerated; for example, the removal of the azole nitrogen gave the isosteric thiophene **26** with comparable activity. It was difficult to replace the thiazole sulfur, and pyridine **27**, a classical isostere for a thiazole, gave a greater than 180-fold reduction in potency. The only non-sulfur-containing five-membered ring with acceptable activity proved to be the pyrazole analogue **28**, but this did not enhance LLE, while other close analogues such as the imidazole **29** showed no HSET inhibition.

The addition of a methyl group to the alkyl chain linking the two aromatics rings introduced a chiral center and interestingly showed a fourfold preference for the (*S*)-enantiomer **30** over the (*R*)-enantiomer **31**, which we would later build on. With the ester substituent seemingly essential for activity, analogues probing the ester alkyl group showed that an extension to a propyl chain **32** gave a fivefold increase in inhibition while maintaining a favorable LLE. Although we had discovered several activity cliffs, we had identified compounds with nanomolar potencies in the biochemical HSET assay. Counter-screening of **26** and **32** confirmed that no Eg5 inhibition had been introduced. Conducting the ADP-Glo HSET assay for these compounds in the presence of an increased ATP concentration (500 μM) showed a drop in inhibitory activity as seen for **2** (see Table 4).

**Table 3:**
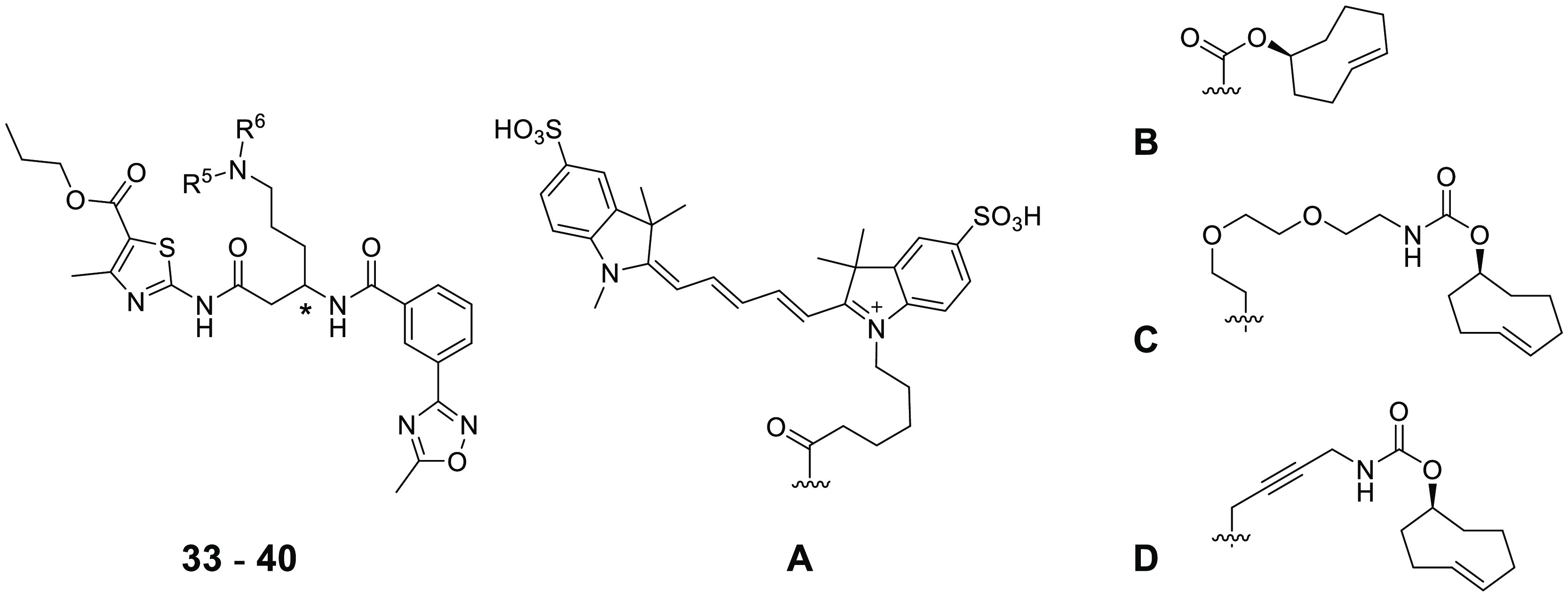
In Vitro Inhibition of HSET and Kinetic Aqueous Solubility Measurements of **33**–**39**

compound	chirality	R^5^	R^6^	HSET ADP-Glo IC_50_ (nM)^a^	*K*_Sol_ data (μM)^b^
**33**	(*S*)	H	H	13 (±3)	>90
**34**	(*R*)	CH_3_	H	260 (±47)	>90
**35**	(*S*)	CH_3_	H	19 (±7)	>90
**36**	(*S*)	CH_3_	CH_3_	11 (±4)	>90
**37**	(*S*)	H	**A**	257 (±70)^c^	n.d.
**38**	(*S*)	CH_3_	**B**	217^d^	1.8
**39**	(*S*)	CH_3_	**C**	107 (±24)	53
**40**	(*S*)	CH_3_	**D**	39 (±18)	1.1

a Inhibition of recombinant full-length HSET with preformed microtubules and 3 μM ATP measured in ADP-Glo format, mean (±SD) for *n* ≥ 3.

b Solubility measured by HPLC with UV detection in PBS buffer at pH 7.4 (100 μM solution with 1% DMSO starting solution). The calibration curve was prepared by injecting 0.5, 2.5, and 5 μL of a 100% DMSO stock solution.

c *n* = 2.

d Single determination.

**Table 4:** Cell and Counterscreening Assay Data for Selected Compounds

compound	HSET ADP-Glo IC_50_ (μM)^a^	HSET ADP-Glo (ATP 500 μM) IC_50_ (μM)^b^	Eg5 ADP-Glo IC_50_ (μM)^c^,^e^	% multipolarity above baseline @ 15 μM (4NCA cells)^f^	% multipolarity above baseline @ 15 μM (4N cells)^g^
**1**	0.171 (±0.10)	0.825^d^	35.6 (36.4, 34.9)		
**2**	2.73 (±0.71)		>200	0%	1%
**13**	0.063 (±0.017)		>200	9%	1%
**18**	0.027 (±0.007)	0.086^c^	>200	11%	0%
**26**	0.093 (±0.041)	0.332^c^	>200	13%	1%
**32**	0.012 (±0.006)	0.051^c^	>200	21%	2%
**35**	0.019 (±0.007)	0.066^c^	>200	11%	1%
**36**	0.011 (±0.004)	0.090^d^	>200	15%	1%

a Inhibition of recombinant full-length HSET with preformed microtubules and 3 μM ATP measured in ADP-Glo format, mean (±SD) for *n* ≥ 3.

b Inhibition of recombinant full-length HSET with preformed microtubules and a high ATP concentration of 500 μM measured in ADP-Glo format.

c Single determination.

d Mean of two results.

e Inhibition of commercially available GST-tagged Eg5 kinesin with preformed microtubules and 4.8 μM ATP measured in ADP-Glo format.

f Multipolar spindle assay (4NCA cell line). The percentage of multipolar mitoses was calculated by dividing the number of multipolar mitoses by the total number of all visible mitoses in one well of a 96-well plate (*n* > 100 for each replicate, 2 replicates per concentration point).

g Multipolar spindle assay (4N cell line). The percentage of multipolar mitoses was calculated by dividing the number of multipolar mitoses by the total number of all visible mitoses in one well of a 96-well plate (*n* > 100 for each replicate, 2 replicates per concentration point).

To explore the behavior of the HSET inhibitors in a cellular context, we developed an assay to compare their effects on the degree of mitotic spindle multipolarity observed in tetraploid (4N) DLD1 human colon cancer cell lines and diploid (2N) DLD1 cells induced to exhibit high centrosome amplification through treatment with dihydro-cytochalasin B (DCB) to transiently block cytokinesis and induce tetraploidisation and centrosome amplification (4NCA).^26,27^ An increase of typically 10% mitotic spindle multipolarity was observed in the centrosome-amplified (4NCA) DLD1 cell line when treated with HSET inhibitors **13**, **18**, and **26** at 15 μM. Importantly, no increases in multipolar mitoses were observed in the non-centrosome amplified DLD1 (4N) cell line at the same concentration (Table 4). Compound **32**, which was more potent in our biochemical assay, showed an increased multipolarity (21%) in the 4NCA cells, again with a minimal effect on the 4N cell line (Table 4, Figure S4). This compound gave an estimated half-life of 215 min in a BALB/c mouse plasma stability assay, which gave us an indication of the enzymatic and hydrolytic stability of the ester moiety. Encouraged that our inhibitors were showing the expected phenotypic effect in cancer cells, we wished to further understand their mechanism by demonstrating their direct interaction with the HSET protein in vitro and to confirm their colocalization and specific binding to HSET in the relevant cellular compartment.

Demonstrating compound colocalization with the intended target in the relevant cellular compartment in cells that are proficient for that target, and not in cells that lack the expression of the intended target, is a desirable step in confirming the mechanism of new inhibitors.^20^ In conjunction with biophysical binding assays, this can demonstrate specific target binding.^28^ A fluorescent tag may be added to a cell-active compound in order to visualize its localization.^29−31^ However, this approach often generates high-molecular-weight probes with poor cell permeability.^31^ To overcome this, one elegant solution is to assemble the fluorescent probe inside the cell using bioorthogonal click-chemistry.^32−34^ Bioorthogonal chemistry has been widely used in chemical biology strategies, including for imaging small molecules in cells, and more than 20 different biorthogonal reactions are well-established in the literature.^32,33,35^ In particular, inverse electron demand Diels–Alders (IEDDA) cycloaddition has gained popularity due to its extremely fast rate (rate constant *k*_2_ ∼ 10^2^–10^6^ M^–1^ s^–1^), high signal-to-noise ratio, and simple reaction conditions.^36,37^ A common variant of IEDDA involves using a tetrazine-tagged fluorescent dye as the electron-poor diene and a strained alkene or alkyne (such as cyclopropene, bicyclononyne, or *trans*-cyclooctene) added to a ligand as the electron rich dienophile.^38^ The *trans*-cyclooctene (TCO) ring has become the strained alkene of choice for this reaction due to its commercial availability and fast reactivity.^39^

We envisaged a TCO probe based on **32** substituted with an alkylamine handle to enable the addition of a linker and the TCO headgroup. Several attachment points on our scaffold for an alkyl linker were considered, though most resulted in an unacceptable loss of HSET inhibitory activity (compounds not shown). However, using the information from **30**, **31**, and **32**, we identified that an alkyl side chain was tolerated on the linker adjacent to the benzamide (Table 3). Starting from the more active (*S*)-methyl-substituted analogue **30**, the length of the substituent was probed, and the aminopropyl analogue **33** provided the best balance of retained potency without the addition of excessive lipophilicity. The enhanced potency of the (*S*)-enantiomer over the (*R*)-enantiomer was confirmed with the secondary amine analogues **34** and **35** (CCT368772). The tertiary amine **36** retained good in vitro potency against HSET, possessed one less HBD, which was beneficial for permeability, and exhibited high kinetic aqueous solubility. Compounds **35** and **36** exhibited similar selectivity over Eg5 and sensitivity to the ATP concentration in the ADP-Glo assay as the progenitor compounds and enhanced multipolar mitotic spindle formation selectively in DLD1 centrosome-amplified cells (Table 4, Figure S4).

**Figure 1:**
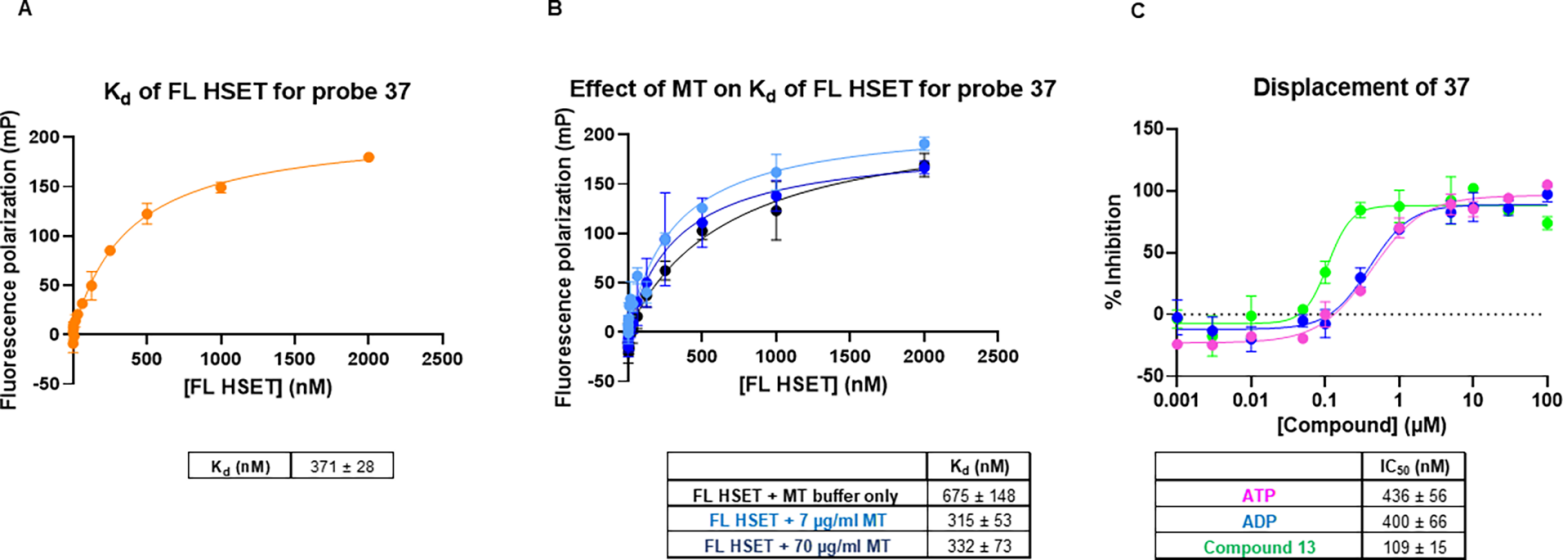
(A) Determination of the binding affinity (*K*_d_) to FL HSET for probe **37** in a fluorescence polarization assay. (B) Binding of **37** to FL HSET is minimally affected by the addition of MTs. (C) ADP, ATP, and compound **13** displace the binding of the FP probe from the FL HSET protein.

Before investigating the cellular probes, we first prepared a fluorescence-tagged analogue of **33** to demonstrate direct binding to HSET in a MT-free environment. Since the ADP-Glo assay measures the MT-stimulated turnover of ATP by HSET, it does not distinguish between compounds that directly bind to HSET to inhibit the dynamic cycle and compounds that interfere with the binding of HSET and MT through interactions with MT binding sites. The sulfoCy5-tagged analogue **37** was shown to inhibit HSET-dependent ATP hydrolysis with moderate activity (Table 3). The affinity of the sulfoCy5-tagged probe for the isolated full-length HSET protein was determined using a fluorescence polarization (FP) assay, which showed saturable binding consistent with a 1:1 stoichiometry (*K*_d_ = 371 nM) (Figure 1a). To determine the effect of MTs on compound binding, a titration of HSET protein was carried out in the presence of MTs at 7 and 70 μg mL^–1^ concentrations (Figure 1b). MT reconstitution buffer alone showed a twofold reduction in the probe affinity for HSET; however, this is within assay variation. In the presence of MTs in reconstitution buffer, minimal effects were seen on the binding affinity of the probe, indicating the compound does not interfere with MT binding. We tested the ability of ATP, ADP, and the unsubstituted compound **13** to displace the binding of FP probe **37** from the isolated HSET protein using a competitive binding FP assay (Figure 1c). Both nucleotide analogues showed the displacement of the probe, as did the parent thiazole **13**. These data confirm that this compound series binds to a site on the HSET protein independent of the presence of MTs and is biochemically competitive with nucleotide binding.

With proof of direct binding to HSET shown in biochemical assays using the fluorescent probe **37**, we sought to adapt the molecule to provide a click-chemistry probe for intracellular target engagement. Three compounds **38**–**40** were synthesized, two of which contained a further extension of the side chain to **35** before the addition of the TCO group in all cases. The impact of the linkers and the TCO ring on the potency and solubility were assessed (Table 4). The directly linked carbamate **38** showed the largest decrease (11-fold) in activity compared to its parent compound. A large decrease in solubility was also observed, which we assumed to be due to the removal of the basic amine in the linker upon the conversion to the carbamate. Distancing the TCO moiety from the core with an extended polyethylene glycol linker in **39** or a more rigid butyne-containing linker in **40** (CCT369834) was found to be beneficial for retaining HSET inhibition. Despite the presence of the amine in **40**, the aqueous solubility was still significantly reduced compared to that of the parent **35**, possibly due to a reduction in the basicity of the propargylamine. In contrast, the addition of the flexible 2-(aminoethoxy)ethoxy chain to the linker in **39** maintained solubility.

**Scheme 1:**
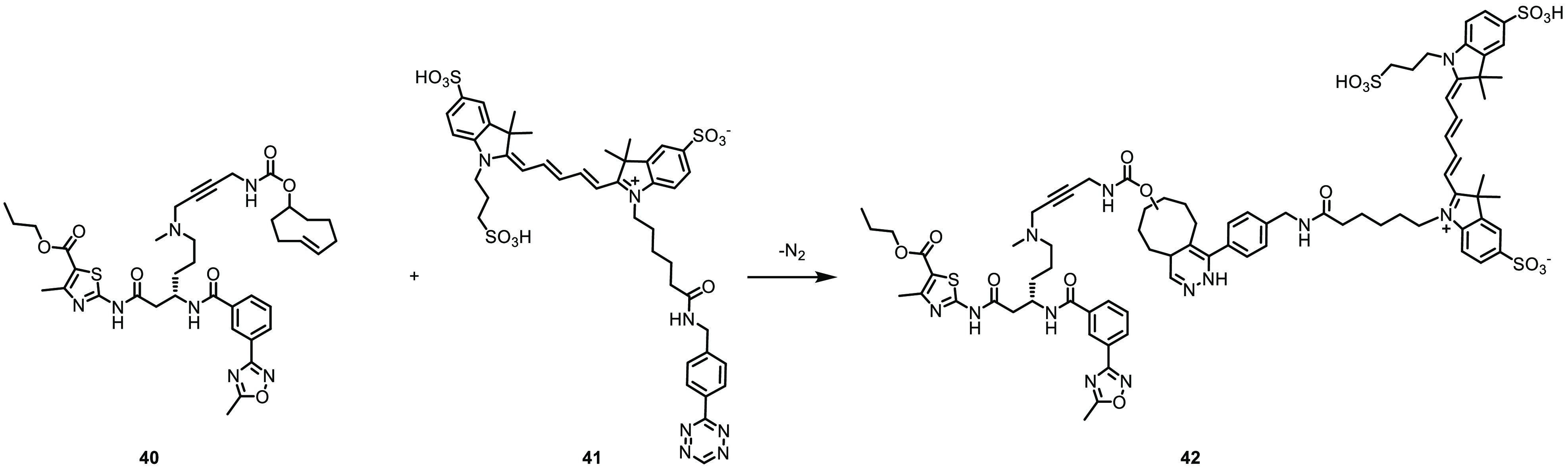
Anticipated IEDDA Reaction between TCO Probe **40** and Tetrazine-Cy5 **41**

Next, the efficiency of the IEDDA reaction between the three TCO probes and [4-(1,2,4,5-tetrazin-3-yl)phenyl]methanamine hydrochloride was investigated (see Figures S1–S3). The TCO probes and the tetrazine were mixed in a 1:2 ratio at room temperature, and the mixtures were analyzed by tandem LC-MS after 5 min. The TCO probes **38** and **40** reacted quickly, with 91% and 84% conversion, respectively. Surprisingly, the reaction with TCO probe **39** gave only 35% conversion after 5 min. We speculate that the potential for the formation of intramolecular H-bonds between the flexible 2-(aminoethoxy)ethoxy chain and the HSET binding core of **39** could render the TCO reactive group less accessible and lead to a lower reaction rate compared to the other two probes. Due to their high affinity for the target and fast reactivity with the model tetrazine, TCO probes **38** and **40** were selected to react with a Cy-5 tetrazine dye (Scheme 1) to explore the target binding in cells.

**Figure 2:**
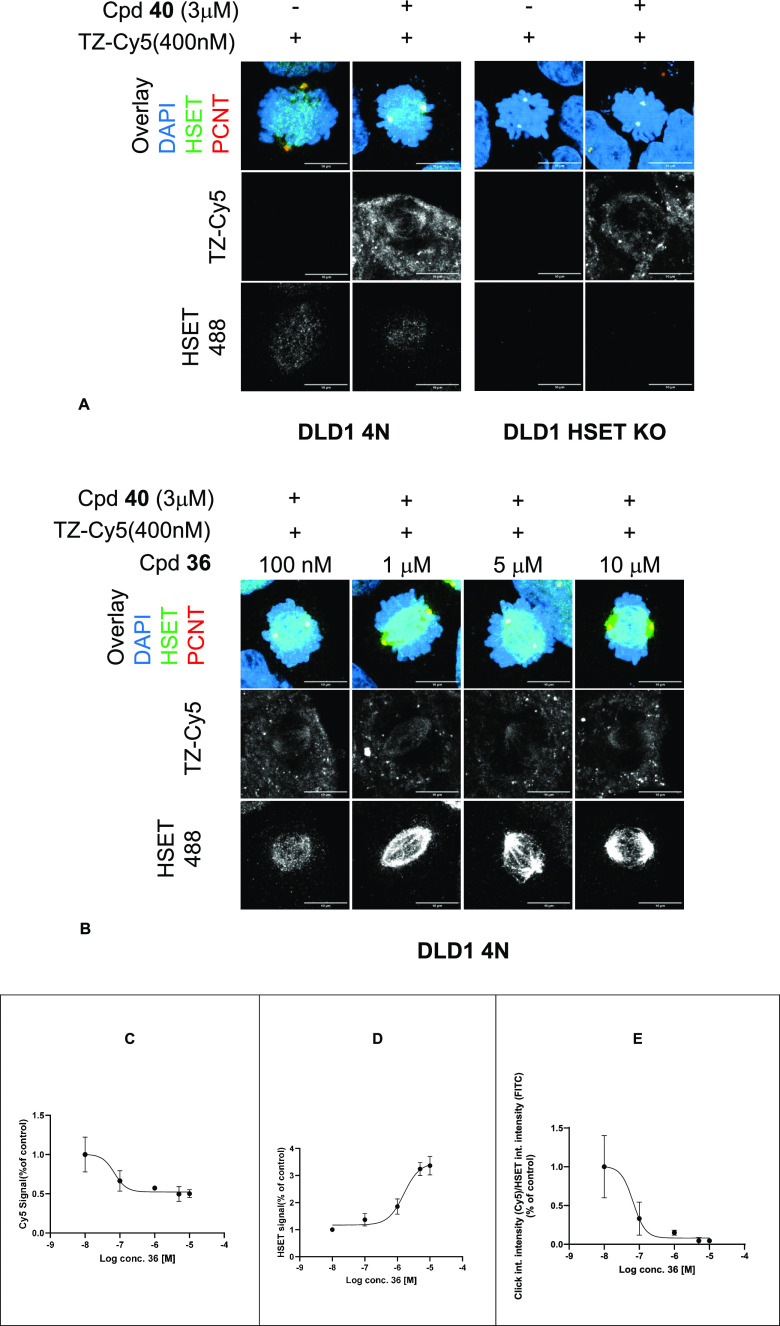
(A) Images showing the distribution of Cy5-induced fluorescence from tetrazine-Cy5 (Txz-Cy5) with and without the addition of TCO probe **40** compared to the HSET intensities identified by indirect immunofluorescence using an Alexa-488 fluorophore in DLD1 4N and DLD1 HSET KO cell lines. The overlay shows the mitotic pole areas identified by staining for pericentrin (red), the HSET intensity as measured by indirect immunofluorescence using an Alexa-488 fluorophore (green) and the nucleus stained with DAPI (blue). (B) Images showing the effects on the fluorescence of increasing the concentration of **36**, which outcompeted the TCO probe **40**. (C) Plot showing the decreasing Cy5 signal intensity measured at the mitotic pole areas (defined by pericentrin staining), normalized to % control, with the increasing concentration of **36**. (D) Plot showing the increasing HSET 488 signal measured at the mitotic pole areas (defined by pericentrin staining), normalized to % control, with the increasing concentration of **36**. (E) Plot showing the ratio of the corrected click probe Cy5 signal intensity (subtracting KO cell signal) the over total HSET protein 488 signal, normalized to % control, with the increasing concentration of **36**.

The Cy-5 tetrazine dye selected for this experiment is not cell-permeable, thus fixation and permeabilization of cells were necessary prior to its addition. Experiments were conducted in DLD1 4N cells and, as a negative control, isogenic 2N DLD1 HSET KO cells in which HSET expression was prevented by the disruption of the *HSET* gene using CRISPR. Cells were treated first with proTAME for 2 h to accumulate mitotic events, followed by treatment with the TCO probe **40** for 45 min. The live cells were then fixed and permeabilized before treatment with the tetrazine-Cy5 dye and incubation to allow the IEDDA reaction to occur. Additionally, the cells were stained for the endogenous HSET protein using indirect immunofluorescence and imaged to observe the fluorescence from both the TCO probe and antibody-labeled HSET. Tetrazine-linked dye concentrations (200–400 nM) and the incubation time (10 min) were identified that did not produce a signal in the absence of a TCO probe. A 3 μM concentration of the probe **40** gave an acceptable signal-to-noise ratio of around 2:1. Even under these optimal conditions, some residual uniform cytoplasmic Cy5 signal was observed within the cells. However, a clear Cy5 decoration of the mitotic spindle was seen, which overlapped with the signal from the antibody-probed endogenous HSET protein (Figures 2A and S6). Importantly, despite the same diffuse background cytoplasmic signal, minimal localization of the dye on the spindle was observed in the negative control DLD1 HSET KO cells. We observed a 14–17-fold increase in signal in DLD1 4N cells treated with **40** relative to tetrazine-Cy5 alone. In comparison, a 7–8-fold increase was observed in HSET nonexpressing cells, indicating this as the level of nonspecific background generated by the labeled probe. Comparing the intensity of click probe labeling coincident with the mitotic spindle in DLD1 4N cells to that in DLD1 HSET KO cells after both were treated with **40**, we observed a twofold higher signal in the former. In an additional optimization of the assay conditions using **38** as the probe, we found that longer washing steps improved the signal/background ratio through better wash-off of the unbound click probe from the cells (Figure S5).

With this refinement in place, we performed competition experiments to displace the TCO probe **40** with increasing concentrations of unlabeled compound **36**. Inhibitor **36** reduced the localization of the TCO probe **40** on the HSET in a dose-dependent manner (Figures 2B and 2C and S6), a direct replication of the probe displacement seen in our biochemical FP assay but in a cellular context. However, even at the highest concentration used for **36** (10 μM), some residual localization of the probe **40** was still observed on HSET. This may be due to the limited solubility of **36** at higher concentrations or the increased localization of HSET on the mitotic spindle in response to binding these HSET inhibitors. Indeed, we observed that as the concentration of the inhibitor increased, the HSET antibody signal was observed to increase on the mitotic spindle toward the mitotic pole in a concentration-responsive manner (Figure 2D). This suggests that the inhibition of HSET by the more potent **36** causes the kinesin to bind more tightly on the microtubule ends, closer to the mitotic pole centrosomes. Therefore, to correctly depict the competitive effect of increasing concentrations of **36**, we plotted the ratio of the corrected click probe Cy5 signal intensity (subtracting KO cell signal) over the total HSET protein 488 signal, which was normalized to % control (Figure 2E)

The tetrazine-Cy5 dye alone did not produce any signal in either HSET-expressing or HSET-nonexpressing cells, while minimal localization of **40** to the mitotic spindle was observed in HSET-nonexpressing cells (Figure 2A). The difference in the amount of inhibitor required to displace **40** from HSET in this target engagement assay to that needed to observe the downstream phenotypic effects suggests that a high threshold of HSET binding on the mitotic pole end of microtubules may be required to induce multipolarity with these inhibitors. More investigation into the underlying mechanism of action is required with inhibitors that are more selective and potent in cells. However, these results showed the colocalization of the labeled probe compound with HSET on the mitotic spindle, which can be displaced by an unlabeled compound, and provided evidence of direct cellular target engagement in cells for this series of HSET inhibitors.

## Conclusion

This work reports the successful progression of a functional biochemical HTS hit (**2**) with micromolar in vitro inhibition of HSET to compounds with nanomolar biochemical potencies and high selectivity against the opposing mitotic kinesin Eg5. A suitable point for the attachment of reporter groups without perturbing HSET inhibition was identified through the development of structure–activity relationships using the functional biochemical assay for MT-stimulated HSET-dependent ATP hydrolysis. The linkers provided the opportunity to introduce basic amines to enhance solubility and mitigate the increased size of the reporter molecules. A fluorescently labeled probe, **37**, directly bound to HSET in the absence and presence of microtubules and confirmed the binding site of the new HSET inhibitors to be located on the HSET protein. Moreover, fluorescent probe binding was competed by the nucleotides ATP and ADP, consistent with the observation that increasing the ATP concentration reduced the inhibitor potency in the HSET-dependent ATP hydrolysis assay. The compounds caused an increase in the formation of multipolar mitotic spindles in dividing aneuploid centrosome-amplified human colon cancer cells, while minimal effects on the frequency of multipolar mitoses were seen in an isogenic non-centrosome-amplified cell line. To confirm target engagement in cells and explore the intracellular localization of the new inhibitors, the substituted analogue **35** was used as a scaffold to design *trans*-cyclooctene-tagged probes. We demonstrated the specific colocalization of HSET and the TCO-tagged probe **40** at the mitotic spindle through an IEDDA click-reaction with a tetrazine-Cy5 dye following the permeabilization of the cells. Furthermore, concentration-dependent competitive displacement of the probe with a non-labeled ligand was achieved, providing an assay for assessing target engagement in cells. These data confirm the potential of the thiazole-derived compounds as novel HSET inhibitors, and future reports will focus on the optimization of cellular activity.

## Chemistry

**Scheme 2:**
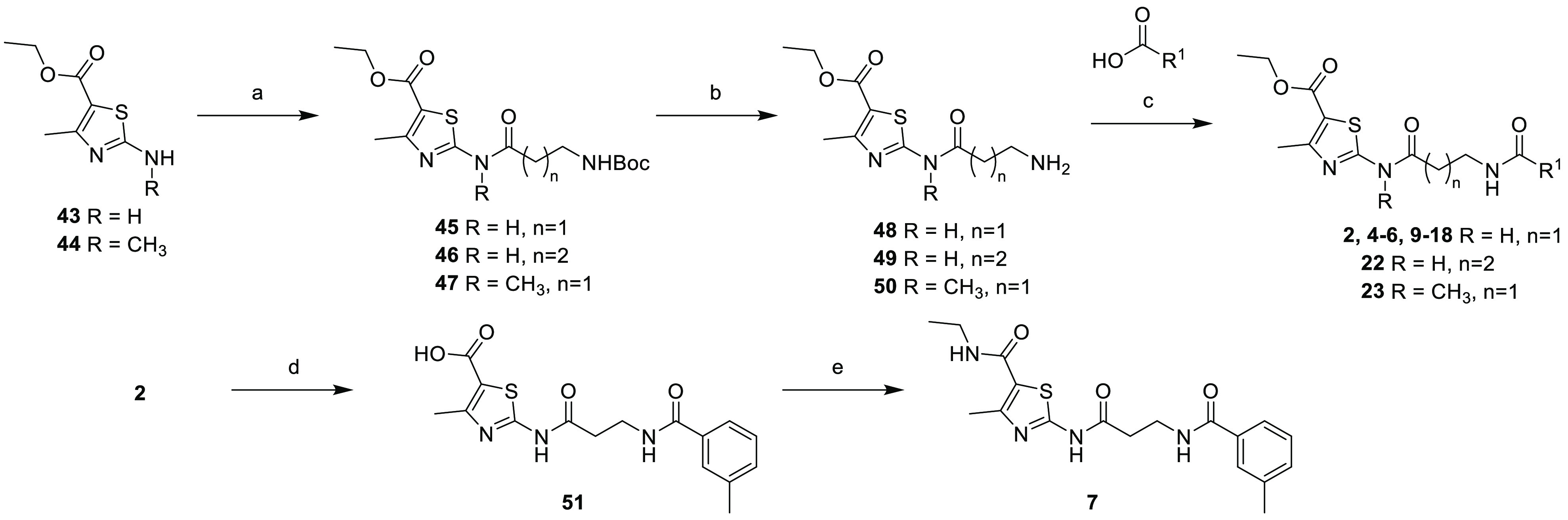
Synthesis of Compounds **2**, **4**–**7**, **9**–**18**, **22**, and **23** Reaction conditions are as follows: (a) Boc-β-Ala-OH (for **45** and **47**) or Boc-GABA–OH (for **46**), HOBt, EDC·HCl, DMF, N_2_, 50 °C, overnight, 80–95%; (b) 4 N HCl in 1,4-dioxane, EtOH, rt, 3 h, 34–97%; (c) R^1^CO_2_H (see Table 1), HATU, DIPEA, DMF, rt, overnight, 7–79%; (d) NaOH, MeOH/H_2_O (2:5), 55 °C, 1 h, 27%; and (e) EtNH_2_·HCl, HOBt, EDC, DIPEA, DMF, rt, 2 h, 43%.

A straightforward synthetic route to access compounds **2**, **4**–**7**, and **9**–**18** was developed (Scheme 2). First, the commercially available ethyl 2-amino-4-methylthiazole-5-carboxylate **43** was coupled with 3-((*tert*-butoxycarbonyl)amino)propanoic acid in the presence of HOBt/EDC in DMF at 50 °C to give ethyl 2-(3-((*tert*-butoxycarbonyl)amino)propanamido)-4-methylthiazole-5-carboxylate **45**. *N*-Deprotection of **45** was performed using 4 N HCl in 1,4-dioxane at room temperature, and subsequent HATU-mediated amide coupling of a range of benzoic acid derivatives afforded compounds **2**, **4**–**6**, and **9**–**18** (Table 1). The homologated example **22** and *N*-methylated derivative **23** were prepared in an analogous manner using 4-(*tert*-butoxycarbonylamino)butanoic acid or ethyl 4-methyl-2-(methylamino)thiazole-5-carboxylate **44**, respectively (Table 2). Example **7** was obtained by reacting **2** with NaOH in MeOH/water at 55 °C for 1 h to give compound **51**. Finally, the amide bond was formed by the sequential addition of HOBt/EDC and ethanaminium chloride in the presence of DIPEA in DMF to give compound **7**.

**Scheme 3:**
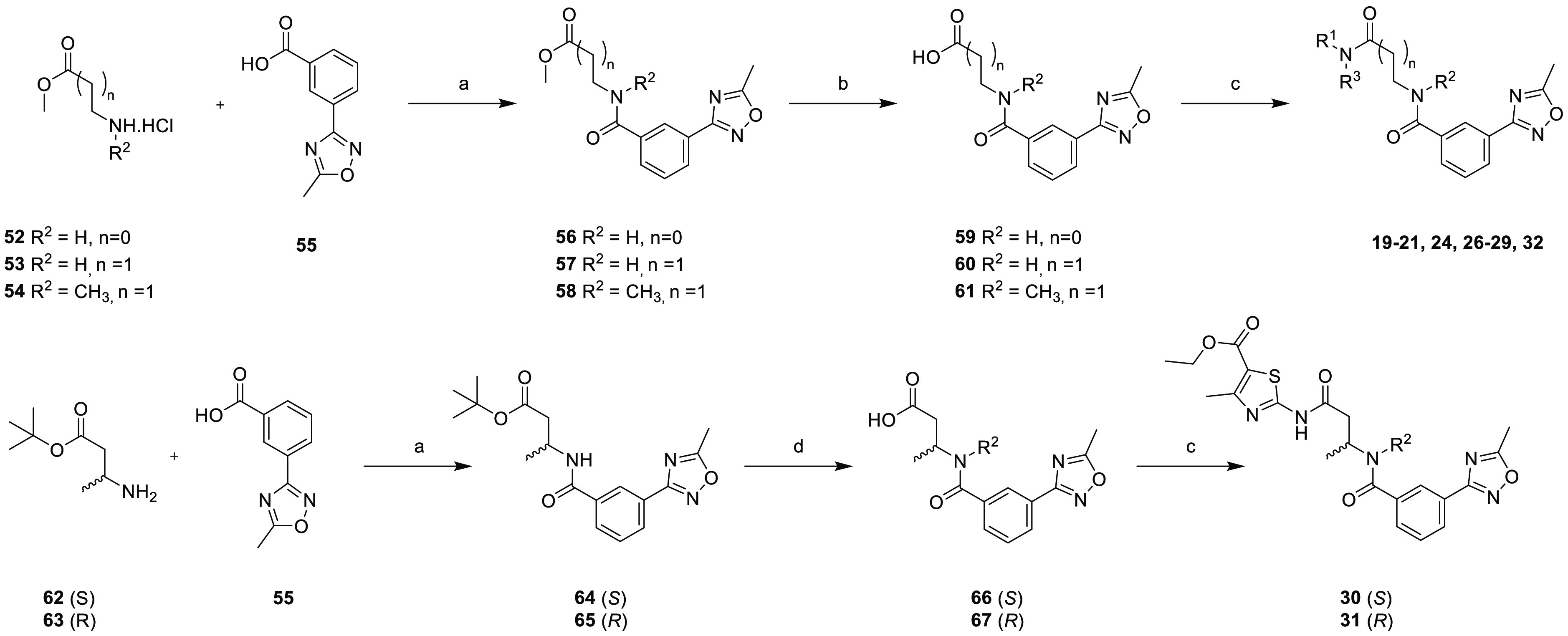
Synthesis of Compounds **19**–**21**, **24**, **26**–**29**, and **32** Reaction conditions are as follows: (a) HATU, DIPEA, DMF, rt, overnight, 81–97%; (b) LiOH·H_2_O, THF/H_2_O (1:1), rt, 1.5 h, 72–97%; (c) R^1^R^3^NH, HOBt, EDC, DMF, 60 °C, 18 h, 5–75%; and (d) TFA, DCM, rt, 2 h, 74–100% over two steps.

An adapted three-step synthetic route permitted the variation and replacement of the thiazole moiety (Scheme 3). To make the amide bond, 3-(5-methyl-1,2,4-oxadiazol-3-yl)benzoic acid **55** was reacted with the corresponding amino acid methyl esters **52**–**54**. Next, saponification of the methyl esters **56**–**58** to the equivalent acids **59**–**61** was performed using aqueous lithium hydroxide. Finally, **19**–**21**, **24**, **26**–**29**, and **32** (Table 2) were prepared by reacting the appropriate amino heterocycles using HOBt/EDC as the coupling agent accompanied by heating at 60 °C for 18 h. A similar synthesis coupled the (*R*)- and (*S*)-enantiomers of *tert*-butyl-3-aminobutanoate to **55** before the removal of the *tert*-butyl group with TFA and gave the acids **66** and **67** for the final amide formation, affording the epimers **30** and **31**, respectively.

**Scheme 4:**
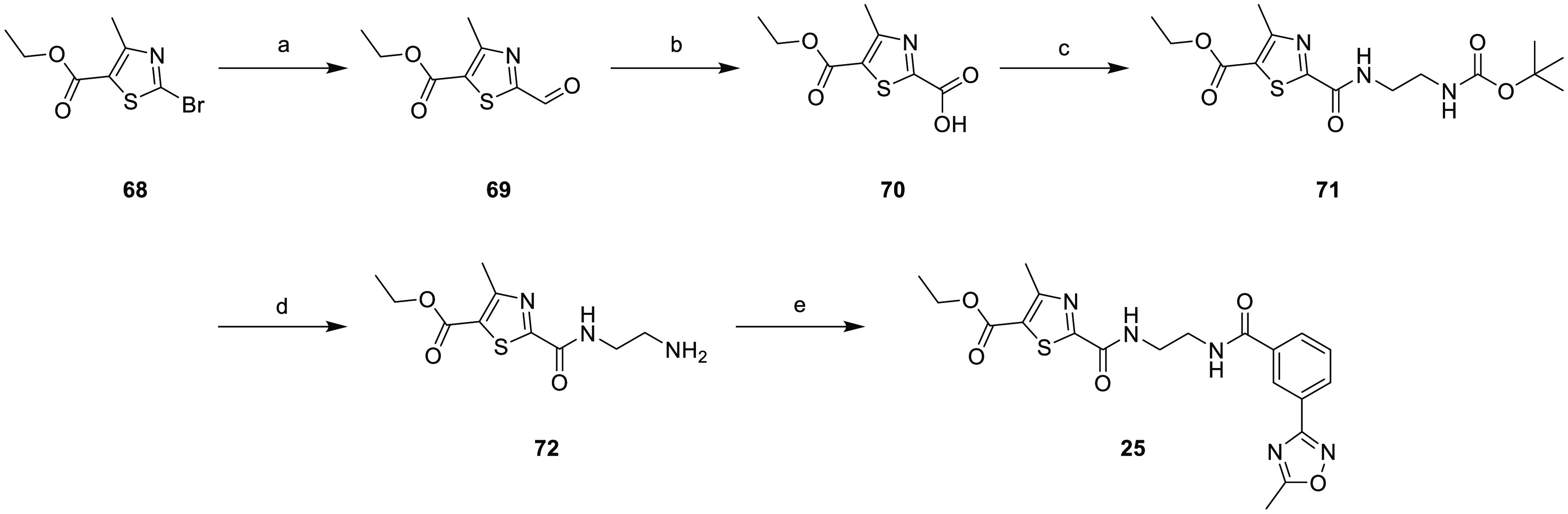
Synthesis of Compound **25** Reaction conditions are as follows: (a) *i*PrMgCl·LiCl, THF, −78 °C, 10 min, then *N*-formylmorpholine, 25 min, 69%; (b) (i) 2-methyl-2-butene, THF, *t*-BuOH, rt, 5 min, (ii) NaH_2_PO_4_, NaClO_2_, H_2_O, rt, 1.5 h, 72% over two steps; (c) *N*-Boc-ethylenediamine, HOBt, EDC.HCl, DMF, rt, 48 h, 37%; (d) 4 N HCl in 1,4-dioxane, 1,4-dioxane, rt, overnight; and (e) 3-(5-methyl-1,2,4-oxadiazol-3-yl)benzoic acid **56**, HOBt, EDC, DMF, rt, overnight, 51% over two steps.

The synthesis was adapted to obtain the reverse amide **25** (Scheme 4). The Grignard reagent of commercially available ethyl 2-bromo-4-methylthiazole-5-carboxylate **68** was prepared by a magnesium-bromide exchange reaction using Knochel’s turbo-Grignard reagent (*i*PrMgCl·LiCl) followed by quenching with *N*-formylmorpholine to afford ethyl 2-formyl-4-methylthiazole-5-carboxylate **69**. This was transformed to the corresponding acid **70** by Pinnick oxidation, and the *N*-Boc-ethylenediamine linker was attached using standard HOBt/EDC·HCl coupling conditions to produce intermediate **71** in moderate yield. *N*-Deprotection of **71** and subsequent HOBt/EDC-mediated coupling with **55** afforded **25** in a 51% yield.

**Scheme 5:**
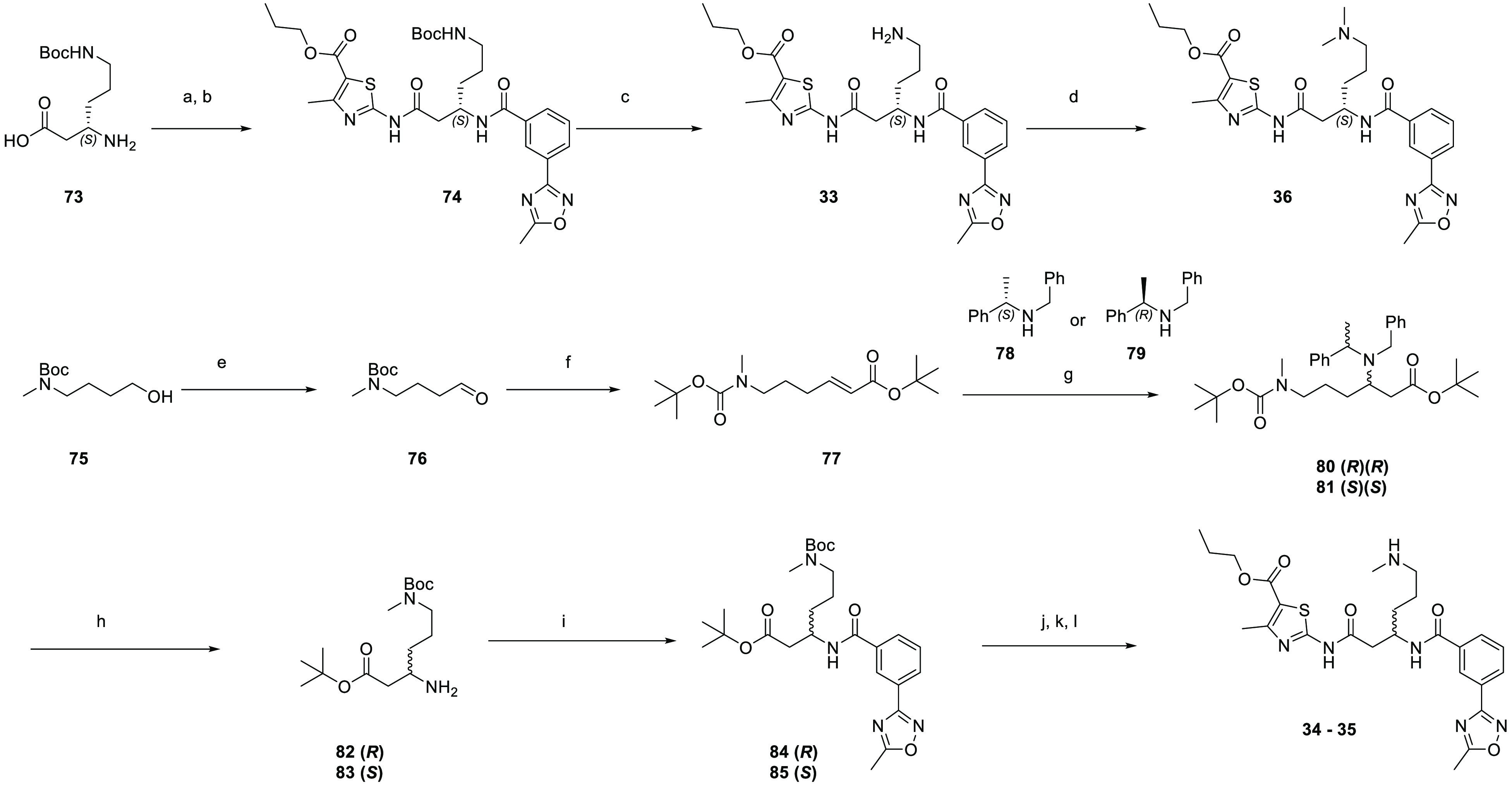
Synthesis of Compounds **33**–**36** Reaction conditions are as follows: (a) **55**, DIPEA, HATU, DMF, rt, 16 h; (b) propyl 2-amino-4-methyl-thiazole-5-carboxylate, EDC·HCl, HOBt, DMF, 60 °C, 18 h, 22% after two steps; (c) HCl in dioxane, propanol, rt, 50 min, 46%; (d) formaldehyde, NaBH(OAc)_3_, AcOH/DCE, rt, 16 h, 34%; (e) DMP, DCM, rt, 2 h; (f) Ph_3_P = CHCO_2_^t^Bu, toluene, 120 °C, 16 h, 70% over two steps; (g) **79** or **80**, ^n^BuLi, THF, −78 °C, 1 h, 42–69%; (h) Pd(OH)_2_, HCO_2_·NH_4_, HCO_2_H, MeOH, 60 °C, 16 h, 51–78%; (i) 3-(5-Methyl-1,2,4-oxadiazol-3-yl)benzoic acid, Et_3_N, T3P, DMF, rt, 2 h, 79–80%; (j) KOH, 50 °C, 5 h, THF/MeOH/H_2_O, 44%; (k) propyl 2-amino-4-methyl-thiazole-5-carboxylate, EDC·HCl, HOBt, DMF, 45 °C, 16 h, 66-80%; and (l) TFA, DCM, rt, 1 h, 74%–80%.

The synthesis of **33** (Table 3) was achieved in three steps using commercially available chiral amine **73**, which was subjected to two successive amide coupling steps and the removal of the Boc protecting group (Scheme 5). A double reductive amination with formaldehyde and sodium triacetoxyborohydride gave **36** in a modest yield. To access **34** and **35**, the appropriate chiral linker containing a Boc-protected secondary amine was made in four steps from **75** using a chiral aza-Michael addition as the key step.^40^ Linker component **83** was obtained with a 97:3 e.r. as determined by ^1^H NMR after derivatization using Mosher’s acid (see the Supporting Information). Coupling the appropriate benzoic acid **55** to the chiral amino linker using propanephosphonic acid anhydride (T3P) gave **85.** Hydrolysis of the ester and amide coupling with EDC and HOBt followed by the removal of the Boc group with TFA gave **35**. A similar sequence was employed to obtain the epimer **34** from **82**.

**Scheme 6:**
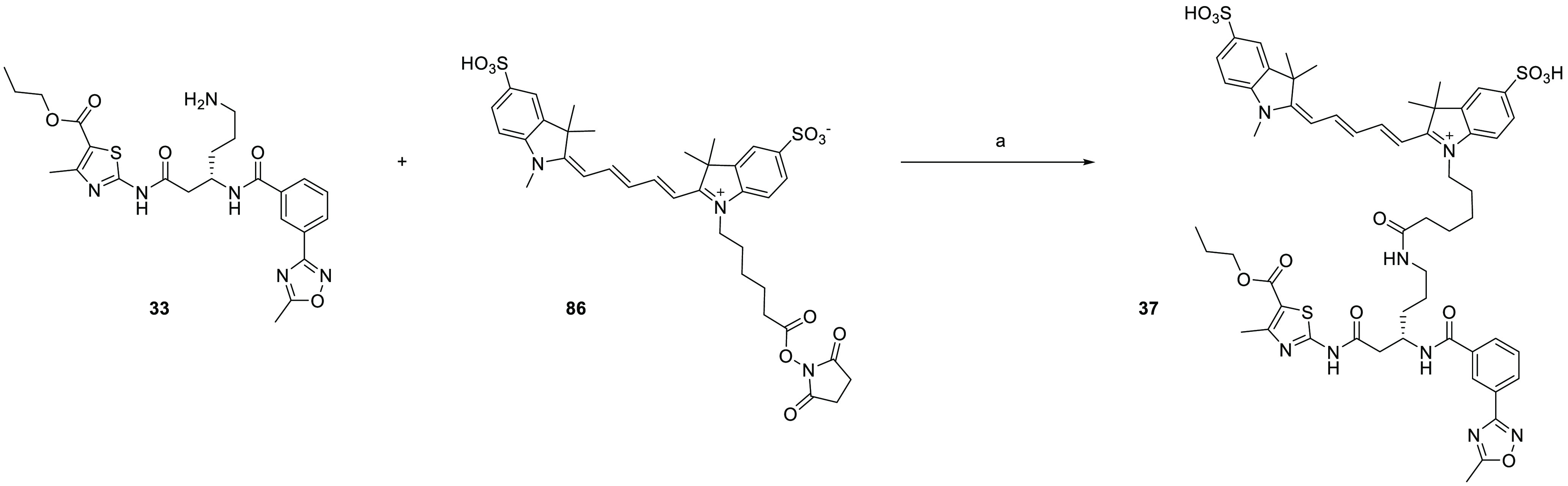
Synthesis of FP Probe **37** Reaction conditions are as follows: (a) TEA, DMF, rt, 16 h, 62%.

The FP probe was synthesized by reacting the primary amine on **33** with commercially available sulfoCy5-NHS ester **86** (Scheme 6).

**Scheme 7:**
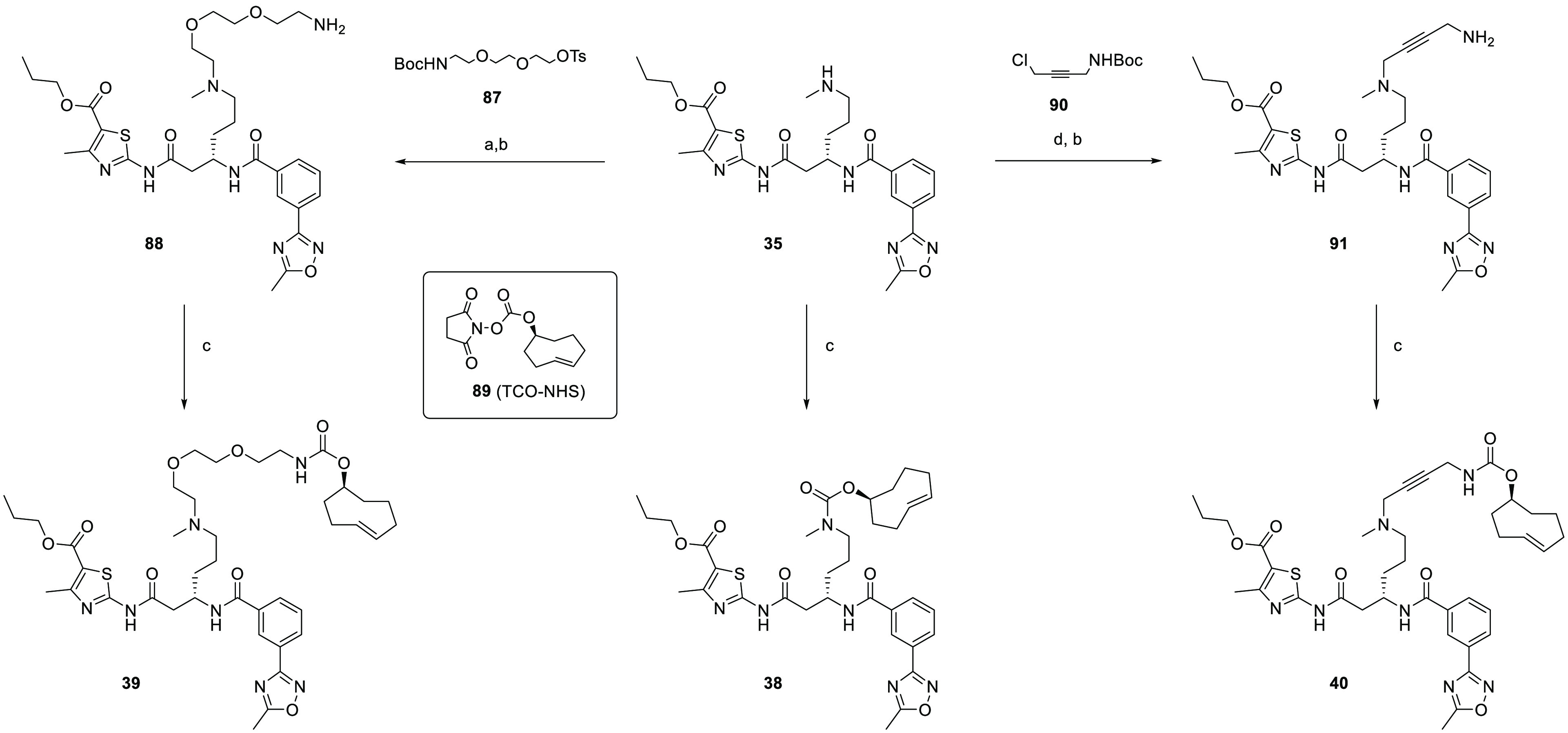
Synthesis of TCO Probes **38**–**40** Reaction conditions are as follows: (a) **87**, DMF, rt, 7 d, 64%; (b) HCl in dioxane, rt, 4–6 h, 44–65%; (c) **89**, DIPEA, DMF, rt, 16 h, 67–96%; and (d) **90**, DMF, rt, 24 h, 46%.

From compound **35**, the synthesis of the TCO probes began with the direct addition of the *trans*-cyclooctene ring with a carbamate linkage using commercially available TCO-NHS carbonate (Scheme 7) to yield **38**. Other TCO probes **39** and **40** were synthesized by incorporating either an additional flexible 2-(aminoethoxy)ethoxy chain or a more rigid 1-amino-but-2-yne into the linker. The Boc-protected amino linkers with a suitable leaving group were reacted with the secondary amine of **35**. Removal of the Boc protecting group with HCl gave free amines **88** and **91**, which were progressed to the final carbamate formation with TCO-NHS **89** to give compounds **39** and **40**, respectively.

## Experimental Section

### ADP-Glo HSET (3 μM ATP), ADP-Glo HSET (500 μM ATP), and ADP-Glo Eg5 Assays

The HSET or Eg5 ATPase activity was measured using the ADP-Glo Kinase Assay kit (Promega, V9102). The assays were run using final concentrations of 5 nM full-length human N-terminal His-tag HSET or 4 nM GST-tagged Eg5 motor domain protein (Cytoskeleton, EG01) and an assay buffer containing 20 mM HEPES pH 6.8, 10 mM MgCl_2_, 0.25 mM EGTA, 0.4 mM Triton X-100, and 1 mM DTT. Preformed microtubules (Universal Biologicals Cambridge, bovine MT001-XL, or porcine MT002-XL), which were reconstituted in 15 mM PIPES pH 7, 1 mM MgCl_2_, and 20 μM paclitaxel, were present at a final assay concentration of 70 μg/mL. A reaction volume of 5 μL was used in Proxiplate 384 plus white assay plates (PerkinElmer, 6008280). To measure compound inhibition, 100 nL of compound dissolved in DMSO or DMSO alone was preincubated with the HSET and microtubules for 10 min before 3 μM Ultra-Pure ATP was added to start the ATPase reaction for the 3 μM ATP HSET assay. For the 500 μM ATP HSET assay, the addition of 500 μM Ultra-Pure ATP was used. For the Eg5 ATPase assay, 4.8 μM Ultra-Pure ATP was used. All reactions were incubated at 25 °C for 80 min and then stopped by the addition of 5 μL of the ADP-Glo reagent. 10 μL of Kinase Detection Reagent was then added after a further 45 min of incubation. The luminescence was read after 45 min on a PheraStar FSX plate reader (BMG Labtech). The data processing was performed using Dotmatics Studies software package. Percentage inhibition was determined based on normalization to high controls (0% inhibition, all reagents with 100 nL of DMSO alone) and low controls (100% inhibition as a high control, without HSET). The IC_50_ values for each compound were determined using a four-parameter logistic curve fit of % inhibition versus concentration.

### Fluorescence Polarization Assays

Steady-state fluorescence polarization binding assays were performed using the fluorescence-tagged probe **37**. All assays were performed in a black ProxiPlate-384 Plus plate (PerkinElmer, 6008260) in a 10 μL volume in buffer containing 20 mM HEPES pH 7.5, 200 mM NaCl, 1 mM TCEP, 10 mM MgCl_2_, 0.1 mM Triton X-100, and 5% glycerol (v/v). Full-length human N-terminal His-tag HSET was used, with a final concentration of 2.5 nM **37** probe. The assays were sealed and incubated in the dark at 25 °C and then the FP signal was read on an EnVision multimode plate reader (PerkinElmer Life Sciences) using excitation at 620 nm and parallel and perpendicular emissions at 688 nm. All data processing was done using Prism (GraphPad Software).

For the probe *K*_d_ determinations, FL HSET final concentrations were used between 2000 and 0.5 nM. When present, preformed microtubules (Universal Biologicals Cambridge, bovine MT001-XL or porcine MT002-XL), which were reconstituted in 15 mM PIPES pH 7, 20 μM paclitaxel, or reconstitution buffer alone, were preincubated with HSET for 10 min and then added to the probe. The plate was read after 3 h of incubation in the dark at 25 °C. *K*_d_ values were determined using a 1:1 site binding model of the fluorescence polarization versus protein concentration.

For the competition assays, 100 nL of compound dissolved in DMSO or DMSO alone was added to create 11 pt concentration response curves between 100 and 0.001 μM final concentrations. This assay used 400 nM FL HSET, and the plate was read after 2 h of incubation in the dark at 25 °C. IC_50_ values were determined as for the ATPase assay using a four-parameter statistical logistic curve fit.

### DLD1 Cellular Models

The cellular models consisted of chromosomally stable human colon cancer DLD1 diploid (2N) and tetraploid (4N) cells, along with tetraploid centrosome -amplified (4NCA) DLD1 cells and DLD1 HSET knockout (KO) cells. DLD1 2N and 4N cells were generated and characterized as previously described.^27,41,42^ In order to generate 4NCA cells, we used dihydro-cytochalasin B (DCB) to transiently block cytokinesis and induce tetraploidisation and centrosome amplification in DLD1 cells. Centrosome amplification in 4NCA cells is transient and therefore they were generated from 2N cells for each run of the assay.^27^ The DLD1 HSET KO cells were knockout clones generated by CRISPR and validated by sequencing to have a homozygous deletion in the *KIFC1* gene.

#### Multipolarity Assay

Inhibition of HSET-mediated clustering was measured using a phenotypic assay measuring the formation of multipolar metaphase spindles in DLD1 cells with (4NCA) or without (4N) centrosome amplification. 4NCA cells were generated by treating 2N DLD1 cells with 2 μΜ DCB for 24 h, washed, and released in growth media for 24 h. 4N and 4NCA DLD1 cells were plated in 96-well ibidi μ-Clear plates (IB-89626, ibidi) with a no. 1.5 polymer coverslip bottom with optimal imaging properties. To enrich the mitotic population, cells were first treated for 3.5 h with 8 μM pro-TAME (I-440-01M, R&D system), an inhibitor of APC/C that transiently arrests cells in metaphase without affecting the structure of the spindle. Following the initial arrest, cells were treated with HSET compounds in the presence of 8 μM MG-132 (S2619, SelleckChem), a proteasome inhibitor that helps to maintain the metaphase arrest, where the number of spindle poles/cell can be optimally quantified by an automated segmentation analysis.

Cells were then fixed in methanol and stained for 3 h for mitotic pole marker aurora A (1:1000 610939 mouse anti-IAK1, BD Biosciences) and phosphorylated histone H3 (1:1500 ab47297 anti-histone H3 pS10, Abcam) as a marker of mitotic cells in 1.5% FBS in PBS. Following primary antibody treatment, cells were incubated with secondary antibodies Alexa Fluor 488 goat antimouse and Alexa Fluor 555 goat antirabbit (1:1500, A11029 and A21429, respectively, Life Technologies) together with 1 μg/mL DAPI (D9542, Sigma) in 1.5% FBS in PBS. Image acquisition was performed at 10× using the GE INCELL 2200 High Content Imaging System (Cytiva) and analyzed using the InCell Investigator software. Mitotic cells were identified by high pHH3 staining and were scored as multipolar if they had more than two mitotic spindle poles. The percentage of multipolar mitoses was calculated by dividing the number of multipolar mitoses by the total of all visible mitoses in one well of a 96-well plate (*n* > 100 for each replicate, two replicates per concentration point).

#### Click Probe Localization Target Engagement Assay

Direct target engagement of active inhibitor **36** was measured by calculating its capacity to displace the TCO-containing probe **40**. 4N DLD1 cells and DLD1 HSET KO cells (negative control) were plated in 96-well ibidi μ-Clear plates (IB-89626, ibidi). Cells were first treated for 2 h with 8 μM pro-TAME (I-440–01M, R&D system) to accumulate mitotic cells and were then treated with 8 μM MG-132 (S2619, SelleckChem) and 3 μM **40**, alone or together with active inhibitor **36** in DMEM media with 1% FBS for 45 min. Cells were then washed three times in DMEM, with the second wash being incubated for 10 min to ensure the optimal reduction of unspecific compound binding. After an additional PBS wash, cells were then fixed in 4% PFA/PBS (15670799, Thermo Scientific) prior to incubation with 400 nM tetraszine-Cy5 (CLK-015-05, Jena Biosciences) in 1.5% FBS/PBS for 10 min at room temperature. Cells were permeabilized by the addition of 0.5% TritonX-100/PBS for 5 min before being stained overnight for Pericentrin (1:1500 ab28144 mouse anti-PCNT, Abcam) and KIFC1 (1:1500 12313S rabbit anti-KIFC1, Cell Signaling Technologies). Secondary antibodies Alexa Fluor 488 donkey antirabbit and Alexa Fluor 555 donkey antimouse (1:1500, A21206 and A31570, respectively, Life Technologies) were added together with 1 μg/mL DAPI (D9542, Sigma) in 1.5% FBS in PBS.

Plates were imaged at 40× using a Zeiss LSM980 confocal microscope with Airyscan 2. The subsequent analysis and quantification were performed using 3D image segmentation with the Arivis4D software. Mitotic cells were segmented by selecting for the highest density (integrated DAPI intensity/total volume), and the mitotic pole areas were segmented using the Pericentrin signal. The signal of the click probe (Cy5) and total KIFC1 (FITC) was measured on the mitotic pole area, and both were normalized to the control condition containing **40** probe alone. The ratio of probe per target (click probe pole integrated intensity signal/KIFC1 pole integrated intensity signal) was used to calculate the occupancy of the probe in the target protein and normalized to the control condition containing **40** probe alone (*n* > 100 for each replicate, two replicates per concentration point).

#### In Silico Chemistry

cLogP calculations were performed using MoKa from Molecular Discovery.

#### General Synthetic Chemistry

Reactions were carried out under N_2_. Organic solutions were dried over MgSO_4_ or Na_2_SO_4_. Starting materials and solvents were purchased from commercial suppliers and were used without further purification. Reactions heated by microwave irradiation were carried out using a Biotage Initiator microwave reactor. Ion-exchange chromatography was performed using ISOLUTE Flash SCX-II (acidic) or Flash NH2 (basic) resin cartridges. Silica column chromatography was performed using Biotage SP1 or Isolera medium-pressure chromatography systems using prepacked silica gel cartridges (normal phase (NP), Biotage SNAP KP-Si; reverse phase (RP), Biotage SNAP Ultra C18). Preparative high-performance liquid chromatography (HPLC) was carried out at rt using a 1200 series preparative HPLC (Agilent, Santa Clara) over a 15 min gradient elution from 60:40 to 0:100 water/MeOH (both modified with 0.1% formic acid) at a flow rate of 5, 20, or 40 mL/min depending on the column size used. Standard injections of 500 μL to 2 mL (with needle wash) of the sample were made onto ACE 5 C18-PFP columns (5 μm, 250 × 10/250 × 21.2/250 × 30 mm^2^, Advanced Chromatography Technologies, Aberdeen, U.K.). UV–vis spectra were acquired at 254 nm on a 1200 Series Prep Scale diode array detector (Agilent, Santa Clara).

NMR spectra were recorded on Bruker AMX500 or AV600 instruments using internal deuterium locks. Chemical shifts (δ) are reported relative to tetramethylsilane (δ 0) and/or referenced to the solvent in which they were measured. Compounds were assessed for purity by tandem HPLC–MS. Combined HPLC–MS analyses were performed using an Agilent 6210 time-of-flight (ToF) HPLC–MS with a Merck Chromolith Flash column (RP-18e, 25 × 2 mm), a Waters Xevo G2QToF HPLC–MS, or an Agilent 1260 Infinity II UPLC-MS with either a Phenomenex Kinetex C18 column (30 × 2.1 mm, 2.6 μm, 100 Å) or a Agilent Poroshell C18 column (30 × 2.1 mm, 2.6 μm, 100 Å). Analytical separation was carried out at 30 °C (40 °C for Agilent 6210 2 min run) with UV detection at 254 nm, and ionization was performed using positive-ion electrospray. The mobile phase was a mixture of MeOH (solvent A) and water (solvent B), both of which contained formic acid at 0.1%. Standard 2 min runs: Agilent 6120, gradient elution 5:95 (A/B) to 100:0 (A/B) over 1.25 min, 100:0 (A/B) for 0.5 min, reversion back to 5:95 (A/B) over 0.05 min, finally 5:95 (A/B) for 0.2 min; Agilent 1260 and Xevo, gradient elution 10:90 (A/B) to 90:10 (A/B) over 1.25 min, 90:10 (A/B) for 0.5 min, reversion back to 10:90 (A/B) over 0.15 min, finally 10:90 (A/B) for 0.1 min. HRMS 4 min runs: Agilent 6120:5:95 (A/B) to 100:0 (A/B) over 2.5 min, 100:0 (A/B) for 1 min, reversion back to 5:95 (A/B) over 0.1 min, finally 5:95 (A/B) for 0.4 min; Xevo, 10:90 (A/B) to 90:10 (A/B) over 3 min, 90:10 (A/B) for 0.5 min, reversion back to 10:90 (A/B) over 0.3 min, finally 10:90 (A/B) for 0.2 min; Agilent 1260, 10:90 (A/B) to 90:10 (A/B) over 2.5 min, 90:10 (A/B) for 1 min, reversion back to 10:90 (A/B) over 0.3 min, finally 10:90 (A/B) for 0.2 min. Flow rates for over 2 min runs: Agilent 6120, 1.5 mL/min; Xevo, 0.5 mL/min; Agilent 1260, 0.6 mL/min. Flow rates for 4 min: Agilent 6120, 0.75 mL/min; Xevo, 0.3 mL/min; Agilent 1260 0.4 mL/min.

Biologically evaluated compounds gave >95% purity as determined by these methods (see Supporting Information).

5-Methyl-6N-((*R*)-1-oxo-1-((*R*)-pyrrolidin-3-ylamino)-3-(6-(3-(trifluoromethoxy)phenyl)pyridin-3-yl)propan-2-yl)-4-propylthiophene-2-carboxamide [AZ82] (**1**) was purchased from Sigma-Aldrich (U.K.). Ethyl 4-methyl-2-(3-(2-methylbenzamido)propanamido)thiazole-5-carboxylate (**3**) and ethyl 2-(3-acetamidopropanamido)-4-methylthiazole-5-carboxylate (**8**) were purchased from Enamine (Kyiv, Ukraine).

##### Ethyl 2-[3-(*tert*-Butoxycarbonylamino)propanoylamino]-4-methyl-thiazole-5-carboxylate (**45**)

To a stirred solution of 3-((*tert*-butoxycarbonyl)amino)propanoic acid (2 g, 10.57 mmol), and HOBt (3.24 g, 21.14 mmol) in dry DMF, under a nitrogen atmosphere at room temperature, were added EDC (4.05 g, 21.14 mmol) and ethyl 2-amino-4-methylthiazole-5-carboxylate (2.165 g, 11.63 mmol) sequentially. The mixture was stirred overnight at 50 °C. The mixture was concentrated in vacuo. The crude product was dissolved in EtOAc and washed with water (×1), 1 N HCl (×1), aq. sat. bicarb. (×1), and brine (×1). The organic layer was dried over sodium sulfate and concentrated in vacuo to give **45** (3.428 g, 9.59 mmol, 91% yield) as a yellow solid. ^1^H NMR (500 MHz, DMSO-*d*_6_) δ 12.45 (s, 1H), 6.90 (t, *J* = 5.7 Hz, 1H), 4.23 (q, *J* = 7.1 Hz, 2H), 3.23 (q, *J* = 6.6 Hz, 2H), 2.59 (t, *J* = 6.9 Hz, 2H), 2.53 (s, 3H), 1.36 (s, 9H), 1.27 (t, *J* = 7.1 Hz, 3H). ^13^C NMR (126 MHz, DMSO) δ 170.94, 162.61, 159.99, 156.55, 155.94, 114.23, 78.15, 60.92, 36.41, 36.06, 28.67, 17.49, 14.66. HPLC/MS (ESI): *m*/*z* 358.1431 [M + H]^+^. *R*_t_ (2 min): 1.32 min.

##### Ethyl 2-(3-Aminopropanoylamino)-4-methyl-thiazole-5-carboxylate (**48**)

Ethyl 2-[3-(*tert*-butoxycarbonylamino)propanoylamino]-4-methyl-thiazole-5-carboxylate **45** (1.22 g, 3.41 mmol) was dissolved in EtOH (34 mL), and to the mixture was added 4 N HCl in dioxane (17 mL, 68.27 mmol) dropwise while stirring at room temperature. The mixture was stirred 3 h at rt. The mixture was concentrated in vacuo. The residue was dissolved in EtOAc and washed with aq. sat. bicarb. (×1) and brine (×1). The aqueous layer was back-extracted with EtOAc (×2). The combined organic layers were dried over sodium sulfate and concentrated in vacuo to give **48** (849 mg, 97%, 3.30 mmol) as an off-white solid. The product was used as such in the next step. HPLC/MS (ESI): *m*/*z* 258.0909 [M + H]^+^. *R*_t_ (2 min): 0.84 min.

##### Ethyl 4-Methyl-2-(3-(3-methylbenzamido)propanamido)thiazole-5-carboxylate (**2**)

To 3-methylbenzoic acid (38.1 mg, 0.280 mmol), ethyl 2-(3-aminopropanoylamino)-4-methyl-thiazole-5-carboxylate **48** (80 mg, 0.311 mmol), and DIPEA (163 μL, 0.933 mmol) in DMF (3.1 mL) was added HATU (110 mg, 0.466 mmol), and the reaction mixture was stirred overnight at rt. The mixture was concentrated in vacuo. The crude was dissolved in EtOAc and washed with 1 N HCl (×1), aq. sat. bicarb. (×1), and brine (×1). The organic layer was dried over sodium sulfate and concentrated in vacuo and purified by reverse phase column chromatography (eluent: 0–80% methanol/water, +0.1% formic acid in each) to afford **2** (83 mg, 0.221 mmol, 71% yield) as a colorless solid. ^1^H NMR (500 MHz, DMSO) δ 12.51 (s, 1H), 8.55 (t, *J* = 5.6 Hz, 1H), 7.65–7.62 (m, 1H), 7.62–7.58 (m, 1H), 7.34–7.31 (m, 2H), 4.23 (q, *J* = 7.1 Hz, 2H), 3.60–3.51 (m, 2H), 2.75 (t, *J* = 6.9 Hz, 2H), 2.53 (s, 3H), 2.34 (s, 3H), 1.27 (t, *J* = 7.1 Hz, 3H). ^13^C NMR (126 MHz, DMSO) δ 171.04, 166.86, 162.60, 160.04, 156.57, 137.94, 134.80, 132.16, 128.60, 128.18, 124.76, 114.25, 60.92, 35.77, 35.62, 21.40, 17.49, 14.67. HPLC/HRMS (ESI): *m*/*z* calculated for C_18_H_22_N_3_O_4_S^+^ [M + H]^+^ 376.1326, found 376.1328. *R*_t_ (4 min): 2.80 min.

##### Ethyl 4-Methyl-2-(3-(4-methylbenzamido)propanamido)thiazole-5-carboxylate (**4**)

Prepared as described for **2** using 4-methylbenzoic acid (28.6 mg, 0.210 mmol) and **48** (60 mg, 0.233 mmol). The material isolated after column chromatography was further purified by SCX-II ion exchange chromatography. Yield: 25 mg (0.067 mmol, 28.6%) pale yellow solid. ^1^H NMR (500 MHz, DMSO-*d*_6_) δ 12.48 (s, 1H), 8.51 (t, *J* = 5.6 Hz, 1H), 7.75–7.69 (m, 2H), 7.28–7.21 (m, 2H), 4.23 (q, *J* = 7.1 Hz, 2H), 3.55 (td, *J* = 6.9, 5.4 Hz, 2H), 2.75 (t, *J* = 6.9 Hz, 2H), 2.53 (s, 3H), 2.34 (s, 3H), 1.27 (t, *J* = 7.1 Hz, 3H). ^13^C NMR (126 MHz, DMSO) δ 171.01, 166.63, 162.60, 159.95, 156.56, 141.47, 131.99, 129.22, 127.63, 114.29, 60.93, 35.74, 35.62, 21.39, 17.49, 14.66. HPLC/HRMS (ESI): *m*/*z* calculated for C_18_H_22_N_3_O_4_S^+^ [M + H]^+^ 376.1326, found 376.1336. *R*_t_ (4 min): 2.80 min.

##### Ethyl 2-(3-(3-Ethylbenzamido)propanamido)-4-methylthiazole-5-carboxylate (**5**)

Prepared as described for **2** using 3-ethylbenzoic acid (31.5 mg, 0.210 mmol) and **48** (60 mg, 0.233 mmol). An additional washing step of the crude in EtOAc with NaHCO_3_ and brine was required. Yield: 53 mg (0.136 mmol, 58.4%) colorless solid. ^1^H NMR (500 MHz, DMSO) δ 12.51 (s, 1H), 8.56 (t, *J* = 5.6 Hz, 1H), 7.67–7.65 (m, 1H), 7.62 (ddd, *J* = 5.4, 3.9, 1.8 Hz, 1H), 7.37–7.34 (m, 2H), 4.23 (q, *J* = 7.1 Hz, 2H), 3.56 (q, *J* = 6.7 Hz, 2H), 2.75 (t, *J* = 6.9 Hz, 2H), 2.64 (q, *J* = 7.6 Hz, 2H), 2.53 (s, 3H), 1.27 (t, *J* = 7.1 Hz, 3H), 1.19 (t, *J* = 7.6 Hz, 3H). ^13^C NMR (126 MHz, DMSO) δ 170.54, 166.45, 162.13, 159.50, 156.10, 143.79, 134.41, 130.59, 128.20, 126.55, 124.56, 113.82, 60.46, 35.31, 35.16, 28.08, 17.02, 15.50, 14.20. HPLC/HRMS (ESI): *m*/*z* calculated for C_19_H_24_N_3_O_4_S^+^ [M + H]^+^ 390.1482, found 390.1490. *R*_t_ (4 min): 2.93 min.

##### Ethyl 2-(3-(3-Chlorobenzamido)propanamido)-4-methylthiazole-5-carboxylate (**6**)

Prepared as described for **2** using 3-chlorobenzoic acid (32.9 mg, 0.210 mmol) and **48** (60 mg, 0.233 mmol). An additional washing step of the crude in EtOAc with NaHCO_3_ and brine was required. Yield: 55 mg (0.139 mmol, 59.6%) colorless solid. ^1^H NMR (500 MHz, DMSO-*d*_6_) δ 12.52 (s, 1H), 8.73 (t, *J* = 5.5 Hz, 1H), 7.86 (t, *J* = 1.9 Hz, 1H), 7.78 (dt, *J* = 7.8, 1.3 Hz, 1H), 7.59 (ddd, *J* = 8.0, 2.2, 1.1 Hz, 1H), 7.50 (t, *J* = 7.9 Hz, 1H), 4.23 (q, *J* = 7.1 Hz, 2H), 3.57 (q, *J* = 6.6 Hz, 2H), 2.75 (t, *J* = 6.8 Hz, 2H), 2.53 (s, 3H), 1.27 (t, *J* = 7.1 Hz, 3H). ^13^C NMR (126 MHz, DMSO) δ 170.45, 164.87, 162.13, 159.52, 156.10, 136.30, 133.13, 131.02, 130.31, 126.99, 125.96, 113.83, 60.47, 35.43, 34.99, 17.02, 14.20. HPLC/HRMS (ESI): *m*/*z* calculated for C_17_H_19_ClN_3_O_4_S^+^ [M + H]^+^ 396.0779, found 396.0782. *R*_t_ (4 min): 2.87 min.

##### *N*-Ethyl-4-methyl-2-(3-(3-methylbenzamido)propanamido)thiazole-5-carboxamide (**7**)

Ethyl 4-methyl-2-(3-(3-methylbenzamido)propanamido)thiazole-5-carboxylate **2** (93 mg, 0.248 mmol) was dissolved in MeOH/water while stirring at room temperature. NaOH (198 mg, 4.95 mmol) was added, and the mixture was stirred at 55 °C for 1 h. It was then allowed to stir at rt overnight. The mixture was acidified with 2 N HCl and concentrated in vacuo. The residue was partitioned between water and EtOAc. The organic layer was washed with brine and dried over sodium sulfate. The crude product was purified by RP chromatography (0–60% methanol/water, +0.1% formic acid) to give 4-methyl-2-(3-(3-methylbenzamido)propanamido)thiazole-5-carboxylic acid **51** (23 mg, 0.066 mmol, 26.7% yield) as a colorless solid. The product was used as such in the next step. HPLC/MS (ESI): *m*/*z* 348.1028 [M + H]^+^. *R*_t_ (2 min): 1.13 min.

To a stirred solution of **51** (15 mg, 0.043 mmol) and HOBt (13.22 mg, 0.086 mmol) in dry DMF at room temperature were added EDC (16.6 mg, 0.086 mmol), DIPEA (0.023 mL, 0.130 mmol), and ethylamine·HCl (7.04 mg, 0.086 mmol) sequentially. The mixture was stirred at rt for 2 h. The mixture was concentrated in vacuo. The crude product was dissolved in EtOAc and washed with water (×1), aq. sat. bicarb. (×1), and brine (×1). The organic layer was dried over sodium sulfate and concentrated in vacuo. The crude product was purified by NP chromatography (0–2% MeOH/EtOAc), followed by SCX-II ion exchange chromatography, to give **7** (7 mg, 0.019 mmol, 43.3% yield) as a colorless solid. ^1^H NMR (500 MHz, DMSO-*d*_6_) δ 12.29 (s, 1H), 8.54 (t, *J* = 5.6 Hz, 1H), 7.96 (t, *J* = 5.6 Hz, 1H), 7.64 (t, *J* = 1.1 Hz, 1H), 7.60 (ddd, *J* = 5.8, 4.3, 1.8 Hz, 1H), 7.35–7.30 (m, 2H), 3.55 (q, *J* = 6.7 Hz, 2H), 3.20 (qd, *J* = 7.4, 5.7 Hz, 2H), 2.73 (t, *J* = 6.9 Hz, 2H), 2.46 (s, 3H), 2.34 (s, 3H), 1.08 (t, *J* = 7.2 Hz, 3H). ^13^C NMR (126 MHz, DMSO) δ 170.44, 166.85, 161.99, 156.96, 150.43, 137.94, 134.81, 132.16, 128.60, 128.17, 124.75, 119.26, 35.86, 35.56, 34.48, 21.40, 17.35, 15.29. HPLC/HRMS (ESI): *m*/*z* calculated for C_18_H_23_N_4_O_3_S^+^ [M + H]^+^ 375.1485, found 375.1477. *R*_t_ (4 min): 2.29 min.

##### Ethyl 2-(3-Benzamidopropanamido)-4-methylthiazole-5-carboxylate (**9**)

Prepared as described for **2** using benzoic acid (19.0 mg, 0.156 mmol, 1 equiv) and **48** (40 mg, 0.156 mmol). An additional washing step of the crude in EtOAc with NaHCO_3_ and brine was required. Yield: 36 mg (0.100 mmol, 64.1%), colorless solid. ^1^H NMR (500 MHz, DMSO-*d*_6_) δ 12.52 (s, 1H), 8.60 (t, *J* = 5.6 Hz, 1H), 7.85–7.79 (m, 2H), 7.54–7.49 (m, 1H), 7.47–7.42 (m, 2H), 4.23 (q, *J* = 7.1 Hz, 2H), 3.57 (td, *J* = 6.8, 5.4 Hz, 2H), 2.76 (t, *J* = 6.8 Hz, 2H), 2.53 (s, 3H), 1.27 (t, *J* = 7.1 Hz, 3H). ^13^C NMR (126 MHz, DMSO) δ 170.52, 166.30, 162.13, 159.48, 156.10, 134.32, 131.17, 128.25, 127.15, 113.85, 60.48, 35.33, 35.12, 17.03, 14.21. HPLC/HRMS (ESI): *m*/*z* calculated for C_17_H_20_N_3_O_4_S^+^ [M + H]^+^ 362.1169, found 362.1171. *R*_t_ (4 min): 2.63 min.

##### Ethyl 4-Methyl-2-(3-(3-methylcyclohexane-1-carboxamido)propanamido)thiazole-5-carboxylate (**10**)

Prepared as described for **2** using 3-methylcyclohexanecarboxylic acid (22.1 mg, 0.156 mmol) and **48** (40.0 mg, 0.156 mmol). The crude product was purified by NP chromatography (0–2% MeOH/DCM). Yield: 47 mg (0.123 mmol, 79.3%), yellowish solid. (Note: mixture of inseparable diastereomers) ^1^H NMR (500 MHz, DMSO- *d*_6_) δ 12.44 (s, 1H), 7.88–7.71 (m, 1H), 4.23 (q, *J* = 7.1 Hz, 2H), 2.63–2.56 (m, 2H), 2.53 (s, 3H), 2.34 (dt, *J* = 7.6, 3.8 Hz, 0.6H), 2.11–2.03 (m, 0.7H), 1.83–1.74 (m, 0.3H), 1.71–1.36 (m, 5.1H), 1.34–1.29 (m, 0.6H), 1.27 (t, *J* = 7.1 Hz, 3.3H), 1.24–1.04 (m, 2.6H), 0.93 (q, *J* = 12.2 Hz, 0.8H), 0.86–0.81 (m, 3.2H), 0.81–0.70 (m, 0.8H). HPLC/HRMS (ESI): *m*/*z* calculated for C_18_H_28_N_3_O_4_S^+^ [M + H]^+^ 382.1795, found 382.1794. *R*_t_ (4 min): 2.98 min.

##### Ethyl 2-(3-(3-Methoxybenzamido)propanamido)-4-methylthiazole-5-carboxylate (**11**)

Prepared as described for **2** using 3-methoxybenzoic acid (23.7 mg, 0.156 mmol) and **48** (40 mg, 0.156 mmol). Yield: 35 mg (0.0894 mmol, 58.0%), colorless solid. ^1^H NMR (500 MHz, DMSO-*d*_6_) δ 12.52 (s, 1H), 8.59 (t, *J* = 5.5 Hz, 1H), 7.44–7.27 (m, 3H), 7.08 (ddd, *J* = 7.9, 2.6, 1.2 Hz, 1H), 4.23 (q, *J* = 7.1 Hz, 2H), 3.79 (s, 3H), 3.56 (q, *J* = 6.8 Hz, 2H), 2.75 (t, *J* = 6.9 Hz, 2H), 2.53 (s, 3H), 1.27 (t, *J* = 7.1 Hz, 3H). ^13^C NMR (126 MHz, DMSO) δ 170.53, 166.04, 162.13, 159.51, 159.11, 156.10, 135.77, 129.39, 119.38, 116.96, 113.82, 112.39, 60.47, 55.23, 35.36, 35.11, 17.02, 14.20. HPLC/HRMS (ESI): *m*/*z* calculated for C_18_H_22_N_3_O_5_S^+^ [M + H]^+^ 392.1275, found 392.1276. *R*_t_ (4 min): 2.72 min.

##### Ethyl 2-(3-([1,1′-Biphenyl]-3-carboxamido)propanamido)-4-methylthiazole-5-carboxylate (**12**)

Prepared as described for **2** using [1,1′-biphenyl]-3-carboxylic acid (30.8 mg, 0.156 mmol) and **48** (40 mg, 0.156 mmol). An additional washing step of the crude in EtOAc with NaHCO_3_ and brine was required. Yield: 28 mg (0.064 mmol, 41.2%), colorless solid. ^1^H NMR (500 MHz, DMSO-*d*_6_) δ 12.53 (s, 1H), 8.75 (s, 1H), 8.09 (t, *J* = 1.9 Hz, 1H), 7.81 (dd, *J* = 7.7, 1.8 Hz, 2H), 7.74–7.69 (m, 2H), 7.55 (t, *J* = 7.7 Hz, 1H), 7.49 (dd, *J* = 8.4, 7.0 Hz, 2H), 7.40 (s, 1H), 4.23 (d, *J* = 7.1 Hz, 2H), 3.60 (d, *J* = 6.2 Hz, 2H), 2.79 (d, *J* = 6.8 Hz, 2H), 2.53 (s, 3H), 1.27 (t, *J* = 7.1 Hz, 3H). ^13^C NMR (126 MHz, DMSO) δ 170.55, 166.23, 162.13, 159.51, 156.10, 140.14, 139.53, 135.02, 129.35, 128.99, 128.96, 127.77, 126.82, 126.38, 125.36, 113.84, 60.47, 35.41, 35.18, 17.02, 14.20. HPLC/HRMS (ESI): *m*/*z* calculated for C_23_H_24_N_3_O_4_S^+^ [M + H]^+^ 438.1482, found 438.1473. *R*_t_ (4 min): 3.09 min.

##### Ethyl 4-Methyl-2-(3-(3-(5-methyl-1,2,4-oxadiazol-3-yl)benzamido)propanamido)thiazole-5-carboxylate (**13**)

Prepared as described for **2** using 3-(5-methyl-1,2,4-oxadiazol-3-yl)benzoic acid (31.7 mg, 0.156 mmol) and **48** (40 mg, 0.155 mmol). The crude product was purified by normal phase chromatography (3% MeOH in DCM). Yield: 47 mg (0.106 mmol, 68.0%), off-white solid. ^1^H NMR (500 MHz, DMSO-*d*_6_) δ 12.54 (s, 1H), 8.86 (t, *J* = 5.5 Hz, 1H), 8.46 (t, *J* = 1.5 Hz, 1H), 8.16–8.10 (m, 1H), 8.07–7.98 (m, 1H), 7.65 (t, *J* = 7.9 Hz, 1H), 4.23 (q, *J* = 7.1 Hz, 2H), 3.60 (d, *J* = 5.7 Hz, 2H), 2.78 (t, *J* = 6.8 Hz, 2H), 2.68 (s, 3H), 2.53 (s, 3H), 1.27 (t, *J* = 7.1 Hz, 3H). ^13^C NMR (126 MHz, DMSO) δ 177.71, 170.54, 167.26, 165.44, 162.15, 159.55, 156.12, 135.23, 130.09, 129.46, 129.40, 126.43, 125.77, 113.82, 60.48, 35.47, 35.07, 17.04, 14.21, 12.06. HPLC/HRMS (ESI): *m*/*z* calculated for C_20_H_22_N_5_O_5_S^+^ [M + H]^+^ 444.1336, found 444.1338. *R*_t_ (4 min): 2.77 min.

##### Ethyl 2-(3-(3-(5-Ethyl-1,2,4-oxadiazol-3-yl)benzamido)propanamido)-4-methylthiazole-5-carboxylate (**14**)

Prepared as described for **2** using 3-(5-ethyl-1,2,4-oxadiazol-3-yl)benzoic acid (33.9 mg, 0.156 mmol) and **48** (40 mg, 0.156 mmol). The mixture was diluted with EtOAc and washed with water (×1), aq. sat. bicarb. (×1), and brine (×1). The organic layer was dried over sodium sulfate and concentrated in vacuo. The crude was purified by NP chromatography (2–8% EtOH/DCM), followed by RP chromatography (20–100% methanol/water +0.1% formic acid). Yield: 15 mg (0.0328 mmol, 21.0%), fine white powder. ^1^H NMR (500 MHz, DMSO-*d*_6_) δ 12.5 (s, 1H), 8.9 (t, *J* = 5.5 Hz, 1H), 8.5 (t, *J* = 1.7 Hz, 1H), 8.1 (dt, *J* = 1.3, 7.7 Hz, 1H), 8.0 (dt, *J* = 1.3, 7.9 Hz, 1H), 7.7 (t, *J* = 7.8 Hz, 1H), 4.2 (q, *J* = 7.1 Hz, 2H), 3.6 (q, *J* = 6.5 Hz, 2H), 3.0 (q, *J* = 7.6 Hz, 2H), 2.8 (t, *J* = 6.8 Hz, 2H), 2.5 (s, 3H), 1.4 (t, *J* = 7.6 Hz, 3H), 1.3 (t, *J* = 7.1 Hz, 3H). ^13^C NMR (126 MHz, DMSO) δ 181.94, 171.06, 167.60, 165.93, 162.61, 160.13, 156.58, 135.72, 130.51, 129.94, 129.84, 126.93, 126.27, 99.99, 60.91, 35.94, 35.56, 20.08, 17.49, 14.67, 10.92. HPLC/HRMS (ESI): *m*/*z* calculated for C_21_H_24_N_5_O_5_S^+^ [M + H]^+^ 458.1493, found 458.1490. *R*_t_ (4 min): 3.00 min.

##### Ethyl 4-Methyl-2-(3-(3-(5-methyl-1*H*-1,2,4-triazol-3-yl)benzamido)propanamido)thiazole-5-carboxylate (**15**)

Prepared as described for **2** using 3-(5-methyl-1*H*-1,2,4-triazol-3-yl)benzoic acid hydrochloride (37.26 mg, 0.156 mmol) and **48** (40 mg, 0.155 mmol) and stirred for 48 h. The mixture was diluted with EtOAc (25 mL) and washed with water (25 mL), then aq. sat. bicarb. (10 mL). The organic layer was dried over sodium sulfate and concentrated in vacuo. This crude was purified by RP chromatography (30–90% methanol/water + 0.1% formic acid). Yield: 5 mg (0.0113 mmol, 7.3%), white powder. ^1^H NMR (500 MHz, DMSO-*d*_6_) δ 13.8 (s, 1H), 12.5 (s, 1H), 8.8 (t, *J* = 5.5 Hz, 1H), 8.4 (t, *J* = 1.8 Hz, 1H), 8.1 (dt, *J* = 1.4, 7.7 Hz, 1H), 7.8 (dt, *J* = 1.4, 7.8 Hz, 1H), 7.5 (t, *J* = 7.8 Hz, 1H), 4.2 (q, *J* = 7.1 Hz, 2H), 3.6 (q, *J* = 6.7 Hz, 2H), 2.8 (t, *J* = 6.9 Hz, 2H), 2.5 (s, 3H), 2.4 (s, 3H), 1.3 (t, *J* = 7.1 Hz, 3H). ^13^C NMR (126 MHz, DMSO) δ 171.1, 166.5, 162.6, 160.1, 156.6, 135.3, 129.2, 128.7, 128.0, 125.0, 114.2, 60.9, 35.9, 35.6, 17.5, 14.7, 12.4 (3 × C not observed). HPLC/HRMS (ESI): *m*/*z* calculated for C_20_H_23_N_6_O_4_S^+^ [M + H]^+^ 443.1496, found 443.1496. *R*_t_ (4 min): 2.54 min.

##### Ethyl 4-Methyl-2-(3-(3-(5-methyl-1,3,4-oxadiazol-2-yl)benzamido)propanamido)thiazole-5-carboxylate (**16**)

Prepared as described for **2** using 3-(5-methyl-1,3,4-oxadiazol-2-yl)benzoic acid (31.7 mg, 0.156 mmol) and **48** (40 mg, 0.155 mmol). Stirred at rt for 3 days. The mixture was diluted with EtOAc (25 mL) and washed with water (25 mL) then aq. sat. bicarb. (10 mL). The organic layer was dried over sodium sulfate and concentrated in vacuo. The crude product was purified by RP chromatography (30–90% methanol/water + 0.1% formic acid). Yield: 10 mg (0.0225 mmol, 14.5%), white powder. ^1^H NMR (500 MHz, DMSO-*d*_6_) δ 12.5 (s, 1H), 8.9 (t, *J* = 5.5 Hz, 1H), 8.4 (t, *J* = 1.8 Hz, 1H), 8.1–8.1 (m, 1H), 8.0 (dt, *J* = 1.3, 7.9 Hz, 1H), 7.7 (t, *J* = 7.8 Hz, 1H), 4.2 (q, *J* = 7.1 Hz, 2H), 3.6 (td, *J* = 6.6 Hz, 2H), 2.8 (t, *J* = 6.8 Hz, 2H), 2.6 (s, 3H), 2.5 (s, 3H), 1.3 (t, *J* = 7.1 Hz, 3H). ^13^C NMR (126 MHz, DMSO) δ 170.9, 165.7, 164.7, 164.0, 162.6, 159.9, 156.6, 135.8, 130.8, 130.1, 129.3, 125.5, 124.1, 114.3, 61.0, 35.9, 35.5, 17.5, 14.7, 11.1. HPLC/HRMS (ESI): *m*/*z* calculated for C_20_H_22_N_5_O_5_S^+^ [M + H]^+^ 444.1336, found 444.1341. *R*_t_ (4 min): 2.73 min.

##### Ethyl 4-Methyl-2-(3-(3-(3-methyl-1,2,4-oxadiazol-5-yl)benzamido)propanamido)thiazole-5-carboxylate (**17**)

Prepared as described for **2** using 3-(3-methyl-1,2,4-oxadiazol-5-yl)benzoic acid (31.7 mg, 0.156 mmol) and **48** (40.0 mg, 0.155 mmol). The mixture was diluted with EtOAc and washed with water (×1), aq. sat. bicarb. (×1), and brine (×1). The organic layer was dried over sodium sulfate and concentrated in vacuo. The crude product was purified by NP chromatography (0–3% methanol/DCM). Yield: 8 mg (0.0180 mmol, 11.6%), off-white solid. ^1^H NMR (500 MHz, DMSO-*d*_6_) δ 12.54 (s, 1H), 8.93 (t, *J* = 5.5 Hz, 1H), 8.54 (dt, *J* = 1.8, 1.0 Hz, 1H), 8.22 (ddd, *J* = 7.8, 1.8, 1.1 Hz, 1H), 8.13 (ddd, *J* = 7.9, 1.9, 1.2 Hz, 1H), 7.76–7.69 (m, 1H), 4.23 (q, *J* = 7.1 Hz, 2H), 3.61 (q, *J* = 6.5 Hz, 2H), 2.79 (t, *J* = 6.8 Hz, 2H), 2.54 (s, 3H), 2.44 (s, 3H), 1.27 (t, *J* = 7.1 Hz, 3H). ^13^C NMR (126 MHz, DMSO) δ ^13^C NMR (126 MHz, DMSO) δ 174.25, 170.48, 167.79, 165.00, 162.14, 159.50, 156.12, 135.36, 131.73, 130.23, 129.78, 126.38, 123.57, 113.85, 60.48, 35.49, 35.02, 17.03, 14.21, 11.28. HPLC/HRMS (ESI): *m*/*z* calculated for C_20_H_22_N_5_O_5_S^+^ [M + H]^+^ 444.1336, found 444.1336. *R*_t_ (4 min): 2.90 min.

##### Ethyl 4-Methyl-2-(3-(3-(2-methyl-2*H*-tetrazol-5-yl)benzamido)propanamido)thiazole-5-carboxylate (**18**)

Prepared as described for **2** using 3-(2-methyltetrazol-5-yl)benzoic acid (31.7 mg, 0.156 mmol) and **48** (40 mg, 0.155 mmol). The mixture was diluted with EtOAc and washed with water (×1), aq. sat. bicarb. (×1), and brine (×1). The organic layer was dried over magnesium sulfate and concentrated in vacuo. The crude product was purified by RP chromatography (30–100% methanol/water + 0.1% formic acid). Yield: 21 mg (0.0474 mmol, 30.0%), white powder. ^1^H NMR (500 MHz, DMSO-*d*_6_) δ 12.54 (s, 1H), 8.86 (t, *J* = 5.5 Hz, 1H), 8.52 (t, *J* = 1.7 Hz, 1H), 8.19 (dt, *J* = 7.7, 1.3 Hz, 1H), 7.98 (dt, *J* = 7.9, 1.3 Hz, 1H), 7.66 (t, *J* = 7.8 Hz, 1H), 4.45 (s, 3H), 4.23 (q, *J* = 7.1 Hz, 2H), 3.60 (q, *J* = 6.5 Hz, 2H), 2.79 (t, *J* = 6.8 Hz, 2H), 2.53 (s, 3H), 1.27 (t, *J* = 7.1 Hz, 3H). ^13^C NMR (126 MHz, DMSO) δ 170.55, 165.56, 163.67, 162.14, 159.57, 156.11, 135.26, 129.40, 129.14, 128.82, 127.04, 125.10, 113.80, 60.47, 35.46, 35.09, 17.03, 14.21 (1 × C underneath solvent peak). HPLC/HRMS (ESI): *m*/*z* calculated for C_19_H_22_N_7_O_4_S^+^ [M + H]^+^ 444.1448, found 444.1442. *R*_t_ (4 min): 3.12 min.

##### Methyl 3-(3-(5-Methyl-1,2,4-oxadiazol-3-yl)benzamido)propanoate (**57**)

To methyl 3-aminopropanoate hydrochloride (547 mg, 3.9 mmol) and 3-(5-methyl-1,2,4-oxadiazol-3-yl)benzoic acid (800 mg, 3.9 mmol) in DMF (20 mL) was added DIPEA (2.7 mL, 15.7 mmol), followed by HATU (1.38 g, 5.9 mmol). The obtained yellow solution was stirred at rt for 20 h. The reaction mixture was diluted with ethyl acetate (200 mL) and washed with water (250 mL). The aqueous phase was extracted with fresh ethyl acetate (100 mL). The combined organic layers were washed with aqueous saturated sodium bicarbonate solution (150 mL) and brine (200 mL), then dried over MgSO_4_. Filtering and concentrating in vacuo afforded methyl 3-(3-(5-methyl-1,2,4-oxadiazol-3-yl)benzamido)propanoate (1.12 g, 99%, 3.9 mmol) as an off-white colored amorphous powder. ^1^H NMR (500 MHz, CDCl_3_) δ 8.40 (td, *J* = 1.8, 0.5 Hz, 1H), 8.19 (dt, *J* = 7.8, 1.4 Hz, 1H), 7.95 (ddd, *J* = 7.8, 1.9, 1.2 Hz, 1H), 7.57 (td, *J* = 7.8, 0.6 Hz, 1H), 6.90 (s, 1H), 3.76 (q, *J* = 6.0 Hz, 2H), 3.73 (s, 3H), 2.70–2.66 (m, 5H); HPLC/MS (ESI): *m*/*z* 312.0963 [M + Na]^+^. *R*_t_ (2 min): 1.02 min.

##### 3-(3-(5-Methyl-1,2,4-oxadiazol-3-yl)benzamido)propanoic Acid (**60**)

To methyl 3-(3-(5-methyl-1,2,4-oxadiazol-3-yl)benzamido)propanoate **58** (200.0 mg, 0.691 mmol) in THF (3.46 mL) was added water (3.46 mL), followed by lithium hydroxide hydrate (116.0 mg, 2.77 mmol). After stirring for 1.5 h, to the mixture was added water (20 mL), and the THF was removed in vacuo. The solution was acidified with 1 N citric acid solution and extracted with EtOAc (2 × 30 mL). The organics were combined, washed with brine and dried over MgSO_4_. This gave 3-(3-(5-methyl-1,2,4-oxadiazol-3-yl)benzamido)propanoic acid (174 mg, 91%, 0.632 mmol) as a colorless solid. No further purification was performed. ^1^H NMR (500 MHz, DMSO-*d*_6_) δ 12.22 (brs, 1H), 8.78 (brt, *J* = 5.4 Hz, 1H), 8.46 (t, *J* = 1.7 Hz, 1H), 8–14–8.12 (m, 1H), 8.07–7.95 (m, 1H), 7.66 (t, *J* = 7.8 Hz, 1H), 3.50–3.46 (m, 2H), 2.69 (s, 3H), 2.54 (t, *J* = 7.1 Hz, 2H). HPLC/MS (ESI): *m*/*z* 298.0808 [M + Na]^+^. *R*_t_ (2 min): 0.93 min.

##### Ethyl 2-(3-(3-(5-Methyl-1,2,4-oxadiazol-3-yl)benzamido)propanamido)thiazole-5-carboxylate (**19**)

To a solution of ethyl 2-aminothiazole-5-carboxylate (37.1 mg, 0.216 mmol) and 3-(3-(5-methyl-1,2,4-oxadiazol-3-yl)benzamido)propanoic acid **60** (50.0 mg, 0.182 mmol) in DMF (0.91 mL, 0.200 M) were added HOBt (49.1 mg, 0.363 mmol) and EDC (56.4 mg, 0.3633 mmol). The mixture was stirred for 18 h at 60 °C. The reaction mixture was partitioned between water (50 mL) and EtOAc (40 mL). The organic layer was washed with water (40 mL), 1 N HCl (20 mL), aq. sat. bicarb. (20 mL), and brine (20 mL). The organic layer was dried over sodium sulfate and concentrated. The crude was purified by RP column chromatography eluted with 30–100% MeOH/H_2_O (+0.1% formic acid modifier in both) to afford ethyl 2-[3-[[3-(5-methyl-1,2,4-oxadiazol-3-yl)benzoyl]amino]propanoylamino]thiazole-5-carboxylate (10 mg, 13%, 0.0233 mmol) as a white fluffy powder. ^1^H NMR (500 MHz, DMSO-*d*_6_) δ 12.7 (s, 1H), 8.9 (t, *J* = 5.5 Hz, 1H), 8.5 (t, *J* = 1.7 Hz, 1H), 8.1–8.2 (m, 2H), 8.0–8.1 (m, 1H), 7.7 (t, *J* = 7.8 Hz, 1H), 4.3 (q, *J* = 7.1 Hz, 2H), 3.6 (q, *J* = 6.5 Hz, 2H), 2.8 (t, *J* = 6.8 Hz, 2H), 2.7 (s, 3H), 1.3 (t, *J* = 7.1 Hz, 3H). ^13^C NMR (126 MHz, DMSO) δ 178.17, 171.01, 167.71, 165.92, 162.64, 161.91, 145.54, 135.68, 130.56, 129.92, 129.86, 126.89, 126.23, 121.49, 61.31, 35.91, 35.47, 14.66, 12.52. HPLC/HRMS (ESI): *m*/*z* calculated for C_19_H_20_N_5_O_5_S^+^ [M + H]^+^ 430.1180, found 430.1185. *R*_t_ (4 min): 2.75 min.

##### 3-(5-Methyl-1,2,4-oxadiazol-3-yl)-*N*-(3-((4-methylthiazol-2-yl)amino)-3-oxopropyl)benzamide (**20**)

Prepared as described for **19** using 4-methylthiazol-2-amine (12.30 mg, 0.1078 mmol), EDC·HCl (2 equiv) instead of EDC, and **60** (25.0 mg, 0.0908 mmol). The reaction mixture was partitioned between water (50 mL) and EtOAc (40 mL). The organic layer was washed with water (40 mL), aq. sat. bicarb. (20 mL), and brine (20 mL). The organic layer was dried over MgSO_4_ and concentrated in vacuo before purification. Yield: 13 mg (0.0350 mmol, 39.0%), white powder. ^1^H NMR (500 MHz, DMSO-*d*_6_) δ 12.07 (s, 1H), 8.87 (t, *J* = 5.5 Hz, 1H), 8.47 (d, *J* = 1.8 Hz, 1H), 8.19–8.09 (m, 1H), 8.03 (dt, *J* = 7.8, 1.5 Hz, 1H), 7.65 (t, *J* = 7.8 Hz, 1H), 6.72 (s, 1H), 3.59 (dt, *J* = 6.6 Hz, 2H), 2.74 (t, *J* = 6.9 Hz, 2H), 2.68 (s, 3H), 2.24 (d, *J* = 1.1 Hz, 3H). ^13^C NMR (126 MHz, DMSO) δ 178.14, 169.85, 167.72, 165.86, 157.63, 146.97, 135.71, 130.54, 129.89, 129.82, 126.88, 126.23, 107.95, 36.05, 35.39, 17.33, 12.51. HPLC/HRMS (ESI): *m*/*z* calculated for C_17_H_18_N_5_O_3_S^+^ [M + H]^+^ 372.1125, found 372.1135. *R*_t_ (4 min): 2.92 min.

##### Ethyl 4-Methyl-2-(2-(3-(5-methyl-1,2,4-oxadiazol-3-yl)benzamido)acetamido)thiazole-5-carboxylate (**21**)

To methyl 2-aminoacetate hydrochloride (87.0 mg, 0.693 mmol) and 3-(5-methyl-1,2,4-oxadiazol-3-yl)benzoic acid (141.5 mg, 0.693 mmol) in DMF (3.47 mL) was added DIPEA (0.48 mL, 2.77 mmol), followed by HATU (395.3 mg, 1.040 mmol). The reaction mixture was stirred at rt for 4 h. The mixture was diluted with EtOAc (20 mL) and washed twice with water (15 mL each time). The water was extracted with EtOAc (15 mL). The organic layers were combined and washed with aq. sat. bicarb. (20 mL) and brine (20 mL), then dried over MgSO_4_. After filtration and concentration in vacuo, the methyl (3-(5-methyl-1,2,4-oxadiazol-3-yl)benzoyl)glycinate (185 mg, 97%, 0.672 mmol) was isolated as an orange oil. The product **56** was directly used in the next step without further purification. HPLC/MS (ESI): *m*/*z* 276.0957 [M + H]^+^. *R*_t_ (2 min): 1.16 min.

To **56** (170.0 mg, 0.618 mmol) in THF (3.09 mL, 0.100 M) was added water (3.09 mL, 0.100 M), followed by LiOH hydrate (103.7 mg, 2.47 mmol). The solution was stirred at rt for 1.5 h. To the mixture was added water (15 mL), and THF was removed in vacuo. The residue was washed with EtOAc (15 mL). The aqueous phase was acidified using a HCl 1N solution and then extracted with EtOAc (20 mL). The organic layers were combined, washed with brine, dried over MgSO_4_ ,and concentrated under reduced pressure to afford **59** (140 mg, 87%, 0.536 mmol) as a colorless solid, which was used directly in the next step without further purification. HPLC/MS (ESI): *m*/*z* 262.0810 [M + H]^+^. *R*_t_ (2 min): 1.04 min.

Preparation of **21** was performed as described for **19** using ethyl 2-amino-4-methyl-thiazole-5-carboxylate (27.5 mg, 0.147 mmol), EDC·HCl (2 equiv) instead of EDC, and **59** (35.0 mg, 0.134 mmol). The reaction mixture was partitioned between water (25 mL) and EtOAc (25 mL). The organic phase was washed with water (20 mL), aq. sat. bicarb. (20 mL), and brine (20 mL), dried over MgSO_4_, and concentrated before purification. Yield: 25.2 mg (0.0587 mmol, 44.0%), colorless solid. ^1^H NMR (500 MHz, DMSO-*d*_6_) δ 12.69 (broad s, 1H), 9.20 (t, *J* = 5.7 Hz, 1H), 8.54 (t, *J* = 1.8 Hz, 1H), 8.17 (dt, *J* = 1.4, 7.8 Hz, 1H), 8.09 (dt, *J* = 1.4, 7.9 Hz, 1H), 7.70 (t, *J* = 7.8 Hz, 1H), 4.26–4.19 (m, 4H), 2.69 (s, 3H), 2.55 (s, 3H), 1.27 (t, *J* = 7.1 Hz, 3H). ^13^C NMR (126 MHz, DMSO) δ 178.20, 169.62, 167.69, 166.36, 162.56, 160.25, 156.67, 135.07, 130.71, 130.21, 129.96, 126.97, 126.37, 114.38, 60.94, 43.33, 17.51, 14.65, 12.53. HPLC/HRMS (ESI): *m*/*z* calculated for C_19_H_20_N_5_O_5_S^+^ [M + H]^+^ 430.1180, found 430.1173. *R*_t_ (4 min): 2.78 min.

##### Ethyl 2-(4-((*tert*-Butoxycarbonyl)amino)butanamido)-4-methylthiazole-5-carboxylate (**46**)

To a solution of ethyl 2-amino-4-methyl-thiazole-5-carboxylate (108.7 mg, 0.584 mmol) and 4-(*tert*-butoxycarbonylamino)butanoic acid (100.0 mg, 0.492 mmol) in DMF (2.46 mL, 0.200 M) were added HOBt (133.0 mg, 0.984 mmol) and EDC (152.8 mg, 0.984 mmol). The mixture was stirred for 18.5 h at 60 °C. The reaction mixture was partitioned between water (100 mL) and EtOAc (100 mL). The organic layer was washed with water (100 mL), 1 N HCl (50 mL), aq. sat. bicarb. (75 mL), and brine (80 mL). The organic layer was dried over MgSO_4_ and concentrated under reduced pressure to give ethyl 2-[4-(*tert*-butoxycarbonylamino)butanoylamino]-4-methyl-thiazole-5-carboxylate (145.7 mg, 80%, 0.392 mmol) as a sticky yellow foam. ^1^H NMR (500 MHz, chloroform-*d*) δ 4.80 (brs, 1H), 4.33 (q, *J* = 7.1 Hz, 2H), 3.25 (d, *J* = 6.6 Hz, 2H), 2.66 (s, 3H), 2.62–2.50 (m, 2H), 1.94 (t, *J* = 6.7 Hz, 2H), 1.46 (s, 9H), 1.37 (t, *J* = 7.1 Hz, 3H). HPLC/MS (ESI): *m*/*z* 394.1413 [M + Na]^+^. *R*_t_ (2 min): 1.34 min. HPLC/HRMS (ESI): *m*/*z* calculated for C_16_H_25_N_3_O_5_SNa^+^ [M + Na]^+^ 394.1407, found 394.1403. *R*_t_ (4 min): 2.84 min.

##### Ethyl 2-(4-Aminobutanamido)-4-methylthiazole-5-carboxylate (**49**)

4 N HCl in dioxane (2.95 mL, 11.8 mmol) was added dropwise to a solution of ethyl 2-[4-(*tert*-butoxycarbonylamino)butanoylamino]-4-methyl-thiazole-5-carboxylate **46** (146.0 mg, 0.393 mmol) in EtOH (3.9 mL). The reaction mixture was left stirring at rt. The volatiles were evaporated to dryness. The residue was dissolved in DCM (50 mL) and washed with aq. sat. bicarb. (1 × 75 mL) and brine (1 × 75 mL). The organic layer was dried over MgSO_4_ and concentrated in vacuo to give ethyl 2-(4-aminobutanoylamino)-4-methyl-thiazole-5-carboxylate **49** (36 mg, 34%, 0.133 mmol) as a yellow film. This was taken through to the next step without further purification. HPLC/MS (ESI): *m*/*z* 294.0887 [M + Na]^+^. *R*_t_ (2 min): 0.84 min.

##### Ethyl 4-Methyl-2-(4-(3-(5-methyl-1,2,4-oxadiazol-3-yl)benzamido)butanamido)thiazole-5-carboxylate (**22**)

Prepared as described for **2** using 3-(5-methyl-1,2,4-oxadiazol-3-yl)benzoic acid (26.3 mg, 0.129 mmol), ethyl 2-(4-aminobutanamido)-4-methylthiazole-5-carboxylate **49** (35.0 mg, 0.129 mmol) and DIPEA (68 μL, 0.387 mmol) in DMF (1.29 mL). HATU (215 mg, 0.910 mmol) was added, and the mixture was stirred overnight at rt. Yield: 53 mg (0.136 mmol, 58.4%), colorless solid. The crude product was purified by RP chromatography (30–100% methanol/water + 0.1% formic acid). Yield: 18.7 mg (0.0409 mmol, 32.0%), white solid. ^1^H NMR (500 MHz, DMSO-*d*_6_) δ 12.44 (s, 1H), 8.71 (t, *J* = 5.6 Hz, 1H), 8.45 (t, *J* = 1.7 Hz, 1H), 8.11 (dt, *J* = 7.7, 1.4 Hz, 1H), 8.06–7.99 (m, 1H), 7.64 (t, *J* = 7.8 Hz, 1H), 4.22 (q, *J* = 7.1 Hz, 2H), 3.37–3.33 (m, 2H), 2.68 (s, 3H), 2.54–2.52 (m, *J* = 8.6 Hz, 5H), 1.89 (p, *J* = 7.0 Hz, 2H), 1.27 (t, *J* = 7.1 Hz, 3H). ^13^C NMR (126 MHz, DMSO) δ 178.1, 172.4, 167.7, 165.8, 162.6, 160.1, 156.6, 135.9, 130.5, 129.8, 129.7, 126.8, 126.2, 114.2, 60.9, 39.1, 33.0, 24.8, 17.5, 14.7, 12.5 ppm. HPLC/MS (ESI): *m*/*z* 458.0937 [M + H]^+^. *R*_t_ (2 min): 1.29 min. HPLC/HRMS (ESI): *m*/*z* calculated for C_21_H_24_N_5_O_5_S^+^ [M + H]^+^ 458.1493, found 458.1493. *R*_t_ (4 min): 2.72 min.

##### Ethyl 4-Methyl-2-(*N*-methyl-3-(3-(5-methyl-1,2,4-oxadiazol-3-yl)benzamido)propanamido)thiazole-5-carboxylate (**23**)

To a stirred solution of 3-(*tert*-butoxycarbonylamino)propanoic acid (430 mg, 2.27 mmol) and HOBt (614 mg, 4.55 mmol) in dry DMF (11.36 mL, 0.200 M) under a nitrogen atmosphere at rt were added EDC (705.6 mg, 4.55 mmol) and ethyl 4-methyl-2-(methylamino)thiazole-5-carboxylate (540.0 mg, 2.70 mmol) sequentially. The mixture was stirred for 18 h at 60 °C. The reaction mixture was partitioned between water (50 mL) and EtOAc (40 mL). The organic layer was washed with water (40 mL), aq. sat. bicarb. (20 mL), and brine (20 mL). The organic layer was dried over sodium sulfate and concentrated in vacuo to give **47** (798 mg, 95%, 2.15 mmol) as a yellow solid. HPLC/MS (ESI): *m*/*z* 372.1569 [M + H]^+^. *R*_t_ (2 min): 1.54 min.

Ethyl 2-(3-((*tert*-butoxycarbonyl)amino)-*N*-methylpropanamido)-4-methylthiazole-5-carboxylate **47** (400.00 mg, 1.08 mmol) was dissolved in dioxane (5.38 mL). To the solution was added 4 M HCl in dioxane (5.38 mL, 21.5 mmol) dropwise while stirring at rt. After 2 h, volatiles were removed in vacuo. The crude ethyl 2-(3-((*tert*-butoxycarbonyl)amino)-*N*-methylpropanamido)-4-methylthiazole-5-carboxylate **50** was used immediately in the next step to avoid decomposition. HPLC/MS (ESI): *m*/*z* 272.1145 [M + H]^+^. *R*_t_ (2 min): 0.94 min.

Preparation of **23** was performed as described for **2** using **50** (292.0 mg, 1.08 mmol) and 3-(5-methyl-1,2,4-oxadiazol-3-yl)benzoic acid **55** (219.7 mg, 1.08 mmol). The reaction mixture was diluted with EtOAc and washed with water (×1), aq. sat. bicarb. (×1), and brine (×1). The organic layer was dried over magnesium sulfate and concentrated in vacuo to give a yellow colored crude (469 mg). 90 mg of this material was purified by RP chromatography (30–80% methanol/water + 0.1% formic acid). Yield: 40 mg (0.0874 mmol, 8%), white powder. ^1^H NMR (500 MHz, DMSO-*d*_6_) δ 8.8 (t, *J* = 5.4 Hz, 1H). 8.5 (td, *J* = 0.6, 1.8 Hz, 1H), 8.1–8.2 (m, 1H), 8.0 (ddd, *J* = 1.1, 1.8, 7.8 Hz, 1H), 7.6–7.7 (m, 1H), 4.2 (q, *J* = 7.1 Hz, 2H), 3.6–3.7 (m, 5H), 3.1 (t, *J* = 6.8 Hz, 2H), 2.7 (s, 3H), 2.6 (s, 3H), 1.3 (t, *J* = 7.1 Hz, 3H). ^13^C NMR (126 MHz, DMSO) δ 178.2, 172.6, 167.7, 165.9, 162.8, 160.7, 155.4, 135.7, 130.6, 129.9, 129.9, 126.9, 126.2, 115.7, 61.0, 35.7, 34.6, 34.3, 17.7, 14.6, 12.5. HPLC/HRMS (ESI): *m*/*z* calculated for C_21_H_24_N_5_O_5_S^+^ [M + H]^+^ 458.1493, found 458.1505. *R*_t_ (4 min): 3.23 min.

##### Ethyl 4-Methyl-2-(3-(*N*-methyl-3-(5-methyl-1,2,4-oxadiazol-3-yl)benzamido)propanamido)thiazole-5-carboxylate (**24**)

To methyl 3-(methylamino)propanoate (0.16 mL, 1.22 mmol) and 3-(5-methyl-1,2,4-oxadiazol-3-yl)benzoic acid (250 mg, 1.22 mmol) in DMF (6.12 mL) was added DIPEA (0.64 mL, 3.67 mmol), followed by HATU (698 mg, 1.84 mmol). The reaction mixture was stirred at rt for a 48 h. The mixture was diluted with EtOAc (25 mL) and washed with water (30 mL). The water was extracted with EtOAc (15 mL). The organic layers were combined and washed with aq. sat. bicarb. (50 mL) and brine (50 mL), then dried over MgSO_4_. After filtration and concentration in vacuo, methyl 3-(*N*-methyl-3-(5-methyl-1,2,4-oxadiazol-3-yl)benzamido)propanoate (300 mg, 81%, 0.990 mmol) was isolated as an orange oil. The product methyl 3-(*N*-methyl-3-(5-methyl-1,2,4-oxadiazol-3-yl)benzamido)propanoate **58** was directly used in the next step without further purification. HPLC/MS (ESI): *m*/*z* 304.1297 [M + H]^+^. *R*_t_ (2 min): 1.22 min.

To methyl 3-(*N*-methyl-3-(5-methyl-1,2,4-oxadiazol-3-yl)benzamido)propanoate **58** (300.0 mg, 0.989 mmol) in THF (3.30 mL, 0.150 M) was added water (3.30 mL, 0.150 M), followed by lithium hydroxide hydrate (166 mg, 3.96 mmol). The solution was stirred at rt for 1.5 h. To the solution was added water (15 mL), THF was removed in vacuo, and the residue was washed with EtOAc (15 mL). The aqueous phase was acidified using a 1 N HCl solution and then extracted with EtOAc (20 mL). The organic layers were combined, washed with brine, dried over MgSO_4_, and concentrated under reduced pressure to afford 3-(*N*-methyl-3-(5-methyl-1,2,4-oxadiazol-3-yl)benzamido)propanoic acid **61** (205 mg, 72%, 0.709 mmol) as a colorless solid. 3-(*N*-methyl-3-(5-methyl-1,2,4-oxadiazol-3-yl)benzamido)propanoic acid was used directly in the next without further purification. HPLC/MS (ESI): *m*/*z* 290.1129 [M + H]^+^. *R*_t_ (2 min): 1.12 min.

Preparation of **24** was performed as described for **19** using 4-methylthiazol-2-amine (24.8 mg, 0.133 mmol) and **61** (35.0 mg, 0.121 mmol). Yield: 27.5 mg (0.0601 mmol, 50.0%) colorless solid. Note: rotamers were present. ^1^H NMR (500 MHz, DMSO) δ 12.55 (s, 1H), 8.08–7.75 (m, 2H), 7.67–7.49 (m, 2H), 4.23 (q, *J* = 7.1 Hz, 2H), 3.85–3.58 (m, 2H), 3.03–2.88 (m, 3H), 2.82 (s, 1H), 2.65 (d, *J* = 12.6 Hz, 4H), 2.53 (d, *J* = 8.1 Hz, 2H), 2.47 (s, 1H), 1.28 (t, *J* = 7.1 Hz, 3H). HPLC/HRMS (ESI): *m*/*z* calculated for C_21_H_24_N_5_O_5_S^+^ [M + H]^+^ 458.1493, found 458.1487. *R*_t_ (4 min): 2.78 min.

##### Ethyl 2-Formyl-4-methylthiazole-5-carboxylate (**69**)

A solution of chloro(isopropyl)magnesium chlorolithium (3.60 mL, 4.68 mmol) was added to a solution of ethyl 2-bromo-4-methyl-thiazole-5-carboxylate (0.90 g, 3.60 mmol) in dry THF (5 mL) at −78 °C. The reaction mixture was stirred for 10 min at −78 °C before the addition of morpholine-4-carbaldehyde (0.90 mL, 9.00 mmol). After stirring for 25 min, the reaction mixture was quenched with NH_4_Cl (10 mL). The aqueous layer was extracted with EtOAc (2 × 10 mL), dried over MgSO_4_, and concentrated under reduced vacuum. The crude product was purified by NP chromatography (0–20% EtOAc/cyclohexanes) to give **69** (495 mg, 69%, 2.49 mmol) as a colorless oil. ^1^H NMR (500 MHz, chloroform-*d*) δ 9.94 (s, 1H), 4.38 (q, *J* = 7.2 Hz, 2H), 2.82 (s, 3H), 1.39 (t, *J* = 7.1 Hz, 4H). HPLC/MS (ESI): *m*/*z* 200.0348 [M + H]^+^. *R*_t_ (4 min): 2.46 min.

##### 5-(Ethoxycarbonyl)-4-methylthiazole-2-carboxylic acid (**70**)

2-Methylbut-2-ene (7.82 mL, 73.8 mmol) was added to a solution of ethyl 2-formyl-4-methyl-thiazole-5-carboxylate **69** (490.0 mg, 2.46 mmol) in THF (8.20 mL, 0.150 M) and *t*-BuOH (8.20 mL, 0.150 M) at rt. After 5 min, a solution of sodium dihydrogen phosphate (885 mg, 7.38 mmol) and sodium chlorite (734.0 mg, 8.12 mmol) in H_2_O (4 mL) was added dropwise to the reaction mixture. The reaction mixture was stirred for 1.5 h at rt. The reaction mixture was quenched with a sat. solution of Na_2_S_2_O_3_. The aqueous layer was extracted with EtOAc, then acidified to pH 1–2 with a solution of 2 N HCl and extracted again with EtOAc. The organic layer was dried over MgSO_4_, and concentrated under reduced pressure to give **70** (382 mg, 72%, 1.77 mmol) as a white powder. ^1^H NMR (500 MHz, chloroform-*d*) δ 8.83 (s, 1H), 4.37 (dq, *J* = 12.1, 7.1 Hz, 3H), 2.79 (d, *J* = 3.8 Hz, 5H), 1.39 (td, *J* = 7.1, 5.4 Hz, 5H). HPLC/MS (ESI): *m*/*z* 216.0309 [M + H]^+^. *R*_t_ (2 min): 1.1 min.

##### Ethyl 2-((2-((*tert*-Butoxycarbonyl)amino)ethyl)carbamoyl)-4-methylthiazole-5-carboxylate (**71**)

*tert*-Butyl *N*-(2-aminoethyl)carbamate (0.16 mL, 1.03 mmol) was added to a solution of 5-(ethoxycarbonyl)-4-methylthiazole-2-carboxylic acid **70** (130.00 mg, 0.604 mmol), 3-(ethyliminomethyleneamino)-*N*,*N*-dimethyl-propan-1-amine, HCl (240.4 mg, 1.55 mmol), and 1-hydroxybenzotriazole (208 mg, 1.54 mmol) in dry DMF at rt. The reaction mixture was stirred for 2 days at rt. The mixture was diluted with EtOAc (10 mL); washed with a sat. solution of NaHCO_3_ (2 × 5 mL) and water (2 × 5 mL), dried over MgSO_4_, and concentrated in vacuo. The crude product was purified via NP chromatography (5–50% EtOAc/cyclohexanes) to give **71** (80 mg, 37%, 0.224 mmol) as a white powder. ^1^H NMR (500 MHz, chloroform-*d*) δ 7.67 (s, 1H), 4.88 (s, 1H), 4.35 (q, *J* = 7.1 Hz, 2H), 3.56 (q, *J* = 5.9 Hz, 2H), 3.39 (d, *J* = 6.4 Hz, 2H), 2.72 (s, 3H), 1.43 (s, 9H), 1.38 (t, *J* = 7.1 Hz, 3H). HPLC/MS (ESI): *m*/*z* 380.1197 [M + Na]^+^. *R*_t_ (2 min): 2.90 min.

##### Ethyl 4-Methyl-2-((2-(3-(5-methyl-1,2,4-oxadiazol-3-yl)benzamido)ethyl)carbamoyl)thiazole-5-carboxylate (**25**)

4 N HCl in dioxane (1.08 mL, 4.31 mmol) was added to a solution of **71** (77.0 mg, 0.215 mmol) in dry dioxane (1.08 mL, 0.200 M) at rt. The reaction mixture was stirred overnight at rt. The solvent was removed under reduced pressure to give crude ethyl 2-((2-aminoethyl)carbamoyl)-4-methylthiazole-5-carboxylate **72**. This ethyl 2-(2-aminoethylcarbamoyl)-4-methyl-thiazole-5-carboxylate **72** (55.0 mg, 0.214 mmol), 3-(5-methyl-1,2,4-oxadiazol-3-yl)benzoic acid (52.4 mg, 0.257 mmol), HOBt (57.8 mg, 0.428 mmol), and EDC (66.4 mg, 0.428 mmol) were dissolved in dry DMF (1.07 mL) and stirred overnight at rt. The reaction mixture was diluted with EtOAc, washed with NaHCO_3_ (2 × 8 mL) and water (8 mL), dried over MgSO_4_, and concentrated under reduced pressure. The crude product was purified by RP chromatography (30–100% MeOH/H_2_O + 0.1% formic acid) to give **25** (48 mg, 51%, 0.108 mmol) as a colorless solid. ^1^H NMR (500 MHz, DMSO-*d*_6_) δ 9.16–9.10 (m, 1H), 8.83–8.79 (m, 1H), 8.45 (t, *J* = 1.7 Hz, 1H), 8.12 (dt, *J* = 7.8, 1.4 Hz, 1H), 8.02 (dt, *J* = 7.9, 1.4 Hz, 1H), 7.65 (t, *J* = 7.8 Hz, 1H), 4.30 (q, *J* = 7.1 Hz, 2H), 3.52–3.43 (m, 4H), 2.69 (s, 3H), 2.68 (s, 3H), 1.30 (t, *J* = 7.1 Hz, 3H). ^13^C NMR (126 MHz, DMSO) δ 177.68, 167.26, 165.61, 165.23, 161.13, 159.68, 158.70, 135.45, 130.12, 129.38, 129.34, 126.39, 125.90, 125.80, 61.57, 39.46 (2C), 17.14, 14.03, 12.05. HPLC/HRMS (ESI): *m*/*z* calculated for C_20_H_22_N_5_O_5_S^+^ [M + H]^+^ 444.1336, found 444.1349. *R*_t_ (4 min): 2.80 min.

##### Ethyl 3-Methyl-5-(3-(3-(5-methyl-1,2,4-oxadiazol-3-yl)benzamido)propanamido)thiophene-2-carboxylate (**26**)

Prepared as described for **19** using ethyl 5-amino-3-methyl-thiophene-2-carboxylate (40.0 mg, 0.22 mmol) and **60** (50.0 mg, 0.18 mmol). Yield: 6.8 mg (0.0150 mmol, 8.0%), amorphous colorless solid. ^1^H NMR (500 MHz, DMSO-*d*_6_) δ 11.58 (s, 1H), 8.88 (t, *J* = 5.6 Hz, 1H), 8.46 (d, *J* = 1.8 Hz, 1H), 8.13 (dt, *J* = 7.9, 1.4 Hz, 1H), 8.03 (dt, *J* = 7.7, 1.4 Hz, 1H), 7.65 (t, *J* = 7.8 Hz, 1H), 6.52 (s, 1H), 4.20 (q, *J* = 7.1 Hz, 2H), 3.58 (q, *J* = 6.5 Hz, 2H), 2.71 (t, *J* = 6.9 Hz, 2H), 2.68 (s, 3H), 2.40 (s, 3H), 1.26 (t, *J* = 7.1 Hz, 3H) ppm. ^13^C NMR (126 MHz, DMSO) δ 178.2, 169.1, 167.7, 166.0, 163.1, 144.5, 144.4, 135.7, 130.5, 129.9 (2C), 126.9, 126.2, 116.0, 115.6, 60.3, 36.3, 35.6, 16.2, 14.8, 12.5 ppm. HPLC/HRMS (ESI): *m*/*z* calculated for C_21_H_22_N_4_O_5_SNa^+^ [M + Na]^+^ 465.1203, found 465.1203. *R*_t_ (2 min): 1.32 min.

##### Ethyl 2-Methyl-6-(3-(3-(5-methyl-1,2,4-oxadiazol-3-yl)benzamido)propanamido)nicotinate (**27**)

Prepared as described for **19** using ethyl 6-amino-2-methyl-pyridine-3-carboxylate (47.1 mg, 0.262 mmol) and **60** (60.0 mg, 0.218 mmol). Yield: 17 mg (0.0389 mmol, 18%), off-white powder. ^1^H NMR (500 MHz, DMSO-*d*_6_) δ 10.83 (s, 1H), 8.82 (t, *J* = 5.5 Hz, 1H), 8.46 (t, *J* = 1.8 Hz, 1H), 8.20 (d, *J* = 8.7 Hz, 1H), 8.12 (dt, *J* = 7.8, 1.4 Hz, 1H), 8.07–8.02 (m, 2H), 7.65 (t, *J* = 7.8 Hz, 1H), 4.28 (q, *J* = 7.1 Hz, 2H), 3.61–3.55 (m, 2H), 2.74 (t, *J* = 6.9 Hz, 2H), 2.68 (s, 3H), 2.65 (s, 3H), 1.31 (t, *J* = 7.1 Hz, 3H). ^13^C NMR (126 MHz, DMSO) δ 177.70, 171.03, 167.27, 165.43, 165.37, 158.59, 153.39, 140.82, 135.33, 130.09, 129.40, 129.37, 126.41, 125.78, 119.93, 110.24, 60.63, 36.07, 35.70, 24.03, 14.13, 12.06. HPLC/HRMS (ESI): *m*/*z* calculated for C_22_H_24_N_5_O_5_^+^ [M + H]^+^ 438.1772, found 438.1783. *R*_t_ (4 min): 2.81 min.

##### Propyl 1-Methyl-3-(3-(3-(5-methyl-1,2,4-oxadiazol-3-yl)benzamido)propanamido)-1*H*-pyrazole-5-carboxylate (**28**)

5-Amino-2-methyl-pyrazole-3-carboxylic acid (30.0 mg, 0.213 mmol) and *N*,*N*-dimethylpyridin-4-amine (2.6 mg, 0.0213 mmol) were dissolved in dry DCM (1.50 mL) at rt. *N*,*N*′-Dicyclohexylmethanediimine (0.26 mL, 0.255 mmol) and propan-1-ol (0.16 mL, 2.13 mmol) were added, and the reaction mixture was stirred at rt overnight. The reaction mixture was concentrated in vacuo and purified by NP chromatography (20–50% EtOAc/cyclohexanes) to give propyl 3-amino-1-methyl-1*H*-pyrazole-5-carboxylate (33 mg, 85%, 0.180 mmol) as a white powder. ^1^H NMR (500 MHz, CDCl_3_) δ 6.14 (s, 1H), 4.21 (t, *J* = 6.6 Hz, 2H), 3.99 (s, 3H), 3.61 (s, 2H), 1.80–1.71 (m, 2H), 1.00 (t, *J* = 7.4 Hz, 3H). HPLC/MS (ESI): *m*/*z* 184.1074 [M + H]^+^. *R*_t_ (2 min): 1.05 min.

3-[[3-(5-Methyl-1,2,4-oxadiazol-3-yl)benzoyl]amino]propanoic acid **19** (49.6 mg, 0.180 mmol), propyl 5-amino-2-methyl-pyrazole-3-carboxylate (30.0 mg, 0.164 mmol), and HATU (87.2 mg, 0.229 mmol) were dissolved in dry DMF (1.09 mL) at rt. DIPEA (0.04 mL, 0.246 mmol) was added, and the reaction mixture was stirred overnight at rt. The mixture was diluted with EtOAc and washed with a solution of NH_4_Cl and water (×2), dried over MgSO_4_, and concentrated in vacuo. The residue was purified via RP column chromatography (30–100% MeOH/H_2_O + 0.1% formic acid) to give propyl 1-methyl-3-(3-(3-(5-methyl-1,2,4-oxadiazol-3-yl)benzamido)propanamido)-1*H*-pyrazole-5-carboxylate (54 mg, 75%, 0.123 mmol) as a white powder. ^1^H NMR (500 MHz, DMSO-*d*_6_) δ 10.68 (s, 1H), 8.81 (t, *J* = 5.5 Hz, 1H), 8.47 (t, *J* = 1.8 Hz, 1H), 8.12 (dt, *J* = 7.8, 1.4 Hz, 1H), 8.03 (dt, *J* = 7.9, 1.5 Hz, 1H), 7.65 (t, *J* = 7.8 Hz, 1H), 7.05 (s, 1H), 4.20 (t, *J* = 6.5 Hz, 2H), 3.98 (s, 3H), 3.58–3.51 (m, 2H), 2.68 (s, 3H), 2.63 (t, *J* = 7.0 Hz, 2H), 1.73–1.65 (m, 2H), 0.95 (t, *J* = 7.4 Hz, 3H). ^13^C NMR (126 MHz, DMSO) δ 177.69, 168.96, 167.27, 165.33, 159.05, 145.59, 135.33, 131.65, 130.06, 129.39, 129.36, 126.41, 125.77, 101.19, 66.19, 38.74, 35.88, 35.35, 21.46, 12.05, 10.30. HPLC/HRMS (ESI): *m*/*z* calculated for C_21_H_25_N_6_O_5_^+^ [M + H]^+^ 441.1881, found 441.1867. *R*_t_ (4 min): 2.73 min.

##### Ethyl 1-Methyl-4-(3-(3-(5-methyl-1,2,4-oxadiazol-3-yl)benzamido)propanamido)-1*H*-imidazole-2-carboxylate (**29**)

Prepared as described for **19** using (2-ethoxycarbonyl-1-methyl-imidazol-4-yl)ammonium chloride (55.0 mg, 0.267 mmol) and **60** (62.0 mg, 0.225 mmol). Yield: 4.8 mg (0.011 mmol, 5%), white powder. ^1^H NMR (500 MHz, DMSO) δ 10.72 (s, 1H), 8.80 (t, *J* = 5.6 Hz, 1H), 8.46 (t, *J* = 1.7 Hz, 1H), 8.12 (dt, *J* = 7.7, 1.3 Hz, 1H), 8.03 (dt, *J* = 7.9, 1.4 Hz, 1H), 7.65 (t, *J* = 7.8 Hz, 1H), 7.54 (s, 1H), 4.25 (q, *J* = 7.1 Hz, 2H), 3.90 (s, 3H), 3.55 (q, *J* = 6.8 Hz, 2H), 2.68 (s, 3H), 2.60 (t, *J* = 7.2 Hz, 2H), 1.28 (t, *J* = 7.1 Hz, 3H). ^13^C NMR (151 MHz, DMSO) δ 177.71, 168.22, 167.28, 165.30, 158.44, 137.45, 135.35, 130.82, 130.06, 129.39, 129.37, 126.42, 125.77, 114.84, 60.53, 35.97, 35.40, 35.06, 14.04, 12.05. HPLC/HRMS (ESI): *m*/*z* calculated for C_20_H_23_N_6_O_5_^+^ [M + H]^+^ 427.1724, found 427.1721. *R*_t_ (2 min): 1.14 min.

##### (3*S*)-3-[[3-(5-Methyl-1,2,4-oxadiazol-3-yl)benzoyl]amino]butanoic acid (**66**)

Prepared as for (3*R*)-3-[[3-(5-methyl-1,2,4-oxadiazol-3-yl)benzoyl]amino]butanoic acid shown below using *tert*-butyl (3*S*)-3-aminobutanoate (156 mg, 0.98 mmol). Yield (two steps): 212 mg (0.73 mmol, 74%). ^1^H NMR (500 MHz, DMSO) δ 12.20 (s, 1H), 8.57 (d, *J* = 8.0 Hz, 1H), 8.45 (t, *J* = 1.7 Hz, 1H), 8.13 (dt, *J* = 7.8, 1.4 Hz, 1H), 8.02 (ddd, *J* = 7.8, 1.9, 1.2 Hz, 1H), 7.65 (t, *J* = 7.8 Hz, 1H), 4.37 (dq, *J* = 7.9, 6.7 Hz, 1H), 2.69 (s, 3H), 2.60 (dd, *J* = 15.4, 6.9 Hz, 1H), 2.43 (dd, *J* = 15.4, 7.2 Hz, 1H), 1.20 (d, *J* = 6.7 Hz, 3H). HPLC/HRMS (ESI): *m*/*z* 290.1143 [M + H]^+^. *R*_t_ (2 min): 1.16 min.

##### Ethyl 4-Methyl-2-[[(3*S*)-3-[[3-(5-methyl-1,2,4-oxadiazol-3-yl)benzoyl]amino]butanoyl]amino]thiazole-5-carboxylate (**30**)

Prepared as described for **31** using (3*S*)-3-[[3-(5-methyl-1,2,4-oxadiazol-3-yl)benzoyl]amino]butanoic acid **66** (98.0 mg, 0.34 mmol). Yield: 15 mg (0.033 mmol, 15%), white powder. ^1^H NMR (500 MHz, DMSO-*d*_6_) δ 12.55 (s, 1H), 8.64 (d, *J* = 8.0 Hz, 1H), 8.44 (dt, *J* = 1.7, 1.0 Hz, 1H), 8.15–8.09 (m, 1H), 8.01 (ddd, *J* = 7.8, 1.9, 1.2 Hz, 1H), 7.65 (dt, *J* = 7.6, 0.6 Hz, 1H), 4.51 (dt, *J* = 13.9, 7.0 Hz, 1H), 4.22 (q, *J* = 7.1 Hz, 2H), 2.83–2.70 (m, 2H), 2.69 (s, 3H), 2.53 (s, 3H), 1.26 (t, *J* = 7.1 Hz, 3H), 1.23 (d, *J* = 6.7 Hz, 3H). ^13^C NMR (126 MHz, DMSO) δ 177.70, 170.00, 167.27, 164.66, 162.11, 159.42, 156.13, 135.49, 130.23, 129.40, 129.32, 126.36, 125.76, 113.81, 60.46, 42.63, 41.75, 20.27, 17.01, 14.19, 12.06. HPLC/HRMS (ESI): *m*/*z* calculated for C_21_H_24_N_5_O_5_S^+^ [M + H]^+^ 458.1493, found 458.1498. *R*_t_ (4 min): 2.82 min.

##### *tert*-Butyl (3*R*)-3-[[3-(5-Methyl-1,2,4-oxadiazol-3-yl)benzoyl]amino]butanoate (**65**)

To *tert*-butyl (3R)-3-aminobutanoate (156.0 mg, 0.980 mmol) were added anhydrous DMF (4.90 mL), 3-(5-methyl-1,2,4-oxadiazol-3-yl)benzoic acid (200.0 mg, 0.980 mmol), DIPEA (0.51 mL, 2.94 mmol), and HATU (345.7 mg, 1.47 mmol). The reaction mixture was capped and stirred at rt overnight. Water (70 mL) was added, and the mixture extracted with EtOAc (2 × 60 mL). The combined organics were washed with sat. NaHCO_3_ and brine, dried over MgSO_4_, and concentrated in vacuo to give *tert*-butyl (3*R*)-3-[[3-(5-methyl-1,2,4-oxadiazol-3-yl)benzoyl]amino]butanoate (335 mg, 99%, 0.970 mmol) as a clear viscous oil. Used without further purification. ^1^H NMR (500 MHz, CDCl_3_) δ 8.41 (t, *J* = 1.7 Hz, 1H), 8.18 (dt, *J* = 7.7, 1.4 Hz, 1H), 7.95 (ddd, *J* = 7.8, 1.9, 1.2 Hz, 1H), 7.56 (td, *J* = 7.7, 0.5 Hz, 1H), 7.02 (d, *J* = 8.5 Hz, 1H), 4.55 (ddt, *J* = 8.6, 6.9, 5.3 Hz, 1H), 2.67 (s, 3H), 2.64–2.48 (m, 2H), 1.48 (s, 9H), 1.34 (d, *J* = 6.7 Hz, 3H). HPLC/HRMS (ESI): *m*/*z* 368.1573 [M + H]^+^. *R*_t_ (2 min): 1.27 min.

##### Ethyl 4-Methyl-2-[[(3*R*)-3-[[3-(5-methyl-1,2,4-oxadiazol-3-yl)benzoyl]amino]butanoyl]amino]thiazole-5-carboxylate (**31**)

To *tert*-butyl (3*R*)-3-[[3-(5-methyl-1,2,4-oxadiazol-3-yl)benzoyl]amino]butanoate (167.0 mg, 0.484 mmol) in DCM (1.50 mL) was added TFA (0.37 mL, 4.84 mmol), and the mixture stirred for 2 h at rt. Toluene was added and then evaporated followed by drying to yield (3*R*)-3-[[3-(5-methyl-1,2,4-oxadiazol-3-yl)benzoyl]amino]butanoic acid **67** (142 mg, 102%, 0.491 mmol) as colorless solid. HPLC/HRMS (ESI): *m*/*z* [M + H]^+^ 290.11. *R*_t_ (2 min): 1.16 min. To **67** (140.0 mg, 0.484 mmol), ethyl 2-amino-4-methyl-thiazole-5-carboxylate (90.1 mg, 0.484 mmol), and HOBt (130.8 mg, 0.968 mmol) in DMF (2.42 mL, 0.200 M) was added EDC·HCl (185.54 mg, 0.968 mmol). The reaction mixture was stirred for 18 h at 60 °C. Water (50 mL) was added, and this was extracted with EtOAc (2 × 35 mL). The combined organics were washed with sat. NaHCO_3_ (25 mL) and brine (25 mL), dried over MgSO_4_, and concentrated in vacuo to give a white solid. This purified by RP column chromatography (eluent: 20–80% MeOH/H_2_O + 0.1% formic acid) to yield **31** (10 mg, 5%, 0.0219 mmol) as a white powder. ^1^H NMR (500 MHz, DMSO-*d*_6_) δ 12.55 (s, 1H), 8.64 (d, *J* = 8.0 Hz, 1H), 8.44 (t, *J* = 1.8 Hz, 1H), 8.12 (dt, *J* = 7.8, 1.4 Hz, 1H), 8.01 (dt, *J* = 7.9, 1.4 Hz, 1H), 7.65 (t, *J* = 7.8 Hz, 1H), 4.57–4.44 (m, 1H), 4.22 (q, *J* = 7.1 Hz, 2H), 2.78 (dd, *J* = 14.9, 7.2 Hz, 1H), 2.72 (dd, *J* = 14.9, 6.8 Hz, 1H), 2.69 (s, 3H), 2.53 (s, 3H), 1.26 (t, *J* = 7.1 Hz, 3H), 1.23 (d, *J* = 6.7 Hz, 3H). ^13^C NMR (126 MHz, DMSO) δ 177.69, 169.99, 167.27, 164.66, 162.10, 159.41, 156.12, 135.49, 130.22, 129.39, 129.31, 126.36, 125.75, 113.81, 60.45, 42.63, 41.74, 20.27, 17.00, 14.19, 12.05. HPLC/HRMS (ESI): *m*/*z* calculated for C_21_H_24_N_5_O_5_S^+^ [M + H]^+^ 458.1493, found 458.1490. *R*_t_ (4 min): 2.88 min.

##### Propyl 4-Methyl-2-(3-(3-(5-methyl-1,2,4-oxadiazol-3-yl)benzamido)propanamido)thiazole-5-carboxylate (**32**)

2-Amino-4-methyl-thiazole-5-carboxylic acid (2.5 g, 15.8 mmol), EDC·HCl (3.6 g, 19 mmol) and propan-1-ol (23.7 mL, 316 mmol) were suspended in dry DMF (90 mL) under nitrogen before *N*,*N*-dimethylpyridin-4-amine (193 mg, 1.6 mmol) was added. The mixture was heated to 65 °C for 2 h 30 min. The solution was then cooled and partitioned between EtOAc (300 mL) and water (750 mL). The organic layer was washed with an aq. sat. NaHCO_3_ solution (250 mL) and brine (250 mL), dried over MgSO_4_, filtered, and concentrated in vacuo. The obtained crude was first purified by a SCX-II cartridge (20 g, 70 mL) using MeOH and 2 N NH_3_ in MeOH as eluents. The combined and evaporated basic residue was purified via NP column chromatography (2–30% MeOH/DCM) to give propyl 2-amino-4-methyl-thiazole-5-carboxylate (971 mg, 31%, 4.8 mmol) as a pale-yellow amorphous powder. ^1^H NMR (500 MHz, DMSO-*d*_6_) δ 7.71 (s, 2H), 4.06 (t, *J* = 6.5 Hz, 2H), 2.37 (s, 3H), 1.62 (dtd, *J* = 13.9, 7.4, 6.5 Hz, 2H), 0.91 (t, *J* = 7.4 Hz, 3H). HPLC/MS (ESI): *m*/*z* 201.0686 [M + H]^+^. *R*_t_ (2 min): 1.01 min.

Propyl 2-amino-4-methyl-thiazole-5-carboxylate (24 mg, 0.12 mmol) and **60** (42 mg, 0.16 mmol) were used in the procedure described for **19** to yield propyl 4-methyl-2-(3-(3-(5-methyl-1,2,4-oxadiazol-3-yl)benzamido)propanamido)thiazole-5-carboxylate (30 mg, 55%, 0.0233 mmol) as an off-white-colored solid. ^1^H NMR (500 MHz, DMSO-*d*_6_) δ 12.54 (s, 1H), 8.86 (t, *J* = 5.5 Hz, 1H), 8.46 (t, *J* = 1.7 Hz, 1H), 8.12 (dt, *J* = 7.7, 1.4 Hz, 1H), 8.05–8.01 (m, 1H), 7.65 (t, *J* = 7.8 Hz, 1H), 4.15 (t, *J* = 6.5 Hz, 2H), 3.60 (q, *J* = 6.6 Hz, 2H), 2.78 (t, *J* = 6.8 Hz, 2H), 2.68 (s, 3H), 2.53 (s, 3H), 1.72–1.62 (m, 2H), 0.93 (t, *J* = 7.4 Hz, 3H). ^13^C NMR (126 MHz, DMSO) δ 177.7, 170.5, 167.2, 165.4, 162.2, 159.5, 156.1, 135.2, 130.1, 129.4, 129.4, 126.4, 125.8, 113.9, 65.8, 35.4, 35.0, 21.6, 17.0, 12.0, 10.3. HPLC/HRMS (ESI): *m*/*z* calculated for C_21_H_24_N_5_O_5_S^+^ [M + H]^+^ 458.1493, found 458.1484. *R*_t_ (4 min): 2.93 min.

##### Propyl 2-[[(3*S*)-6-(*tert*-Butoxycarbonylamino)-3-[[3-(5-methyl-1,2,4-oxadiazol-3-yl)benzoyl]amino]hexanoyl]amino]-4-methyl-thiazole-5-carboxylate (**74**)

3-(5-Methyl-1,2,4-oxadiazol-3-yl)benzoic acid (124 mg, 0.61 mmol, 1 equiv) and HATU (231 mg, 0.61 mmol, 1 equiv) were dissolved in dry DMF (3.04 mL). DIPEA (0.21 mL, 1.22 mmol, 2 equiv) was added, and the reaction mixture was stirred for 1 h 25 min at rt before the addition of (3S)-3-amino-6-(*tert*-butoxycarbonylamino)hexanoic acid (150 mg, 0.61 mmol, 1 equiv). The reaction mixture was stirred at rt for 16 h then diluted with brine (50 mL) and extracted with EtOAc (3 × 40 mL). The combined organics were washed with brine, dried over MgSO_4_, and concentrated in vacuo to afford crude (3*S*)-6-(*tert*-butoxycarbonylamino)-3-[[3-(5-methyl-1,2,4-oxadiazol-3-yl)benzoyl]amino]hexanoic acid (378 mg). HPLC/MS: *m*/*z* 455.1894 [M + Na]^+^. *R*_t_ (2 min): 1.41 min.

Crude (3*S*)-6-(*tert*-butoxycarbonylamino)-3-[[3-(5-methyl-1,2,4-oxadiazol-3-yl)benzoyl]amino]hexanoic acid (263 mg, 0.61 mmol), propyl 2-amino-4-methyl-thiazole-5-carboxylate (122 mg, 0.61 mmol, 1 equiv), EDC·HCl (207 mg, 1.08 mmol, 1.8 equiv), and HOBt (146 mg, 1.08 mmol, 1.8 equiv) were dissolved in dry DMF (2.7 mL). The reaction mixture was stirred at 18 h at 60 °C. The reaction mixture was subjected to RP column chromatography (30–100% MeOH/H_2_O + 0.1% formic acid) to give **74** (84 mg, 22% after two steps). ^1^H NMR (500 MHz, DMSO-*d*_6_) δ 8.59 (s, 1H), 8.43 (s, 1H), 8.12 (dt, *J* = 7.8, 1.4 Hz, 1H), 8.01 (d, *J* = 7.8 Hz, 1H), 7.65 (t, *J* = 7.8 Hz, 1H), 6.77 (t, *J* = 5.7 Hz, 1H), 4.54–4.32 (m, 1H), 4.13 (t, *J* = 6.5 Hz, 2H), 2.98–2.81 (m, 2H), 2.80–2.60 (m, 5H), 2.52 (s, 3H), 1.73–1.60 (m, 2H), 1.59–1.49 (m, 2H), 1.47–1.27 (m, 12H), 0.92 (t, *J* = 7.4 Hz, 3H). HPLC/HRMS (ESI): *m*/*z* calculated for C_29_H_39_N_6_O_7_S^+^ [M + H]^+^ 615.2595, found 615.2602. *R*_t_ (4 min): 3.13 min.

##### Propyl (*S*)-2-(6-Amino-3-(3-(5-methyl-1,2,4-oxadiazol-3-yl)benzamido)hexanamido)-4-methylthiazole-5-carboxylate (**33**)

Propyl 2-[[(3*S*)-6-(*tert*-butoxycarbonylamino)-3-[[3-(5-methyl-1,2,4-oxadiazol-3-yl)benzoyl]amino]hexanoyl]amino]-4-methyl-thiazole-5-carboxylate (35 mg, 0.057 mmol, 1 equiv) was dissolved in propanol (1 mL). 4 N HCl in dioxane (1 mL, 4 mmol, 70 equiv) was added, and the reaction mixture was stirred at rt for 50 min. The volatiles were removed in vacuo, and the crude was purified by RP column chromatography (eluant: 30–90% MeOH/H_2_O + 0.1% formic acid modifier in both) to give **33** (13.5 mg, 46%) as a white powder. ^1^H NMR (500 MHz, methanol-*d*_4_) δ 8.44 (t, *J* = 1.7 Hz, 1H), 8.14 (dt, *J* = 7.8, 1.4 Hz, 1H), 7.97–7.90 (m, 1H), 7.57 (t, *J* = 7.8 Hz, 1H), 4.63–4.53 (m, 1H), 4.16 (t, *J* = 6.6 Hz, 2H), 2.87–2.77 (m, 4H), 2.64 (s, 3H), 2.53 (s, 3H), 1.81–1.61 (m, 6H), 0.99 (t, *J* = 7.4 Hz, 3H). ^13^C NMR (126 MHz, MeOD) δ 177.6, 171.5, 167.8, 167.5, 163.1, 162.1, 156.3, 135.3, 129.6, 129.6, 128.9, 127.1, 125.9, 114.3, 65.9, 47.2, 41.1, 40.1, 31.3, 27.2, 21.8, 15.9, 10.7, 9.4. HPLC/MS: *m*/*z* 515.2037 [M + H]^+^. *R*_t_ (2 min): 1.35 min. HPLC/HRMS (ESI): *m*/*z* calculated for C_24_H_31_N_6_O_5_S^+^ [M + H]^+^ 515.2071, found 515.2081. *R*_t_ (4 min): 2.54 min.

##### *tert*-Butyl (*E*)-6-((*tert*-Butoxycarbonyl)(methyl)amino)hex-2-enoate (**77**)

*tert*-Butyl *N*-(4-hydroxybutyl)-*N*-methyl-carbamate (1.00 g, 4.91 mmol, 1 equiv) and Dess–Martin periodinane (2.92 g, 6.88 mmol, 1.4 equiv) were dissolved in DCM (15 mL). The reaction mixture was stirred at rt for 2 h, then diluted with EtOAc and filtered through a Celite. The filtrate was washed with an aq. solution of Na_2_S_2_O_3_, NaHCO_3_, dried over MgSO_4_, and concentrated under reduced pressure to yield *tert*-butyl *N*-methyl-*N*-(4-oxobutyl)carbamate **76** as a colorless oil. The product was used in the next step without further purification. **76** (990 mg, 4.92 mmol, 1 equiv) and *tert*-butyl(triphenylphosphoranylidene)acetate (2.04 g, 5.41 mmol, 1.1 equiv) were dissolved in dry PhMe (16.4 mL). The reaction mixture was heated to 120 °C and stirred for 16 h. The solvent was removed under reduced pressure. Purification by NP column chromatography (eluent: 15–40% EtOAc/cyclohexane) afforded **77** (1.02 g, 70% after 2 steps) as a yellow oil. ^1^H NMR (500 MHz, chloroform-*d*) δ 6.84 (dt, *J* = 15.6, 6.8 Hz, 1H), 5.75 (dt, *J* = 15.7, 1.6 Hz, 1H), 3.28–3.16 (m, 2H), 2.83 (s, 3H), 2.20–2.10 (m, 2H), 1.71–1.61 (m, 2H), 1.46 (d, *J* = 12.3 Hz, 18H). HPLC/MS: *m*/*z* 322.1992 [M + Na]^+^. *R*_t_ (2 min): 1.57 min.

##### *tert*-Butyl (*R*)-3-(Benzyl((*R*)-1-phenylethyl)amino)-6-((*tert*-butoxycarbonyl)(methyl)amino)hexanoate (**80**)

(*R*)-(−)-*N*-Benzyl-α-methylbenzylamine (0.45 mL, 2.14 mmol, 1.6 equiv) was dissolved in dry THF (5.34 mL). The mixture was cooled to −78 °C, *n*-BuLi (2.5 M in hexanes, 0.83 mL, 2.07 mmol, 1.55 equiv) was added, and the reaction mixture was stirred at −78 °C for 15 min. *tert*-Butyl (*E*)-6-[*tert*-butoxycarbonyl(methyl)amino]hex-2-enoate **77** (400 mg, 1.33 mmol, 1 equiv) in dry THF (1.5 mL) was added to the solution. The reaction mixture was stirred at −78 °C for 1 h before being quenched with water. The mixture was quenched with water, extracted with EtOAc, dried over MgSO_4_, and concentrated under reduced pressure. The residue was purified by RP column chromatography (40–100% MeOH/H_2_O + 0.1% formic acid). The desired fractions were filtered through a SCX-2 column, where the compound was released with a solution of 2N NH_3_ in MeOH to give **80** (289 mg, 42%) as a colorless oil. ^1^H NMR (600 MHz, chloroform-*d*) δ 7.45–7.41 (m, 2H), 7.38–7.34 (m, 2H), 7.33–7.30 (m, 4H), 7.29–7.23 (m, 2H), 3.84 (q, *J* = 6.9 Hz, 1H), 3.79 (d, *J* = 14.7 Hz, 1H), 3.51 (d, *J* = 15.1 Hz, 1H), 3.35–3.28 (m, 1H), 3.23–3.10 (m, 2H), 2.84 (s, 3H), 2.00–1.81 (m, 3H), 1.56–1.49 (m, 2H), 1.46 (s, 9H), 1.41 (s, 9H), 1.36 (d, *J* = 7.1 Hz, 3H), 1.33–1.25 (m, 1H). HPLC/MS: *m*/*z* 511.6330 [M + H]^+^. *R*_t_ (2 min): 1.76 min.

##### *tert*-Butyl (*R*)-6-((*tert*-Butoxycarbonyl)(methyl)amino)-3-(3-(5-methyl-1,2,4-oxadiazol-3-yl)benzamido)hexanoate (**84**)

*tert*-Butyl (3*R*)-3-[benzyl-[(1*R*)-1-phenylethyl]amino]-6-[*tert*-butoxycarbonyl(methyl)amino] hexanoate **80** (289 mg, 0.57 mmol, 1 equiv) and Pd(OH)_2_ (159 mg, 0.23 mmol, 0.4 equiv) were dissolved in MeOH (2.53 mL). Ammonium formate (1.25 g, 20 mmol, 35 equiv) was slowly added in small amounts, and the mixture was stirred for 15 min at rt. Formic acid (0.30 mL) was added, and the reaction mixture was stirred at 60 °C for 24 h. The reaction mixture was filtered through Celite, washed with MeOH, and concentrated in vacuo to give *tert*-butyl (*R*)-3-amino-6-((*tert*-butoxycarbonyl)(methyl)amino)hexanoate **82** (140 mg, 78%) as a yellow oil. HPLC/MS: *m*/*z* 317.2307 [M + H]^+^. *R*_t_ (2 min): 1.03 min.

*tert*-Butyl (3*R*)-3-amino-6-[*tert*-butoxycarbonyl(methyl)amino]hexanoate **82** (80 mg, 0.25 mmol), HATU (144 mg, 0.38 mmol, 1.5 equiv), and 3-(5-methyl-1,2,4-oxadiazol-3-yl)benzoic acid (67 mg, 0.33 mmol, 1.3 equiv) were dissolved in dry DMF (2.53 mL). DIPEA (0.13 mL, 0.76 mmol, 3 equiv) was added, and the mixture was stirred at rt for 16 h. The reaction mixture was diluted with EtOAc, and a solution of NaHCO_3_ was added. The product was extracted with EtOAc, dried over MgSO_4_, and concentrated in vacuo. Purification by NP chromatography (eluant: 20–45% EtOAc/cyclohexane) gave **84** (102 mg, 80%) as a pale yellow oil. ^1^H NMR (500 MHz, chloroform-*d*) δ 8.52–8.39 (m, 1H), 8.18 (d, *J* = 7.8 Hz, 1H), 7.97 (s, 1H), 7.56 (t, *J* = 7.8 Hz, 1H), 4.53–4.43 (m, 1H), 3.37–3.13 (m, 2H), 2.82 (s, 3H), 2.67 (s, 3H), 2.63–2.48 (m, 2H), 1.63–1.56 (m, 4H), 1.50–1.37 (m, 18H) (NH not observed). HPLC/MS: *m*/*z* 525.2659 [M + Na]^+^. *R*_t_ (2 min): 1.60 min.

##### Propyl (*R*)-4-Methyl-2-(3-(3-(5-methyl-1,2,4-oxadiazol-3-yl)benzamido)-6-(methylamino)hexanamido)thiazole-5-carboxylate (**34**)

*tert*-Butyl (*R*)-6-((*tert*-butoxycarbonyl)(methyl)amino)-3-(3-(5-methyl-1,2,4-oxadiazol-3-yl)benzamido)hexanoate **84** (100 mg, 0.20 mmol, 1 equiv) and potassium hydroxide (89 mg, 1.59 mmol, 8 equiv) were dissolved in dry THF (1 mL) at rt. A few drops of water (enough to dissolve KOH) and MeOH were added, and the reaction mixture was stirred at 55 °C for 5 h. The solvent was removed in vacuo. The residue was acidified to pH ∼3 with a 1 N solution of HCl. The product was extracted with EtOAc, dried over MgSO_4_, and concentrated in vacuo to give (*R*)-6-((*tert*-butoxycarbonyl)(methyl)amino)-3-(3-(5-methyl-1,2,4-oxadiazol-3-yl)benzamido)hexanoic acid a yellow thick oil (88 mg, used in the next reaction without further purification). HPLC/MS: *m*/*z* 447.2236 [M + H]^+^. *R*_t_ (2 min): 1.32 min.

(*R*)-6-((*tert*-Butoxycarbonyl)(methyl)amino)-3-(3-(5-methyl-1,2,4-oxadiazol-3-yl)benzamido)hexanoic acid (88 mg, 0.20 mmol, 1 equiv), HOBt (53 mg, 0.40 mmol, 2 equiv), EDC·HCl (75 mg, 0.40 mmol, 2 equiv), and propyl 2-amino-4-methyl-thiazole-5-carboxylate (47 mg, 0.23 mmol, 1.3 equiv) were dissolved in dry DMF (1.31 mL). The reaction mixture was warmed to 50 °C and stirred for 48 h. The reaction mixture was diluted with EtOAc and quenched with NH_4_Cl. The organic layer was washed three times with water, dried over MgSO_4_, and concentrated in vacuo. Purification using NP column chromatography (eluent: 30–65% EtOAc/cyclohexane, then 0% to 8% MeOH/EtOAc ) to give propyl (*R*)-2-(6-((*tert*-butoxycarbonyl)(methyl)amino)-3-(3-(5-methyl-1,2,4-oxadiazol-3-yl)benzamido)hexanamido)-4-methylthiazole-5-carboxylate (82 mg, 66%) as a yellow powder. ^1^H NMR (500 MHz, DMSO) δ 12.55 (s, 1H), 8.58 (d, *J* = 8.6 Hz, 1H), 8.44 (s, 1H), 8.12 (dt, *J* = 7.7, 1.4 Hz, 1H), 8.04–7.97 (m, 1H), 7.65 (t, *J* = 7.8 Hz, 1H), 4.46 (s, 1H), 4.14 (t, *J* = 6.5 Hz, 2H), 3.22–3.09 (m, 2H), 2.74 (d, *J* = 8.4 Hz, 5H), 2.68 (s, 3H), 2.53 (s, 3H), 1.66 (dt, *J* = 7.7, 6.8 Hz, 2H), 1.49 (d, *J* = 32.6 Hz, 4H), 1.32 (d, *J* = 16.8 Hz, 9H), 0.92 (t, *J* = 7.4 Hz, 3H). HPLC/MS: *m*/*z* 629.2656 [M + H]^+^. *R*_t_ (2 min): 1.65 min.

Propyl (*R*)-2-(6-((*tert*-Butoxycarbonyl)(methyl)amino)-3-(3-(5-methyl-1,2,4-oxadiazol-3-yl)benzamido)hexanamido)-4-methylthiazole-5-carboxylate (80 mg, 0.13 mmol, 1 equiv) was dissolved in dry propanol (1.27 mL). 4 N HCl in dioxane (0.80 mL, 3.18 mmol, 25 equiv) was added, and the reaction mixture was stirred at rt for 3.5 h. The solvent was removed in vacuo, and the product was dissolved in DMSO (2 mL + 0.3 mL) and purified via RP column chromatography (30–100% MeOH/H_2_O + 0.1% formic acid). The desired fractions were filtered through a 5 g SCX-2 column, and the compound was released with a solution of 2 N NH_3_ in MeOH to give **34** (45 mg, 67%) as a white powder. ^1^H NMR (600 MHz, methanol-*d*_4_) δ 8.45 (t, *J* = 1.7 Hz, 1H), 8.16 (dt, *J* = 7.8, 1.4 Hz, 1H), 7.97–7.93 (m, 1H), 7.59 (t, *J* = 7.8 Hz, 1H), 4.63–4.53 (m, 1H), 4.18 (t, *J* = 6.5 Hz, 2H), 2.85–2.79 (m, 2H), 2.77–2.70 (m, 2H), 2.65 (s, 3H), 2.55 (s, 3H), 2.45 (s, 3H), 1.80–1.63 (m, 6H), 1.00 (t, *J* = 7.4 Hz, 3H). ^13^C NMR (151 MHz, MeOD) δ 179.01, 172.35, 169.26, 168.98, 164.47, 162.75, 157.79, 136.78, 131.06, 131.00, 130.30, 128.57, 127.29, 115.93, 67.35, 51.61, 48.56, 42.32, 35.45, 32.88, 26.15, 23.17, 17.33, 12.10, 10.81. HPLC/HRMS (ESI): *m*/*z* calculated for C_25_H_32_N_6_O_5_SNa^+^ [M + Na]^+^ 551.2047, found 551.2037. *R*_t_ (4 min): 2.54 min.

##### *tert*-Butyl (*S*)-3-(Benzyl((*S*)-1-phenylethyl)amino)-6-((*tert*-butoxycarbonyl)(methyl)amino)hexanoate (**81**)

(*S*)-(−)-*N*-Benzyl-α-methylbenzylamine (1.26 mL, 6.02 mmol, 1.2 equiv) was dissolved in dry THF (11.2 mL). The mixture was cooled to −78 °C, *n*-BuLi (2.25 M in hexanes, 2.67 mL, 6.02 mmol, 1.2 equiv) was added, and the reaction mixture was stirred at −78 °C for 15 min. **77** (1.50 g, 5.00 mmol, 1 equiv) in dry THF (1.5 mL) was added to the solution. The reaction mixture was stirred at −78 °C for 1 h before being quenched with water. The product was extracted with EtOAc, dried over MgSO_4_, and concentrated under reduced pressure to afford compound **81** (117 mg, 69%) as a yellow oil. ^1^H NMR (600 MHz, chloroform-*d*) δ 7.43 (d, *J* = 7.2 Hz, 2H), 7.36 (t, *J* = 7.5 Hz, 2H), 7.32 (d, *J* = 4.3 Hz, 4H), 7.28–7.24 (m, 2H), 3.84 (q, *J* = 7.0 Hz, 1H), 3.79 (d, *J* = 14.9 Hz, 1H), 3.51 (d, *J* = 14.9 Hz, 1H), 3.32 (tt, *J* = 8.8, 4.1 Hz, 1H), 3.17 (ddd, *J* = 14.1, 8.0, 6.0 Hz, 2H), 2.84 (s, 3H), 1.98–1.84 (m, 3H), 1.56–1.49 (m, 2H), 1.46 (s, 9H), 1.41 (s, 9H), 1.37 (d, *J* = 7.0 Hz, 3H), 1.32–1.23 (m, 1H). HPLC/MS: *m*/*z* 511.3548 [M + Na]^+^. *R*_t_ (2 min): 1.73 min.

##### *tert*-Butyl (*S*)-3-Amino-6-((*tert*-butoxycarbonyl)(methyl)amino)hexanoate (**83**)

MeOH (18.7 mL) was added to Pd(OH)_2_ (351 mg, 0.50 mmol, 0.1 equiv). Ammonium formate (1.57 g, 25.00 mmol, 5 equiv) was slowly added in small amounts over 5 min (Caution! Gas evolved), and the mixture was stirred for 10 min at rt. **81** (2.55 g, 5.00 mmol, 1 equiv) in MeOH (2 mL) was slowly added to the mixture. The mixture was stirred for 15 min, then formic acid (1.27 mL) was added. The reaction mixture was stirred 60 °C for 16 h. Ammonium formate (1.57 g, 25.00 mmol, 5 equiv), formic acid (0.6 mL), and Pd(OH)_2_ (70 mg, 0.25 mmol, 0.05 equiv) were added, and the reaction mixture was stirred at 60 °C for 24 h. The reaction mixture was cooled to rt then filtered through Celite, washed with MeOH, concentrated under reduced pressure. The residue was purified by NP column chromatography (eluent: 0–10% MeOH/DCM) to yield **83** (800 mg, 51%) as a colorless oil. ^1^H NMR (500 MHz, chloroform-*d*) δ 3.28–3.10 (m, 3H), 2.83 (s, 3H), 2.38 (dd, *J* = 15.6, 3.9 Hz, 1H), 2.19 (dd, *J* = 15.6, 8.8 Hz, 1H), 1.66–1.48 (m, 2H), 1.45 (s, 18H), 1.42–1.28 (m, 2H) (2 x NH not observed). HPLC/MS: *m*/*z* 317.2426 [M + H]^+^. *R*_t_ (2 min): 1.12 min.

##### *tert*-Butyl (*S*)-6-((*tert*-Butoxycarbonyl)(methyl)amino)-3-(3-(5-methyl-1,2,4-oxadiazol-3-yl)benzamido)hexanoate (**85**)

3-(5-Methyl-1,2,4-oxadiazol-3-yl)benzoic acid (484 mg, 2.37 mmol, 1.5 equiv) and **83** (500 mg, 1.58 mmol, 1.0 equiv) were dissolved in dry DMF (8.0 mL). Triethylamine (0.66 mL, 4.74 mmol, 3 equiv) and T_3_P (50% in DMF, 0.70 mL, 1.5 equiv) were added to the reaction mixture. The reaction mixture was stirred at rt for 2 h, then diluted with EtOAc and water. The organic layer was washed with NaHCO_3_, dried over MgSO_4_, and concentrated in vacuo. The residue was purified by NP column chromatography (eluent: 20–50% EtOAc/cyclohexane) to yield **85** (630 mg, 79%) as a colorless oil. ^1^H NMR (600 MHz, chloroform-*d*) δ 8.52–8.43 (m, 1H), 8.21 (d, *J* = 7.7 Hz, 1H), 8.04–7.95 (m, 1H), 7.58 (t, *J* = 7.8 Hz, 1H), 4.55–4.46 (m, 1H), 3.38–3.18 (m, 2H), 2.84 (s, 3H), 2.69 (s, 3H), 2.67–2.50 (m, 2H), 2.19 (s, 2H), 1.69–1.57 (m, 5H), 1.48 (s, 9H), 1.46 (s, 9H). ^13^C NMR (151 MHz, CDCl_3_) δ 176.9, 171.4, 168.0, 166.1, 135.6, 130.3, 130.1, 129.4, 127.4, 125.6, 79.5, 48.1, 47.2, 40.4, 34.2, 31.7, 28.6, 28.2, 24.9, 12.6. HPLC/MS: *m*/*z* 525.2681 [M + Na]^+^*R*_t_ (2 min): 1.50 min.

##### Propyl (*S*)-4-Methyl-2-(3-(3-(5-methyl-1,2,4-oxadiazol-3-yl)benzamido)-6-(methylamino)hexanamido)thiazole-5-carboxylate (**35**)

**85** (430 mg, 0.85 mmol, 1 equiv) and potassium hydroxide (960 mg, 17.1 mmol, 20 equiv) were dissolved in THF (4.30 mL). A few drops of water and MeOH (enough to dissolve all the KOH) were added, and the reaction mixture was stirred at 50 °C for 5 h. The pH was adjusted to pH 3 with a 1 M solution of HCl. The product was extracted with EtOAc, dried over MgSO_4_, and concentrated in vacuo. The crude was dissolved in DMSO (1.5 mL + 0.3 mL) and purified via column chromatography (30–100% MeOH/H2O + 0.1% formic acid) to give (*S*)-6-((*tert*-butoxycarbonyl)(methyl)amino)-3-(3-(5-methyl-1,2,4-oxadiazol-3-yl)benzamido)hexanoic acid (170 mg, 44%) as a white powder. ^1^H NMR (500 MHz, methanol-*d*_4_) δ 8.51 (t, *J* = 1.7 Hz, 1H), 8.20 (dt, *J* = 7.7, 1.4 Hz, 1H), 7.98 (dt, *J* = 7.9, 1.6 Hz, 1H), 7.63 (t, *J* = 7.8 Hz, 1H), 4.58–4.46 (m, 1H), 3.31–3.21 (m, 2H), 2.85 (s, 3H), 2.68 (s, 3H), 2.66–2.59 (m, 2H), 1.77–1.58 (m, 4H), 1.44 (s, 9H). HPLC/MS: *m*/*z* 469.2066 [M + Na]^+^. *R*_t_ (2 min): 1.32 min.

(*S*)-6-((*tert*-Butoxycarbonyl)(methyl)amino)-3-(3-(5-methyl-1,2,4-oxadiazol-3-yl)benzamido)hexanoic acid (80 mg, 0.18 mmol, 1 equiv), EDC·HCl (69 mg, 0.36 mmol, 2 equiv), HOBt (48 mg, 0.36 mmol, 2 equiv), and propyl 2-amino-4-methyl-thiazole-5-carboxylate (53 mg, 0.27 mmol, 1.5 equiv) were dissolved in dry DMF (0.90 mL). The reaction mixture was stirred at 45 °C for 16 h, then diluted with EtOAc and water. The organic layer was washed with NaHCO_3_, dried over MgSO_4_, and concentrated in vacuo. The crude was dissolved in DMSO (0.5 mL + 0.3 mL) and purified via RP column chromatography (40–90% MeOH/H_2_O + 0.1% formic acid) to give propyl (*S*)-2-(6-((*tert*-butoxycarbonyl)(methyl)amino)-3-(3-(5-methyl-1,2,4-oxadiazol-3-yl)benzamido)hexanamido)-4-methylthiazole-5-carboxylate (90 mg, 80%) as a white powder. ^1^H NMR (600 MHz, DMSO-*d*_6_) δ 12.54 (s, 1H), 8.57 (d, *J* = 8.5 Hz, 1H), 8.44 (s, 1H), 8.12 (dt, *J* = 7.7, 1.4 Hz, 1H), 8.01 (dt, *J* = 7.8, 1.5 Hz, 1H), 7.65 (t, *J* = 7.8 Hz, 1H), 4.49–4.42 (m, 1H), 4.14 (t, *J* = 6.5 Hz, 2H), 3.22–3.10 (m, 2H), 2.74 (d, *J* = 11.8 Hz, 5H), 2.68 (s, 3H), 2.53 (s, 3H), 1.69–1.62 (m, 2H), 1.58–1.44 (m, 4H), 1.37–1.28 (m, 9H), 0.92 (t, *J* = 7.4 Hz, 3H). HPLC/MS: *m*/*z* 629.2784 [M + H]^+^. *R*_t_ (2 min): 1.54 min.

Propyl (*S*)-2-(6-((*tert*-butoxycarbonyl)(methyl)amino)-3-(3-(5-methyl-1,2,4-oxadiazol-3-yl)benzamido)hexanamido)-4-methylthiazole-5-carboxylate (48 mg, 0.076 mmol, 1 equiv) was dissolved in dry DCM (0.76 mL). Trifluoroacetic acid (116 μL, 1.53 mmol, 20 equiv) was added, and the reaction mixture was stirred at rt for 1 h. The solvent was removed in vacuo. The crude was dissolved in DMSO (0.5 mL + 0.3 mL) and purified via RP column chromatography (40–90% MeOH/H_2_O + 0.1% formic acid). The fractions containing the desired product were filtered through a 1 g SCX-2 column, and the compound was released with a 2 N solution of NH_3_ in MeOH to give **35** (30 mg, 74%) as a white powder. ^1^H NMR (600 MHz, MeOD) δ 8.46 (d, *J* = 1.8 Hz, 1H), 8.17 (dt, *J* = 7.7, 1.4 Hz, 1H), 7.95 (dt, *J* = 7.9, 1.5 Hz, 1H), 7.60 (t, *J* = 7.8 Hz, 1H), 4.60–4.53 (m, 1H), 4.18 (t, *J* = 6.6 Hz, 2H), 2.84–2.76 (m, 2H), 2.73–2.64 (m, 5H), 2.55 (s, 3H), 2.42 (s, 3H), 1.80–1.60 (m, 6H), 1.00 (t, *J* = 7.5 Hz, 3H). ^13^C NMR (151 MHz, MeOD) δ 179.03, 172.53, 169.25, 168.99, 164.51, 163.03, 157.79, 136.82, 131.06, 130.99, 130.31, 128.58, 127.28, 115.86, 67.34, 51.77, 48.61, 42.37, 35.60, 32.93, 26.37, 23.18, 17.32, 12.09, 10.81. HPLC/HRMS (ESI): *m*/*z* calculated for C_25_H_33_N_6_O_5_S^+^ [M + H]^+^ 529.2228, found 529.2235. *R*_t_ (4 min): 2.47 min.

##### Propyl (*S*)-2-(6-(Dimethylamino)-3-(3-(5-methyl-1,2,4-oxadiazol-3-yl)benzamido)hexanamido)-4-methylthiazole-5-carboxylate (**36**)

Propyl (*S*)-2-(6-amino-3-(3-(5-methyl-1,2,4-oxadiazol-3-yl)benzamido)hexanamido)-4-methylthiazole-5-carboxylate **33** (28 mg, 0.054 mmol, 1 equiv) was dissolved in dry DCE (0.52 mL). Formaldehyde (34.5 wt % in water, 126 μL, 4.57 mmol, 84 equiv) and two drops of AcOH were added to the reaction mixture. The reaction mixture was stirred for 15 min before sodium triacetoxyborohydride (35 mg, 0.16 mmol, 3 equiv) was added, and the mixture was stirred at rt for 16 h. The solvent was removed in vacuo, and the crude was partitioned between DCM and a sat. aq. solution of NaHCO_3_. The organic layer was dried over MgSO_4_ and concentrated in vacuo. The crude was purified using a Biotage 11 g KP-NH silica SNAP column that was eluted with 2–10% EtOH/DCM over 15 CV to give **36** (10 mg, 34%) as a clear glass solid. ^1^H NMR (500 MHz, methanol-*d*_4_) δ 8.45 (t, *J* = 1.7 Hz, 1H), 8.17 (dt, *J* = 7.7, 1.4 Hz, 1H), 7.97–7.92 (m, 1H), 7.60 (t, *J* = 7.8 Hz, 1H), 4.62–4.54 (m, 1H), 4.18 (t, *J* = 6.5 Hz, 2H), 2.83 (dd, *J* = 6.7, 2.5 Hz, 2H), 2.65 (s, 3H), 2.55 (s, 3H), 2.54–2.46 (m, 2H), 2.34 (s, 6H), 1.78–1.62 (m, 6H), 1.00 (t, *J* = 7.4 Hz, 3H). ^13^C NMR (126 MHz, MeOD) δ 179.01, 171.43, 169.30, 168.95, 164.29, 161.37, 157.77, 136.76, 131.08, 131.00, 130.31, 128.57, 127.25, 116.34, 67.42, 59.92, 48.53, 45.07, 42.06, 33.03, 24.55, 23.15, 17.31, 12.10, 10.81. HPLC/HRMS (ESI): *m*/*z* calculated for C_26_H_35_N_6_O_5_S^+^ [M + H]^+^ 543.2384, found 543.2401. *R*_t_ (4 min): 2.45 min.

##### 3,3-Dimethyl-1-(6-(((*S*)-4-(3-(5-methyl-1,2,4-oxadiazol-3-yl)benzamido)-6-((4-methyl-5-(propoxycarbonyl)thiazol-2-yl)amino)-6-oxohexyl)amino)-6-oxohexyl)-5-sulfo-2-((1*E*,3*E*)-5-((*E*)-1,3,3-trimethyl-5-sulfoindolin-2-ylidene)penta-1,3-dien-1-yl)-3*H*-indol-1-ium (**37**)

SulfoCy5-NHS ester (1.00 mg, 0.0013 mmol) was dissolved in a mixture of 200 μL of DMF and 20 μL of triethylamine and added to propyl 2-[[(3*S*)-6-amino-3-[[3-(5-methyl-1,2,4-oxadiazol-3-yl)benzoyl]amino]hexanoyl]amino]-4-methyl-thiazole-5-carboxylate **33** (0.69 mg, 0.0013 mmol). The solution was mixed on vortex shaker shielded from light for 16 h at rt. Purification by prep-HPLC (all machine internal lights switched off) afforded **37** (0.96 mg, 62%, 0.0008 mmol) as a blue powder. HPLC/HRMS (ESI): *m*/*z* calculated for C_56_H_68_N_8_O_12_S_3_^2+^ [M + H]^2+^ 570.2054, found 570.2056. *R*_t_ (4 min): 3.06 min.

##### Propyl 2-((*S*)-6-(((((*S*,*E*)-Cyclooct-4-en-1-yl)oxy)carbonyl)(methyl)amino)-3-(3-(5-methyl-1,2,4-oxadiazol-3-yl)benzamido)hexanamido)-4-methylthiazole-5-carboxylate (**38**)

[(1*R*,4*E*)-Cyclooct-4-en-1-yl] (2,5-dioxopyrrolidin-1-yl) carbonate **90** (10.1 mg, 0.0378 mmol) was cooled to 0 °C before propyl (*S*)-4-methyl-2-(3-(3-(5-methyl-1,2,4-oxadiazol-3-yl)benzamido)-6-(methylamino)hexanamido)thiazole-5-carboxylate **35** (20.0 mg, 0.0378 mmol), dry DMF (0.38 mL), and DIPEA (9.9 μL, 0.0568 mmol) were added. The mixture warmed to rt and stirred overnight (protected from light). The mixture was purified via RP column chromatography (40–100% MeOH/H_2_O + 0.1% formic acid) to give **38** as a white powder (18 mg, 70%). ^1^H NMR (600 MHz, DMSO-*d*_6_) δ 12.55 (s, 1H, NH), 8.57 (d, *J* = 8.5 Hz, 1H, NH), 8.50–8.41 (m, 1H), 8.14 (d, *J* = 7.7 Hz, 1H), 8.02 (d, *J* = 7.7 Hz, 1H), 7.67 (t, *J* = 7.7 Hz, 1H), 5.61–5.32 (m, 2H), 4.50–4.41 (m, 1H), 4.15 (t, *J* = 6.4 Hz, 3H), 3.21–3.10 (m, 2H), 2.79–2.73 (m, 4H), 2.70 (s, 3H), 2.56–2.53 (m, 4H), 2.29–2.06 (m, 3H), 1.92–1.63 (m, 6H), 1.63–1.40 (m, 7H), 0.93 (t, *J* = 7.4 Hz, 3H). ^13^C NMR (151 MHz, DMSO) δ 178.2, 170.5, 167.8, 165.4, 162.6, 159.9, 156.5, 135.9, 135.2, 132.9, 130.7, 129.9, 129.8, 126.9, 126.2, 114.3, 80.2, 66.3, 48.2, 46.8, 41.3, 40.9, 40.9, 40.5, 38.4, 34.2, 32.5, 31.5, 31.0, 24.6, 22.1, 17.5, 12.5, 10.8. HPLC/HRMS (ESI): *m*/*z* calculated for C_34_H_45_N_6_O_7_S^+^ [M + H]^+^ 681.3065, found 681.3092. *R*_t_ (4 min): 3.32 min.

##### 2,2-Dimethyl-4-oxo-3,8,11-trioxa-5-azatridecan-13-yl 4-methylbenzenesulfonate (**87**)

2-[2-(2-Aminoethoxy)ethoxy]ethanol (400.0 mg, 2.63 mmol) was dissolved in DCM (7.51 mL). To the mixture was added di-*tert*-butyl dicarbonate (0.86 g, 3.94 mmol), followed by triethylamine (0.55 mL, 3.94 mmol). The reaction mixture was stirred at rt for 16 h. Water was added to the reaction mixture, and the product was extracted with DCM, dried over MgSO_4_, and concentrated in vacuo. The residue was purified by NP chromatography (50–100% EtOAc/cyclohexanes) to give *tert*-butyl (2-(2-(2-hydroxyethoxy)ethoxy)ethyl)carbamate (560 mg, 85%, 2.25 mmol) as a colorless oil. ^1^H NMR (500 MHz, CDCl_3_) δ 5.14 (s, 1H), 3.77–3.72 (m, 2H), 3.68–3.59 (m, 6H), 3.55 (dd, *J* = 5.6, 4.8 Hz, 2H), 3.31 (t, *J* = 5.2 Hz, 2H), 2.42 (s, 1H), 1.44 (s, 9H).

*tert*-Butyl (2-(2-(2-hydroxyethoxy)ethoxy)ethyl)carbamate (250.0 mg, 1.00 mmol) and triethylamine (0.17 mL, 1.2 mmol) were dissolved in DCM (4.00 mL). *p*-Toluene-sulfonyl-chloride (228.8 mg, 1.2 mmol) was added, and the reaction mixture was stirred at rt for 48 h. The solvent was removed under reduced pressure and absorbed onto silica. The residue was purified using NP column chromatography (20–80% EtOAc/cyclohexanes) to give **87** (310 mg, 77%) as a colorless oil. ^1^H NMR (500 MHz, CDCl_3_) δ 7.84–7.76 (m, 2H), 7.38–7.31 (m, 2H), 4.93 (s, 1H), 4.19–4.15 (m, 2H), 3.71–3.66 (m, 2H), 3.60–3.52 (m, 4H), 3.49 (t, *J* = 5.2 Hz, 2H), 3.29 (q, *J* = 5.1 Hz, 2H), 2.44 (s, 3H), 1.43 (s, 9H). HPLC/MS: *m*/*z* 426.1551 [M + Na]^+^. *R*_t_ (2 min): 1.33 min.

##### Propyl (*S*)-2-(6-((2-(2-(2-Aminoethoxy)ethoxy)ethyl)(methyl)amino)-3-(3-(5-methyl-1,2,4-oxadiazol-3-yl)benzamido)hexanamido)-4-methylthiazole-5-carboxylate (**88**)

Propyl (*S*)-4-methyl-2-(3-(3-(5-methyl-1,2,4-oxadiazol-3-yl)benzamido)-6-(methylamino)hexanamido)thiazole-5-carboxylate **35** (58.0 mg, 0.110 mmol) and 2,2-dimethyl-4-oxo-3,8,11-trioxa-5-azatridecan-13-yl 4-methylbenzenesulfonate **87** (53.0 mg, 0.131 mmol) were dissolved in dry DMF (0.30 mL). K_2_CO_3_ (30.3 mg, 0.219 mmol) was added, and the reaction mixture was stirred at rt for 9 days. The reaction mixture was purified via RP column chromatography (40–100% MeOH/H_2_O + 0.1% formic acid) to give propyl (*S*)-4-methyl-2-(2,2,14-trimethyl-18-(3-(5-methyl-1,2,4-oxadiazol-3-yl)benzamido)-4-oxo-3,8,11-trioxa-5,14-diazaicosan-20-amido)thiazole-5-carboxylate (30 mg, 36%, 0.0394 mmol) as a colorless oil. HPLC/MS: *m*/*z* 760.3731 [M + H]^+^. *R*_t_ (4 min): 2.84 min.

This propyl (*S*)-4-methyl-2-(2,2,14-trimethyl-18-(3-(5-methyl-1,2,4-oxadiazol-3-yl)benzamido)-4-oxo-3,8,11-trioxa-5,14-diazaicosan-20-amido)thiazole-5-carboxylate (30.0 mg, 0.0394 mmol) was dissolved in dry 1-propanol (0.30 mL). 4 N HCl in dioxane (148 μL, 0.5914 mmol) was added ,and the reaction mixture was stirred at rt for 24 h. Another 10 equiv of 4 N HCl in dioxane was added, and the reaction mixture was stirred at rt for an additional 16 h. The solution was purified using a 1 g SCX-2 column, and the compound was released with a solution of 2 N NH_3_ in MeOH. The solvent was removed in vacuo, and the residue was purified via RP column chromatography (30–100% MeOH/H_2_O + 0.1% formic acid). The desired fractions were filtered through an 1 g SCX-2 column (the compound was released with a solution of 2 N NH_3_ in MeOH) to give **88** (11 mg, 42%) as a white powder. ^1^H NMR (600 MHz, methanol-*d*_4_) δ 8.48 (d, *J* = 1.7 Hz, 1H), 8.21 (dt, *J* = 7.8, 1.4 Hz, 1H), 7.98 (dt, *J* = 7.8, 1.5 Hz, 1H), 7.63 (t, *J* = 7.8 Hz, 1H), 4.58 (p, *J* = 6.8 Hz, 1H), 4.25–4.18 (m, 2H), 3.62 (p, *J* = 4.6 Hz, 6H), 3.56 (t, *J* = 5.2 Hz, 2H), 2.87 (t, *J* = 5.3 Hz, 2H), 2.85–2.79 (m, 2H), 2.68 (d, *J* = 1.1 Hz, 3H), 2.64 (t, *J* = 5.7 Hz, 2H), 2.58 (d, *J* = 1.1 Hz, 3H), 2.56–2.46 (m, 2H), 2.30 (s, 3H), 1.75 (ddd, *J* = 10.3, 7.4, 4.9 Hz, 4H), 1.66 (dq, *J* = 15.9, 7.8 Hz, 2H), 1.02 (td, *J* = 7.4, 1.1 Hz, 3H). HPLC/HRMS (ESI): *m*/*z* calculated for C_31_H_46_N_7_O_7_S^+^ [M + H]^+^ 660.3174, found 660.3181. *R*_t_ (4 min): 2.34 min.

##### Propyl 2-((*S*)-1-(((*S*,*E*)-Cyclooct-4-en-1-yl)oxy)-11-methyl-15-(3-(5-methyl-1,2,4-oxadiazol-3-yl)benzamido)-1-oxo-5,8-dioxa-2,11-diazaheptadecan-17-amido)-4-methylthiazole-5-carboxylate (**39**)

Propyl (*S*)-2-(6-((2-(2-(2-aminoethoxy)ethoxy)ethyl)(methyl)amino)-3-(3-(5-methyl-1,2,4-oxadiazol-3-yl)benzamido)hexanamido)-4-methylthiazole-5-carboxylate **88** (4.80 mg, 0.0073 mmol) and [(1*R*,4*E*)-cyclooct-4-en-1-yl] (2,5-dioxopyrrolidin-1-yl) carbonate (3.10 mg, 0.0116 mmol) were charged in a HPLC vial and dissolved in dry DMF (0.20 mL). DIPEA (1.90 μL, 0.0109 mmol) was added, and the reaction mixture was stirred at rt overnight. The reaction mixture was directly purified by semiprep. HPLC to yield **39** (4 mg, 68%) as a dark purple powder. ^1^H NMR (500 MHz, MeOD) δ 8.54 (s, 1H), 8.48 (t, *J* = 1.7 Hz, 1H), 8.20 (d, *J* = 7.8 Hz, 1H), 8.03–7.93 (m, 1H), 7.63 (t, *J* = 7.8 Hz, 1H), 5.66–5.37 (m, 2H), 4.60 (d, *J* = 7.7 Hz, 1H), 4.27 (s, 1H), 4.20 (t, *J* = 6.5 Hz, 2H), 3.80–3.67 (m, 2H), 3.67–3.55 (m, 4H), 3.48 (t, *J* = 5.5 Hz, 2H), 3.23 (t, *J* = 5.6 Hz, 2H), 3.07 (d, *J* = 5.3 Hz, 2H), 2.94 (s, 1H), 2.85 (d, *J* = 6.7 Hz, 2H), 2.67 (s, 3H), 2.66 (s, 3H), 2.57 (s, 3H), 2.28 (d, *J* = 21.0 Hz, 3H), 1.99–1.53 (m, 14H), 1.01 (t, *J* = 7.4 Hz, 3H). ^13^C NMR (151 MHz, MeOD) δ 179.10, 171.31, 169.34, 168.98, 164.30, 161.24, 157.81, 136.68, 136.05, 133.75, 131.22, 131.06, 130.41, 128.64, 127.28, 116.45, 81.78, 71.37, 71.24, 70.99, 67.48, 67.38, 57.61, 56.99, 48.14, 42.21, 42.04, 41.92, 41.52, 39.63, 35.14, 33.48, 32.76, 32.12, 29.98, 28.42, 26.25, 23.17, 23.03, 17.32, 12.12, 10.82. (Rotameric forms present, 1 X C_Ar_ not observed) HPLC/HRMS (ESI): *m*/*z* calculated for C_40_H_58_N_7_O_9_S^+^ [M + H]^+^ 812.4011, found 812.4007. *R*_t_ (4 min): 2.94 min.

##### *tert*-Butyl (4-Chlorobut-2-yn-1-yl)carbamate (**90**)

4-Chlorobut-2-yn-1-amine hydrochloride (500 mg, 3.21 mmol) was dissolved in DCM (10.20 mL). To the mixture was added di-*tert*-butyl dicarbonate (1.05 g, 4.82 mmol), followed by triethylamine (1.12 mL, 8.0351 mmol). The reaction mixture was stirred at rt for 2 h. Water was added to the reaction mixture, and the product was extracted with DCM, dried over MgSO_4_ and concentrated in vacuo. The residue was purified using NP column chromatography (10–30% EtOAc/cyclohexanes) to give **90** (440 mg, 67%) as a colorless oil. ^1^H NMR (500 MHz, CDCl_3_) δ 4.73 (broad s, 1H), 4.13 (t, *J* = 2.1 Hz, 2H), 4.02–3.90 (broad s, 2H), 1.44 (s, 9H).

##### Propyl (*S*)-2-(6-((4-Aminobut-2-yn-1-yl)(methyl)amino)-3-(3-(5-methyl-1,2,4-oxadiazol-3-yl)benzamido)hexanamido)-4-methylthiazole-5-carboxylate (**91**)

Propyl 4-methyl-2-[[(3S)-6-(methylamino)-3-[[3-(5-methyl-1,2,4-oxadiazol-3-yl)benzoyl]amino]hexanoyl]amino]thiazole-5-carboxylate hydrochloride **90** (95.0 mg, 0.168 mmol) was dissolved in dry DMF (1.12 mL). To the mixture was added Et_3_N (71 μL, 0.504 mmol), followed by *tert*-butyl (4-chlorobut-2-yn-1-yl)carbamate (41.1 mg, 0.202 mmol) in dry DMF (0.1 mL). The reaction mixture was stirred at rt for 24 h. The reaction mixture was purified via RP column chromatography (30–100% MeOH/H_2_O + 0.1% formic acid) to give propyl (*S*)-2-(6-((4-((*tert*-butoxycarbonyl)amino)but-2-yn-1-yl)(methyl)amino)-3-(3-(5-methyl-1,2,4-oxadiazol-3-yl)benzamido)hexanamido)-4-methylthiazole-5-carboxylate (54 mg, 46%) as a white powder. HPLC/MS: *m*/*z* 696.3069 [M + H]^+^. *R*_t_ (4 min): 2.89 min.

This propyl (*S*)-2-(6-((4-((*tert*-butoxycarbonyl)amino)but-2-yn-1-yl)(methyl)amino)-3-(3-(5-methyl-1,2,4-oxadiazol-3-yl)benzamido)hexanamido)-4-methylthiazole-5-carboxylate (48.00 mg, 0.0690 mmol) was dissolved in dry 1-propanol (0.50 mL). 4 N HCl in dioxane (0.26 mL, 1.0347 mmol) was added, and the reaction mixture was stirred at rt for 6 h. The solvent was removed in vacuo, and the residue was purified via RP column chromatography (30–100% MeOH/H_2_O + 0.1% formic acid). The desired fractions were filtered through a 1 g SCX-2 column (the compound was released with a solution of 2 N NH_3_ in MeOH) to give propyl (*S*)-2-(6-((4-aminobut-2-yn-1-yl)(methyl)amino)-3-(3-(5-methyl-1,2,4-oxadiazol-3-yl)benzamido)hexanamido)-4-methylthiazole-5-carboxylate (18 mg, 44%) as a white powder. ^1^H NMR (600 MHz, methanol-*d*_4_) δ 8.47 (t, *J* = 1.8 Hz, 1H), 8.20 (dt, *J* = 7.8, 1.4 Hz, 1H), 7.96 (dt, *J* = 7.9, 1.4 Hz, 1H), 7.63 (t, *J* = 7.8 Hz, 1H), 4.60 (p, *J* = 6.9 Hz, 1H), 4.21 (t, *J* = 6.6 Hz, 2H), 3.40 (t, *J* = 2.0 Hz, 2H), 3.35 (t, *J* = 2.1 Hz, 2H), 2.87–2.79 (m, 2H), 2.68 (s, 3H), 2.58 (s, 3H), 2.57–2.51 (m, 2H), 2.31 (s, 3H), 1.76 (dq, *J* = 9.7, 7.3 Hz, 4H), 1.65 (dq, *J* = 12.3, 7.7 Hz, 2H), 1.02 (t, *J* = 7.4 Hz, 3H). HPLC/HRMS (ESI): *m*/*z* calculated for C_29_H_38_N_7_O_5_S^+^ [M + H]^+^ 596.2650, found 596.2656. *R*_t_ (4 min): 2.35 min.

##### propyl 2-((*S*)-6-((4-(((((*S*,*E*)-Cyclooct-4-en-1-yl)oxy)carbonyl)amino)but-2-yn-1-yl)(methyl)amino)-3-(3-(5-methyl-1,2,4-oxadiazol-3-yl)benzamido)hexanamido)-4-methylthiazole-5-carboxylate (**40**)

Propyl (*S*)-2-(6-((4-aminobut-2-yn-1-yl)(methyl)amino)-3-(3-(5-methyl-1,2,4-oxadiazol-3-yl)benzamido)hexanamido)-4-methylthiazole-5-carboxylate **91** (5.0 mg, 0.0084 mmol) and [(1*R*,4*E*)-cyclooct-4-en-1-yl] (2,5-dioxopyrrolidin-1-yl) carbonate **89** (3.6 mg, 0.0134 mmol) were charged in a vial and dissolved in dry DMF (0.20 mL). DIPEA (2.19 μL, 0.0126 mmol) was added, and the reaction mixture was stirred at rt overnight. Purification by prep. HPLC gave **40** (6 mg, 96%) as a white powder. ^1^H NMR (600 MHz, MeOD) δ 8.49 (d, *J* = 1.8 Hz, 1H), 8.38 (s, 1H), 8.21 (dt, *J* = 7.8, 1.4 Hz, 1H), 7.98 (dt, *J* = 7.8, 1.5 Hz, 1H), 7.64 (t, *J* = 7.8 Hz, 1H), 5.58 (ddd, *J* = 15.2, 10.2, 4.5 Hz, 1H), 5.46 (ddd, *J* = 15.8, 11.1, 3.6 Hz, 1H), 4.62 (pent, *J* = 6.7 Hz, 1H), 4.31 (s, 1H), 4.21 (t, *J* = 6.5 Hz, 2H), 3.89 (s, 2H), 3.74–3.68 (m, 2H), 3.00–2.82 (m, 4H), 2.68 (s, 3H), 2.61 (s, 3H), 2.58 (s, 3H), 2.36–2.29 (m, 2H), 2.00–1.86 (m, 4H), 1.85–1.54 (m, 10H), 1.02 (t, *J* = 7.4 Hz, 3H). ^13^C NMR (151 MHz, MeOD) δ 177.65, 169.88, 167.94, 167.56, 162.88, 159.81, 156.87, 156.39, 135.29, 134.64, 132.34, 129.76, 129.64, 128.96, 127.21, 125.88, 115.04, 84.84, 80.73, 73.05, 66.05, 54.69, 46.76, 44.90, 40.75, 40.54, 39.91, 38.22, 33.71, 32.05, 31.31, 30.70, 29.74, 22.16, 21.76, 15.91, 10.70, 9.41. HPLC/HRMS (ESI): *m*/*z* calculated for C_38_H_50_N_7_O_7_S^+^ [M + H]^+^ 748.3487, found 748.3491. *R*_t_ (4 min): 2.92 min.

## Supporting Information Available

SMILES strings for compounds **2**–**40** and associated assay data (CSV)
Enantiomeric ratio determination of compounds **82** and **83**; test reactions between TCO probes and a commercially available tetrazine; multipolarity spindle assay concentration responses; optimization of washing experiments for fluorescent imaging; fluorescent imaging target engagement assay supplementary images; ^1^H and ^13^C NMR spectra for test compounds **2**, **4**–**7**, **9**–**36**, and **38**–**40**; and HPLC traces for test compounds **2**, **4**–**7**, and **9**–**40** (PDF)

## Abbreviations Used

4N: noncentrosome amplified DLD1 cell line
4NCA: tetraploid centrosome-amplified DLD1 cell line
ADP: adenosine diphosphate
Alexa-488: (2-(3-amino-6-imino-4,5-disulfoxanthen-9-yl)-5-[5-(2,5-dioxopyrrol-1-yl)pentylcarbamoyl]benzoic acid)
ATP: adenosine 5′-triphosphate
DAPI: 4′,6-diamidino-2-phenylindole
Cy5: (2*Z*)-2-[(2*E*,4*E*)-5-[1-(5-carboxypentyl)-3,3-dimethyl-5-sulfoindol-1-ium-2-yl]penta-2,4-dienylidene]-1-ethyl-3,3-dimethylindole-5-sulfonate
DCM: dichloromethane
DIPEA: *N*,*N*-diisopropylethylamine
DMF: dimethylformamide
DMSO: dimethyl sulfoxide
EDC: 1-ethyl-3-(3-(dimethylamino)propyl)carbodiimide
ESI: electrospray ionization
HATU: 1-[bis(dimethylamino)methylene]-1*H*-1,2,3-triazolo[4,5-*b*]pyridinium 3-oxide hexafluorophosphate
HBA: hydrogen-bond acceptor
HBD: hydrogen-bond donor
HCl: hydrochloric acid
HOBt: hydroxybenzotriazole
HSET: human spleen, embryo, and testes protein
IEDDA: inverse electron demand Diels–Alders
*K*_Sol_: aqueous kinetic solubility
LC-MS: liquid chromatography–mass spectrometry
LE: ligand efficiency
LLE: lipophilic ligand efficiency
MBD: microtubule binding domain
MT: microtubules
MTOCs: microtubule organizing centers
NP: normal phase
RP: reverse phase
rt: room (ambient) temperature
SAR: structure–activity relationship
TCO: *trans*-cyclooctene
TFA: trifluoroacetic acid
