# Supplementary material for: Discovery of 2-(3-Benzamidopropanamido)thiazole-5-carboxylate Inhibitors of the Kinesin HSET (KIFC1) and the Development of Cellular Target Engagement Probes
Source: J Med Chem. 2023 Feb 7;66(4):2622–45. doi: 10.1021/acs.jmedchem.2c01591 (PMC9969401; doi:10.1021/acs.jmedchem.2c01591)

Supporting information

Discovery of 2-(3-benzamidopropanamido)thiazole-5-carboxylate inhibitors of the kinesin HSET (KIFC1) and the development of cellular target engagement probes

François Saint-Dizier[1,‡], Thomas P. Matthews[1,‡,*], Aaron M. Gregson[1], Hugues Prevet[1], Tatiana McHardy[1], Giampiero Colombano[1], Harry Saville[1], Martin Rowlands[1], Caroline Ewens[1], P. Craig McAndrew[1], Kathy Tomlin[1], Delphine Guillotin[1], Grace Wing-Yan Mak[1], Konstantinos Drosopoulos[3], Ioannis Poursaitidis[1], Rosemary Burke[1], Rob van Montfort[1,2], Spiros Linardopoulos[1,3,†], and Ian Collins[1,*]

1. Centre for Cancer Drug Discovery, Division of Cancer Therapeutics, The Institute of Cancer Research, London, SW7 3RP, UK
2. Division of Structural Biology, The Institute of Cancer Research, London SW7 3RP, UK
3. Breast Cancer Now Research Centre at The Institute of Cancer Research, London SW7 3RP, UK

* Corresponding authors. Email: (T.P.M.) thomas.matthews@icr.ac.uk, (I.C.) ian.collins02@icr.ac.uk

Table of Contents

1. Enantiomeric ratio determination of compounds 82 and 83 .... S2
2. Test reactions between TCO probes and a commercially available tetrazine.......... S3
3. Multipolarity Spindle Assay concentration responses S6
4. Optimisation of washing experiments for fluorescent imaging.......... S7
5. Fluorescent imaging target engagement assay supplementary images S8
6. Copies of ^{1}H and ^{13}C NMR spectra for test compounds **2**, **4-7**, **9-36**, **38-40**. S9
7. Copies of HPLC traces for test compounds **2**, **4-7**, **9-40** S44

1. Enantiomeric ratio determination of compounds 82 and 83

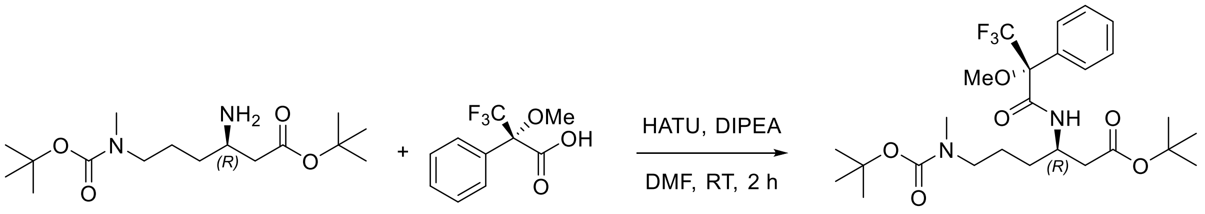

Tert-butyl (3R)-3-amino-6-[*tert*-butoxycarbonyl(methyl)amino]hexanoate **82** (15 mg, 0.047 mmol, 1 eq), HATU (20 mg, 0.052 mmol, 1.1 eq) and (-)-methoxy-trifluoromethylphenylacetic acid (12 mg, 0.052, 1.1 eq) were dissolved in dry DMF (0.32 mL). DIPEA (16 µL, 0.10 mmol, 2 eq) was added and the reaction mixture was stirred at rt for 2 h. The reaction mixture was diluted with EtOAc, washed with water, $NaHCO_3$, dried over $MgSO_4$ and concentrated *in vacuo* to give a yellow oil.

NMR determination of the crude sample showed an enantiomeric ratio of 95:5 (the integration of the methoxy group from the Mosher's amide was used to determine the enantiomeric ratio)

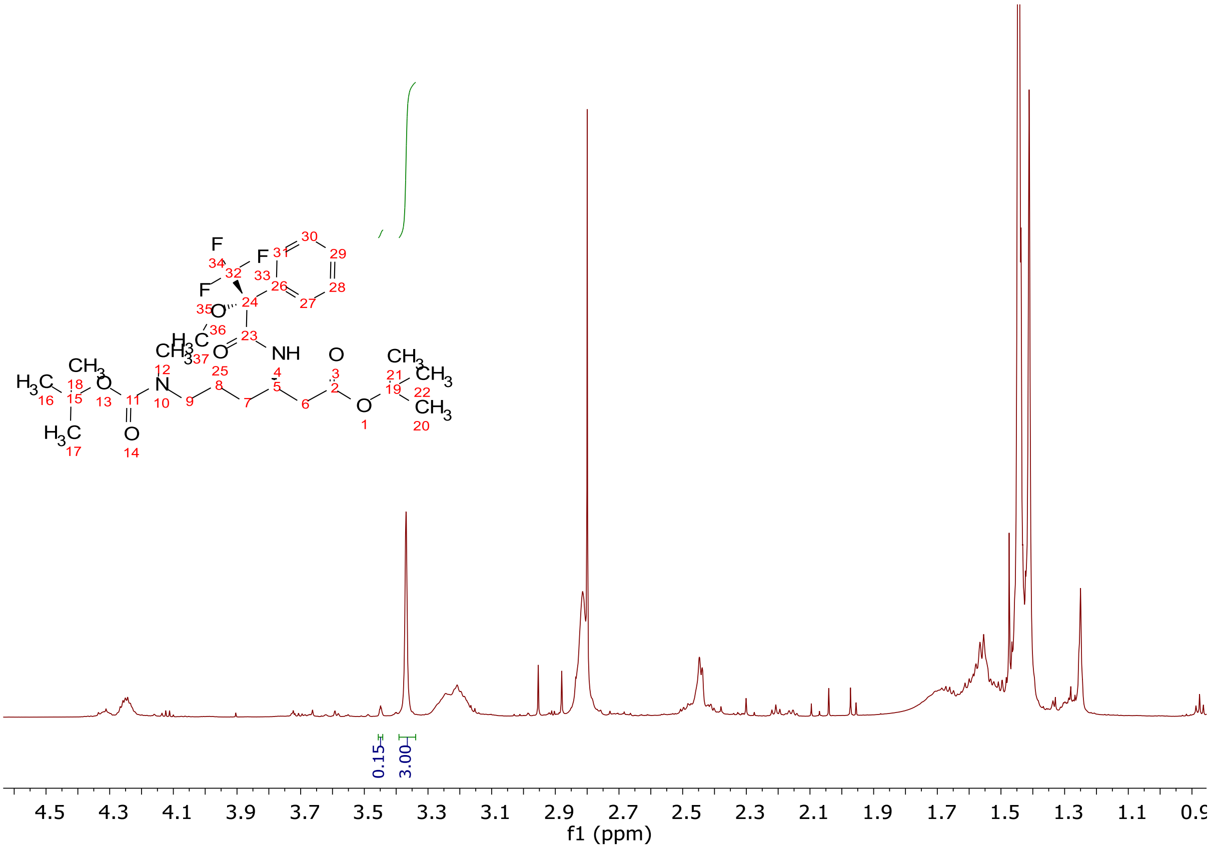

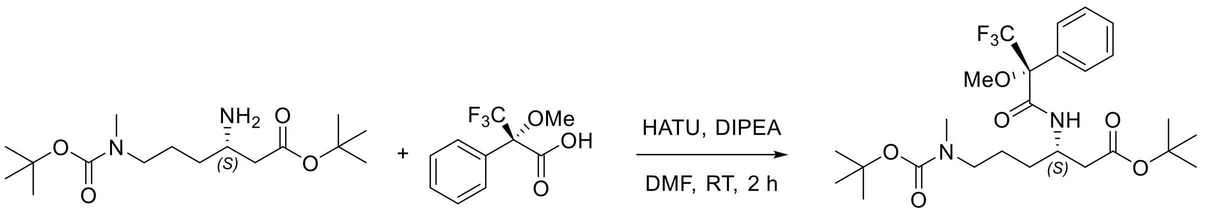

Tert-butyl (3S)-3-amino-6-[tert-butoxycarbonyl(methyl)amino]hexanoate **83** (16 mg, 0.051 mmol, 1 eq), HATU (21 mg, 0.055 mmol, 1.1 eq) and (-)-methoxy-trifluoromethylphenylacetic acid (13 mg, 0.055, 1.1 eq) were dissolved in dry DMF (0.32 mL). DIPEA (17 µL, 0.10 mmol, 2 eq) was added and the reaction mixture was stirred at rt for 2 h. The reaction mixture was diluted with EtOAc, washed with water, $NaHCO_3$, dried over $MgSO_4$ and concentrated *in vacuo* to give a yellow oil.

NMR determination of the crude sample showed an enantiomeric ratio of 97:3 (the integration of the methoxy group from the Mosher's amide was used to determine the enantiomeric ratio)

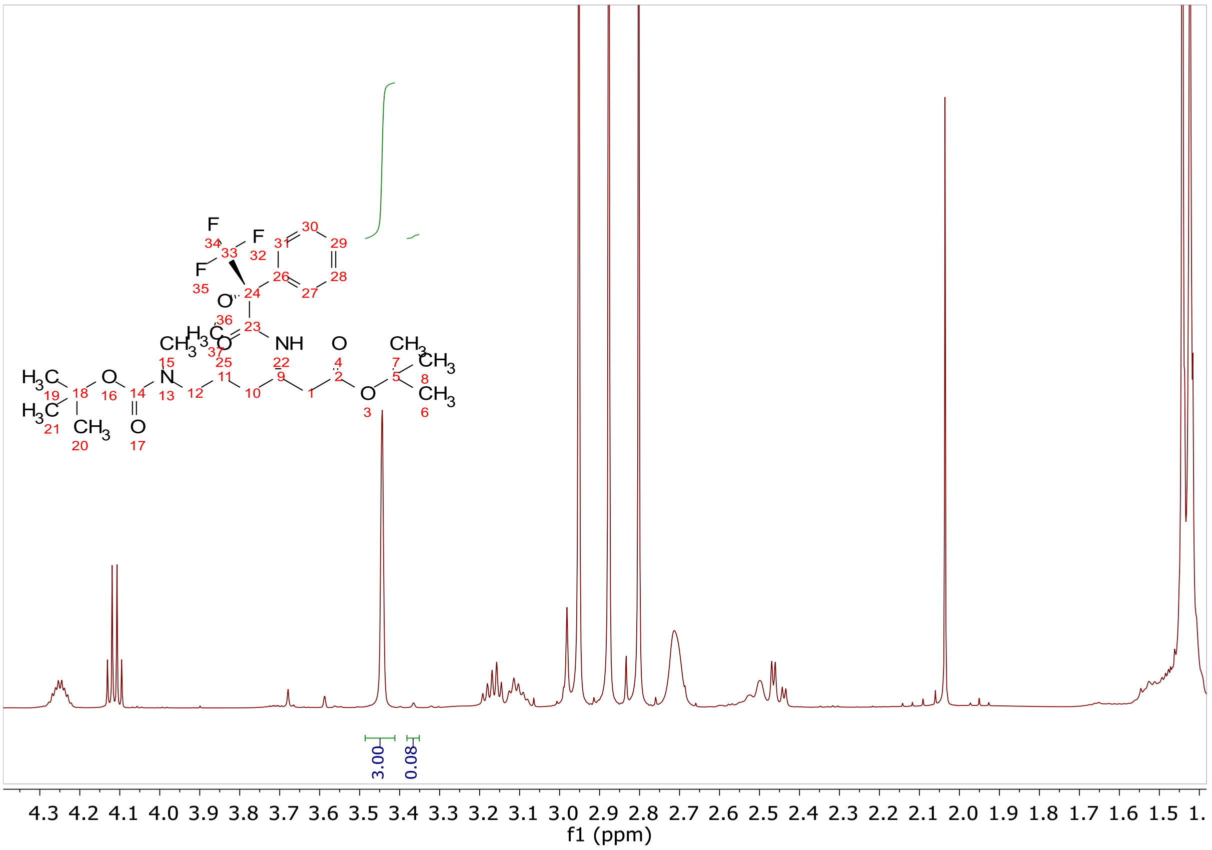

2. Test reactions between TCO probes and a commercially available tetrazine

LCMS profiles of the reaction between TCO probes **38** , **39**, **40** with [4-(1,2,4,5-tetrazin-3-yl)phenyl]methanamine hydrochloride. All three experiments were performed with 5 µL of a 10 mM solution of TCO probe and 10 µL of a 10 mM solution of the tetrazine in 200 µL of THF. LCMS measured after 5 min.

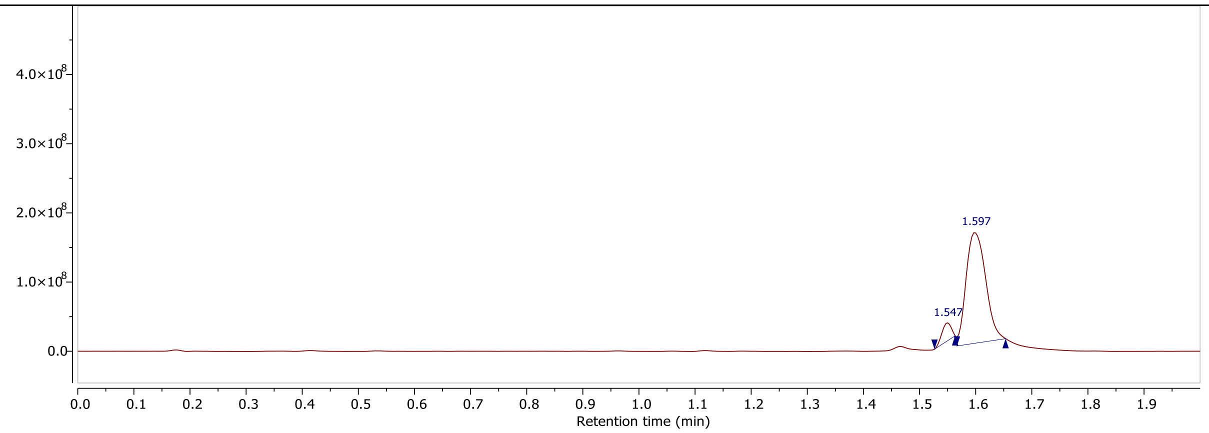

HPLC trace of **38** alone:

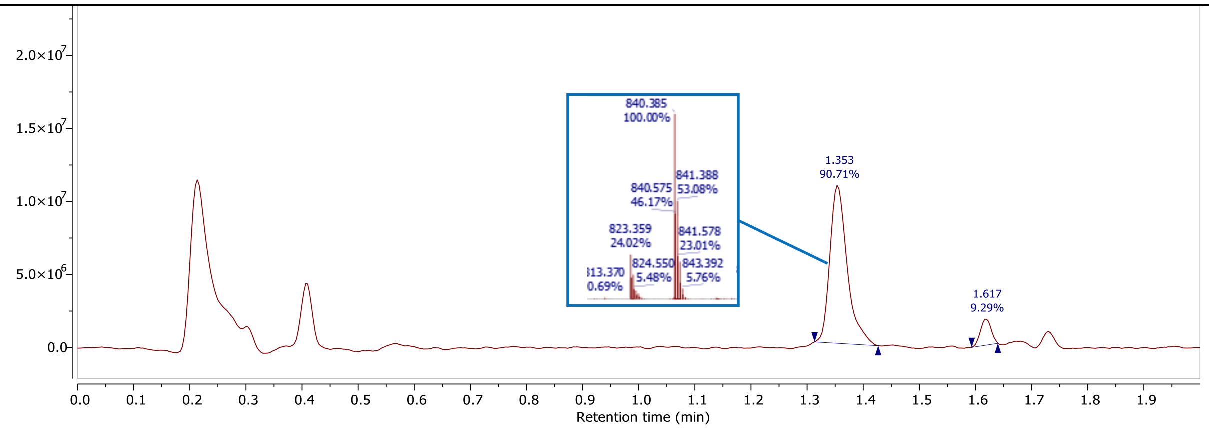

HPLC trace of **38** + benzylamino tetrazine after 5 min: inset, MS analysis of peak

Figure S1: Reaction between **38** and [4-(1,2,4,5-tetrazin-3-yl)phenyl]methanamine hydrochloride (93% conversion – 7% starting material remaining)

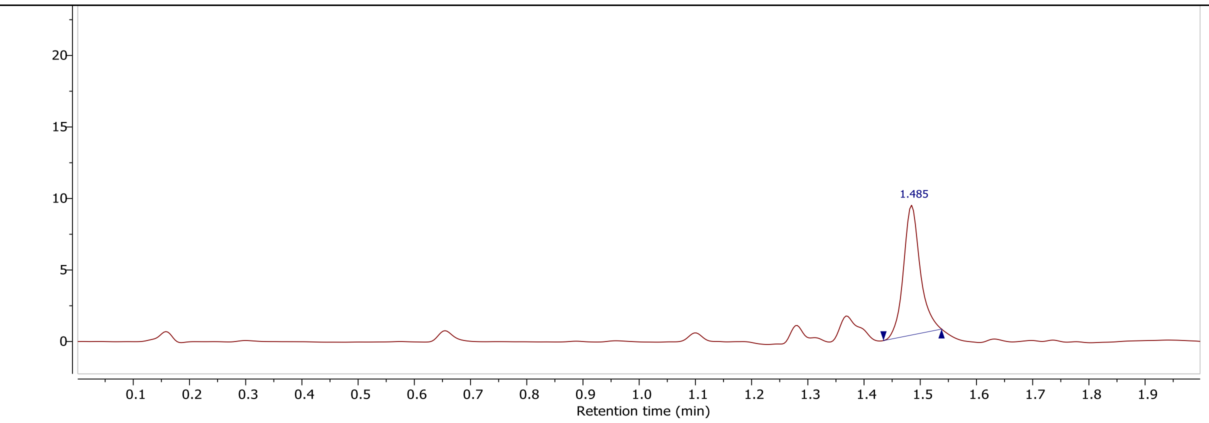

HPLC trace of **39** alone:

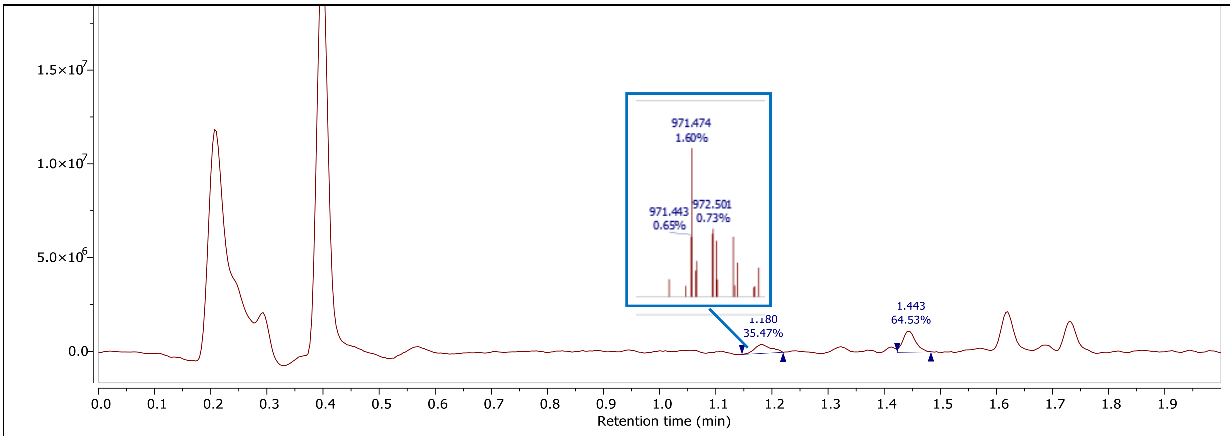

HPLC trace of **39** + benzylamino tetrazine after 5 min: inset, MS analysis of peak

Figure S2: Reaction between **39** and tetrazine (35% conversion – 65% starting material remaining)

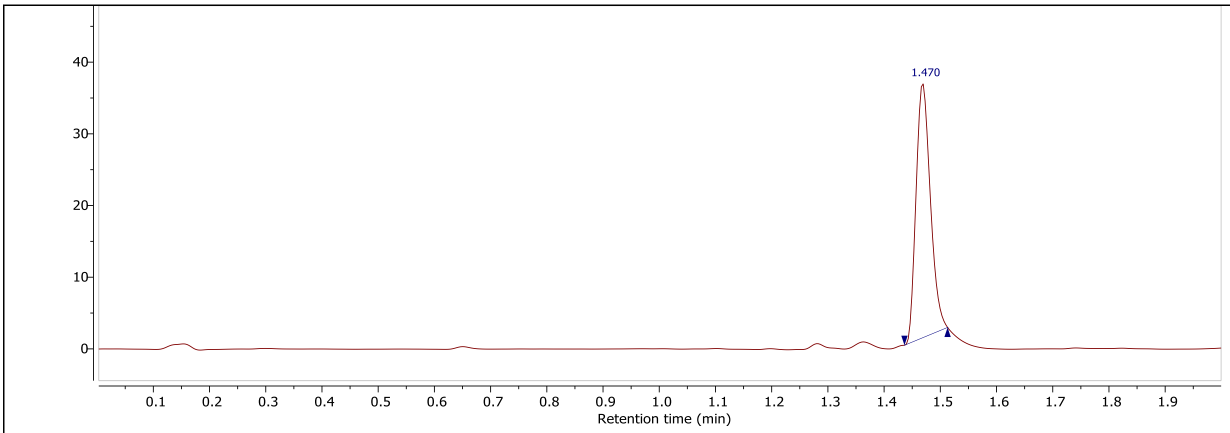

HPLC trace of **40** alone:

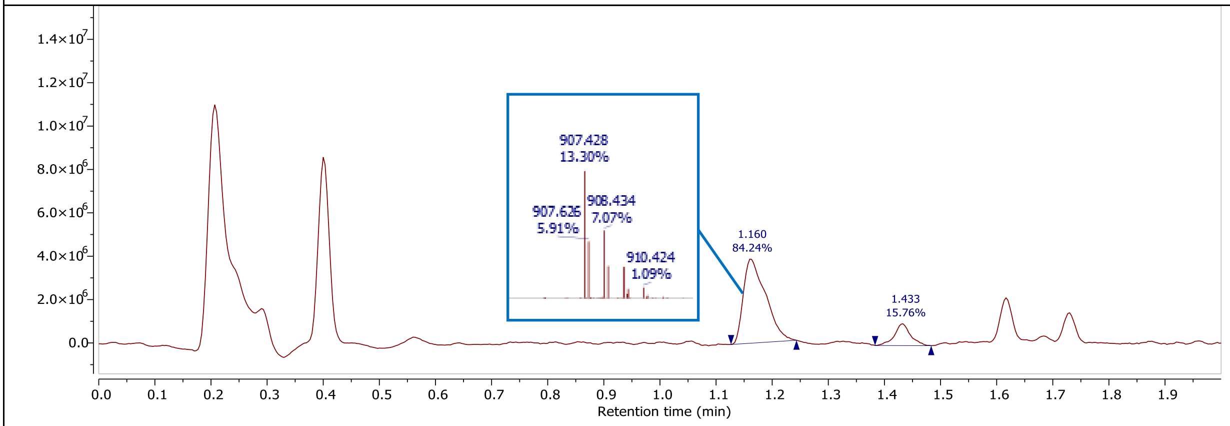

HPLC trace of **40** + benzylamino tetrazine after 5 min: inset, MS analysis of peak

Figure S3: Reaction between **40** and [4-(1,2,4,5-tetrazin-3-yl)phenyl]methanamine hydrochloride (84% conversion – 16% starting material remaining)

3. Multipolarity Spindle Assay concentration responses

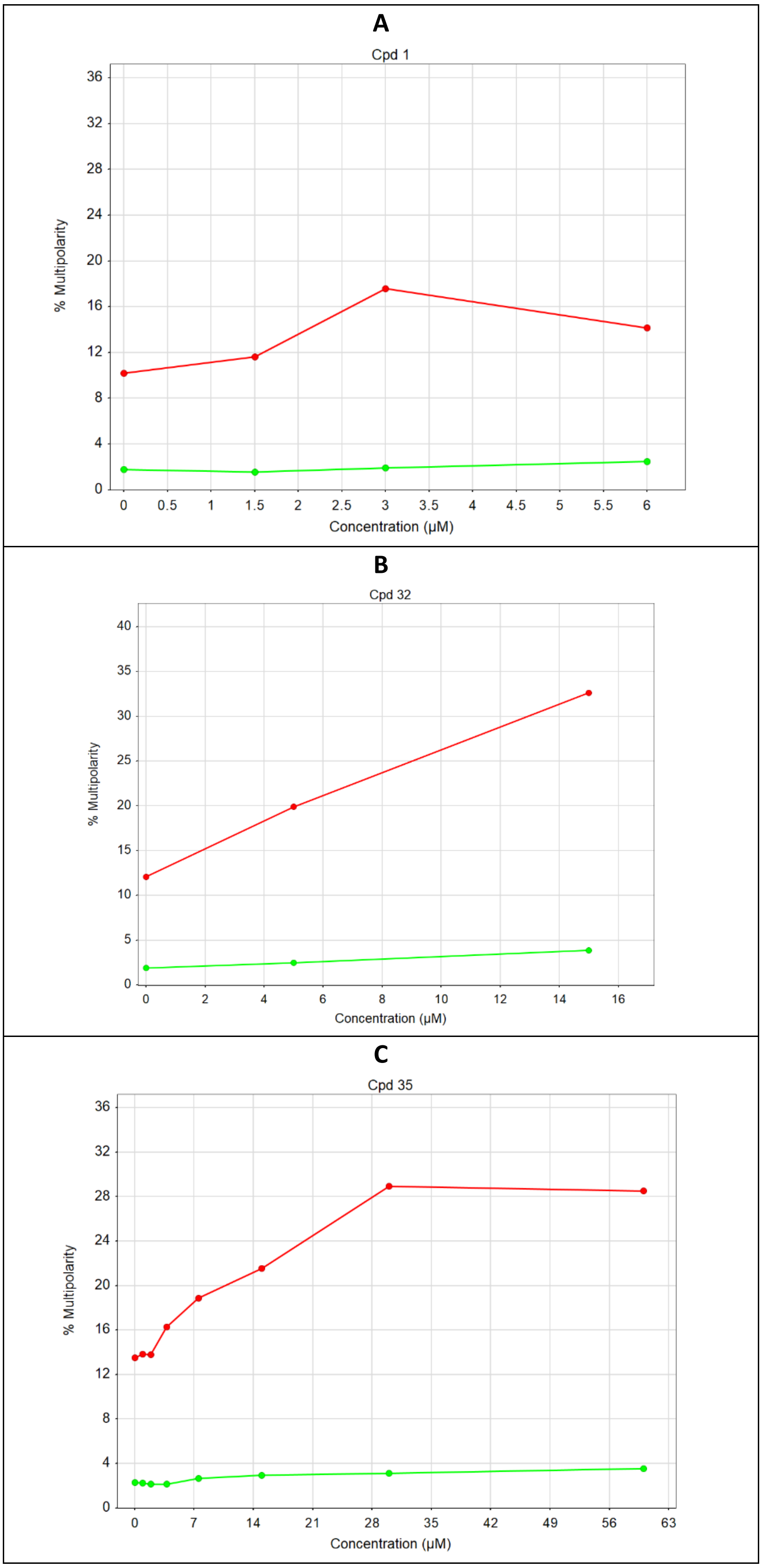

Figure S4: Graphs showing concentration responses in Multipolar Spindle Assay. Red lines= 4NCA DLD1 cell line, Green lines = 4N DLD1 cell line. Panel A: Compound **1** (AZ82). Cellular toxicity observed at 6 µM so extending to higher concentrations was not viable. Panel B: Compound **32**. Cellular toxicity observed at > 15 µM so extending to higher concentrations was not viable. Panel C: Compound **35**. Cellular toxicity observed at 60 µM.

4. Optimisation of washing experiments for fluorescent imaging

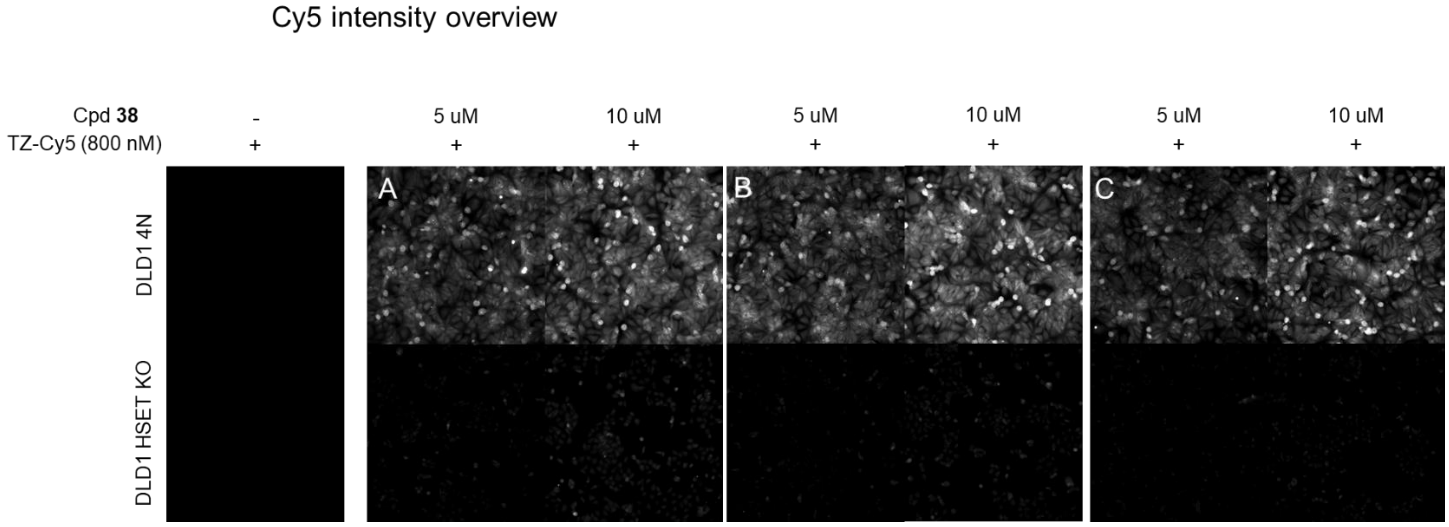

Average Pixel intensity								
		A		B		C	
Cpd **38**	-	5µM	10µM	5µM	10µM	5µM	10µM
Tz-Cy5 (800nM)	+	+	+	+	+	+	+
DLD1 4N	342	5372	7167	6057	7628	4723	6337
DLD1 HSET KO	227	799	1464	752	1121	702	803

Figure S5. Optimisation of washing conditions using **38** to improve fluorescence signal to noise ratio in DLD1 4N and DLD1 HSET knockout cell lines at two different concentrations. **Panel A:** 3 quick washes at room temperature. **Panel B:** 2 quick washes at room temperature followed by 1 at 37 °C for 5 minutes. **Panel C:** 2 quick washes at room temperature followed by 1 at 37 °C for 10 minutes.

5. Fluorescent imaging target engagement assay supplementary images

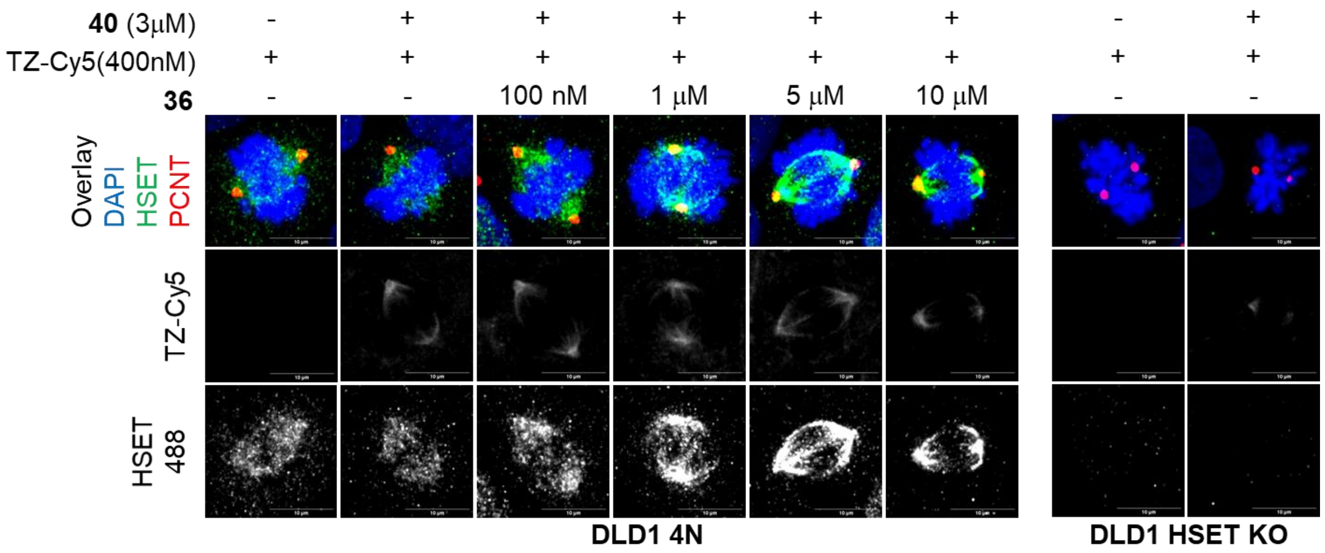

Figure S6. Images showing distribution of Cy5 induced fluorescence from Tetrazine-Cy5 (Txz-Cy5) with and without the addition of TCO probe **40** compared to the HSET intensities identified by indirect immunofluorescence using an Alexa-488 fluorophore in DLD1 4N and DLD1 HSET KO cell lines. The overlay shows the mitotic pole areas identified by staining for Pericentrin (Red), HSET intensity as measured by indirect immunofluorescence using an Alexa-488 fluorophore (Green) and the nucleus stained with DAPI (Blue). Also, images show the effects on the fluorescence of increasing the concentration of **36** which competed out the TCO probe **40**.

6. Copies of ^{1}H and ^{13}C NMR spectra for test compounds **2**, **4-7**, **9-36**, **38-40**.

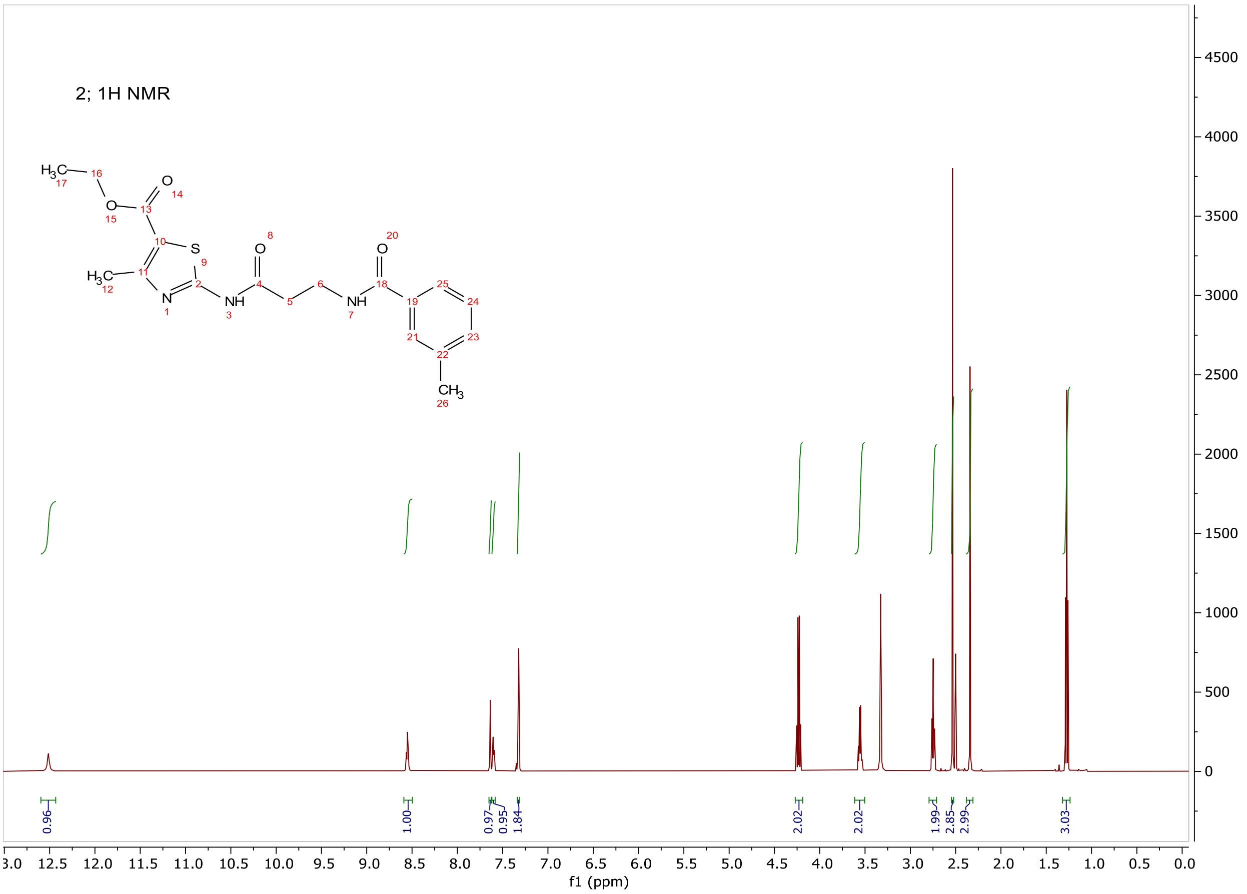

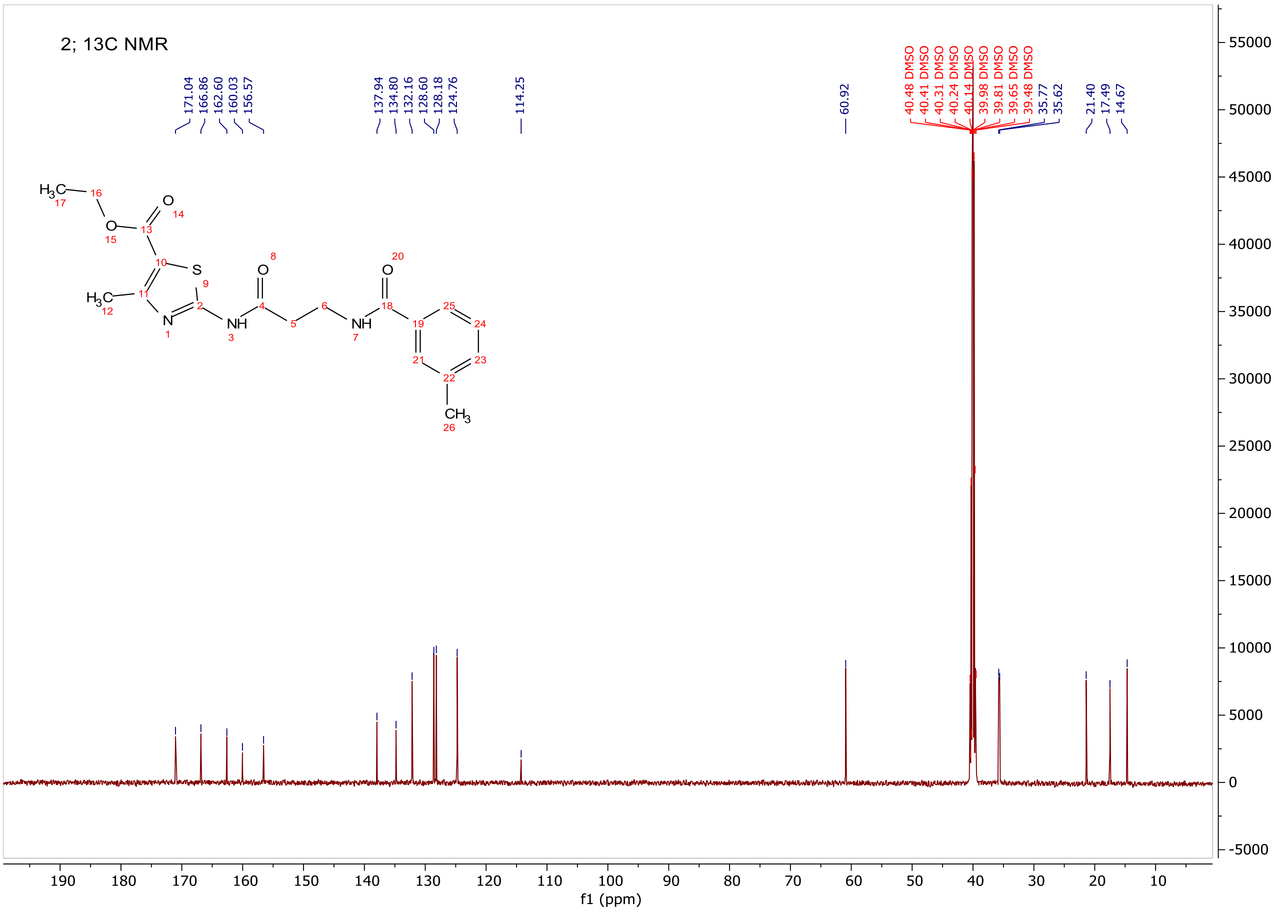

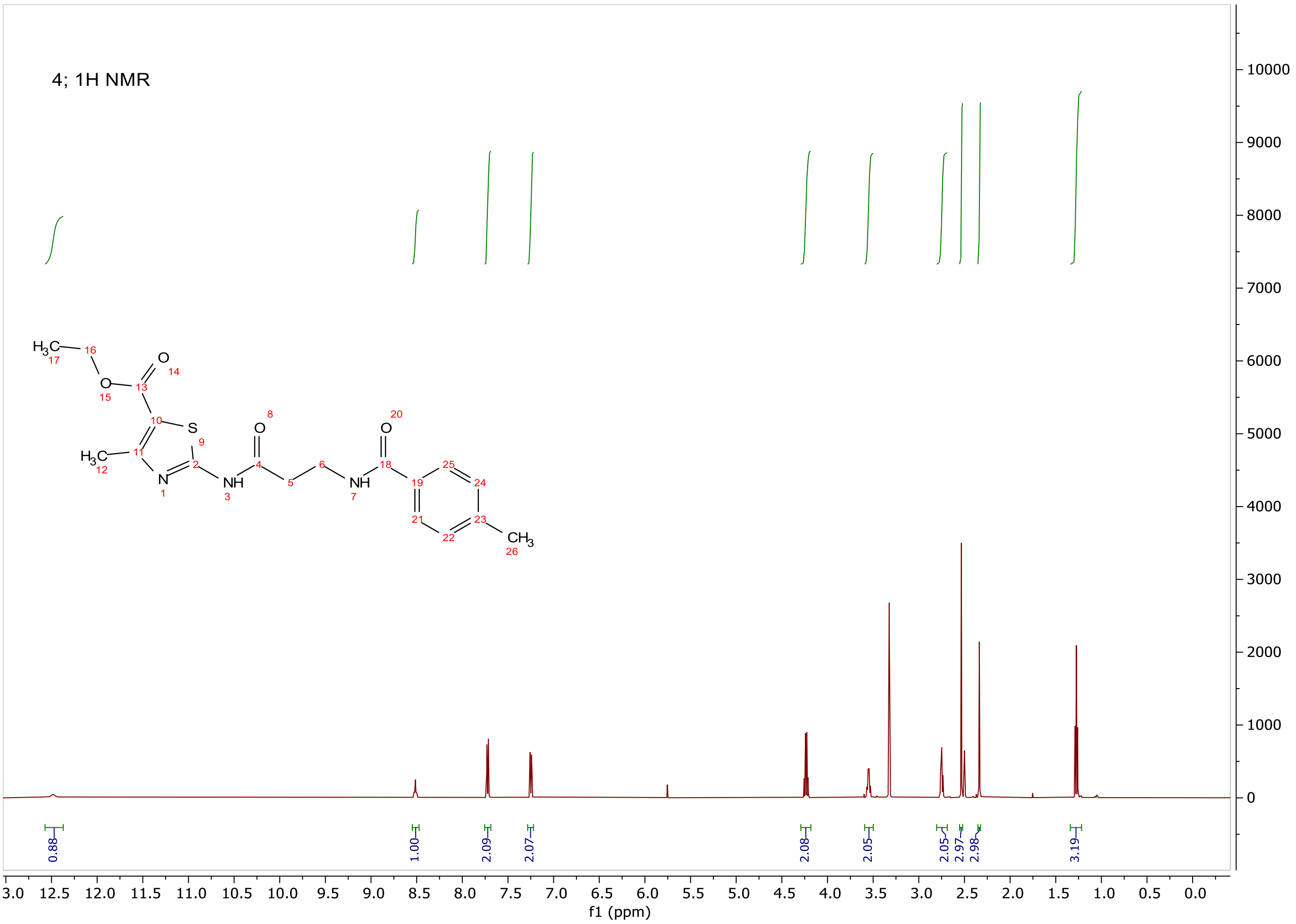
4; 1H NMR
0.88
1.00
2.09
2.07
2.08
2.05
2.05
2.97
2.98
3.19
f1 (ppm)

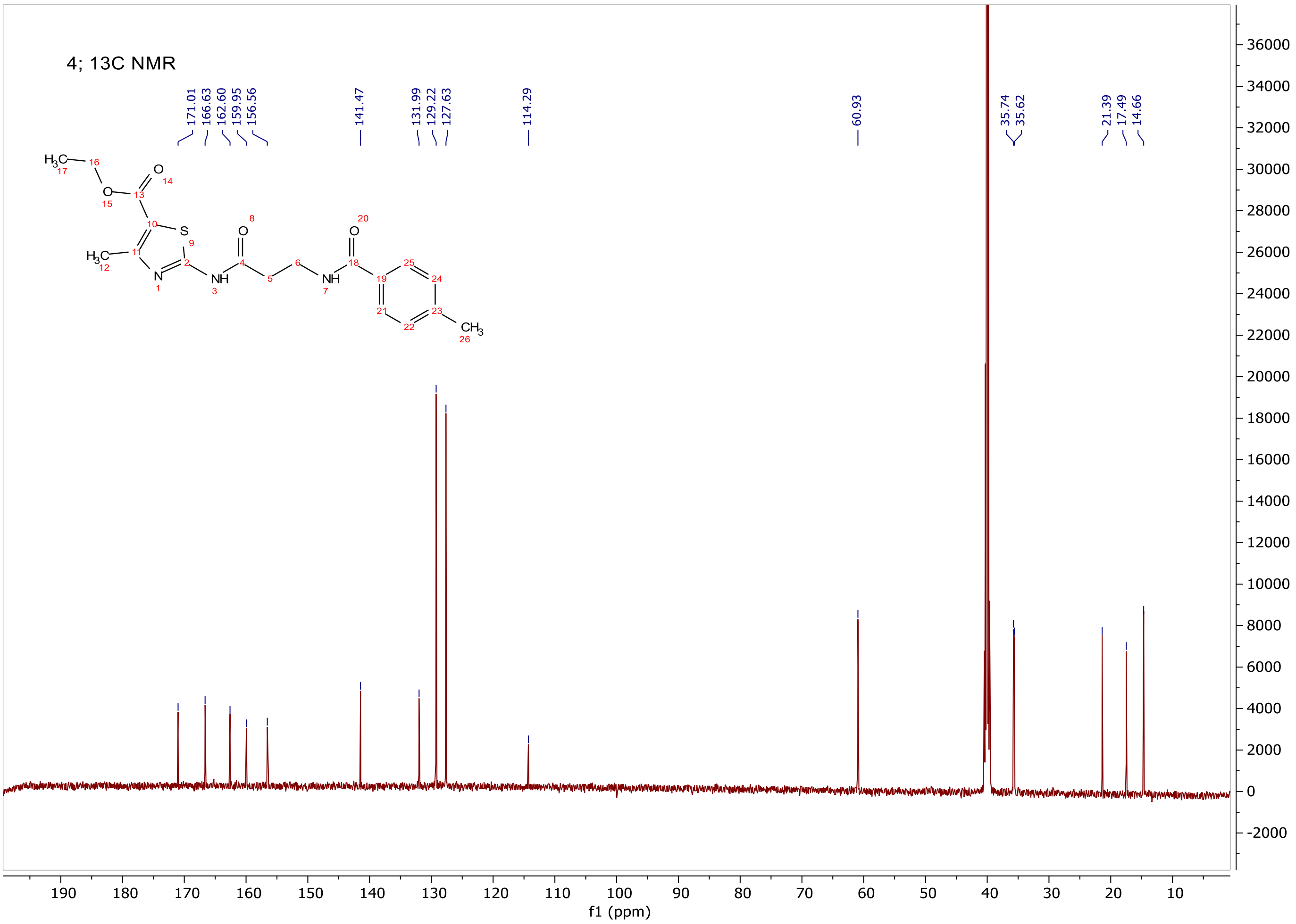
4; 13C NMR
171.01
166.63
162.60
159.95
156.56
141.47
131.99
129.22
127.63
114.29
60.93
35.74
35.62
21.39
17.49
14.66
f1 (ppm)

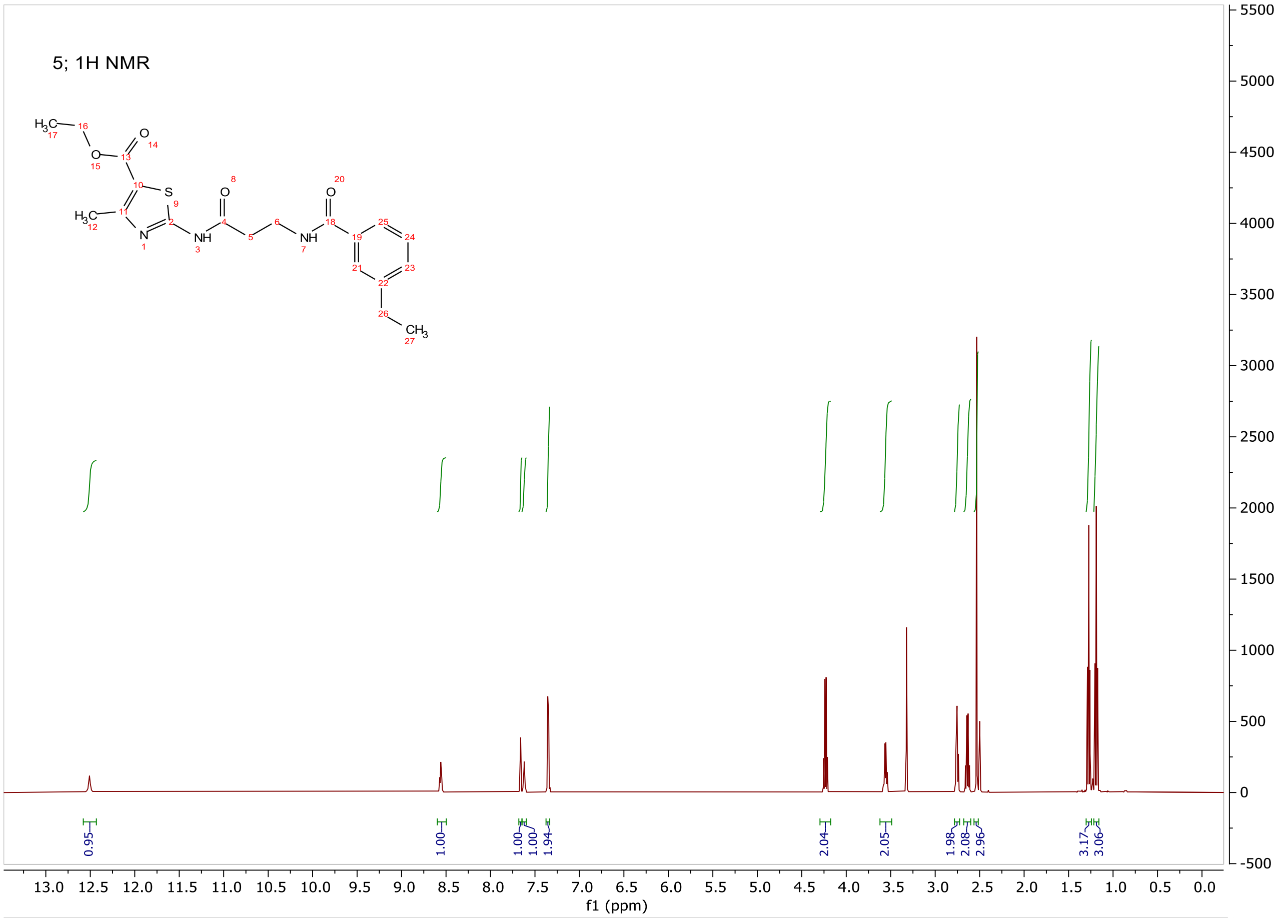
5; 1H NMR
0.95
1.00
1.00
1.00
1.94
2.04
2.05
1.98
2.08
2.96
3.17
3.06
f1 (ppm)

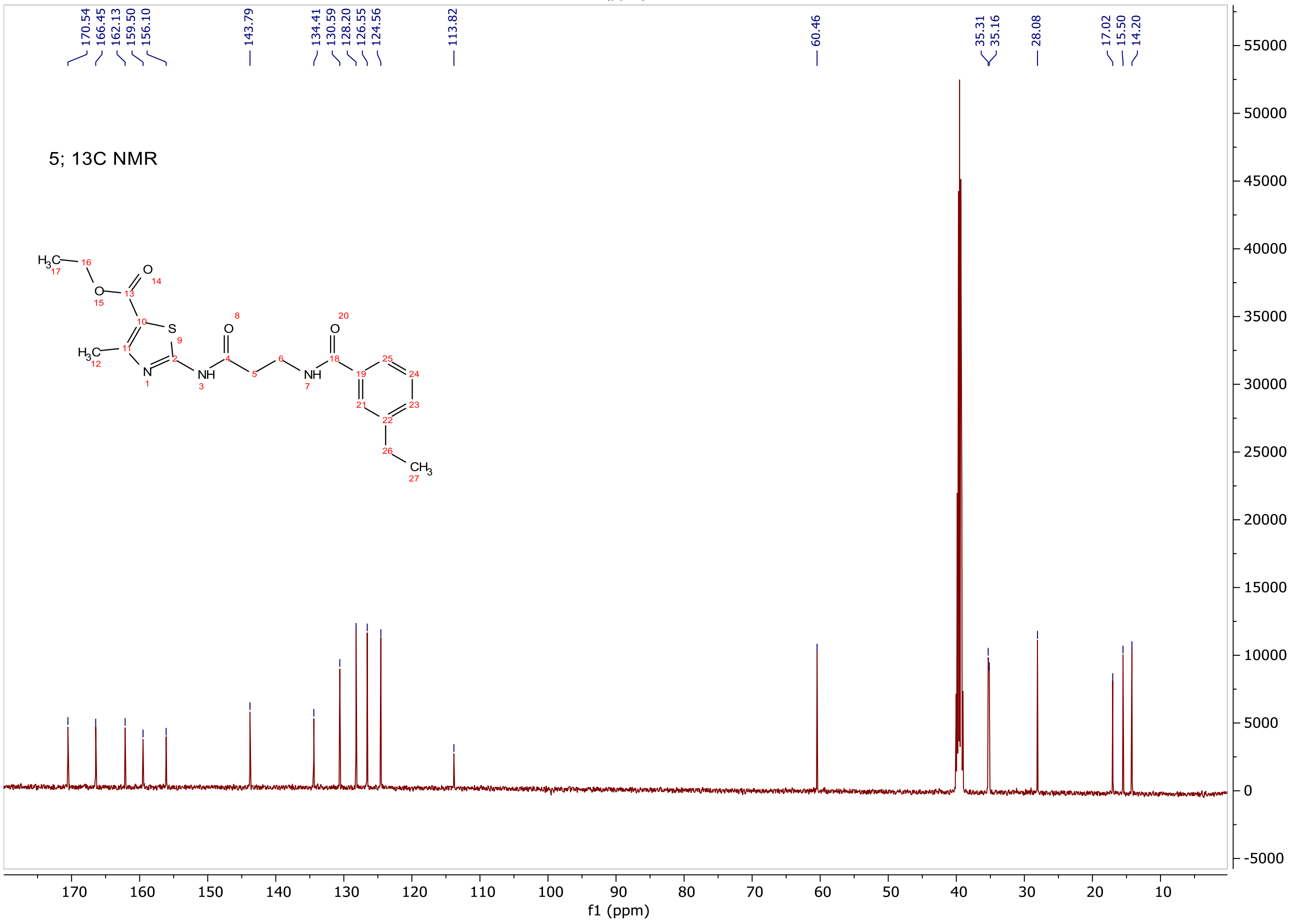
5; 13C NMR
170.54
166.45
162.13
159.50
156.10
143.79
134.41
130.59
128.20
126.55
124.56
113.82
60.46
35.31
35.16
28.08
17.02
15.50
14.20
f1 (ppm)

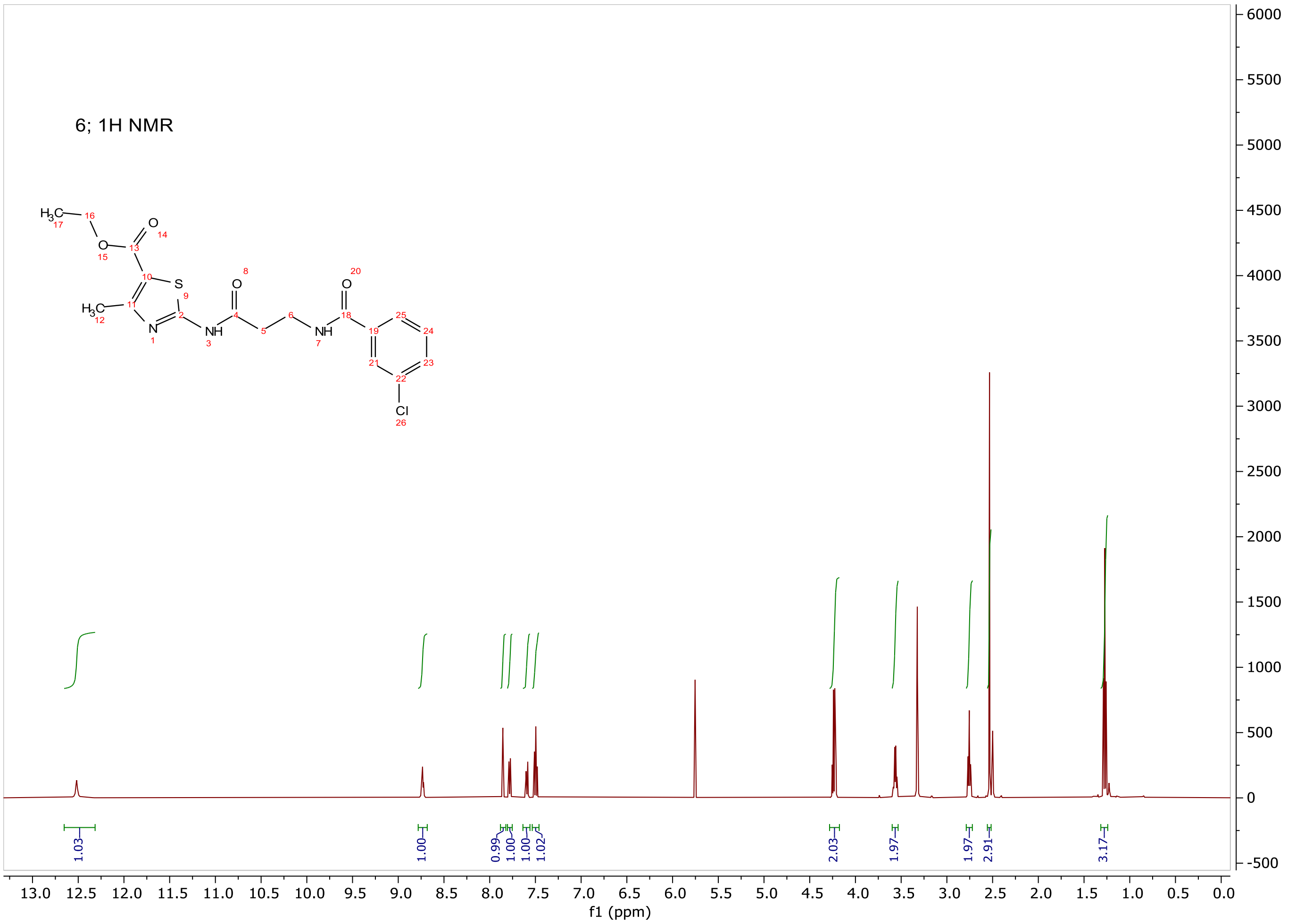
6; 1H NMR
f1 (ppm)

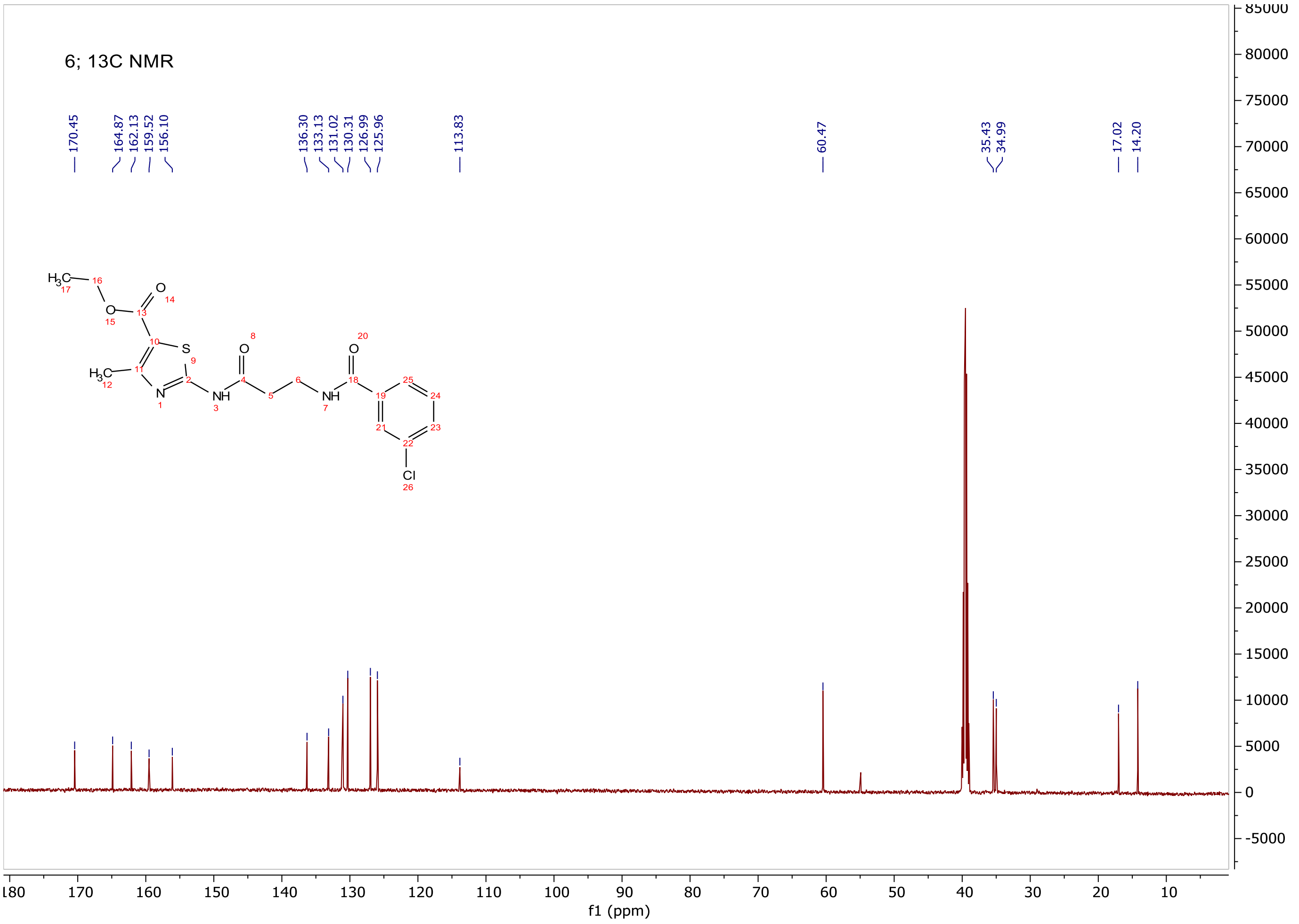
6; 13C NMR
170.45
164.87
162.13
159.52
156.10
136.30
133.13
131.02
130.31
126.99
125.96
113.83
60.47
35.43
34.99
17.02
14.20
f1 (ppm)

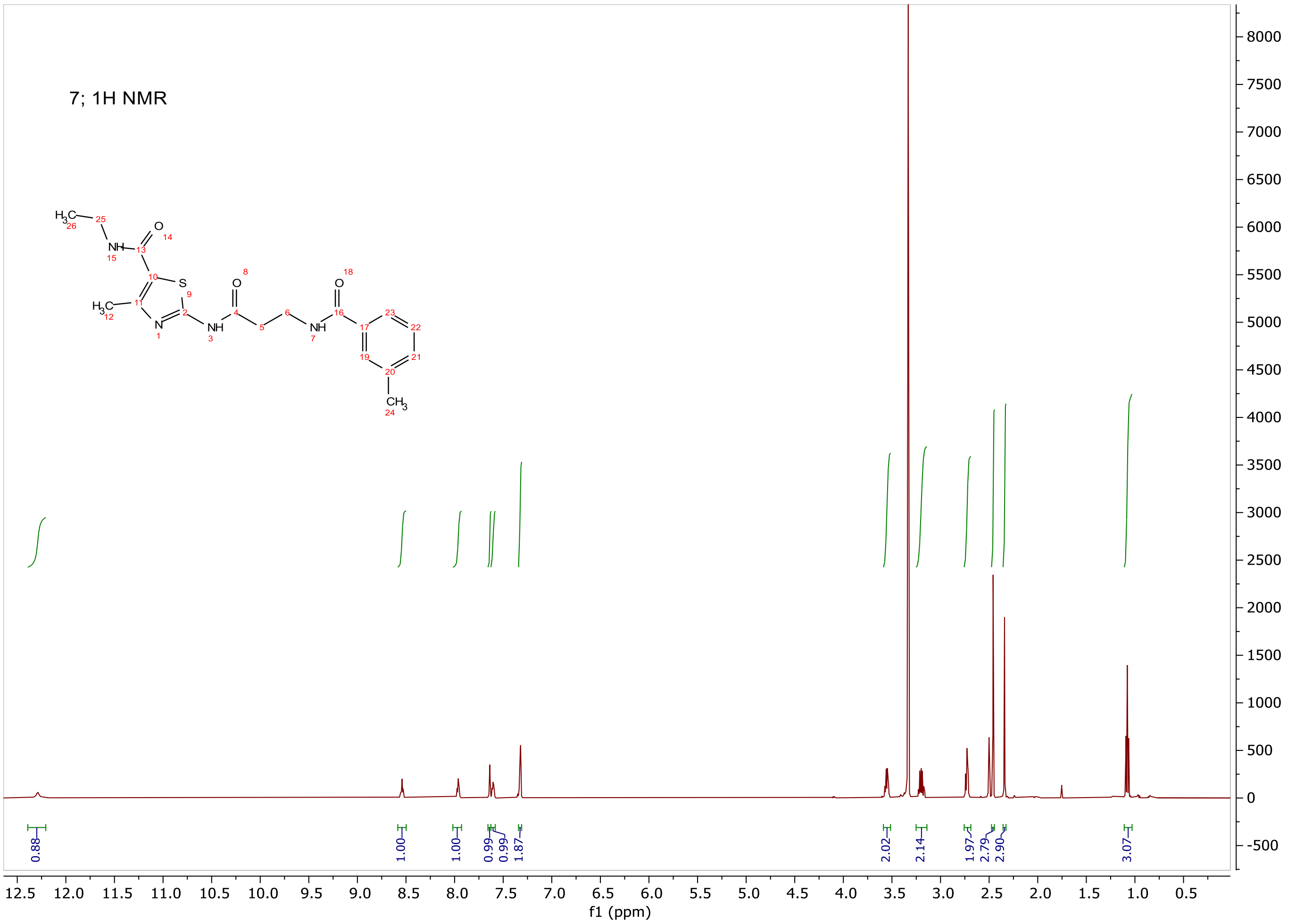
7; 1H NMR
0.88
1.00
1.00
0.99
0.99
1.87
2.02
2.14
1.97
2.79
2.90
3.07
f1 (ppm)

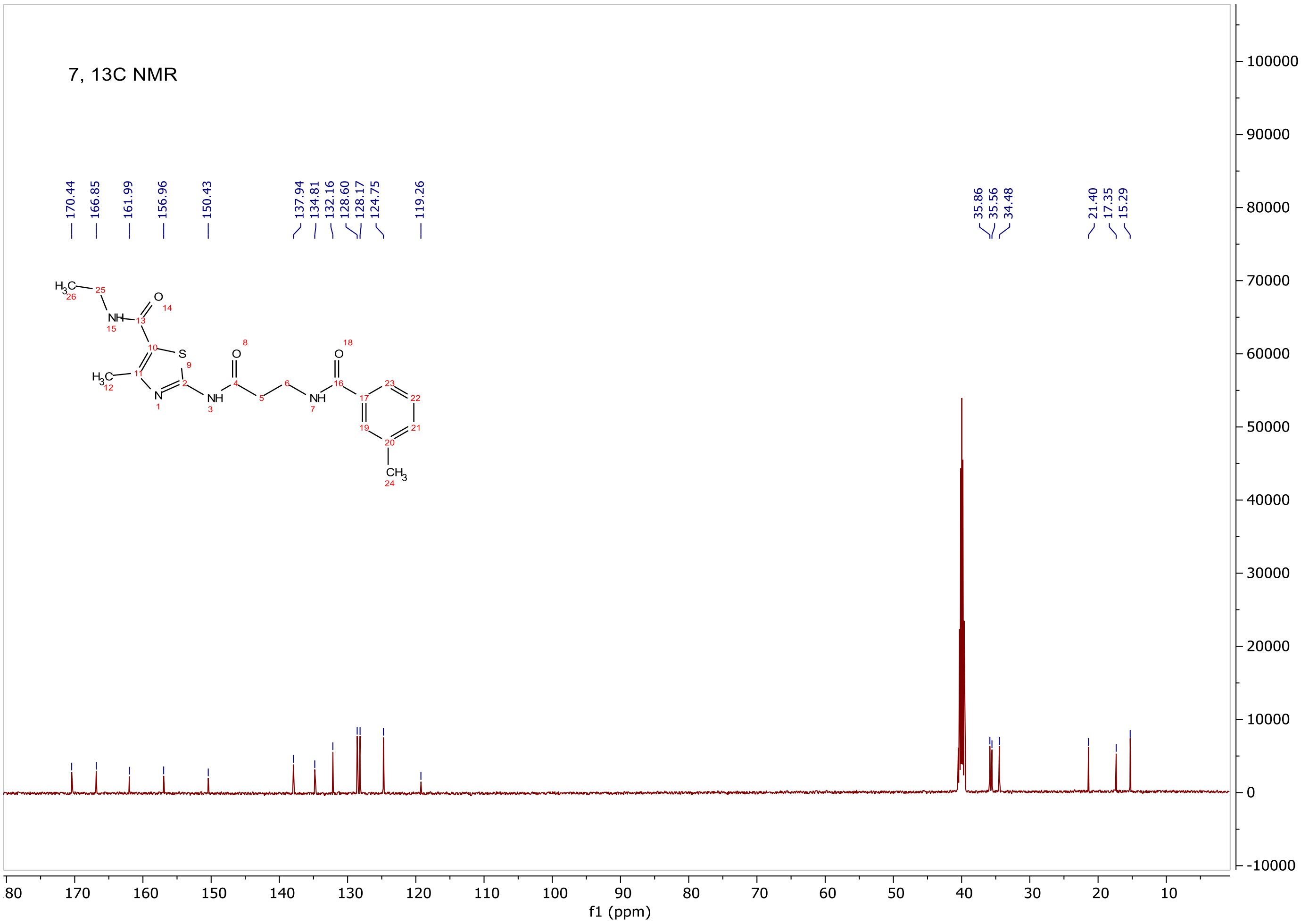
7, 13C NMR
170.44
166.85
161.99
156.96
150.43
137.94
134.81
132.16
128.60
128.17
124.75
119.26
35.86
35.56
34.48
21.40
17.35
15.29
f1 (ppm)

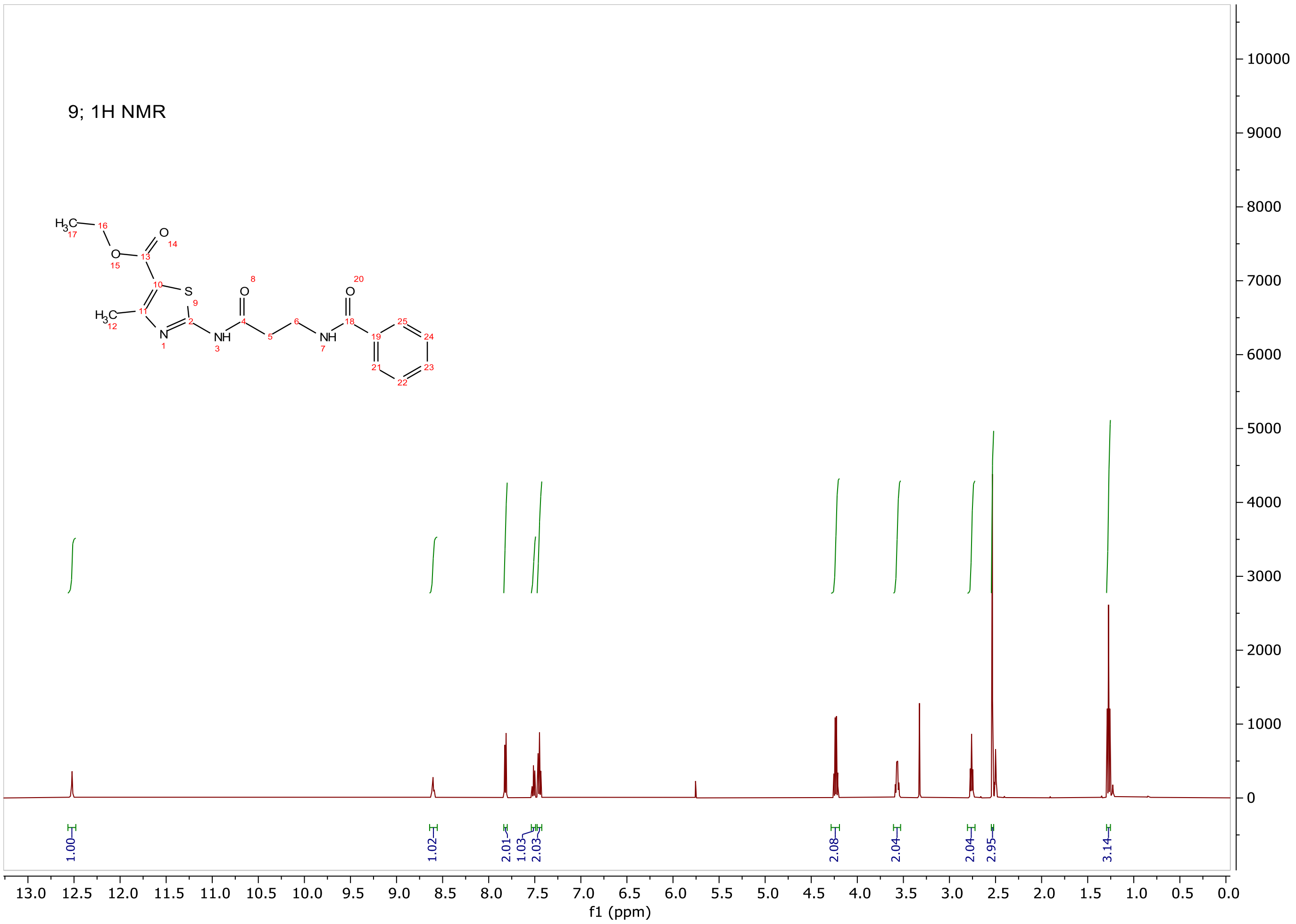
9; 1H NMR
1.00
1.02
2.01
1.03
2.03
2.08
2.04
2.04
2.95
3.14
f1 (ppm)

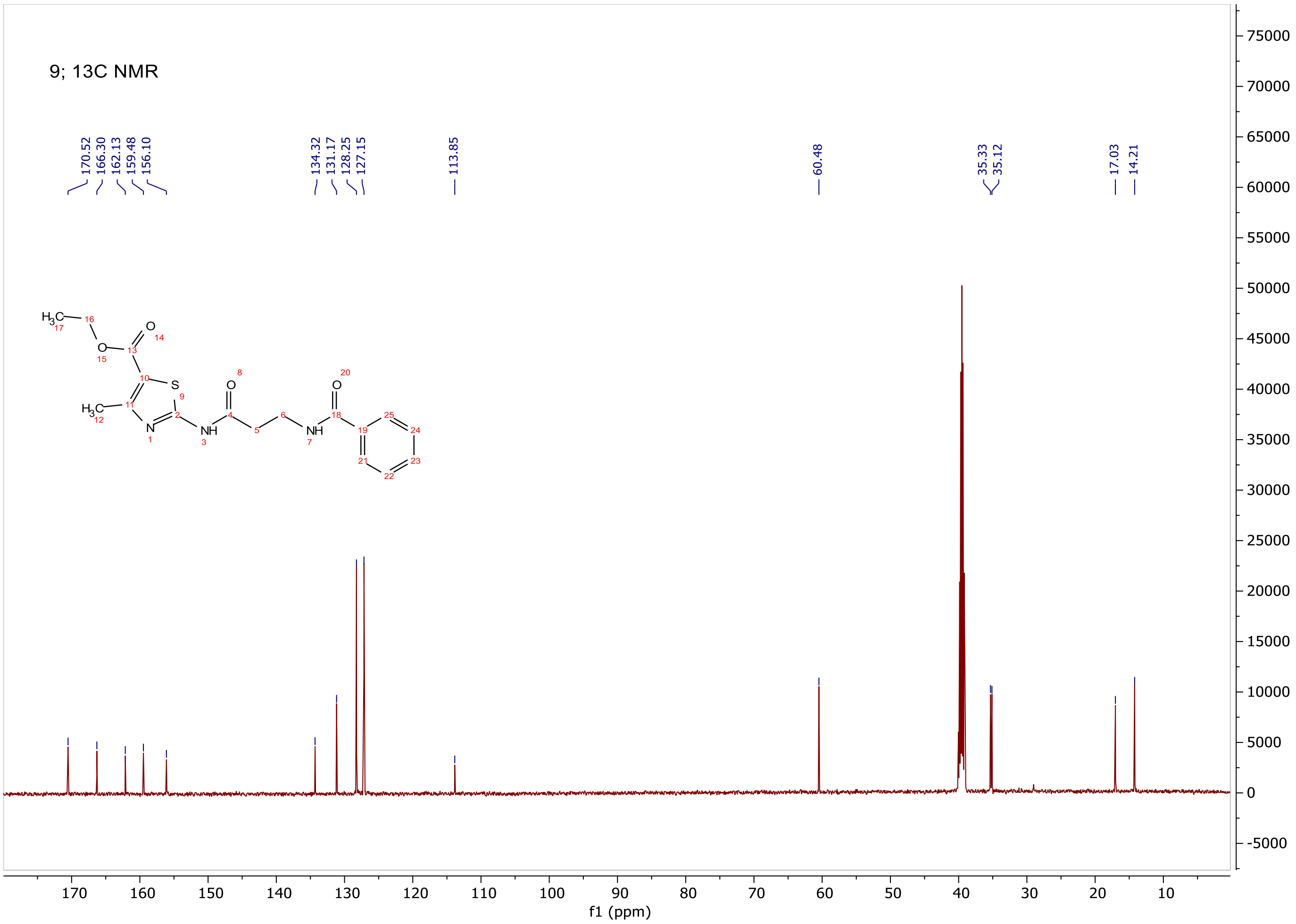
9; 13C NMR
170.52
166.30
162.13
159.48
156.10
134.32
131.17
128.25
127.15
113.85
60.48
35.33
35.12
17.03
14.21
f1 (ppm)

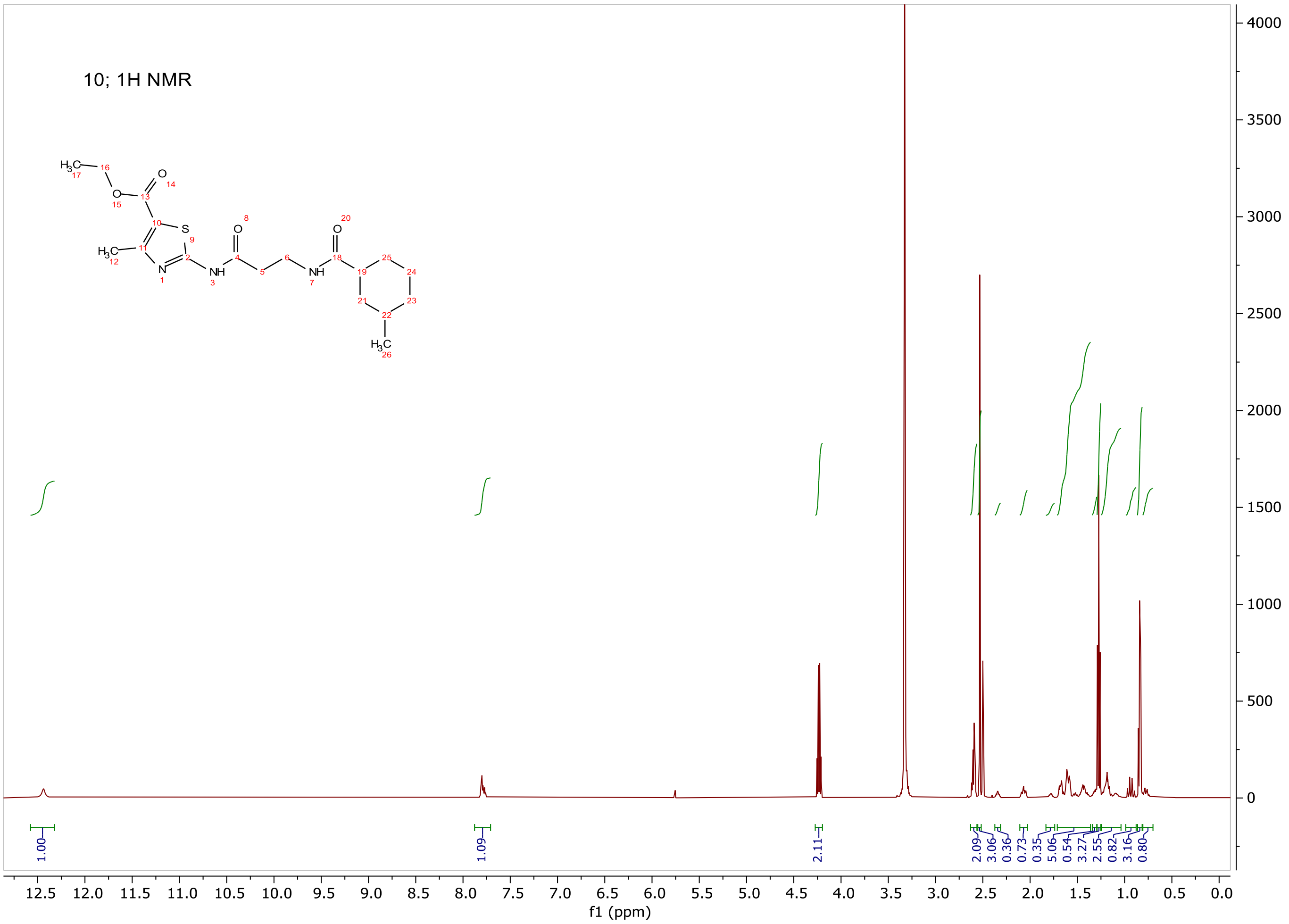
10; 1H NMR
f1 (ppm)

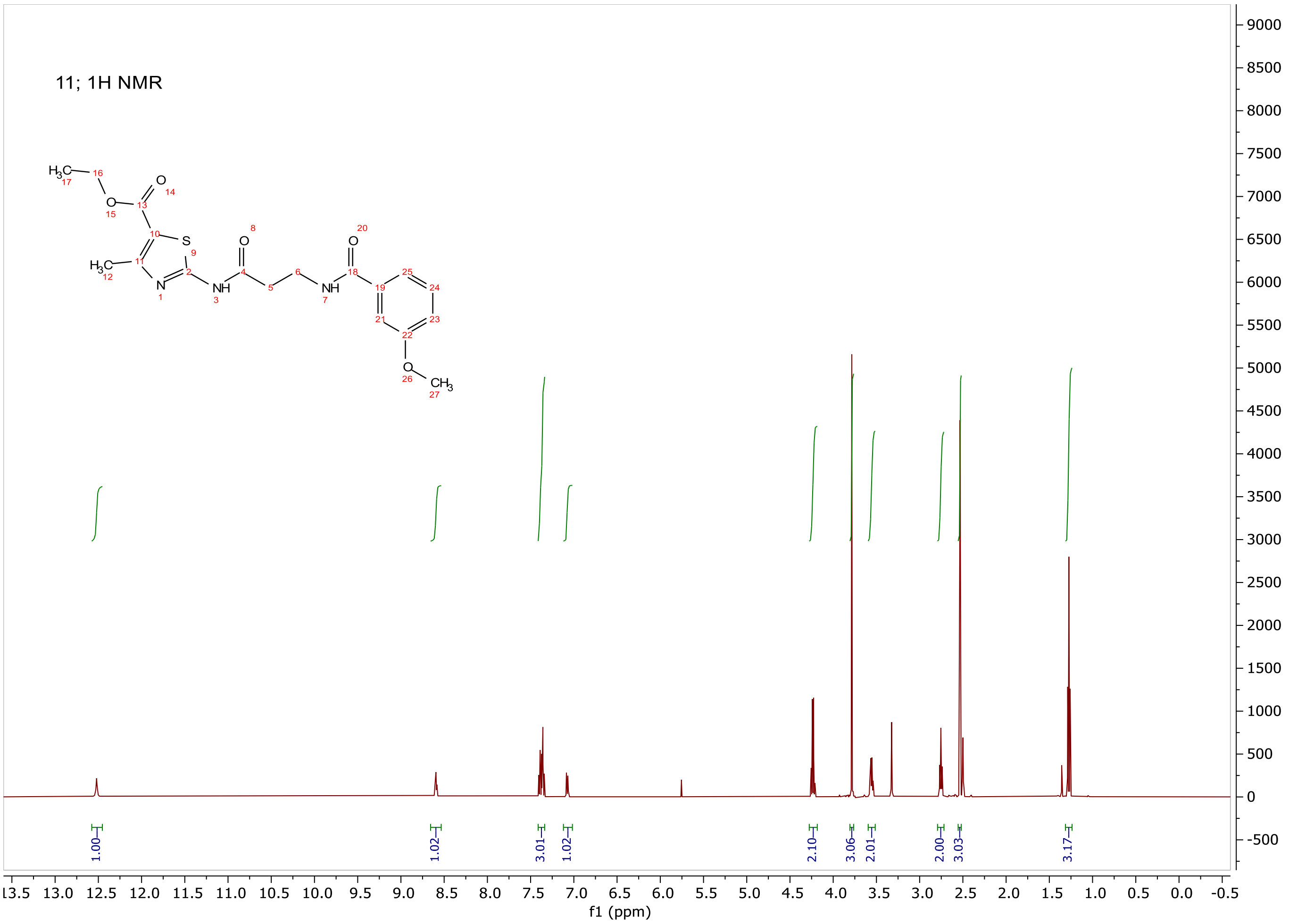
11; 1H NMR
f1 (ppm)

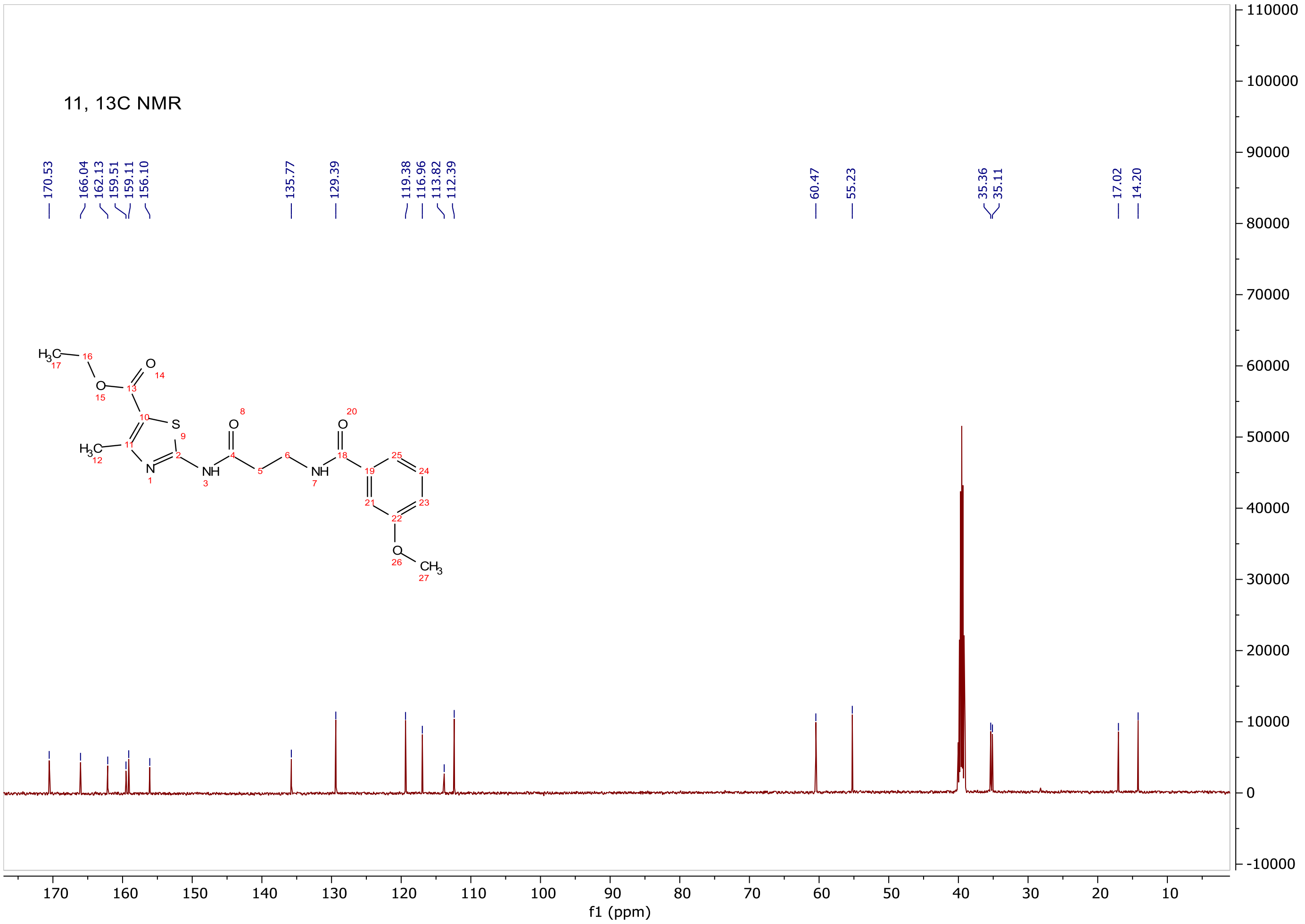
11, 13C NMR
170.53
166.04
162.13
159.51
159.11
156.10
135.77
129.39
119.38
116.96
113.82
112.39
60.47
55.23
35.36
35.11
17.02
14.20
f1 (ppm)

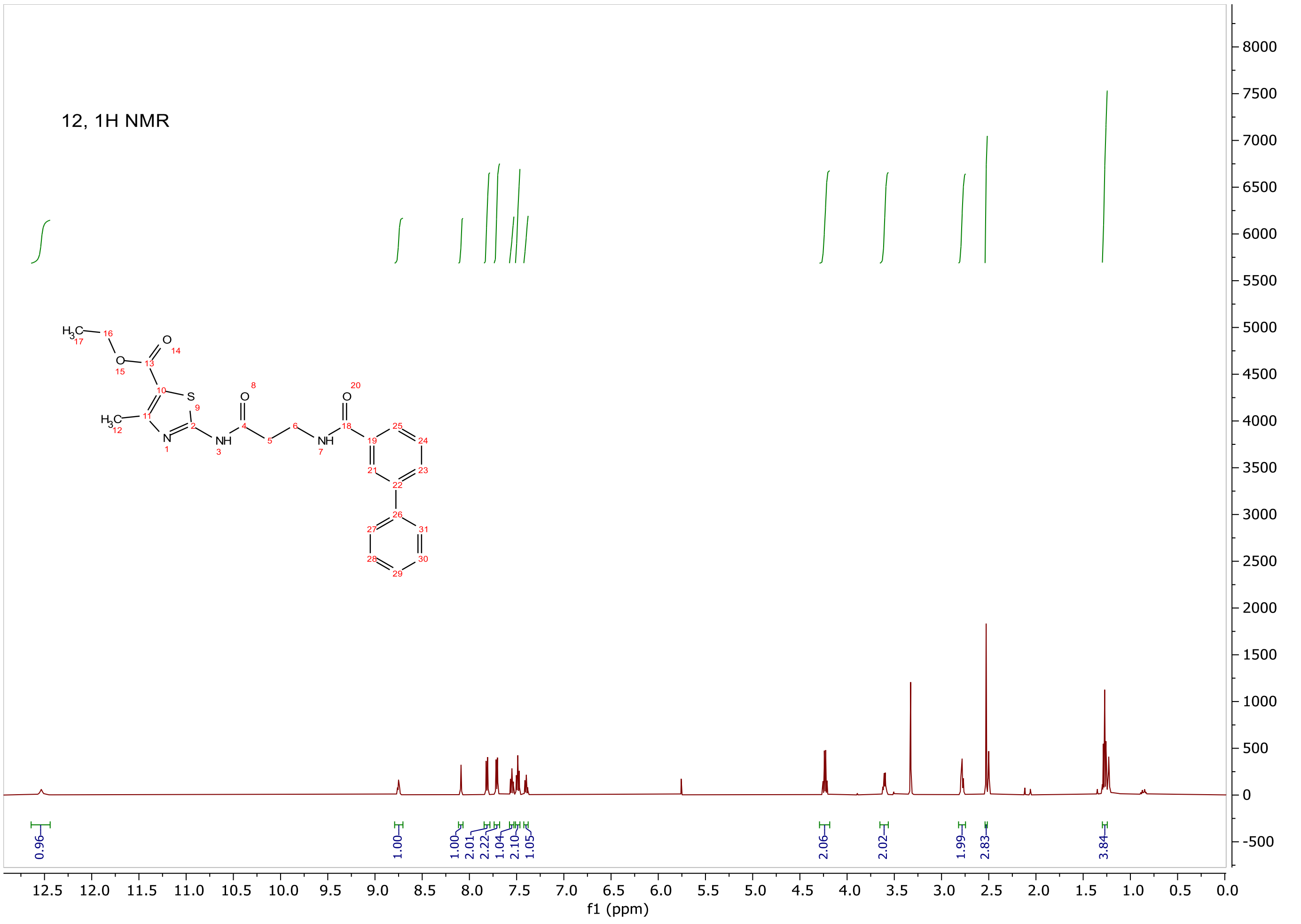
12, 1H NMR
0.96
1.00
1.00
2.01
2.22
1.04
2.10
1.05
2.06
2.02
1.99
2.83
3.84
f1 (ppm)

12; 13C NMR

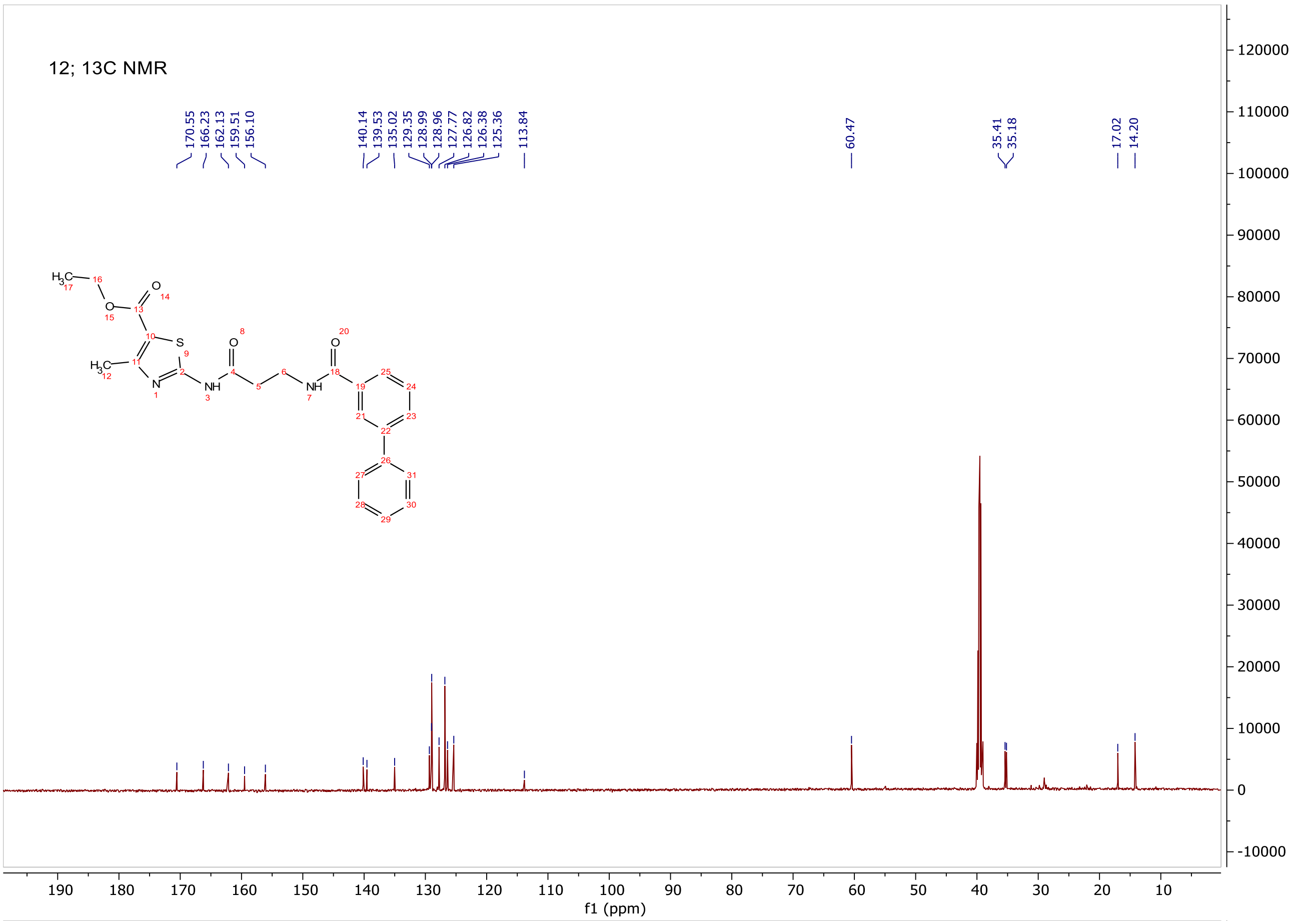

13; 1H NMR

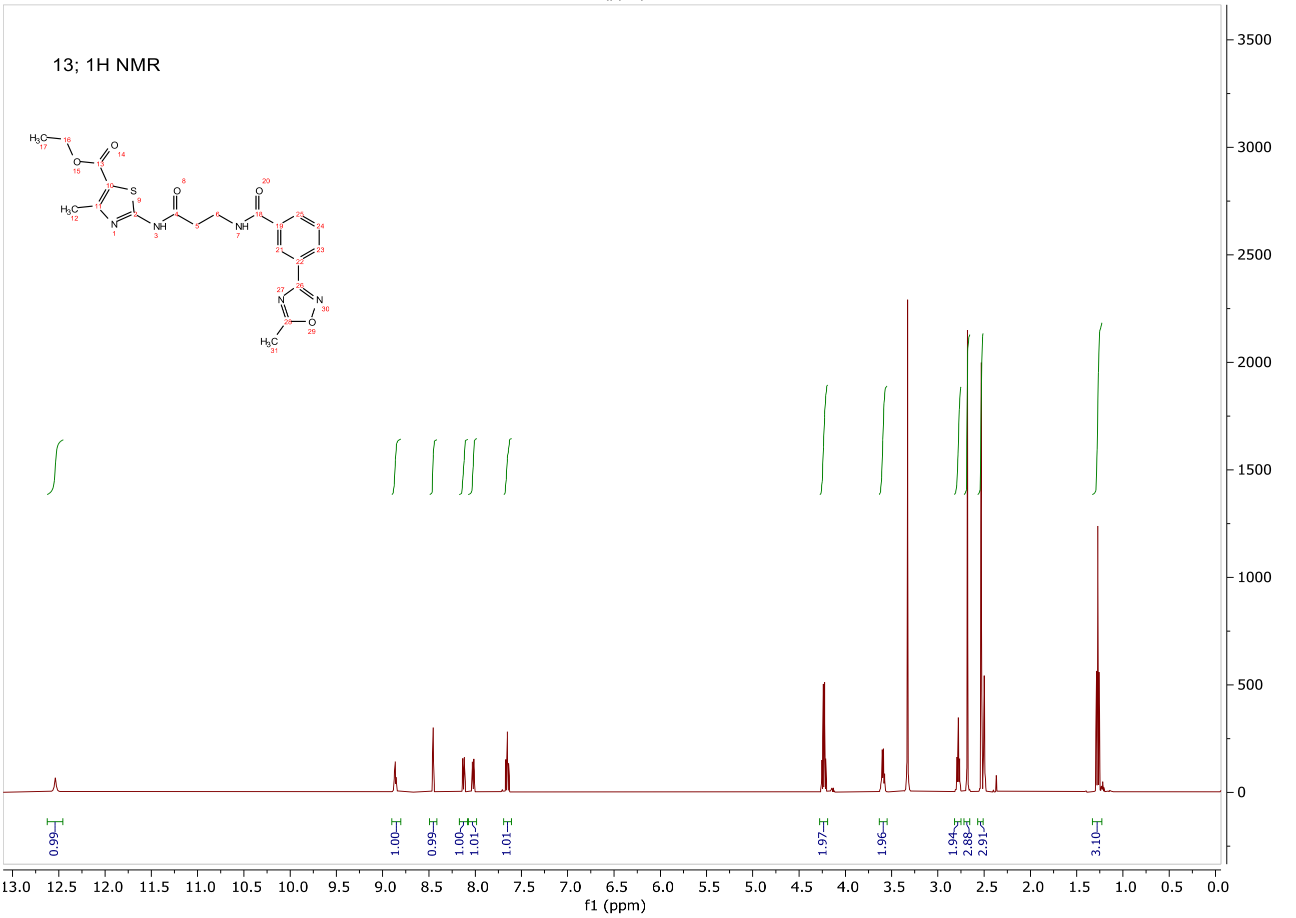

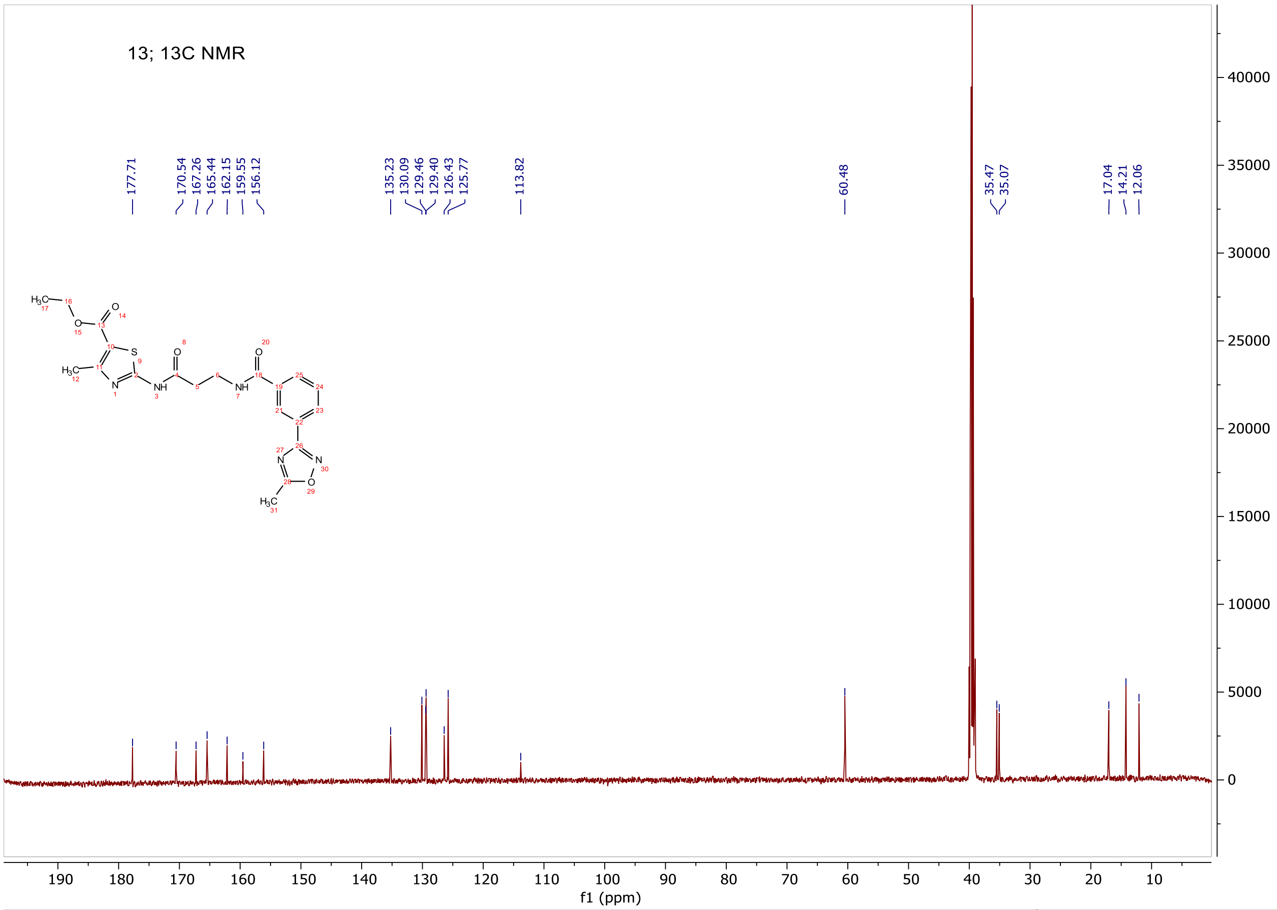
13; 13C NMR
177.71
170.54
167.26
165.44
162.15
159.55
156.12
135.23
130.09
129.46
129.40
126.43
125.77
113.82
60.48
35.47
35.07
17.04
14.21
12.06
f1 (ppm)

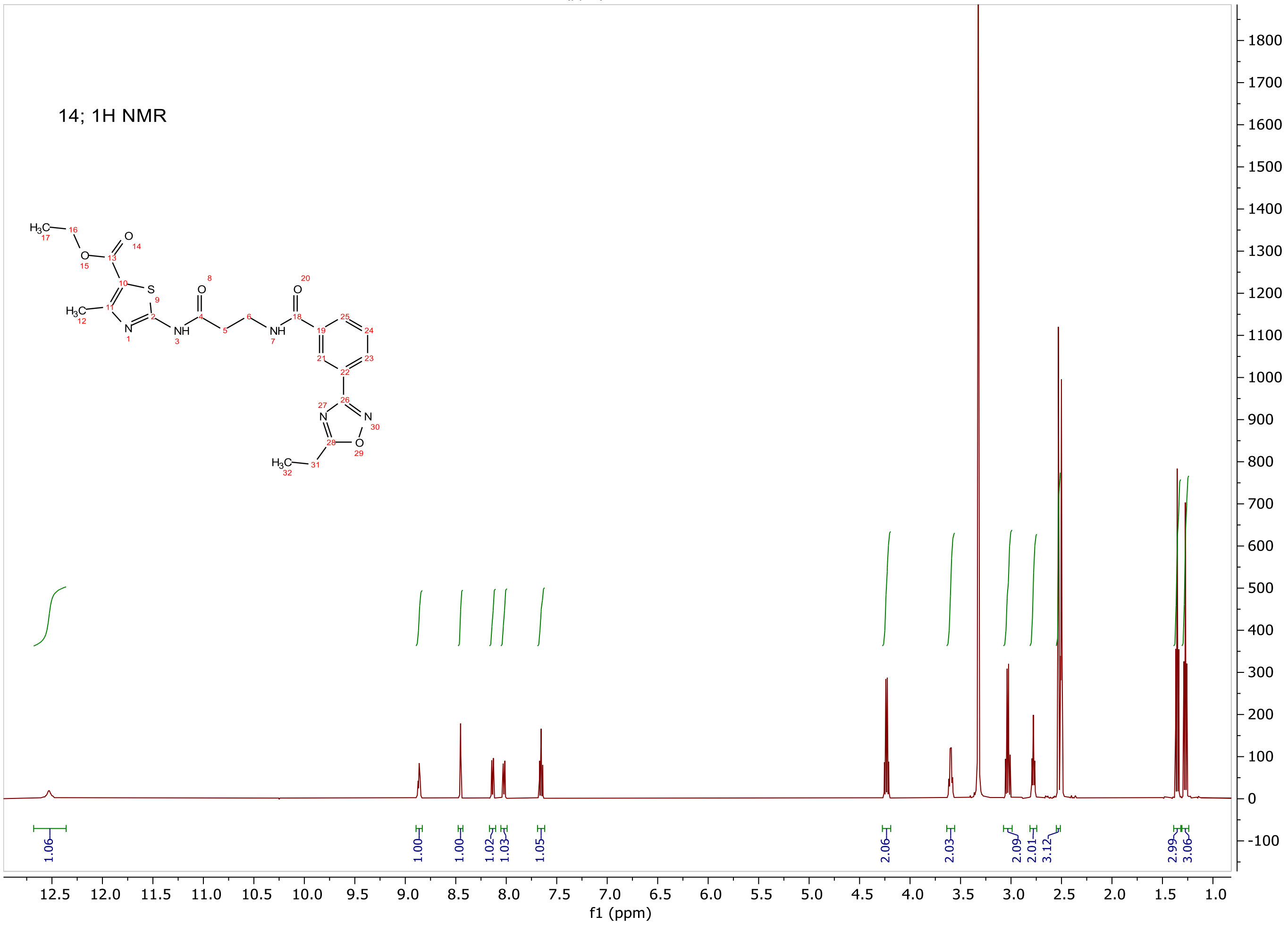
14; 1H NMR
1.06
1.00
1.00
1.02
1.03
1.05
2.06
2.03
2.09
2.01
3.12
2.99
3.06
f1 (ppm)

14; 13C NMR

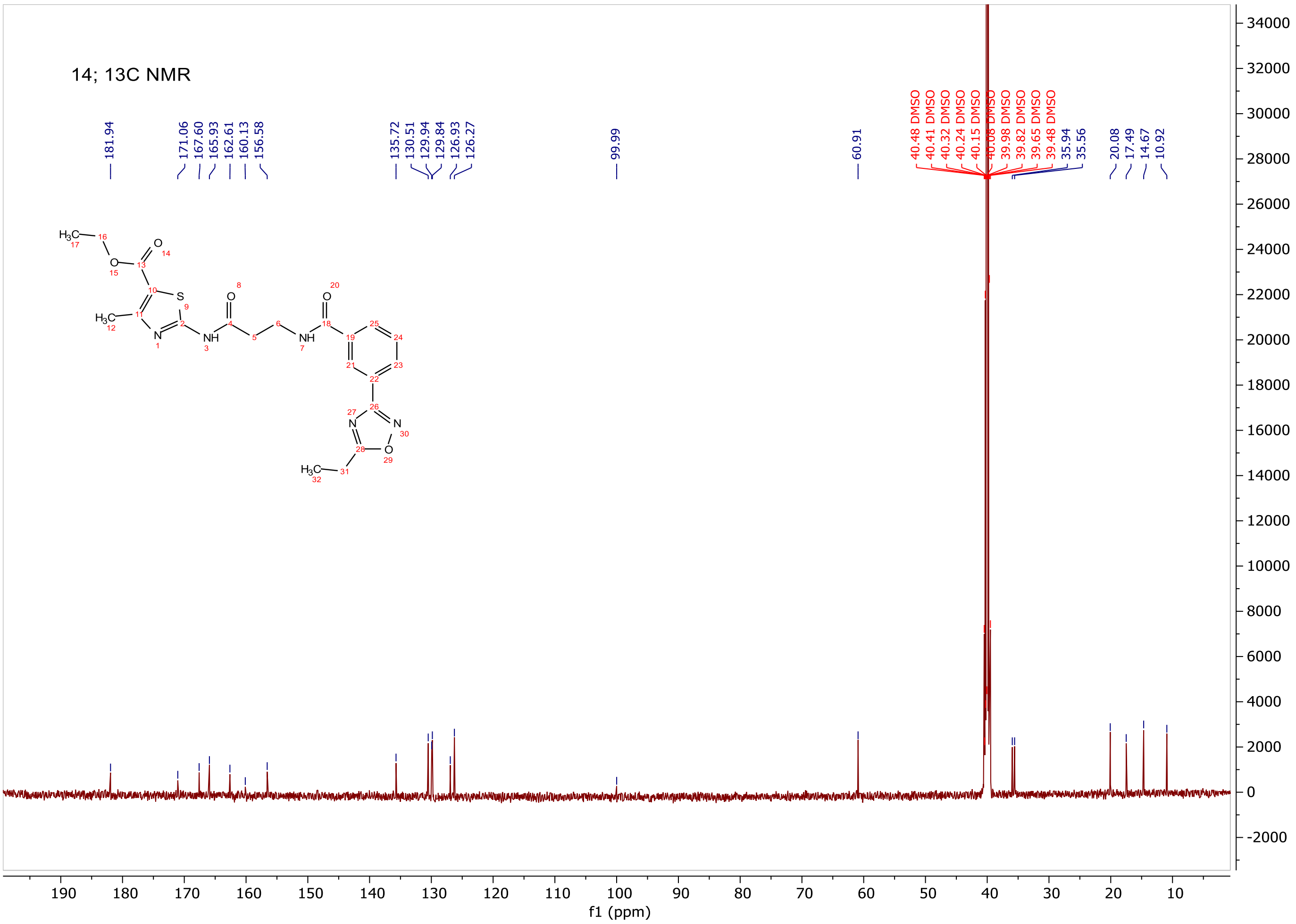

15, 1H NMR

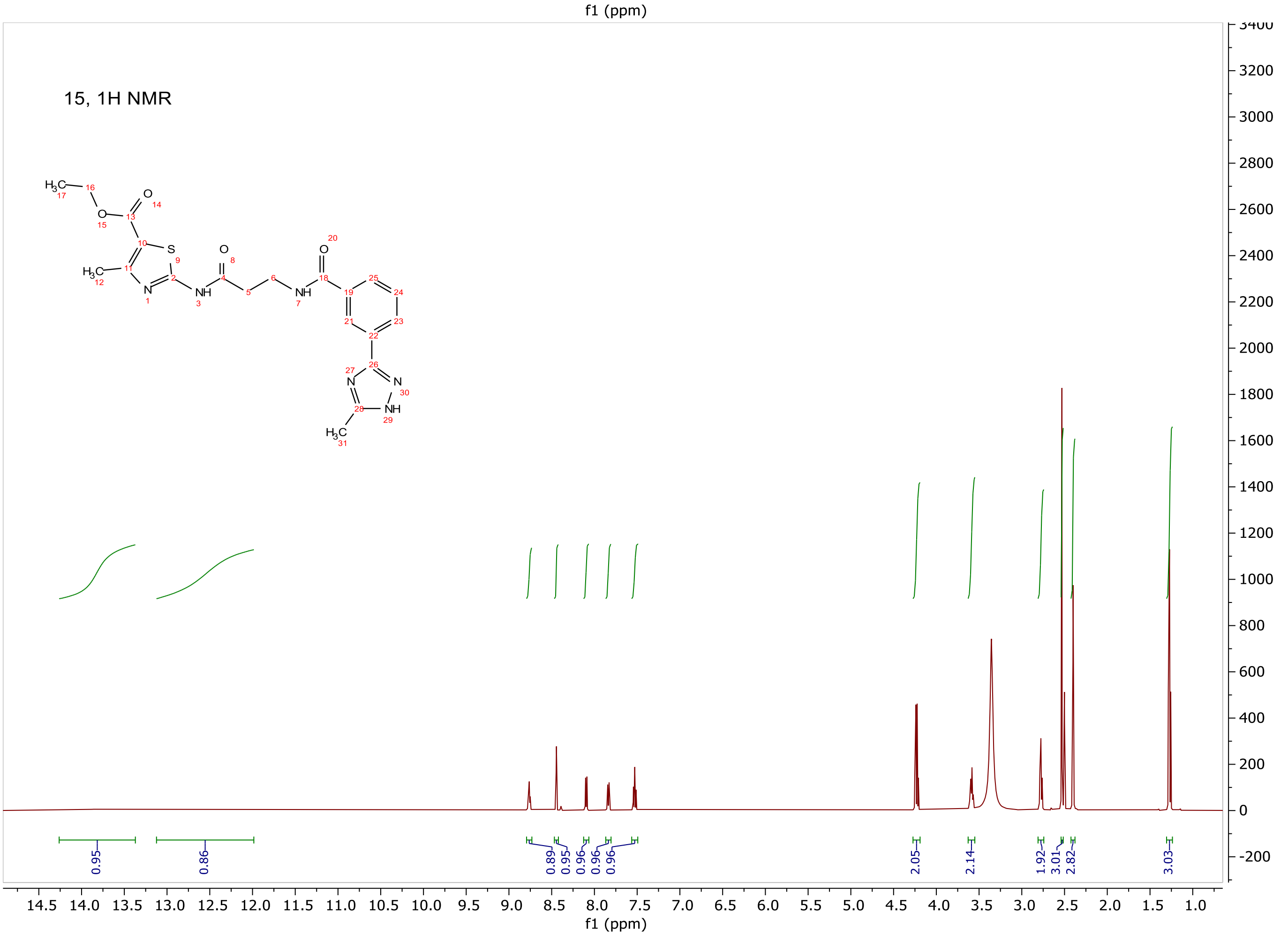

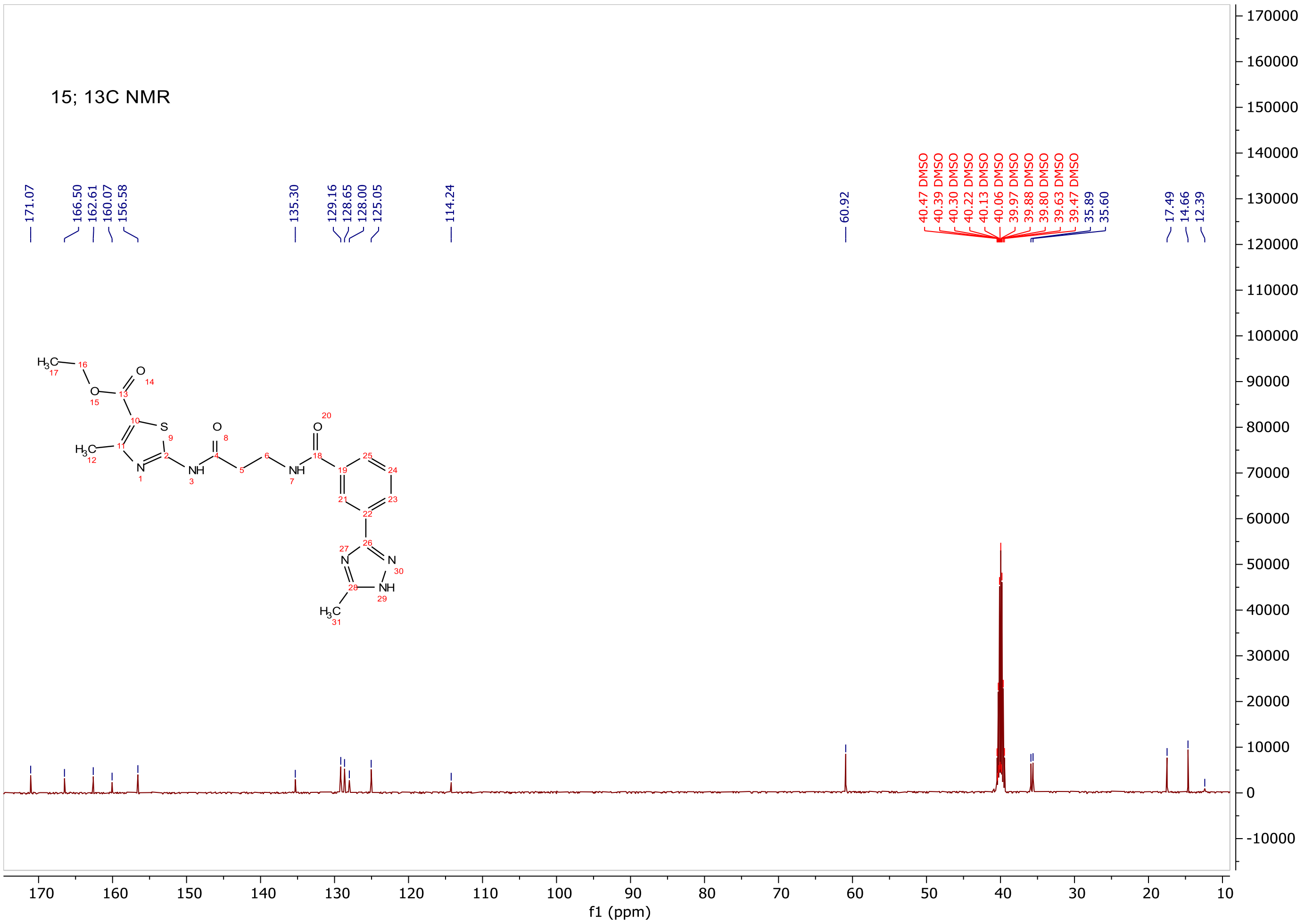
15; 13C NMR
171.07
166.50
162.61
160.07
156.58
135.30
129.16
128.65
128.00
125.05
114.24
60.92
40.47 DMSO
40.39 DMSO
40.30 DMSO
40.22 DMSO
40.13 DMSO
40.06 DMSO
39.97 DMSO
39.88 DMSO
39.80 DMSO
39.63 DMSO
39.47 DMSO
35.89
35.60
17.49
14.66
12.39
f1 (ppm)

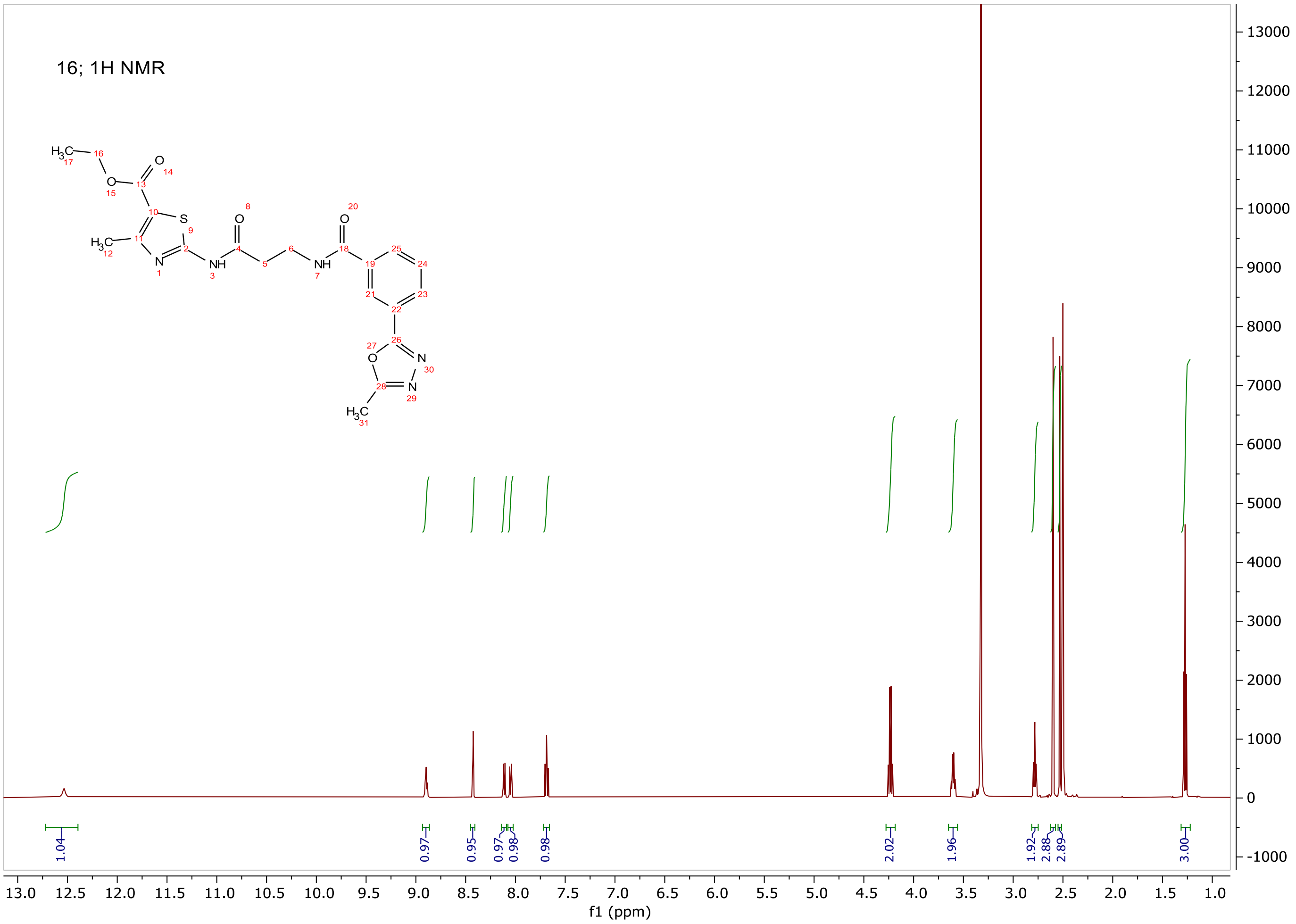
16; 1H NMR
1.04
0.97
0.95
0.97
0.98
0.98
2.02
1.96
1.92
2.88
2.89
3.00
f1 (ppm)

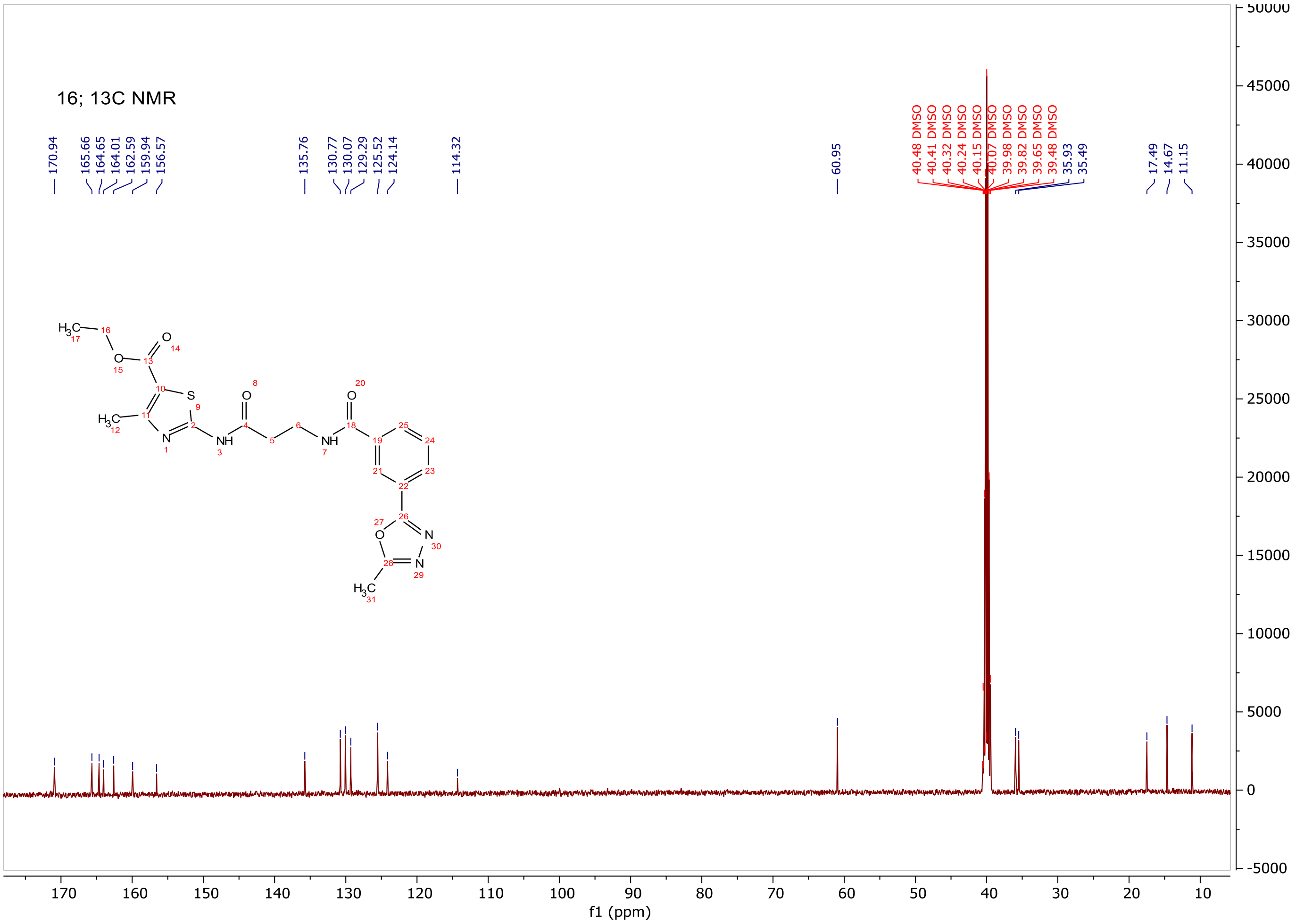
16; 13C NMR
170.94
165.66
164.65
164.01
162.59
159.94
156.57
135.76
130.77
130.07
129.29
125.52
124.14
114.32
60.95
40.48 DMSO
40.41 DMSO
40.32 DMSO
40.24 DMSO
40.15 DMSO
40.07 DMSO
39.98 DMSO
39.82 DMSO
39.65 DMSO
39.48 DMSO
35.93
35.49
17.49
14.67
11.15
f1 (ppm)

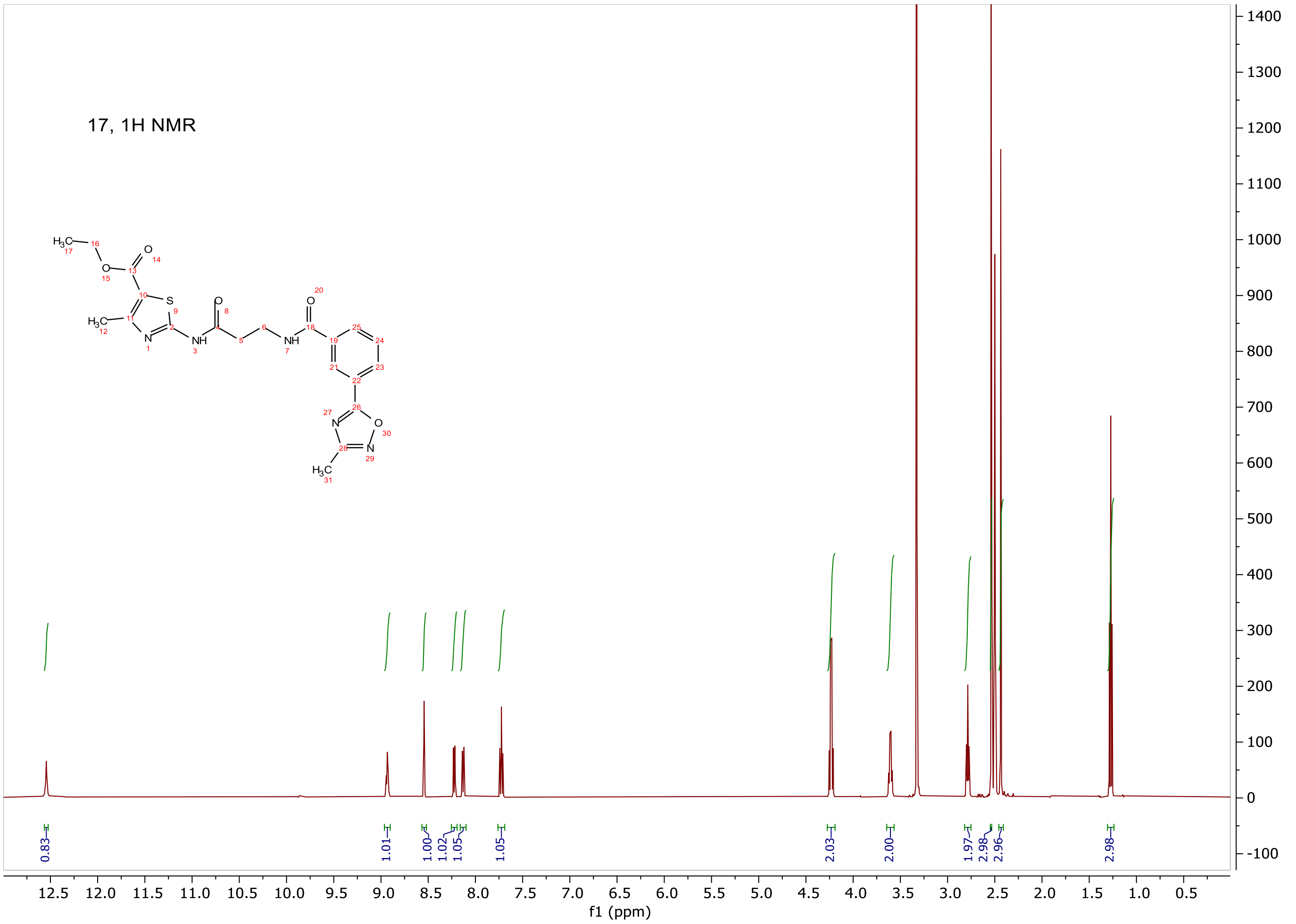
17, 1H NMR
0.83
1.01
1.00
1.02
1.05
1.05
2.03
2.00
1.97
2.98
2.96
2.98
f1 (ppm)

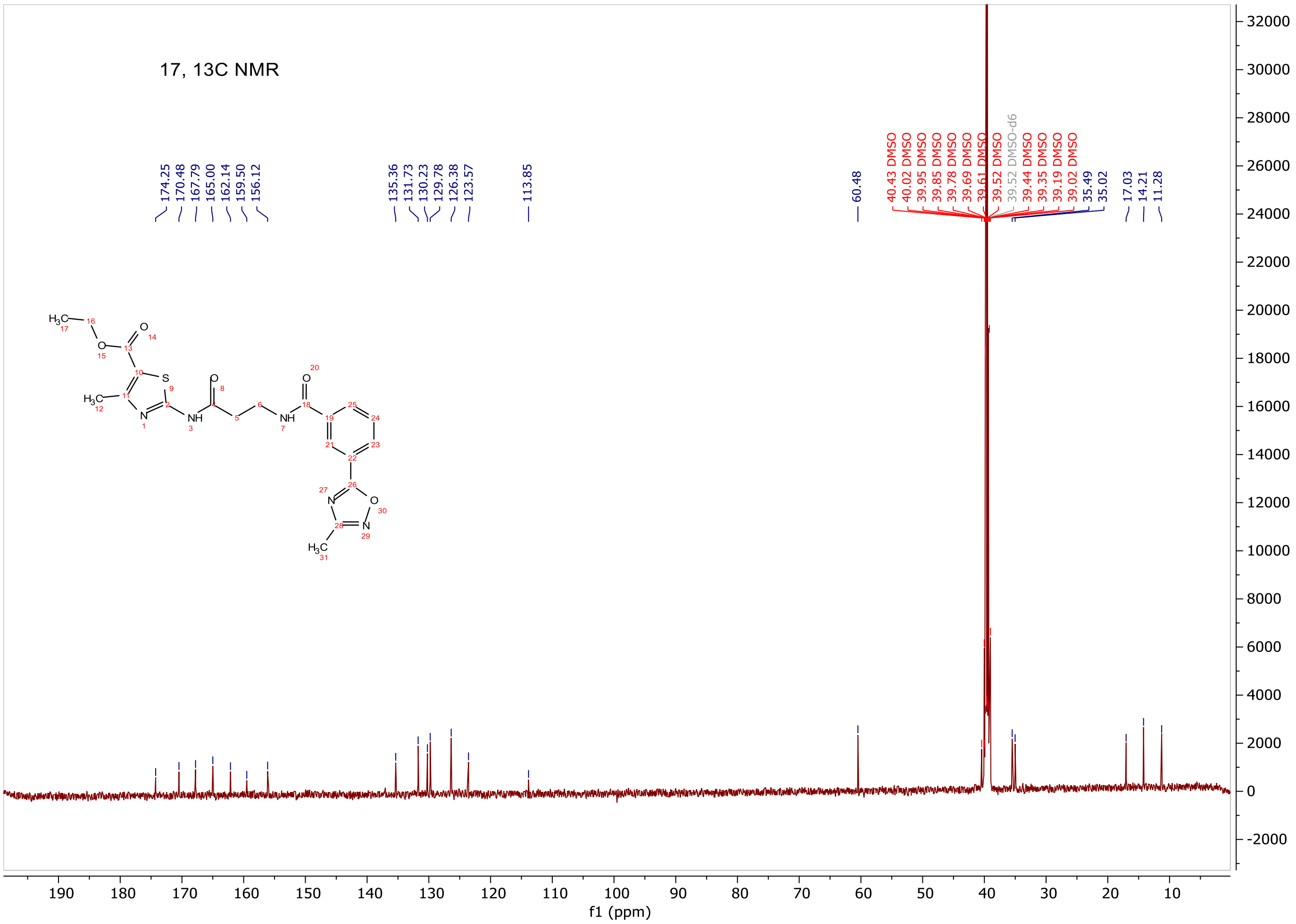
17, 13C NMR
174.25
170.48
167.79
165.00
162.14
159.50
156.12
135.36
131.73
130.23
129.78
126.38
123.57
113.85
60.48
40.43 DMSO
40.02 DMSO
39.95 DMSO
39.85 DMSO
39.78 DMSO
39.69 DMSO
39.61 DMSO
39.52 DMSO
39.52 DMSO-d6
39.44 DMSO
39.35 DMSO
39.19 DMSO
39.02 DMSO
35.49
35.02
17.03
14.21
11.28
f1 (ppm)

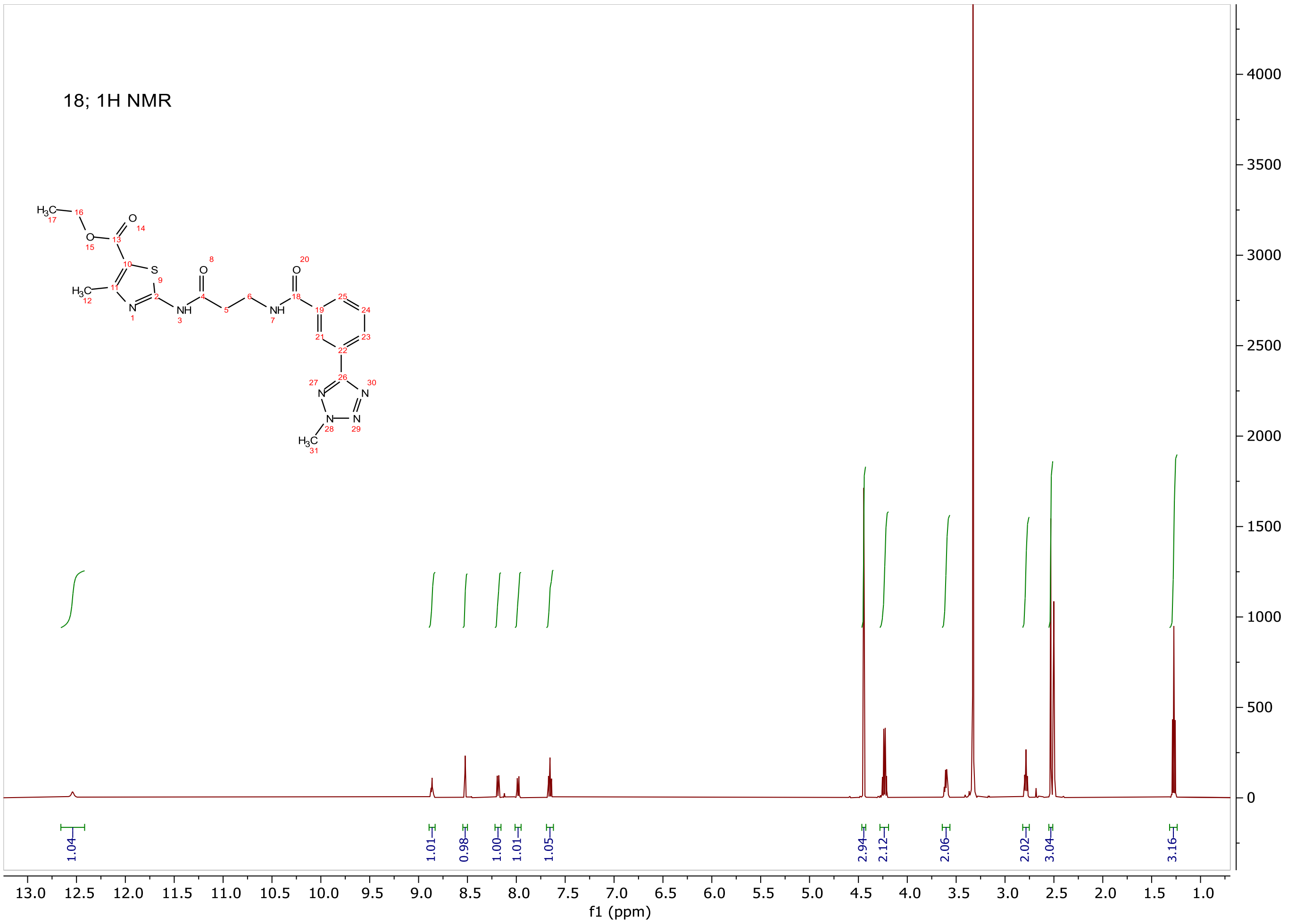
18; 1H NMR
1.04
1.01
0.98
1.00
1.01
1.05
2.94
2.12
2.06
2.02
3.04
3.16
f1 (ppm)

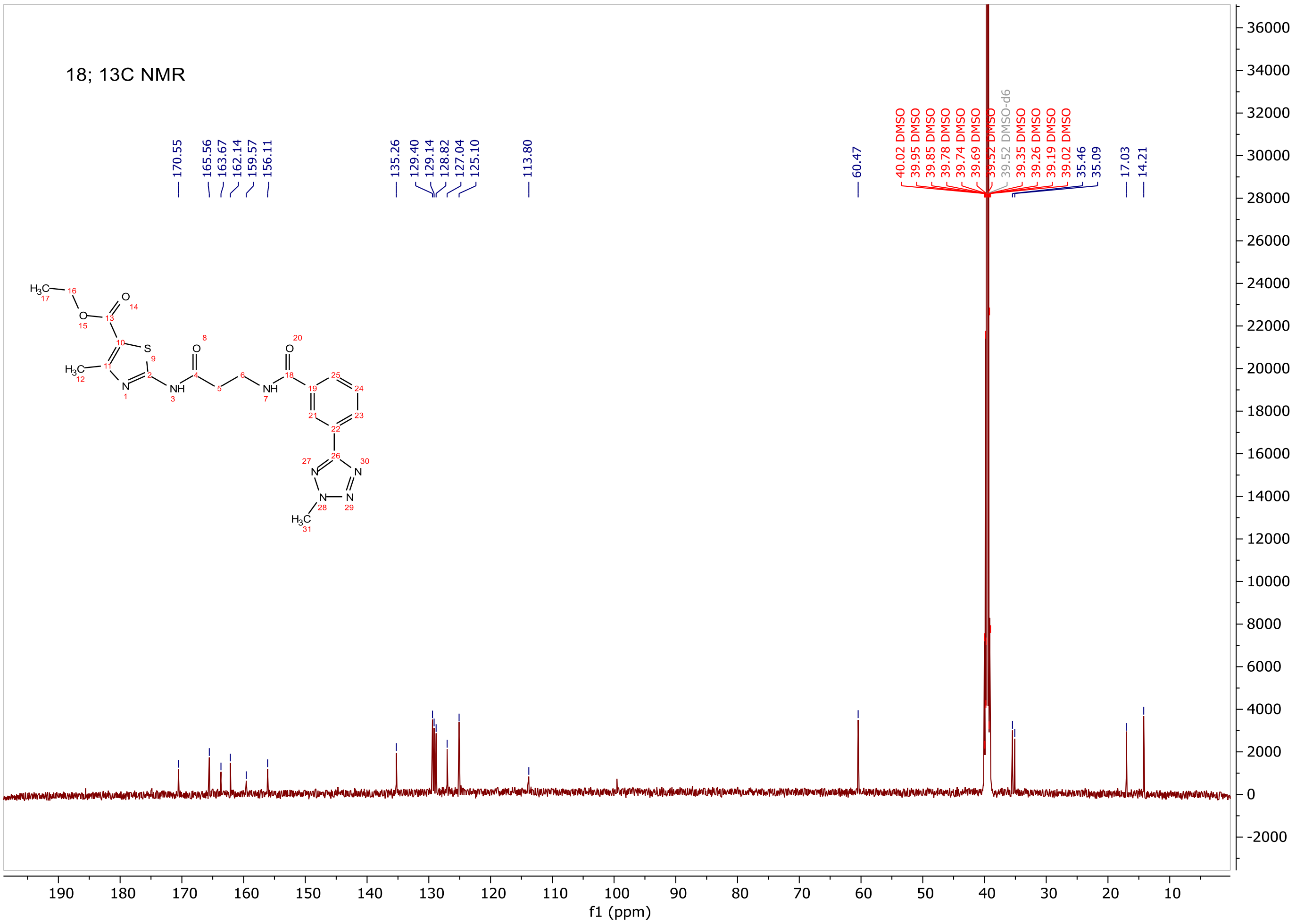
18; 13C NMR
170.55
165.56
163.67
162.14
159.57
156.11
135.26
129.40
129.14
128.82
127.04
125.10
113.80
60.47
40.02 DMSO
39.95 DMSO
39.85 DMSO
39.78 DMSO
39.74 DMSO
39.69 DMSO
39.52 DMSO
39.52 DMSO-d6
39.35 DMSO
39.26 DMSO
39.19 DMSO
39.02 DMSO
35.46
35.09
17.03
14.21
f1 (ppm)

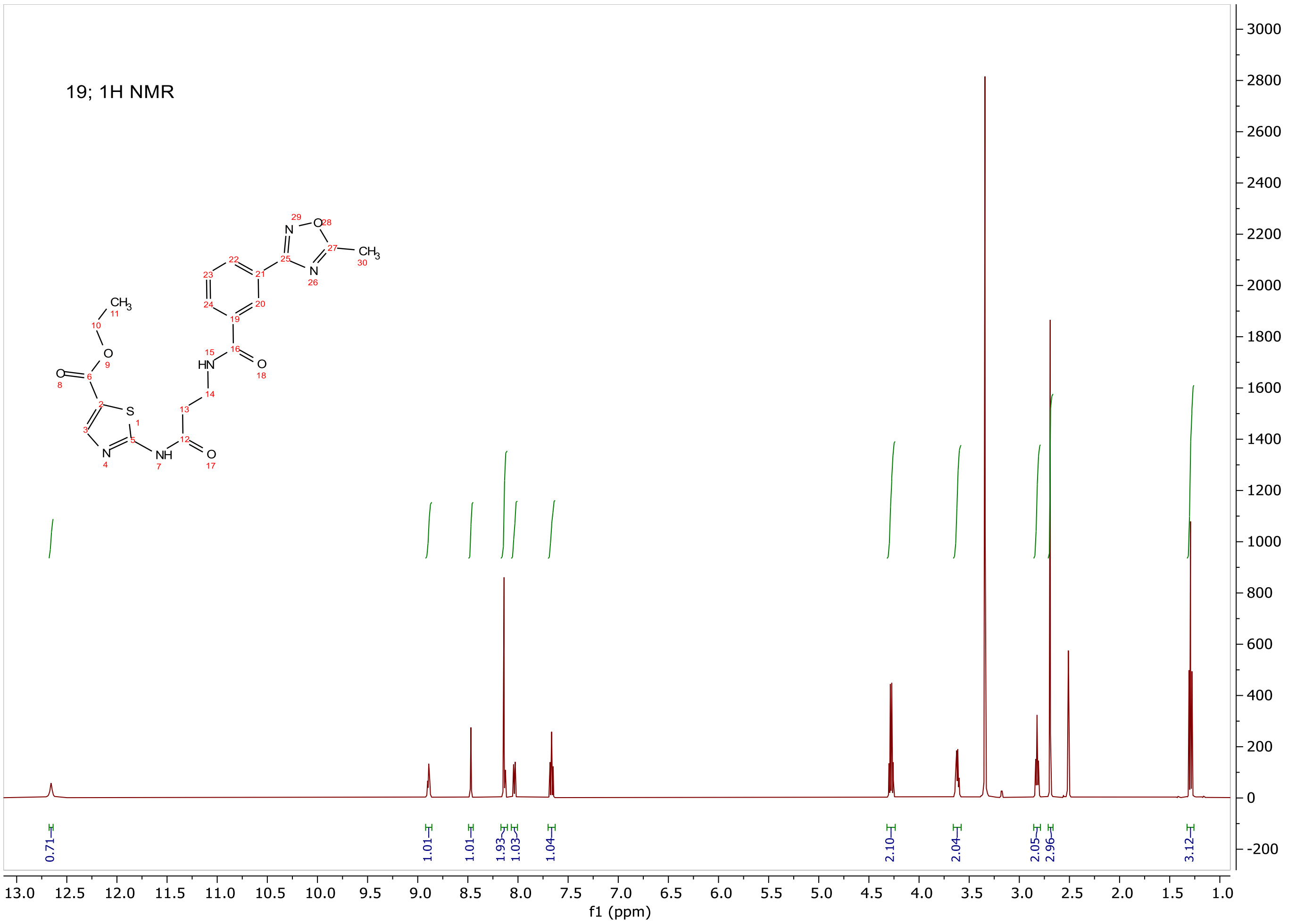
19; 1H NMR
0.71
1.01
1.01
1.93
1.03
1.04
2.10
2.04
2.05
2.96
3.12
f1 (ppm)

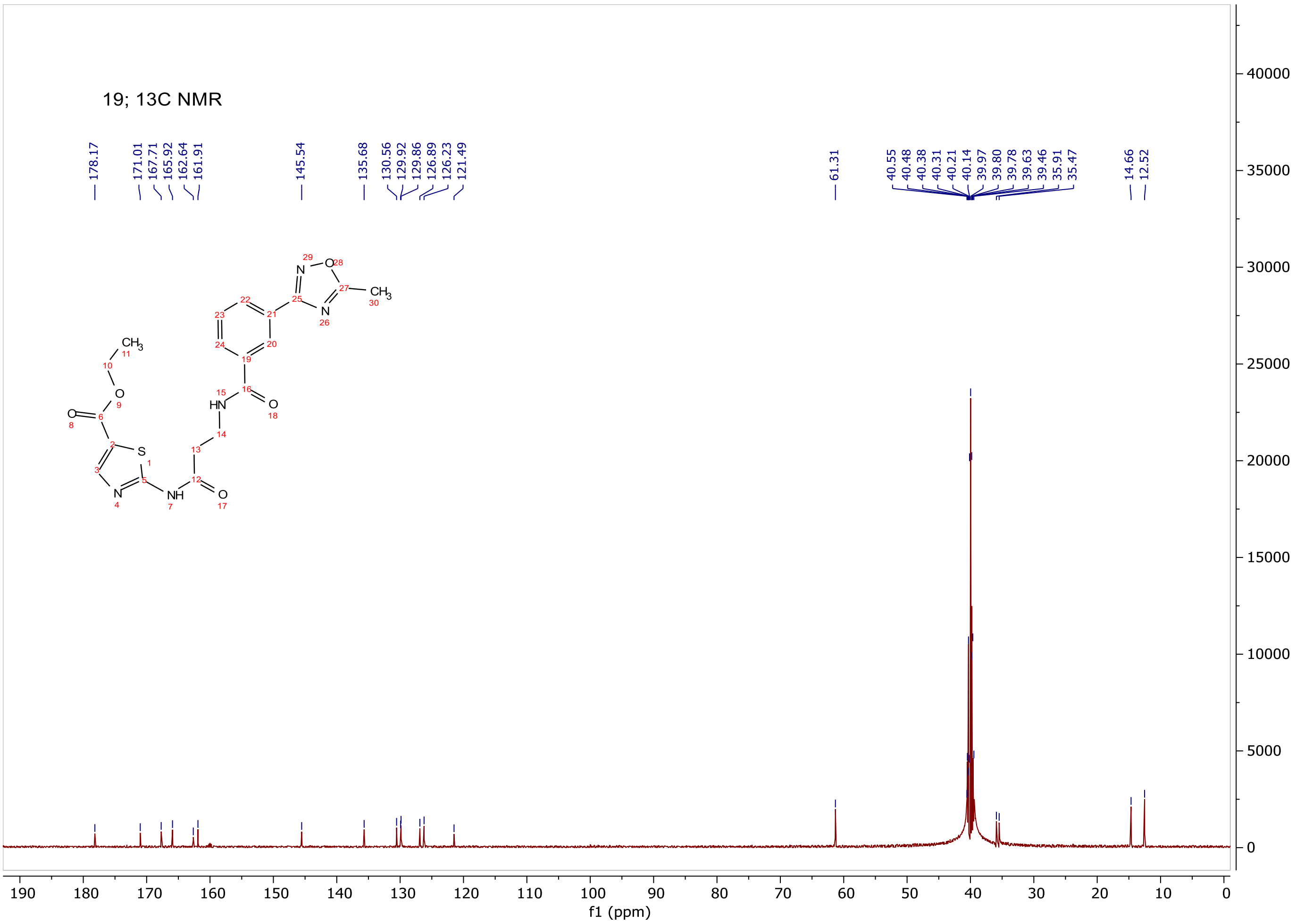
19; 13C NMR
178.17
171.01
167.71
165.92
162.64
161.91
145.54
135.68
130.56
129.92
129.86
126.89
126.23
121.49
61.31
40.55
40.48
40.38
40.31
40.21
40.14
39.97
39.80
39.78
39.63
39.46
35.91
35.47
14.66
12.52
f1 (ppm)

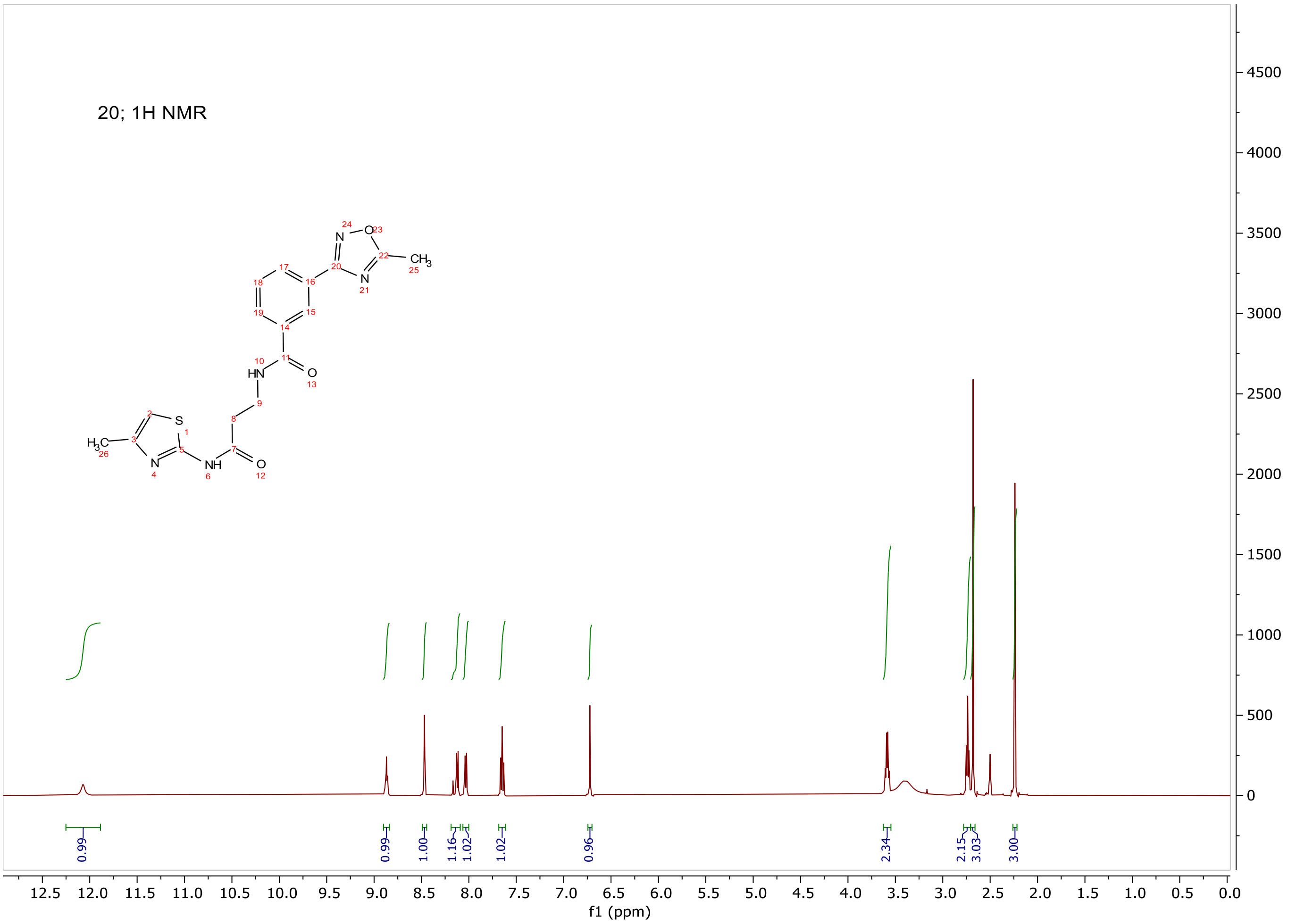
20; 1H NMR
0.99
0.99
1.00
1.16
1.02
1.02
0.96
2.34
2.15
3.03
3.00
f1 (ppm)

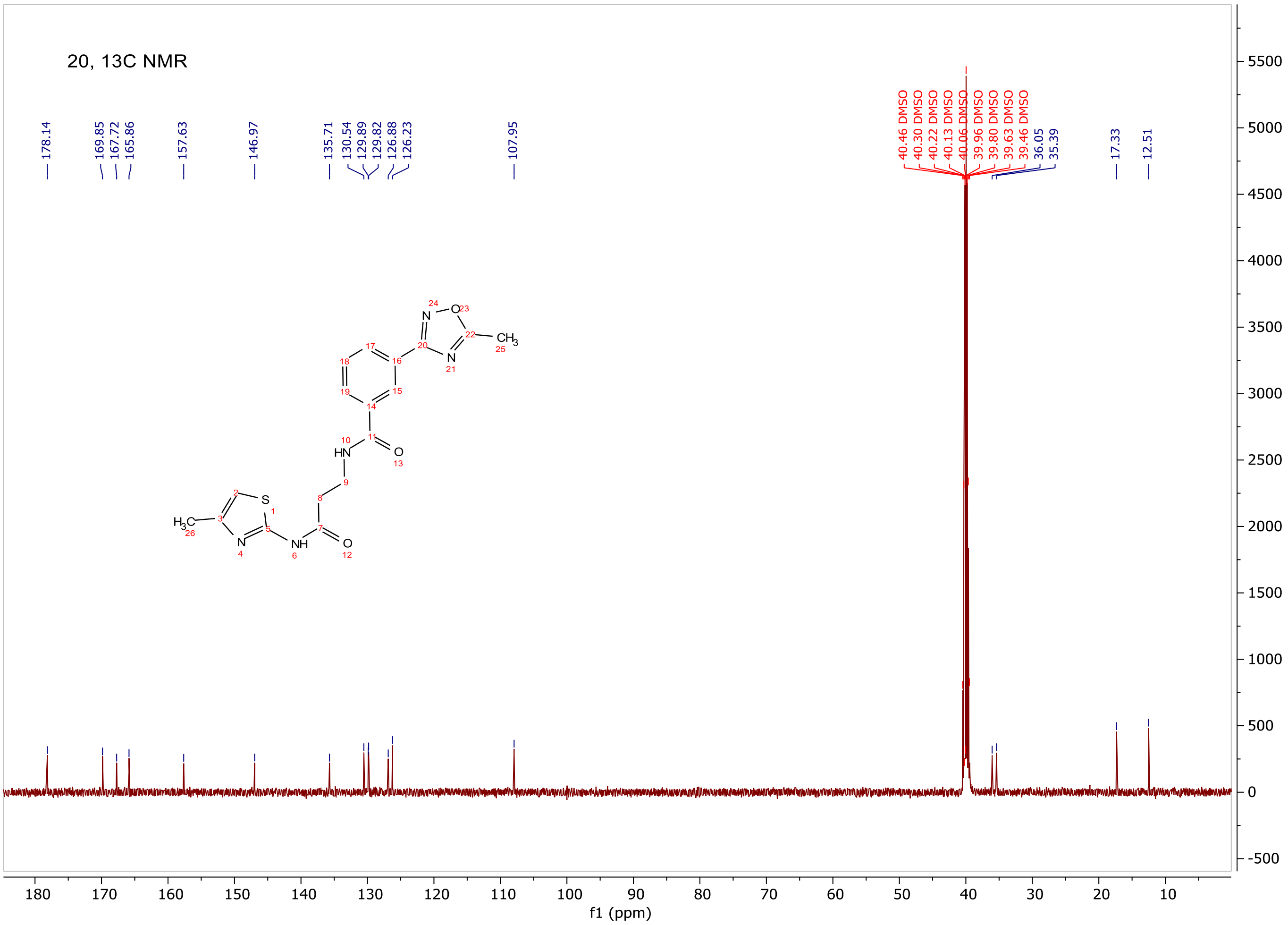

20, 13C NMR
178.14
169.85
167.72
165.86
157.63
146.97
135.71
130.54
129.89
129.82
126.88
126.23
107.95
40.46 DMSO
40.30 DMSO
40.22 DMSO
40.13 DMSO
40.06 DMSO
39.96 DMSO
39.80 DMSO
39.63 DMSO
39.46 DMSO
36.05
35.39
17.33
12.51
f1 (ppm)

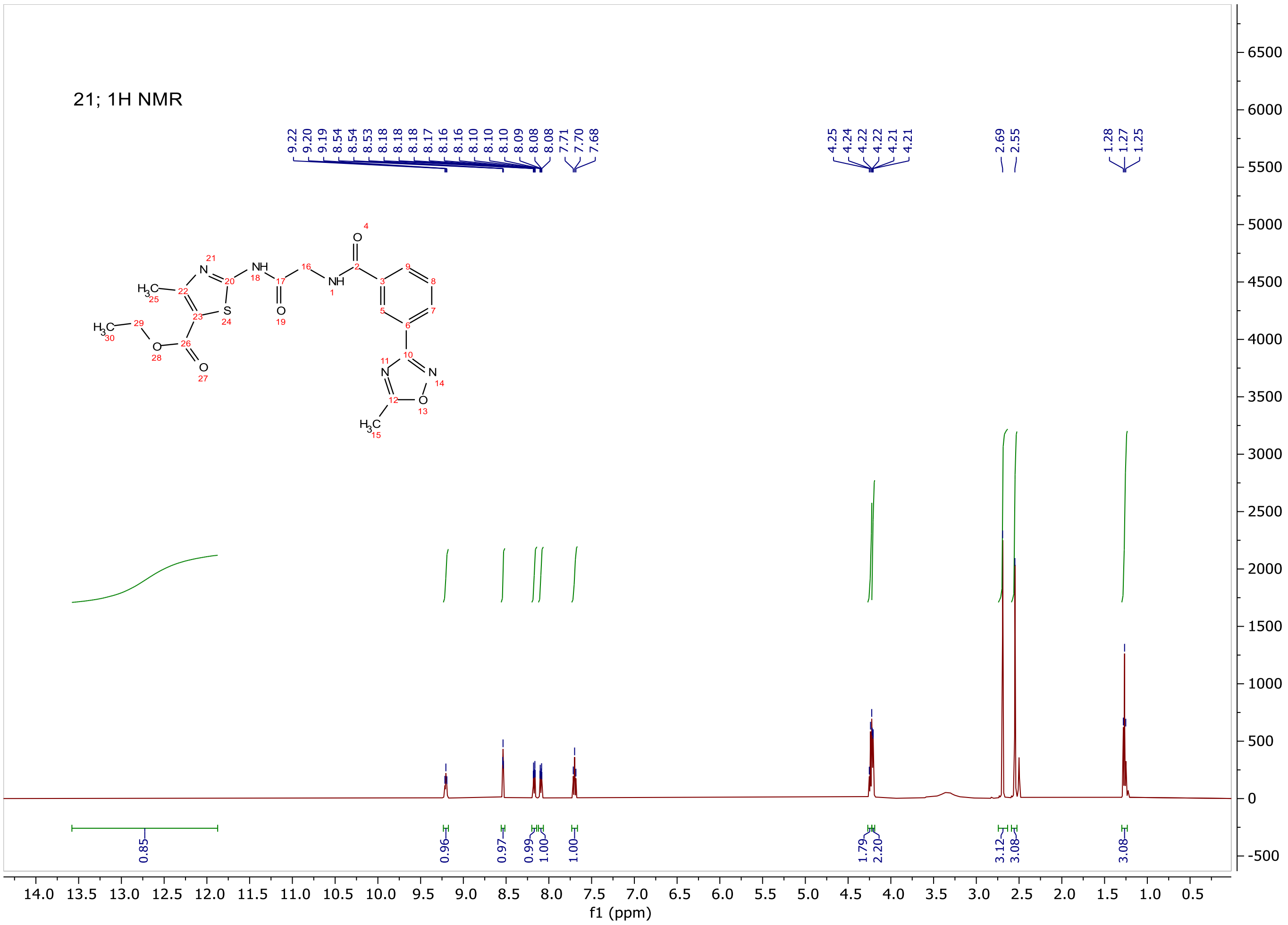

21; 1H NMR
9.22
9.20
9.19
8.54
8.54
8.53
8.18
8.18
8.18
8.17
8.16
8.16
8.10
8.10
8.09
8.08
8.08
7.71
7.70
7.68
4.25
4.24
4.22
4.22
4.21
4.21
2.69
2.55
1.28
1.27
1.25
0.85
0.96
0.97
0.99
1.00
1.00
1.79
2.20
3.12
3.08
3.08
f1 (ppm)

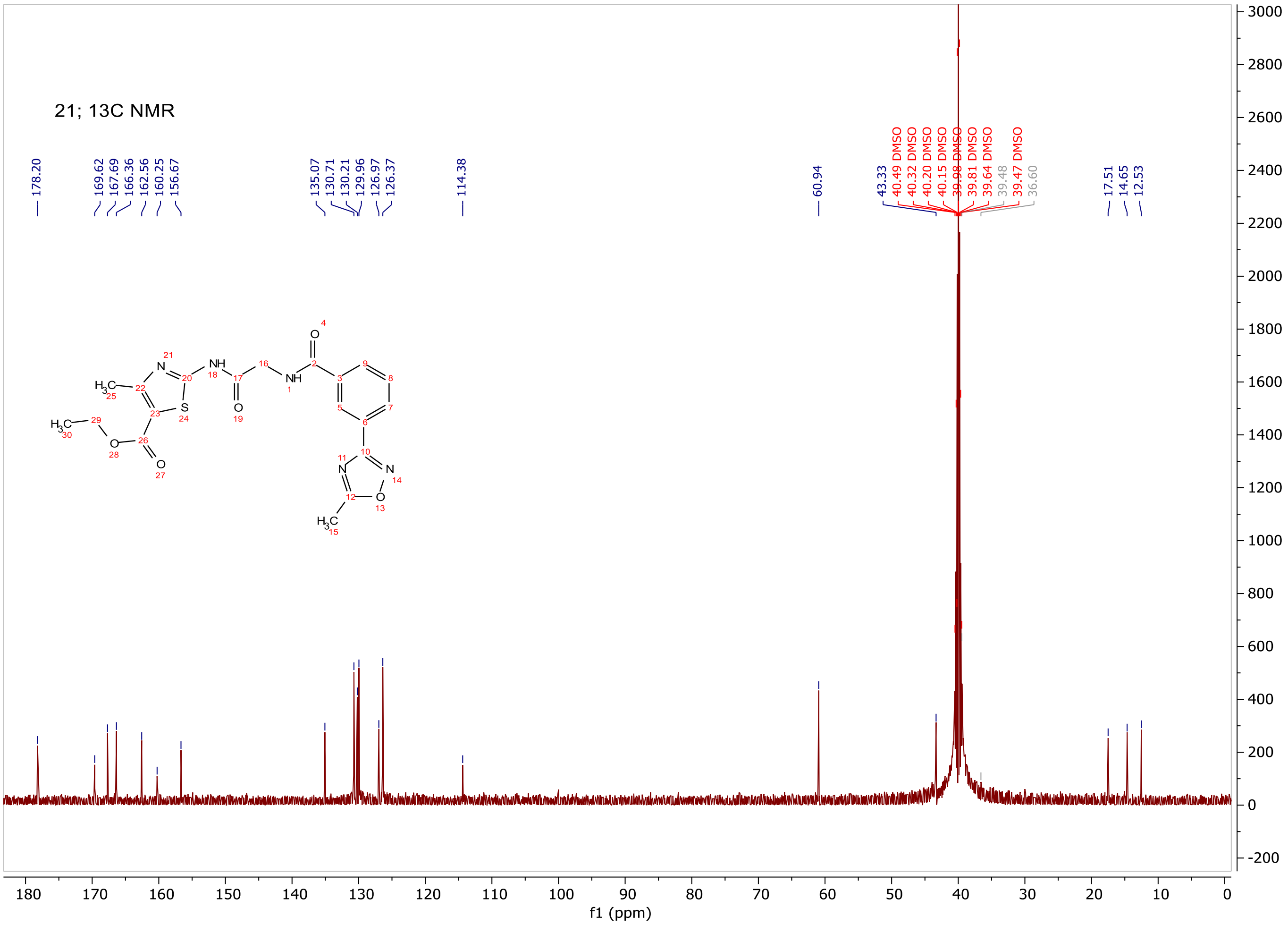
21; 13C NMR
178.20
169.62
167.69
166.36
162.56
160.25
156.67
135.07
130.71
130.21
129.96
126.97
126.37
114.38
60.94
43.33
40.49 DMSO
40.32 DMSO
40.20 DMSO
40.15 DMSO
39.98 DMSO
39.81 DMSO
39.64 DMSO
39.48
39.47 DMSO
36.60
17.51
14.65
12.53
f1 (ppm)

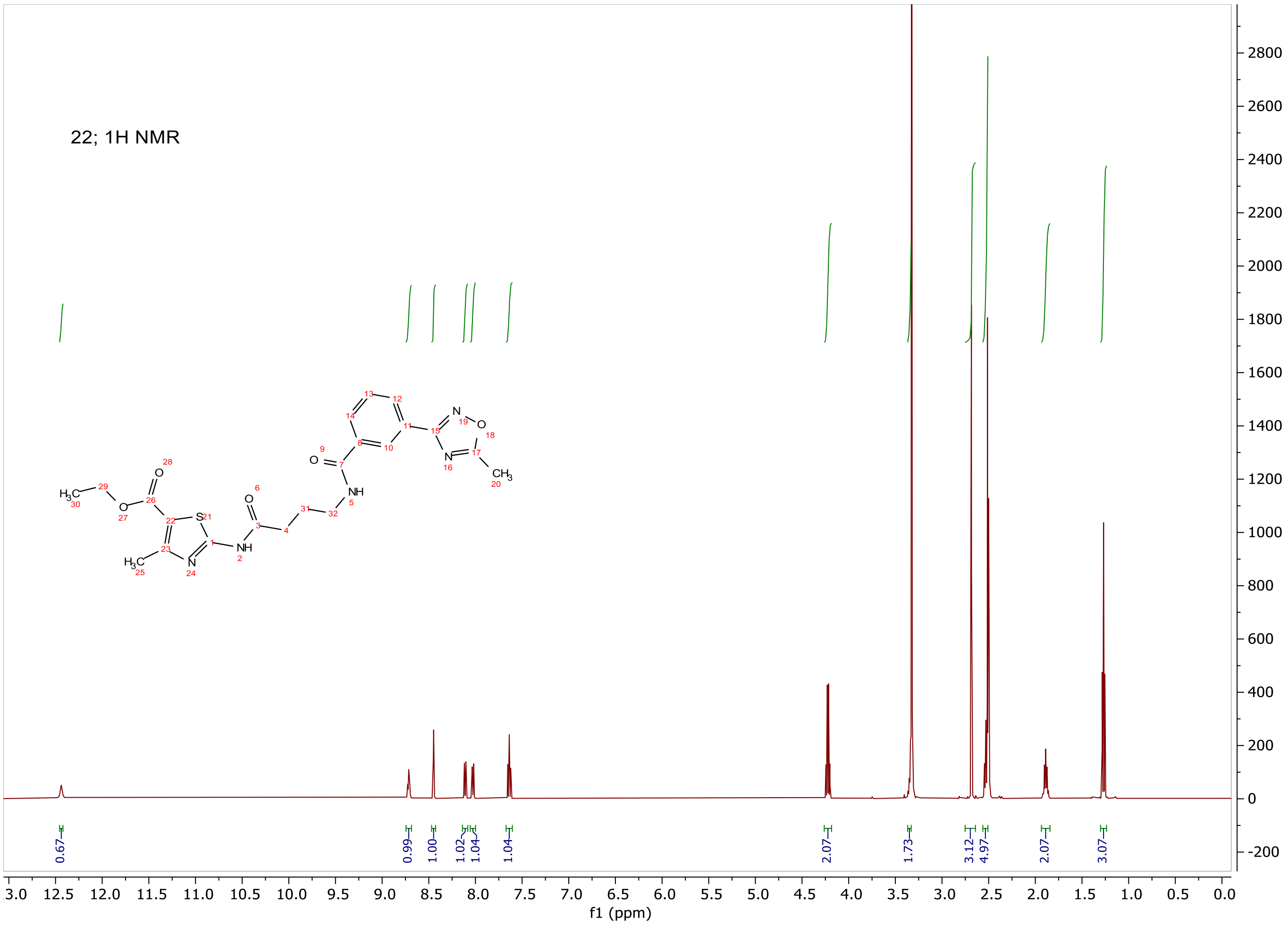
22; 1H NMR
0.67
0.99
1.00
1.02
1.04
1.04
2.07
1.73
3.12
4.97
2.07
3.07
f1 (ppm)

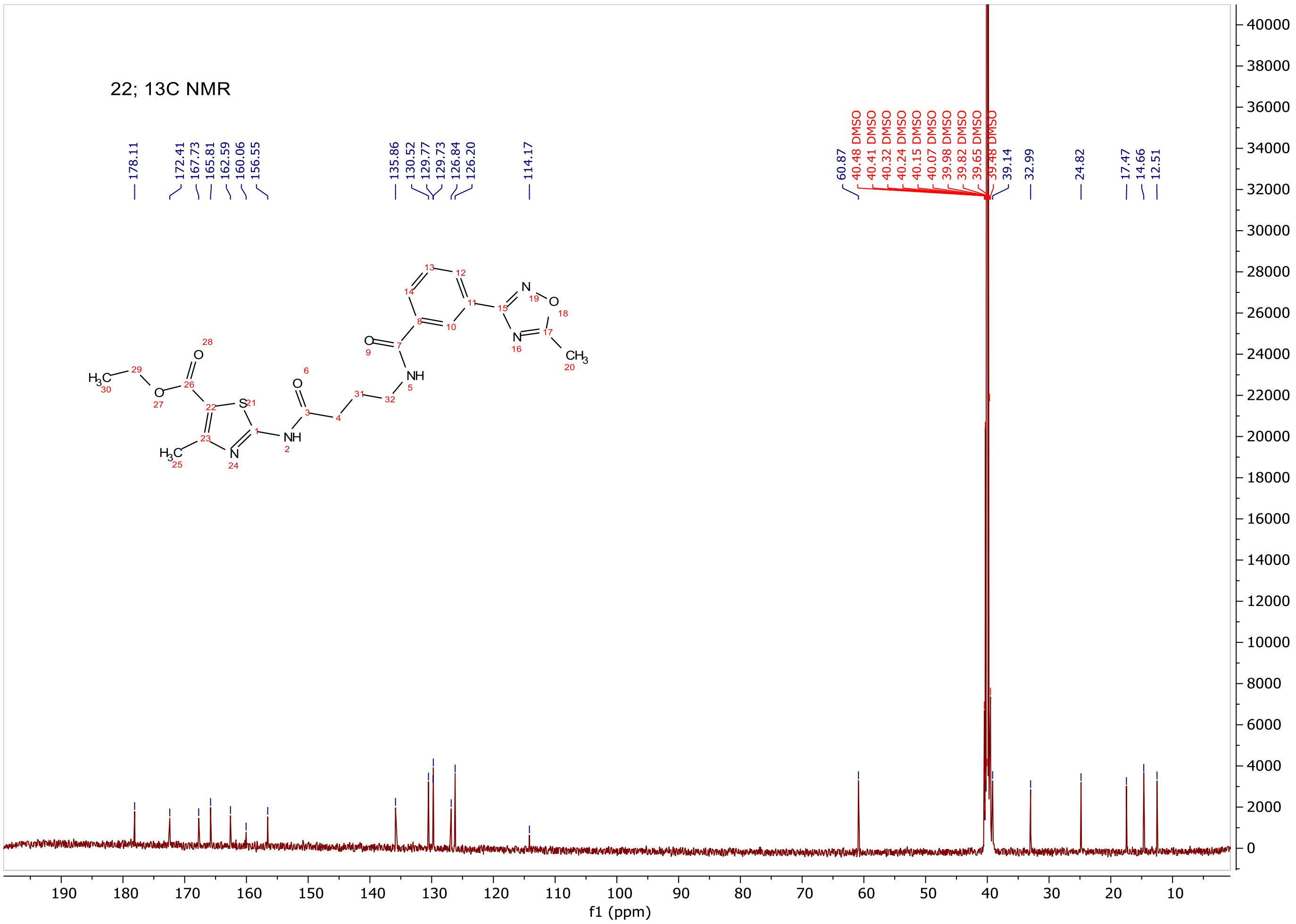
22; 13C NMR
178.11
172.41
167.73
165.81
162.59
160.06
156.55
135.86
130.52
129.77
129.73
126.84
126.20
114.17
60.87
40.48 DMSO
40.41 DMSO
40.32 DMSO
40.24 DMSO
40.15 DMSO
40.07 DMSO
39.98 DMSO
39.82 DMSO
39.65 DMSO
39.48 DMSO
39.14
32.99
24.82
17.47
14.66
12.51
f1 (ppm)

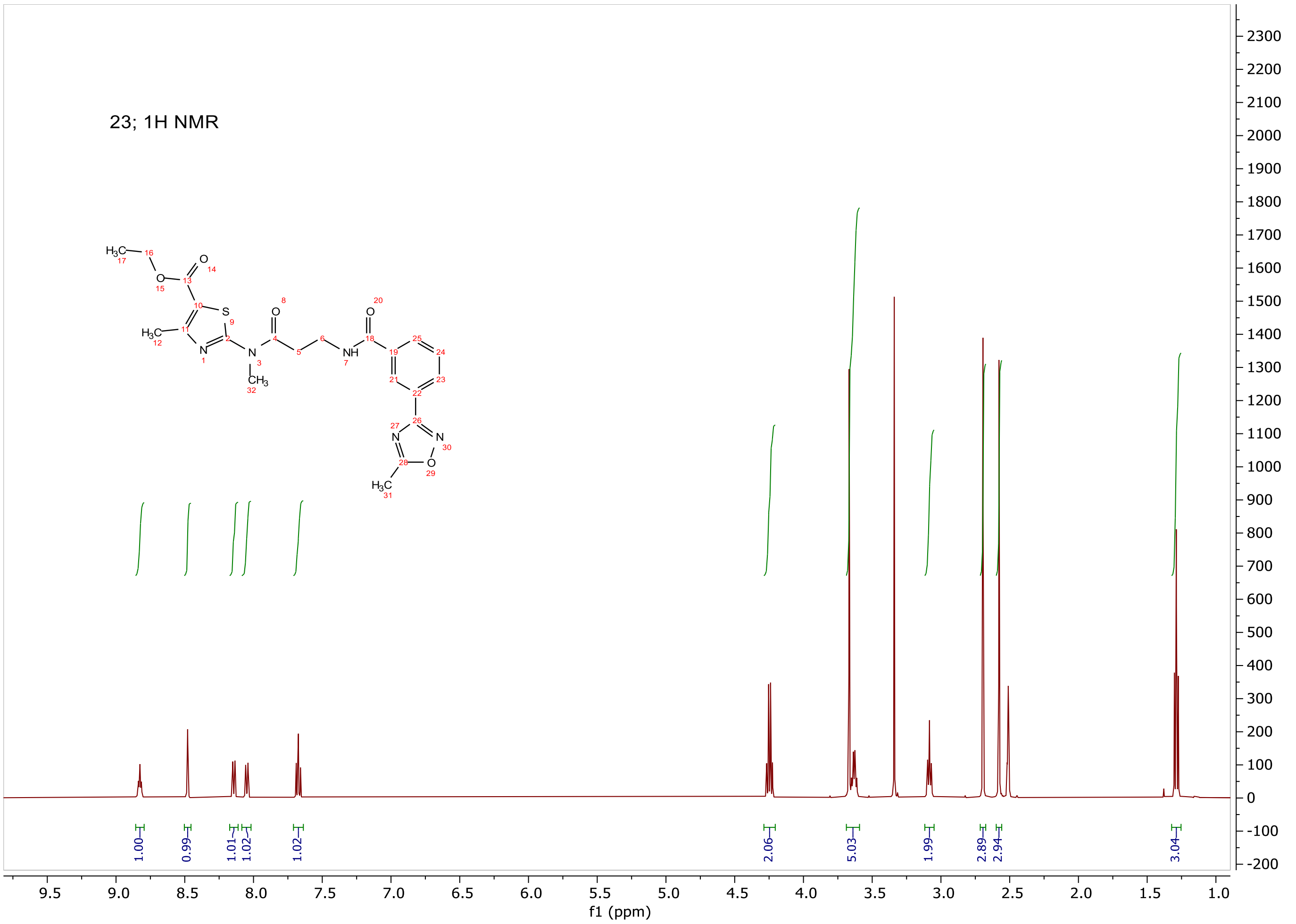
23; 1H NMR
1.00
0.99
1.01
1.02
1.02
2.06
5.03
1.99
2.89
2.94
3.04
f1 (ppm)

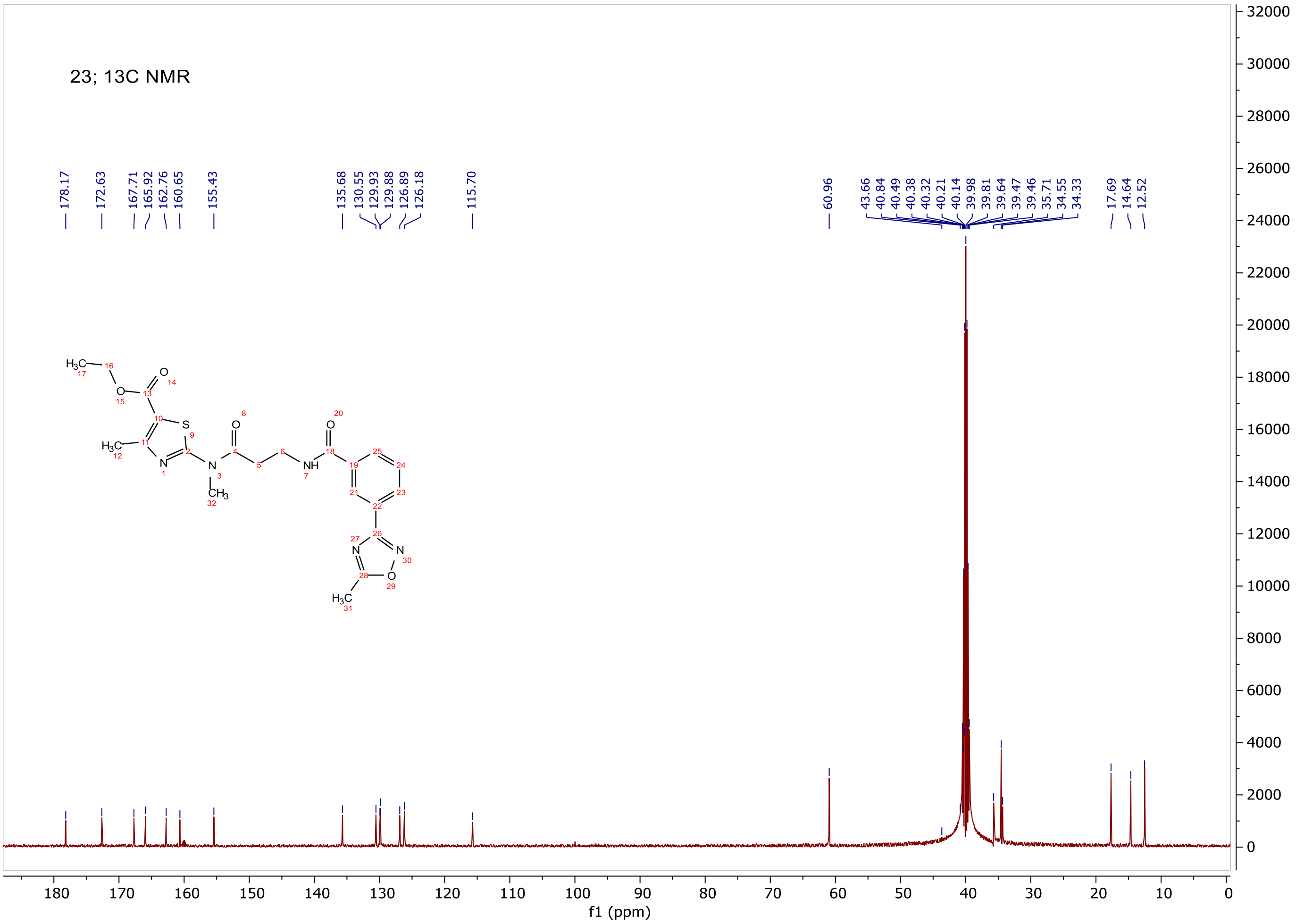
23; 13C NMR
178.17
172.63
167.71
165.92
162.76
160.65
155.43
135.68
130.55
129.93
129.88
126.89
126.18
115.70
60.96
43.66
40.84
40.49
40.38
40.32
40.21
40.14
39.98
39.81
39.64
39.47
39.46
35.71
34.55
34.33
17.69
14.64
12.52
f1 (ppm)

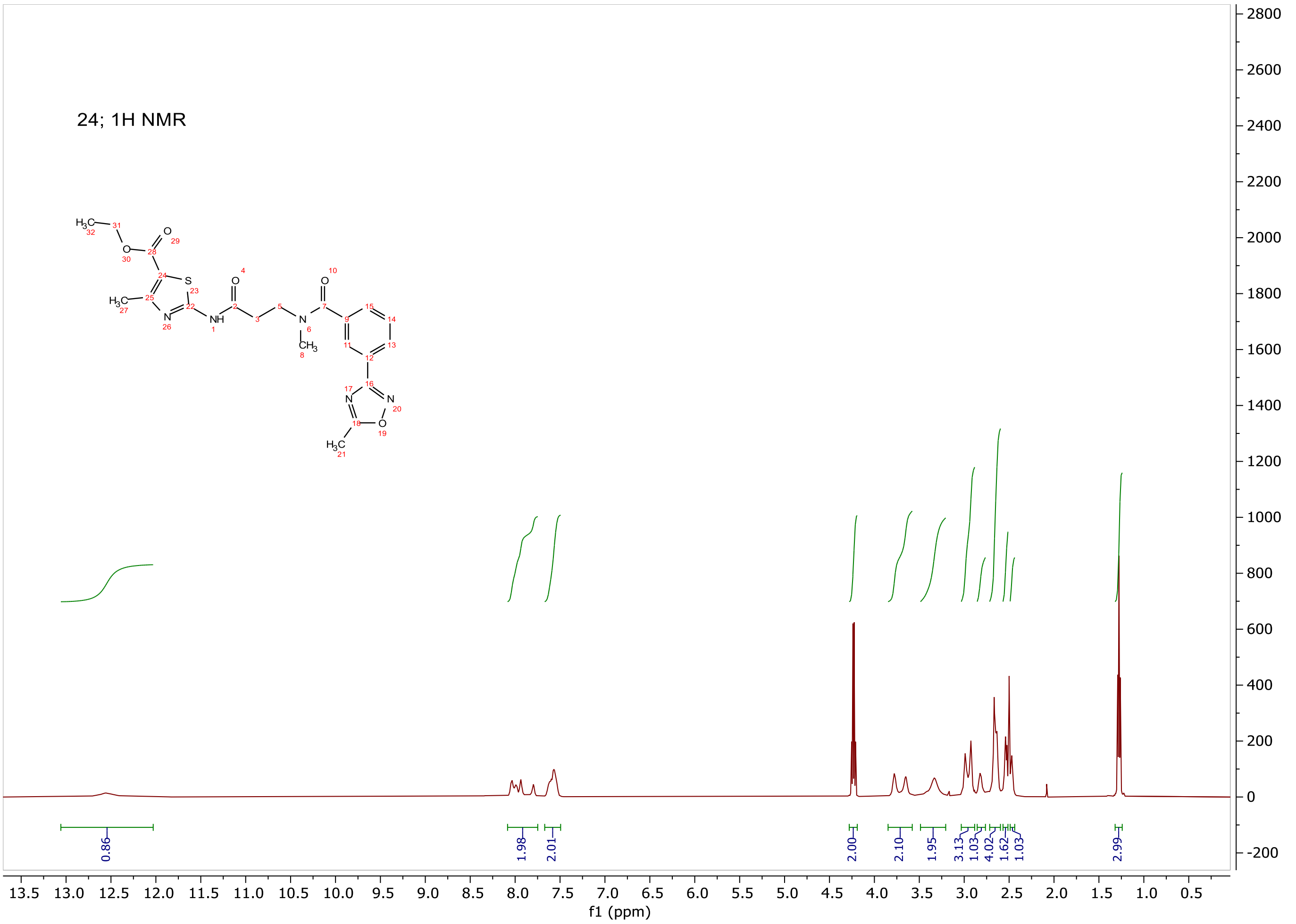
24; 1H NMR
0.86
1.98
2.01
2.00
2.10
1.95
3.13
1.03
4.02
1.62
1.03
2.99
f1 (ppm)

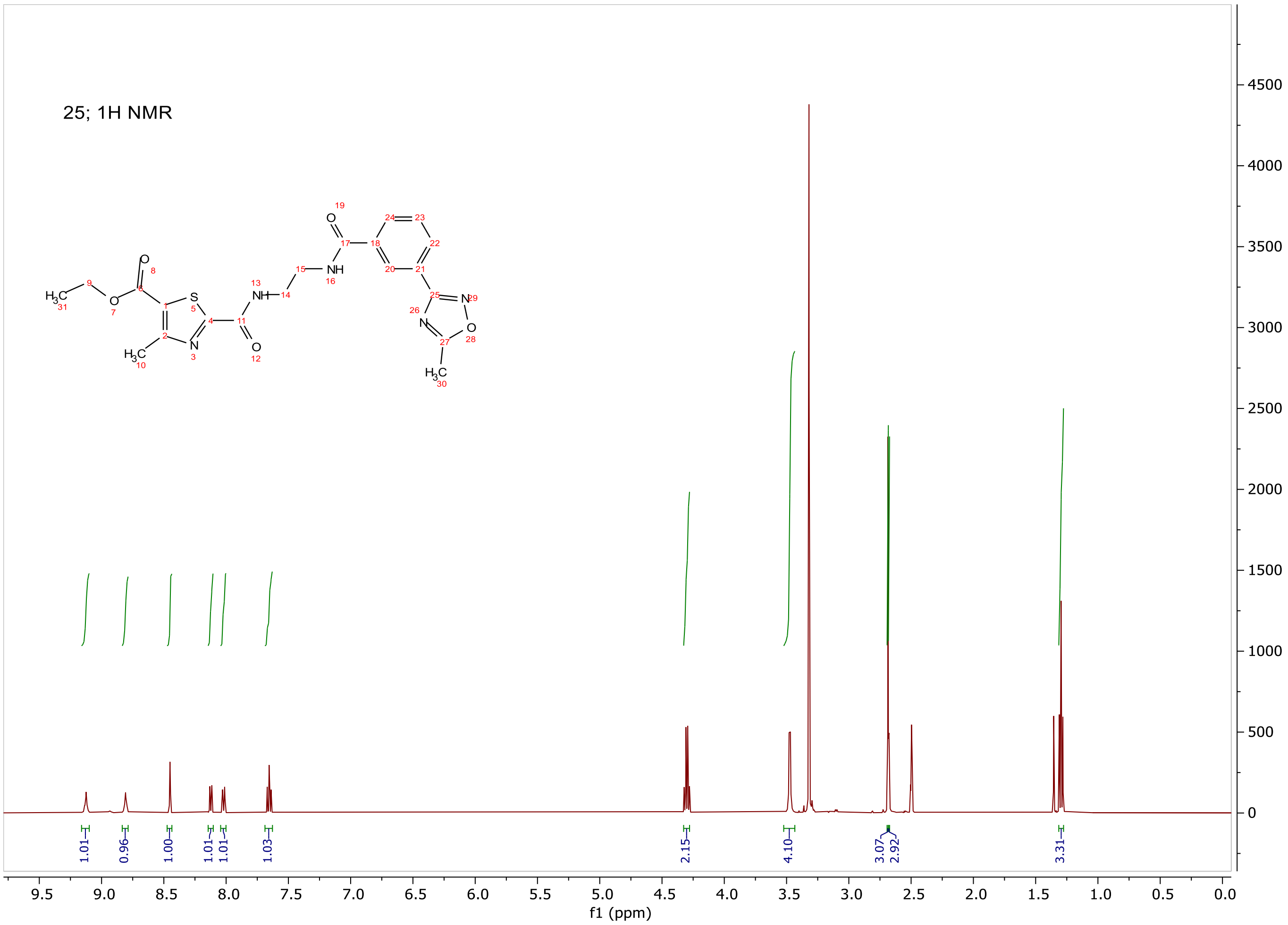
25; 1H NMR
1.01
0.96
1.00
1.01
1.01
1.03
2.15
4.10
3.07
2.92
3.31
f1 (ppm)

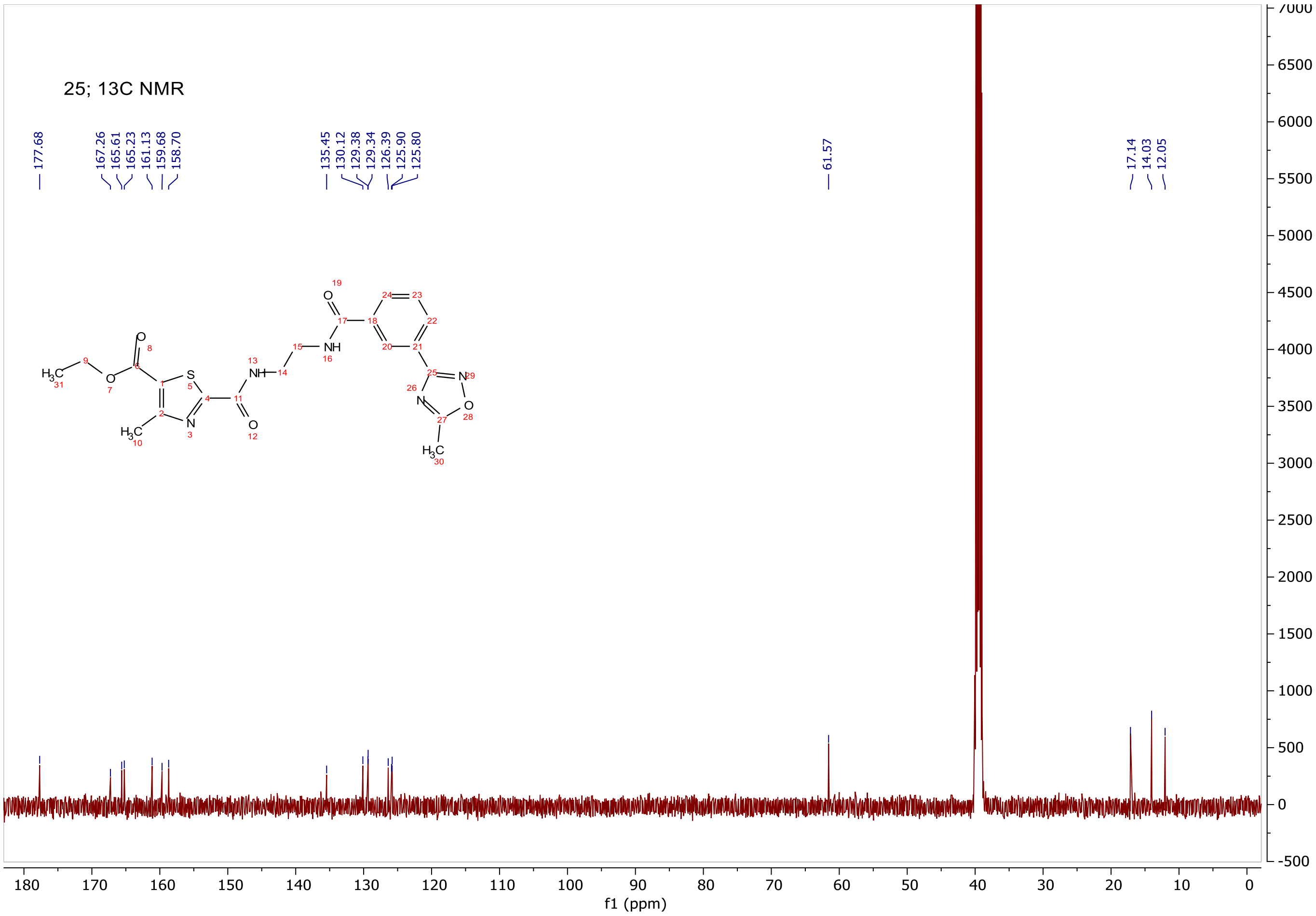
25; 13C NMR
177.68
167.26
165.61
165.23
161.13
159.68
158.70
135.45
130.12
129.38
129.34
126.39
125.90
125.80
61.57
17.14
14.03
12.05
f1 (ppm)

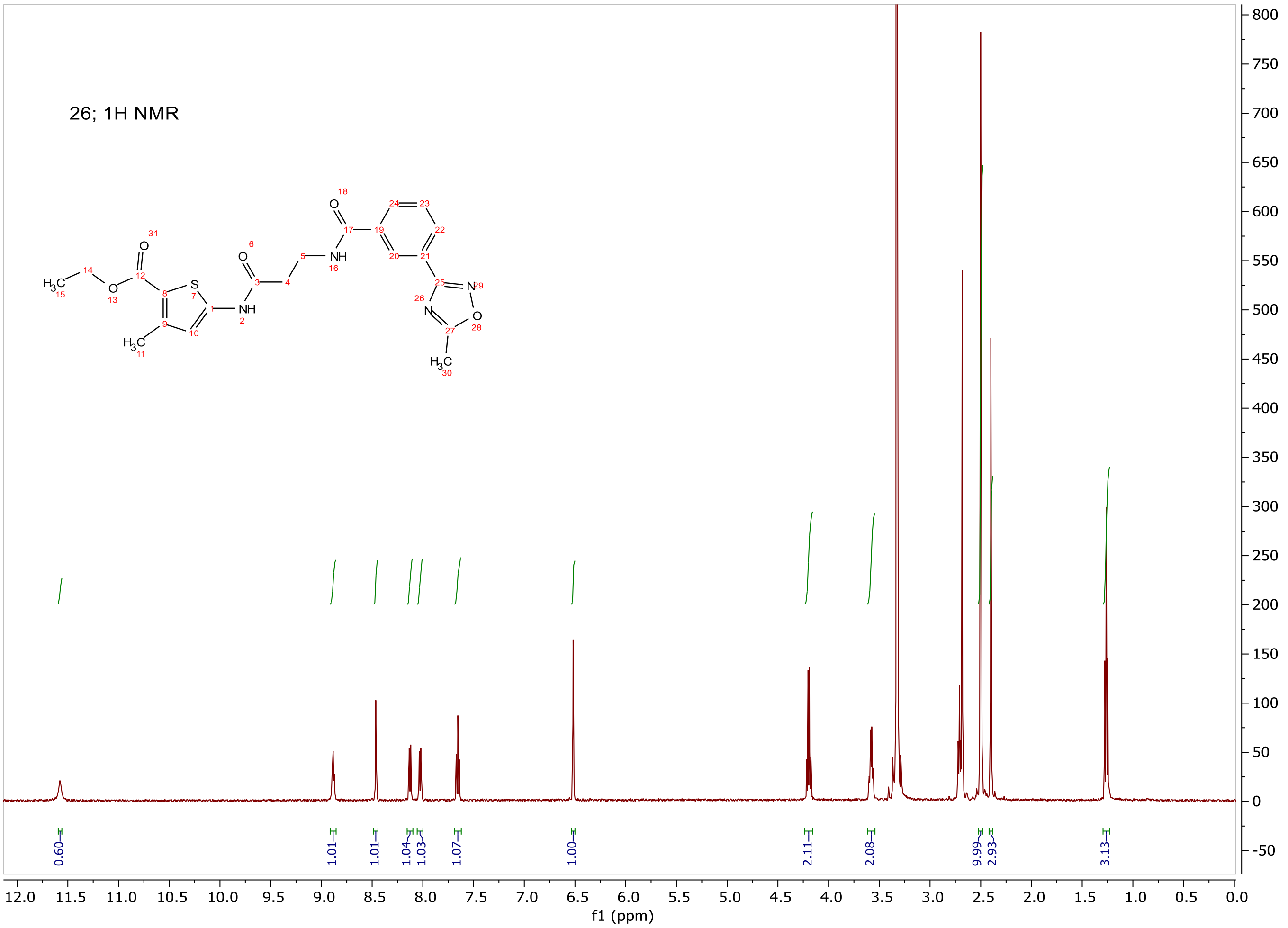
26; 1H NMR
0.60
1.01
1.01
1.04
1.03
1.07
1.00
2.11
2.08
9.99
2.93
3.13
f1 (ppm)

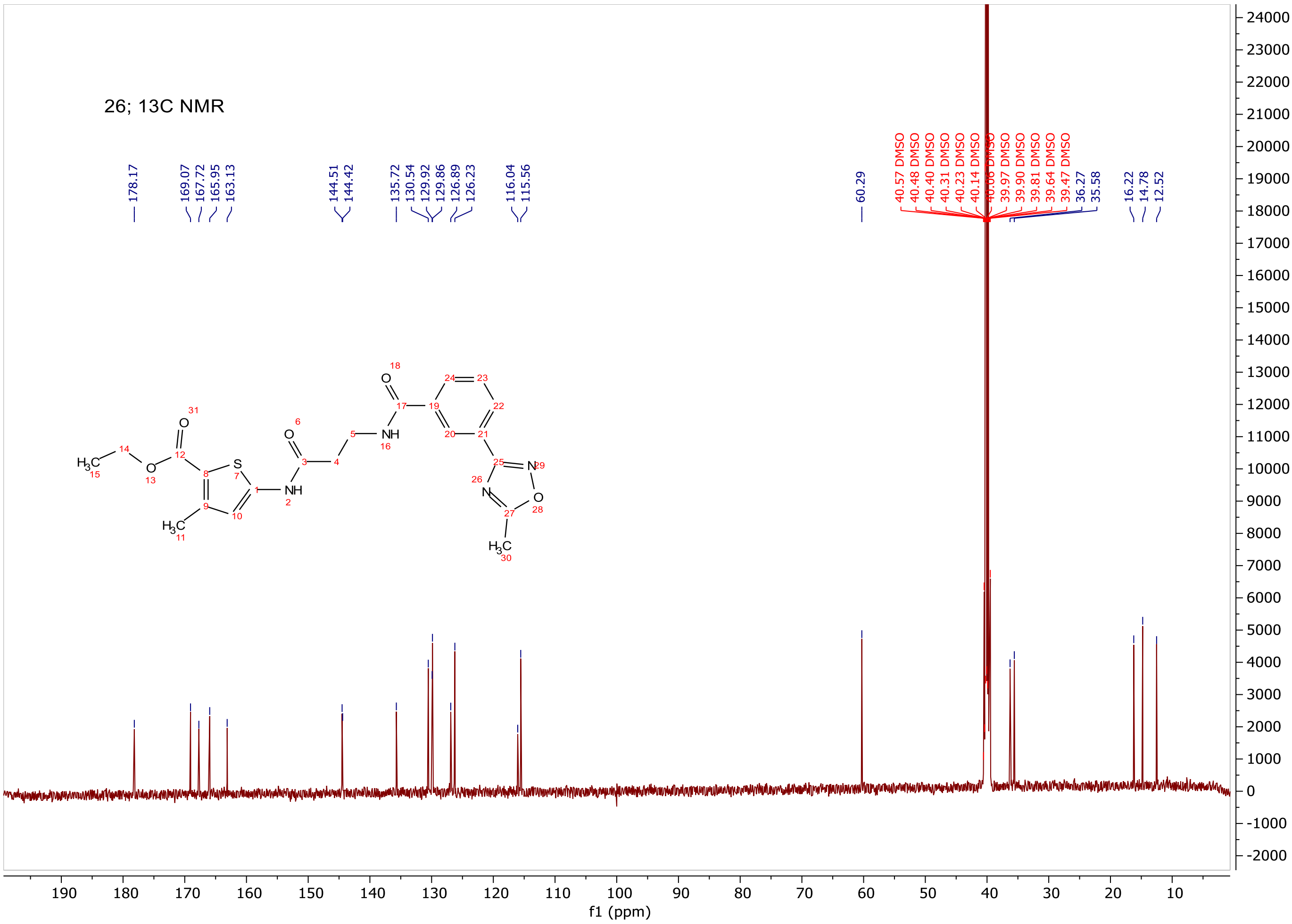
26; 13C NMR
178.17
169.07
167.72
165.95
163.13
144.51
144.42
135.72
130.54
129.92
129.86
126.89
126.23
116.04
115.56
60.29
40.57 DMSO
40.48 DMSO
40.40 DMSO
40.31 DMSO
40.23 DMSO
40.14 DMSO
40.06 DMSO
39.97 DMSO
39.90 DMSO
39.81 DMSO
39.64 DMSO
39.47 DMSO
36.27
35.58
16.22
14.78
12.52
f1 (ppm)

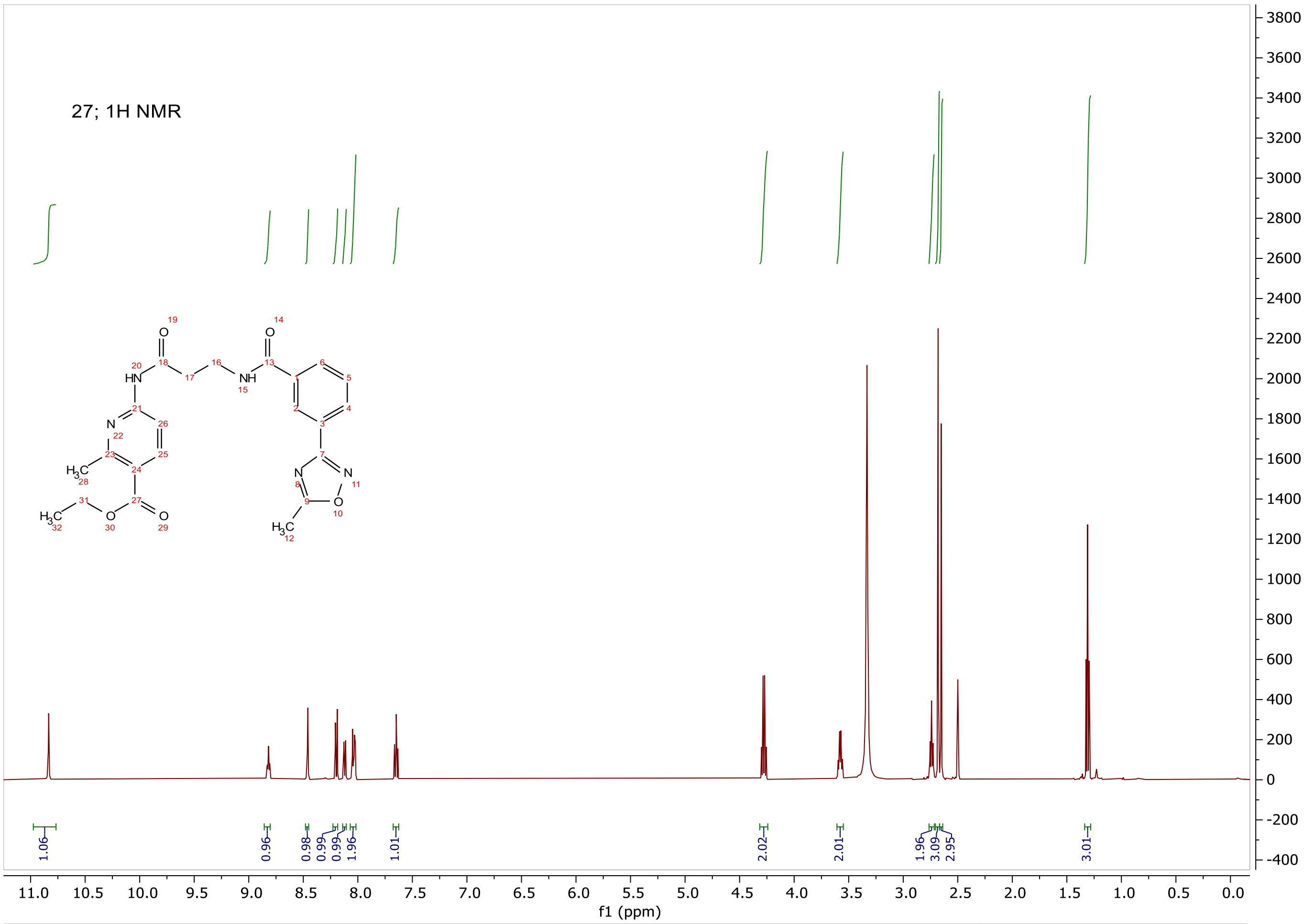
27; 1H NMR
1.06
0.96
0.98
0.99
0.99
1.96
1.01
2.02
2.01
1.96
3.09
2.95
3.01
f1 (ppm)

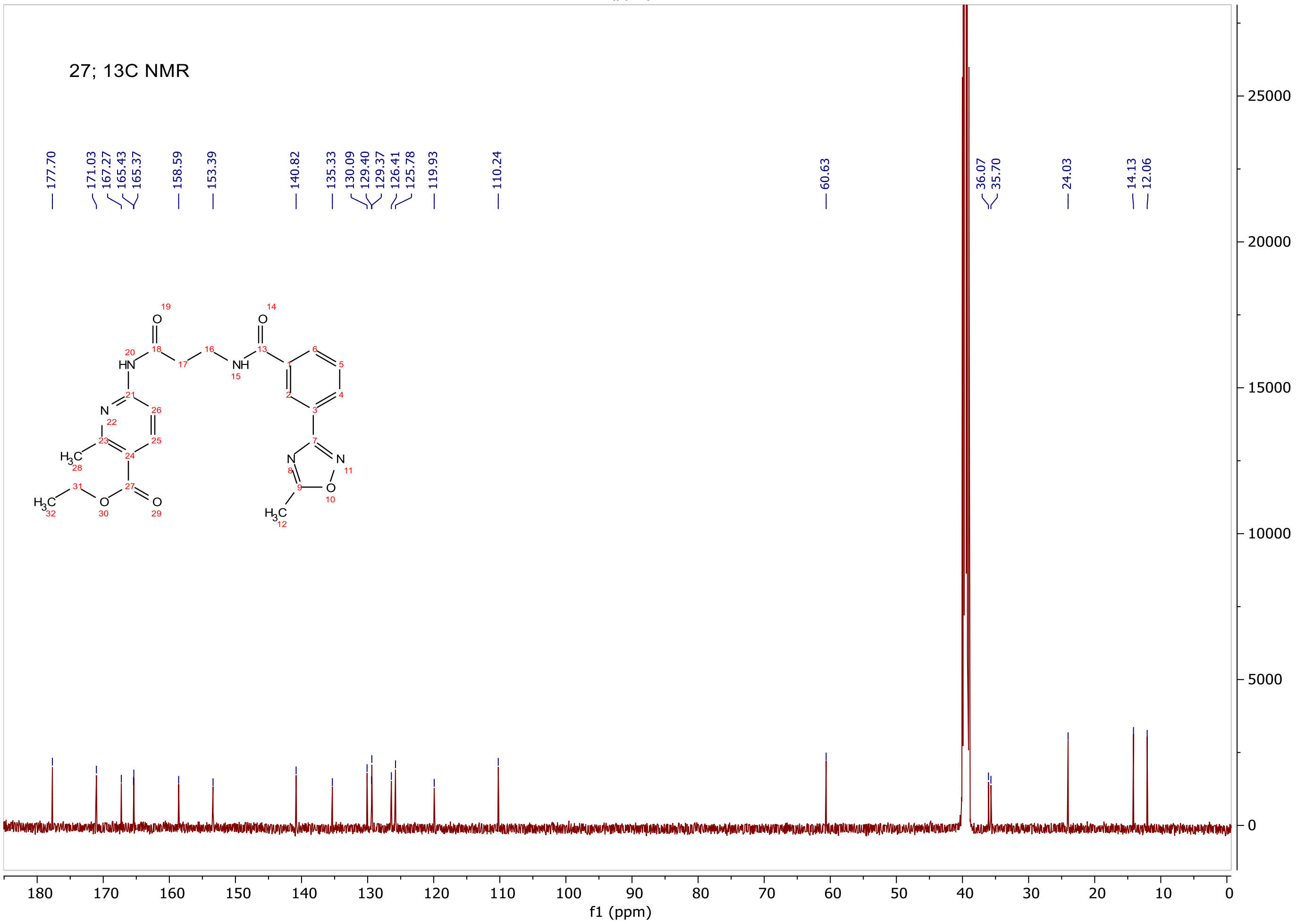
27; 13C NMR
177.70
171.03
167.27
165.43
165.37
158.59
153.39
140.82
135.33
130.09
129.40
129.37
126.41
125.78
119.93
110.24
60.63
36.07
35.70
24.03
14.13
12.06
f1 (ppm)

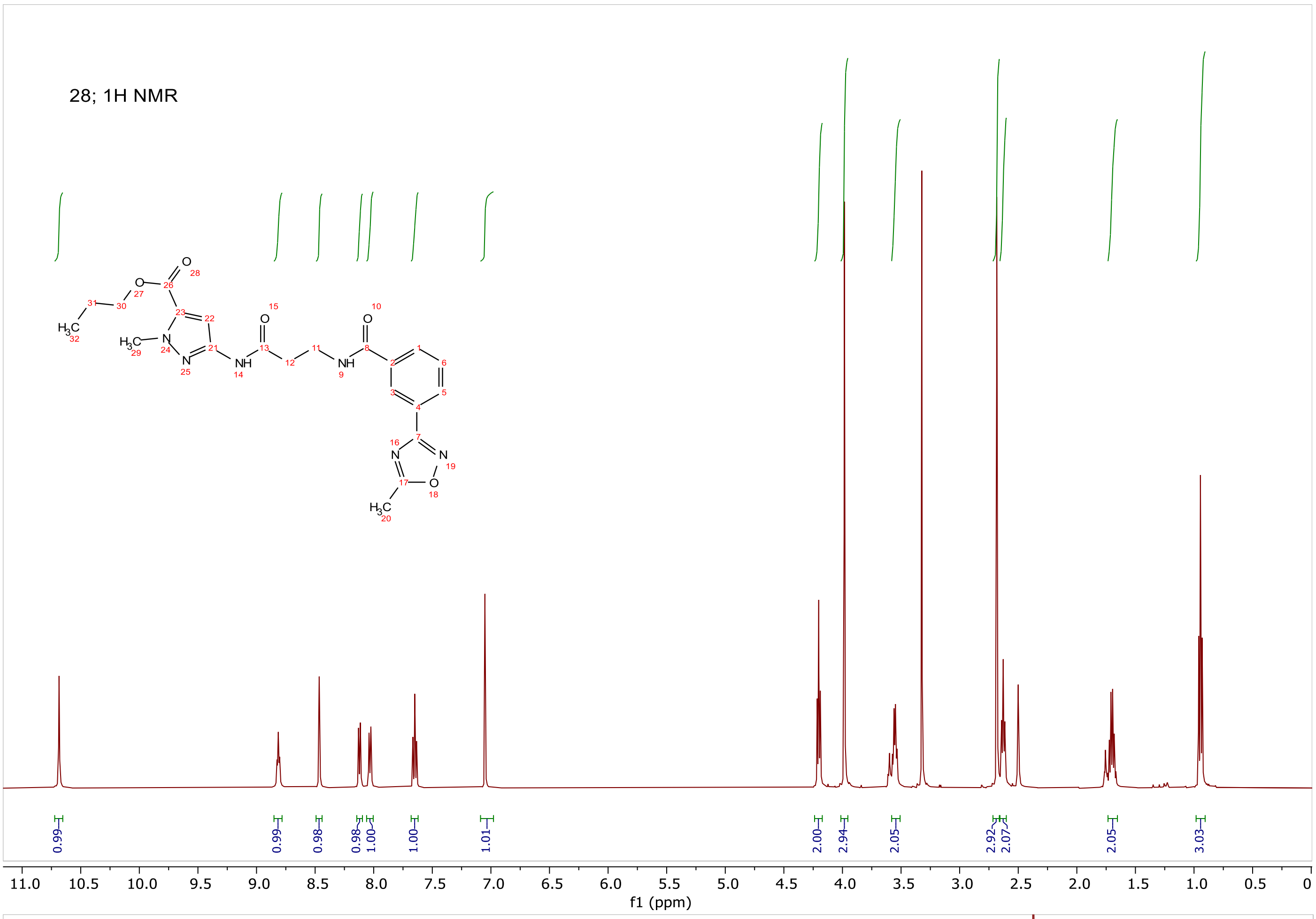
28; 1H NMR
0.99
0.99
0.98
0.98
1.00
1.00
1.01
2.00
2.94
2.05
2.92
2.07
2.05
3.03
f1 (ppm)

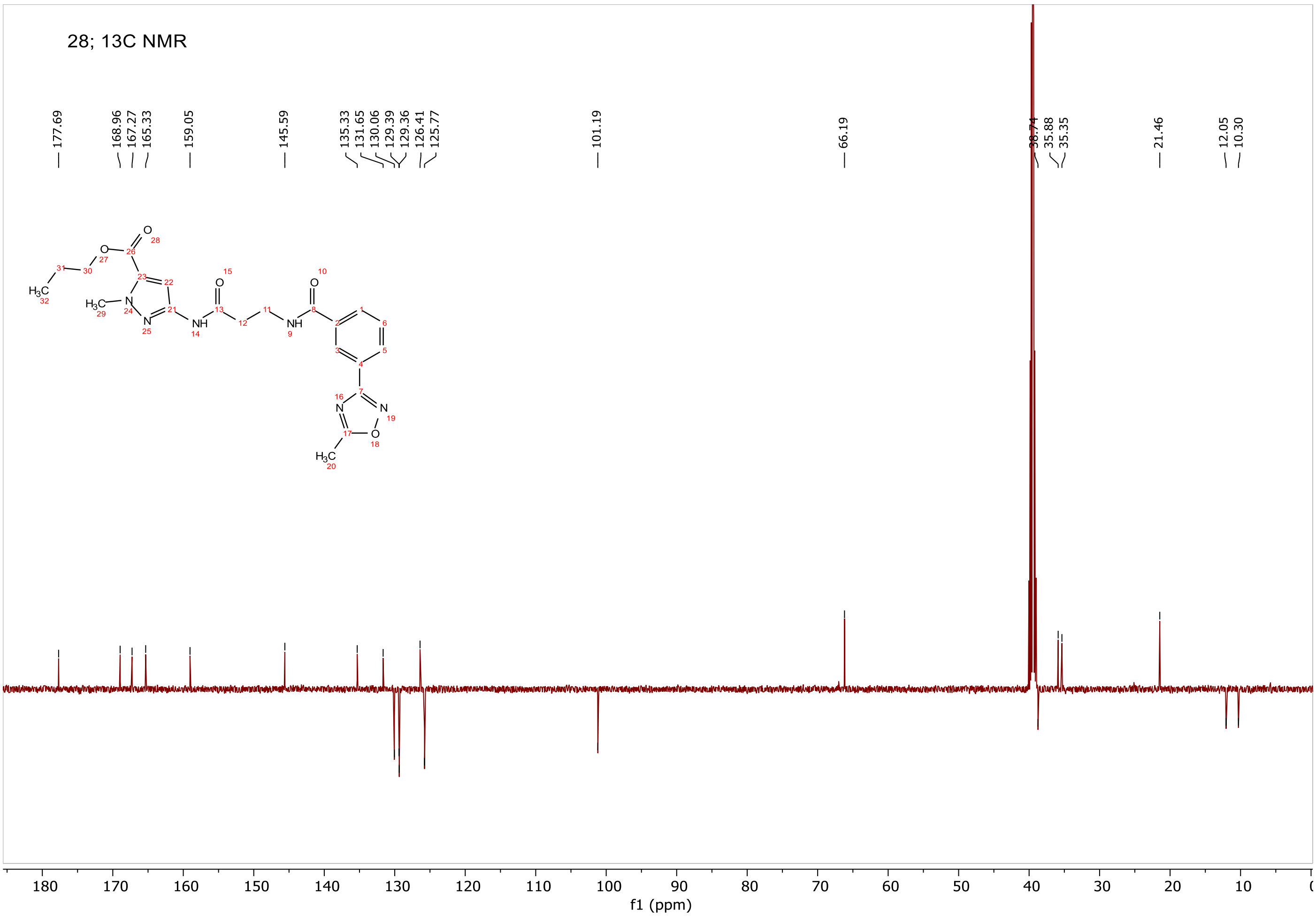
28; 13C NMR
177.69
168.96
167.27
165.33
159.05
145.59
135.33
131.65
130.06
129.39
129.36
126.41
125.77
101.19
66.19
35.88
35.35
21.46
12.05
10.30
f1 (ppm)

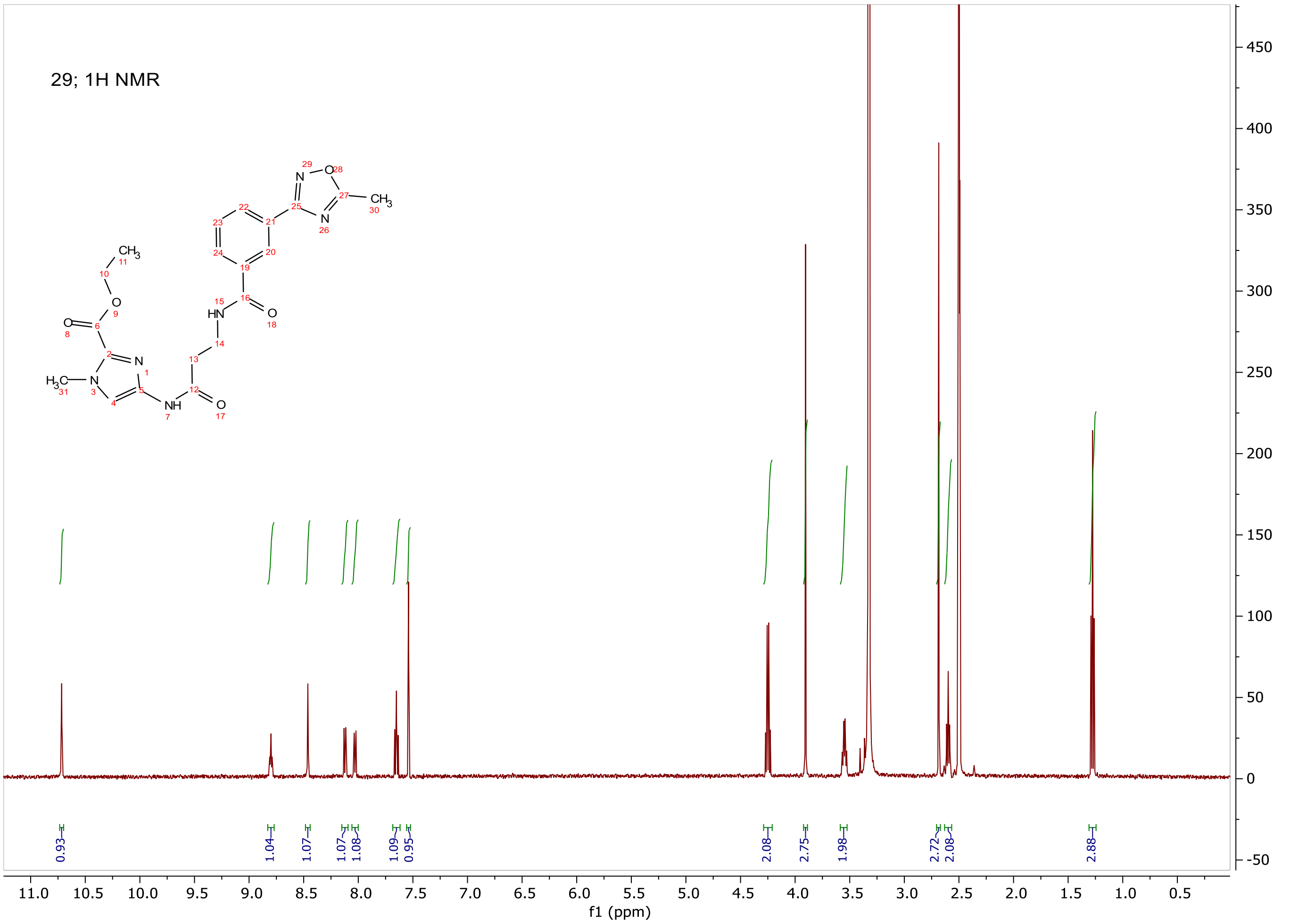
29; 1H NMR
0.93
1.04
1.07
1.07
1.08
1.09
0.95
2.08
2.75
1.98
2.72
2.08
2.88
f1 (ppm)

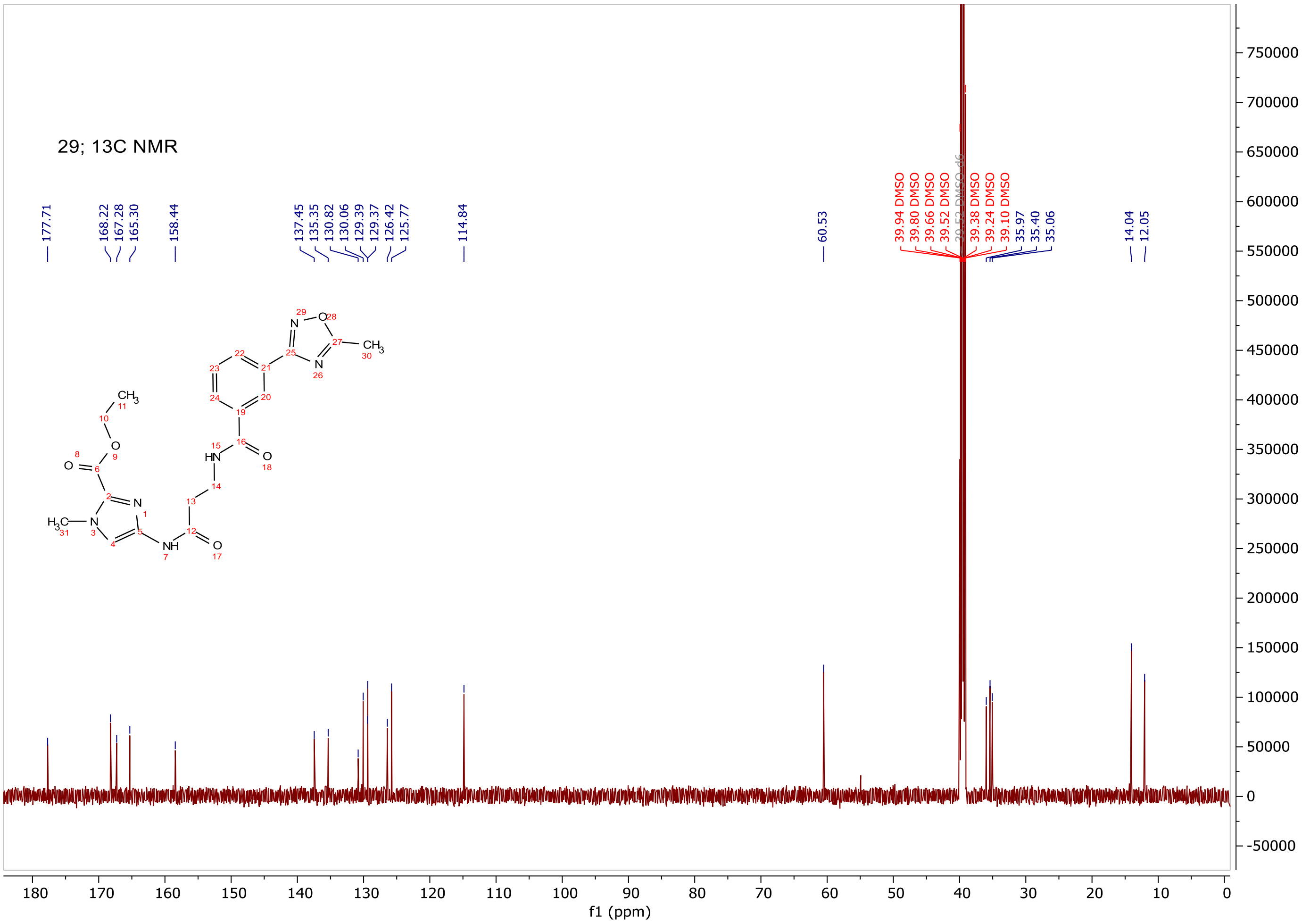
29; 13C NMR
177.71
168.22
167.28
165.30
158.44
137.45
135.35
130.82
130.06
129.39
129.37
126.42
125.77
114.84
60.53
39.94 DMSO
39.80 DMSO
39.66 DMSO
39.52 DMSO
39.38 DMSO
39.24 DMSO
39.10 DMSO
35.97
35.40
35.06
14.04
12.05
f1 (ppm)

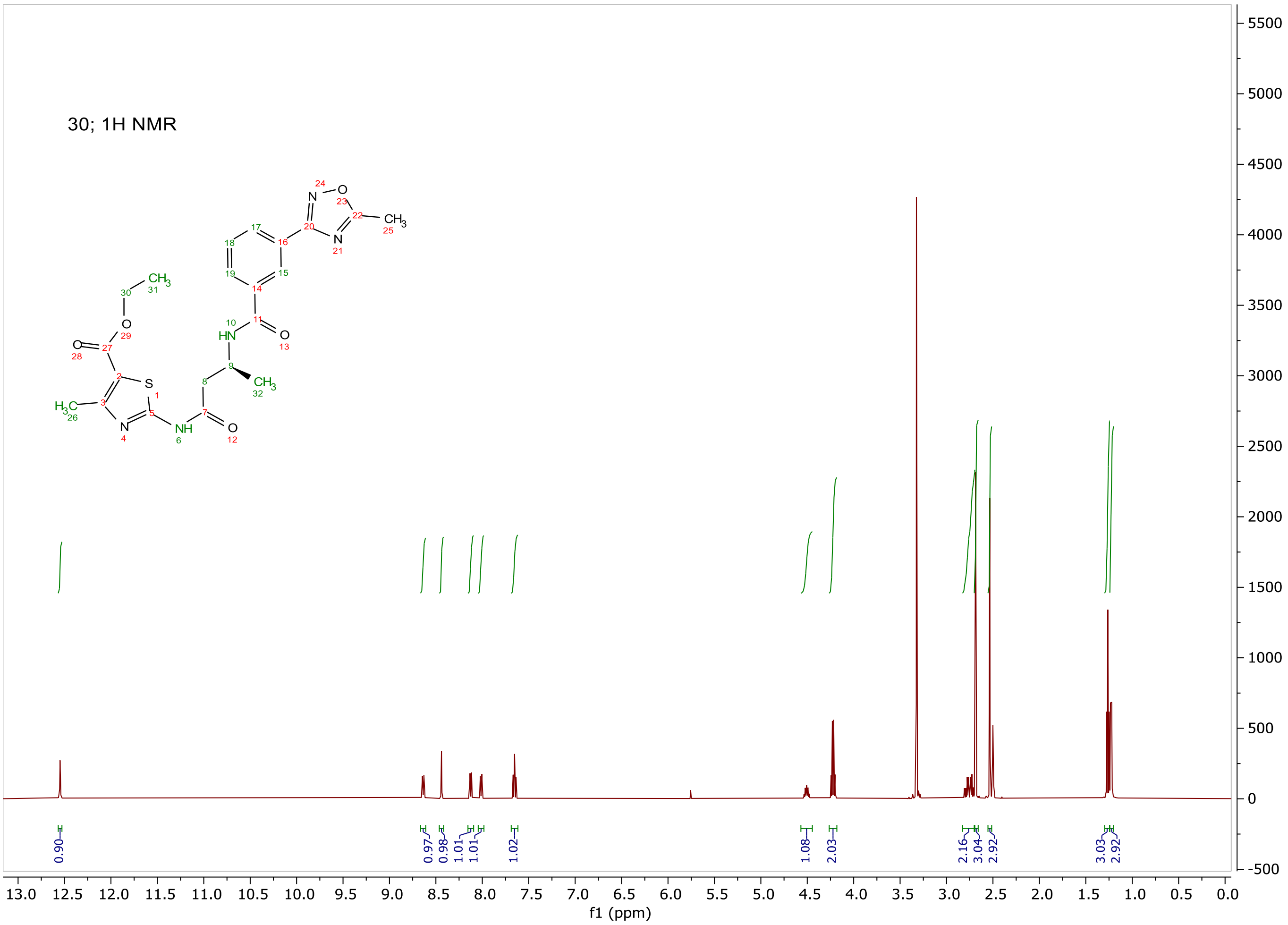

30; 1H NMR
0.90
0.97
0.98
1.01
1.01
1.02
1.08
2.03
2.16
3.04
2.92
3.03
2.92
f1 (ppm)

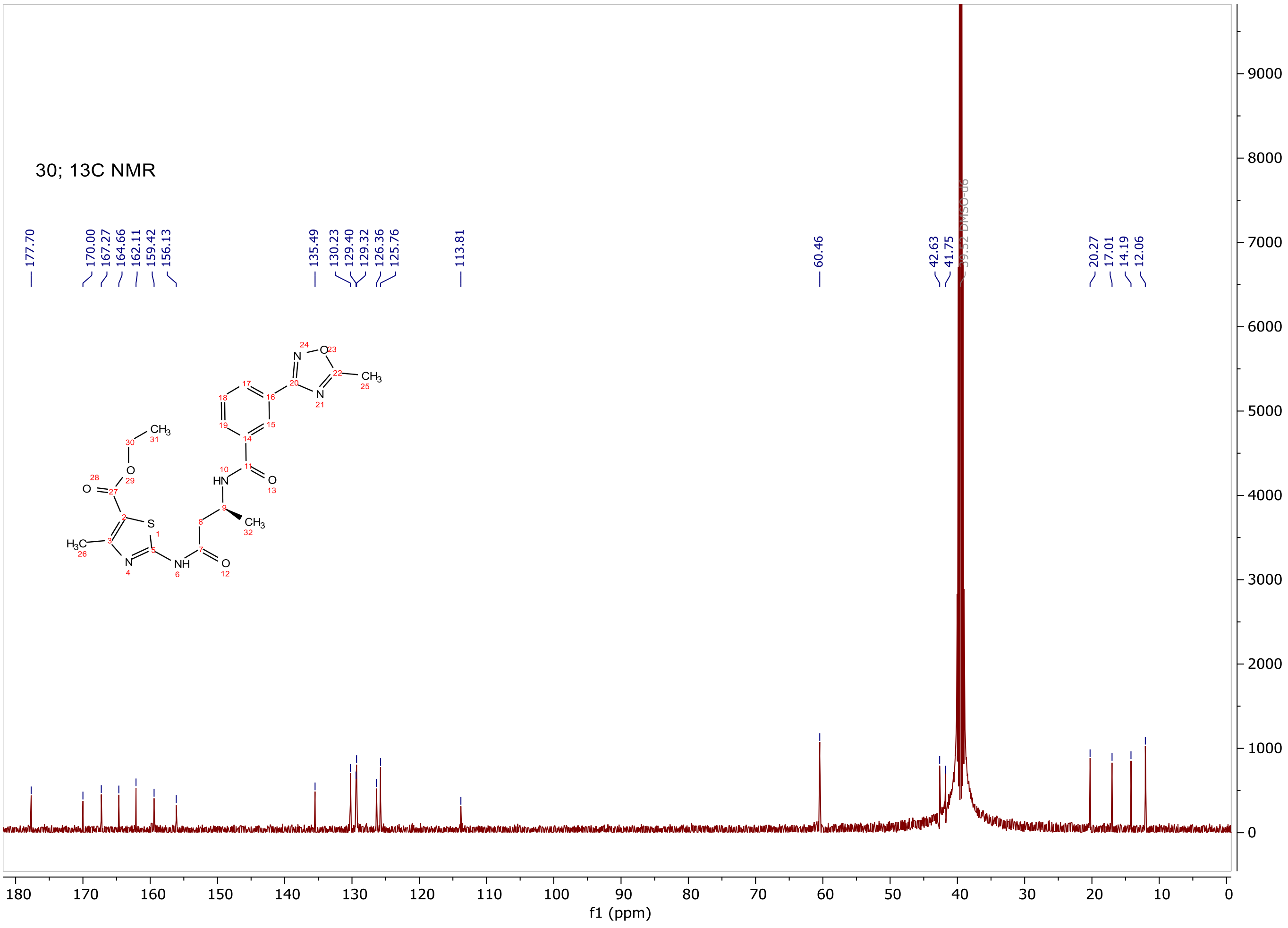

30; 13C NMR
177.70
170.00
167.27
164.66
162.11
159.42
156.13
135.49
130.23
129.40
129.32
126.36
125.76
113.81
60.46
42.63
41.75
39.52 DMSO-d6
20.27
17.01
14.19
12.06
f1 (ppm)

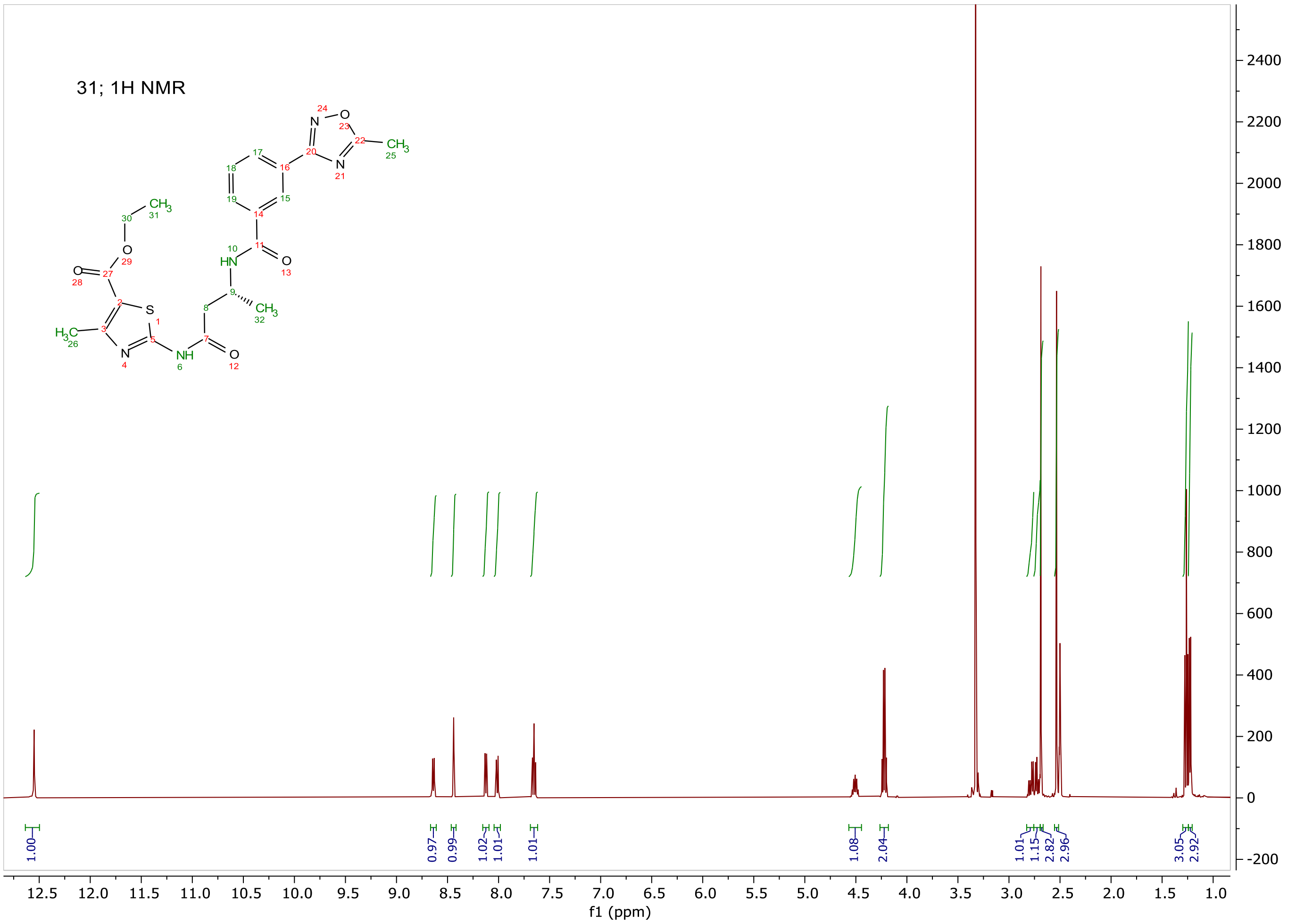
31; 1H NMR
1.00
0.97
0.99
1.02
1.01
1.01
1.08
2.04
1.01
1.15
2.82
2.96
3.05
2.92
f1 (ppm)

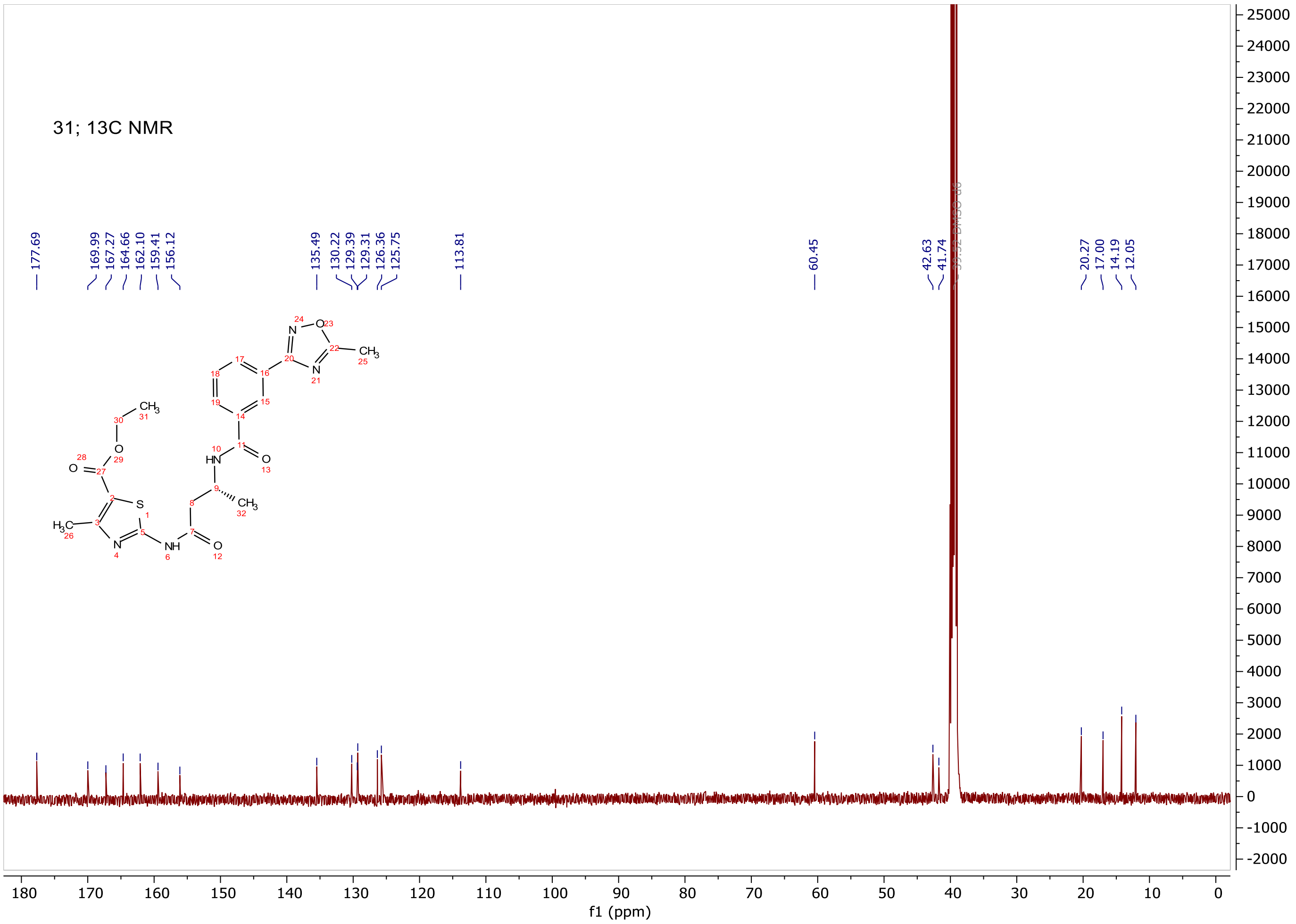
31; 13C NMR
177.69
169.99
167.27
164.66
162.10
159.41
156.12
135.49
130.22
129.39
129.31
126.36
125.75
113.81
60.45
42.63
41.74
20.27
17.00
14.19
12.05
f1 (ppm)

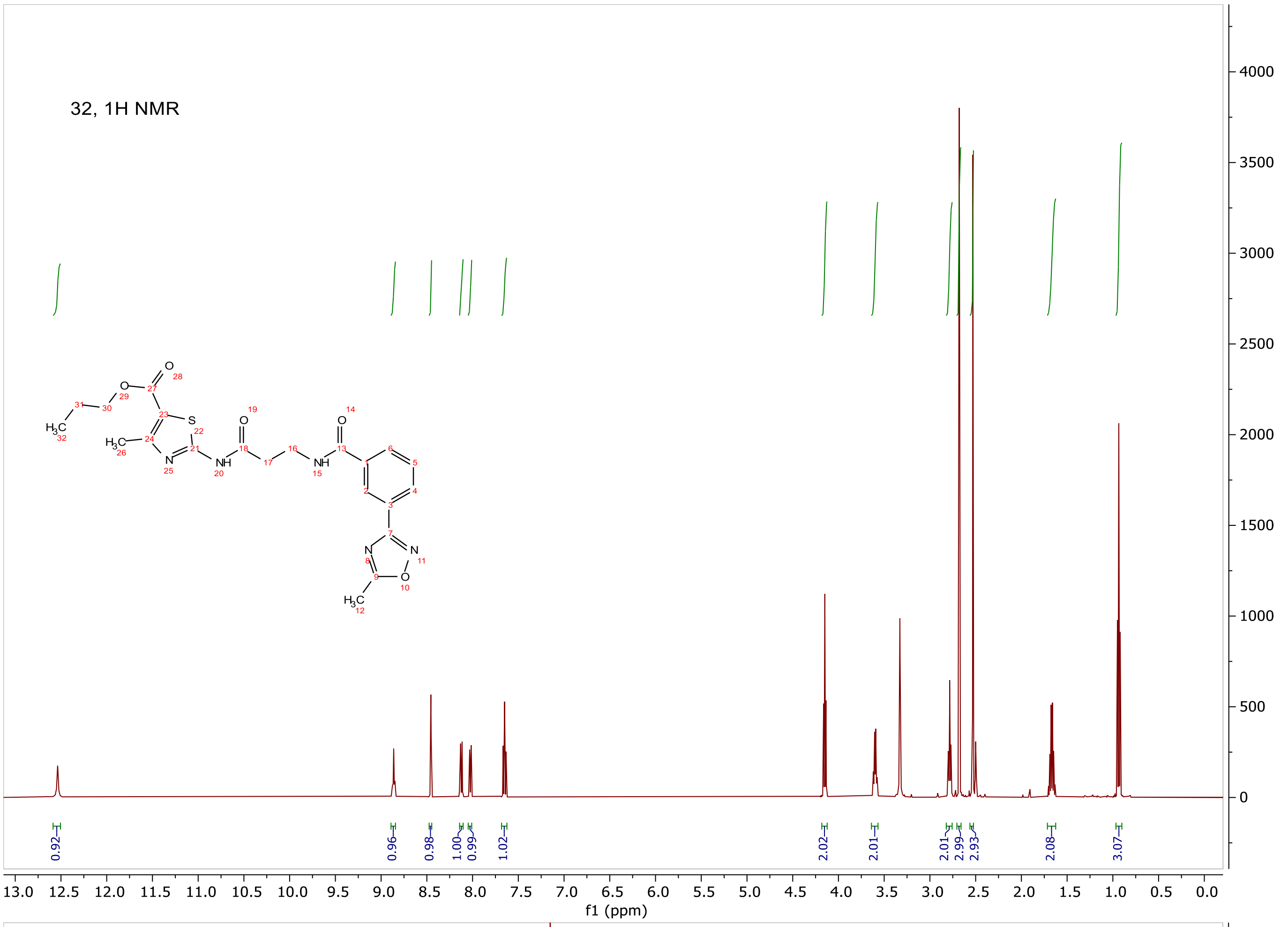

32, 1H NMR
0.92
0.96
0.98
1.00
0.99
1.02
2.02
2.01
2.01
2.99
2.93
2.08
3.07
f1 (ppm)

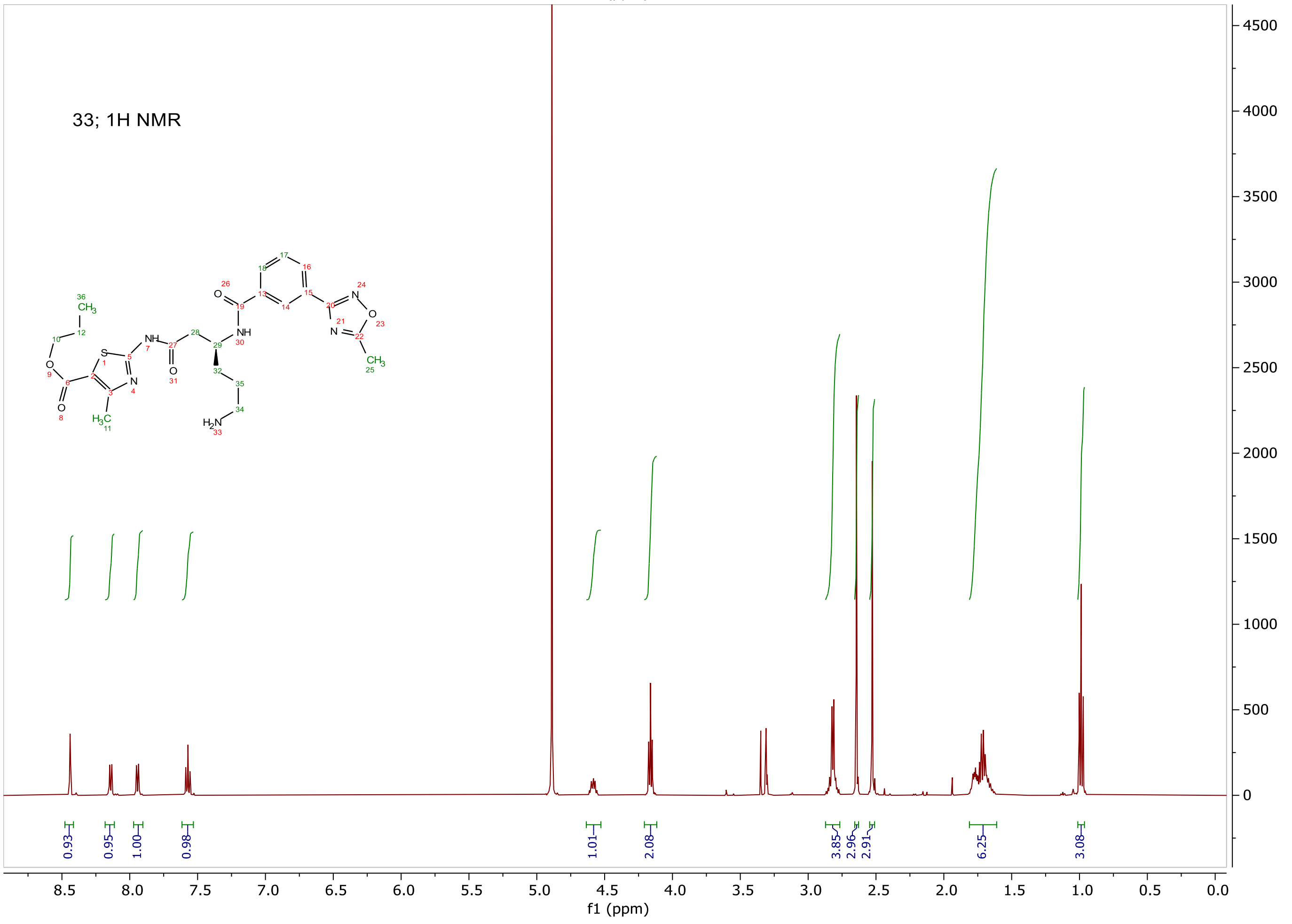

33; 1H NMR
0.93
0.95
1.00
0.98
1.01
2.08
3.85
2.96
2.91
6.25
3.08
f1 (ppm)

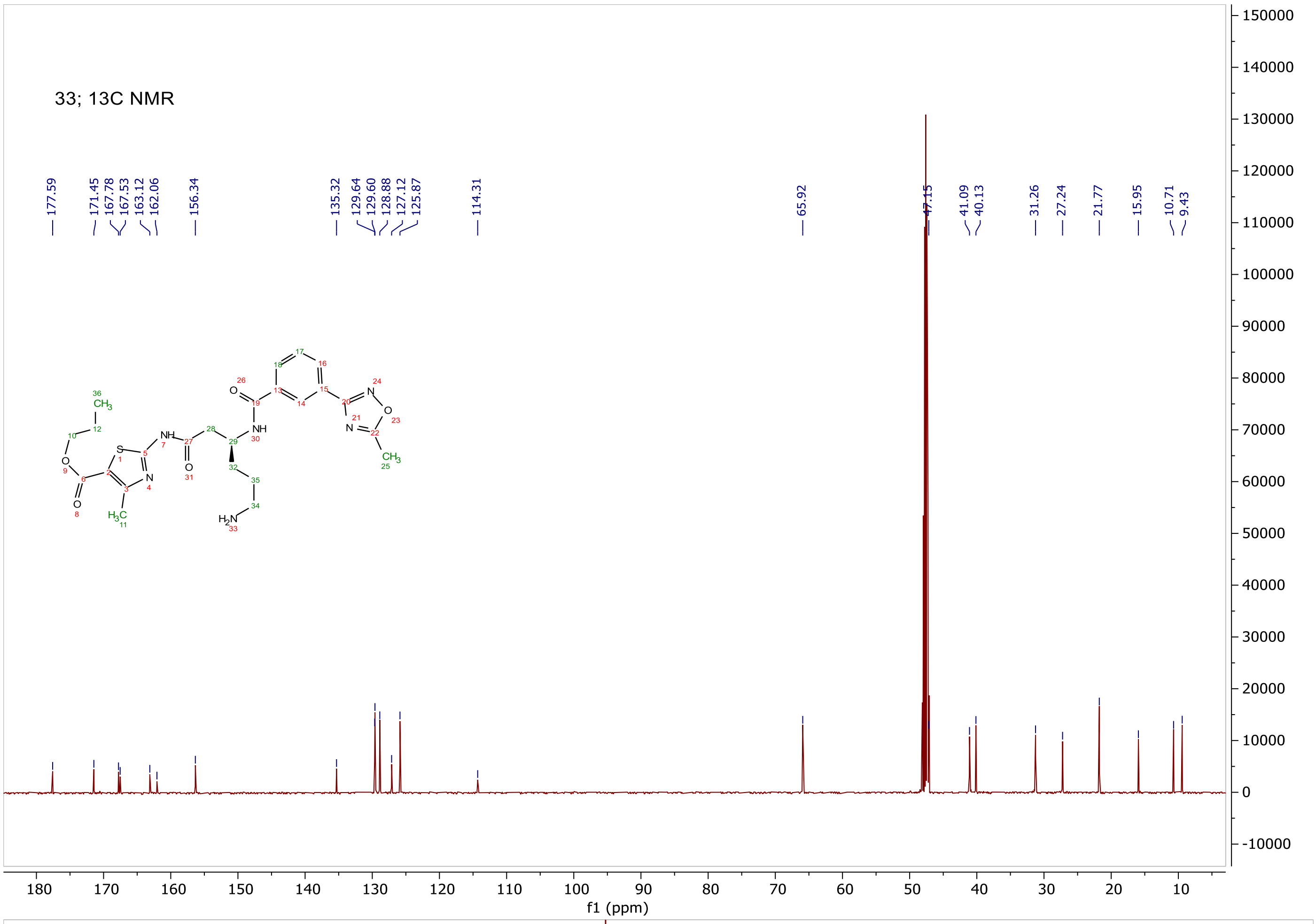
33; 13C NMR
177.59
171.45
167.78
167.53
163.12
162.06
156.34
135.32
129.64
129.60
128.88
127.12
125.87
114.31
65.92
47.15
41.09
40.13
31.26
27.24
21.77
15.95
10.71
9.43
f1 (ppm)

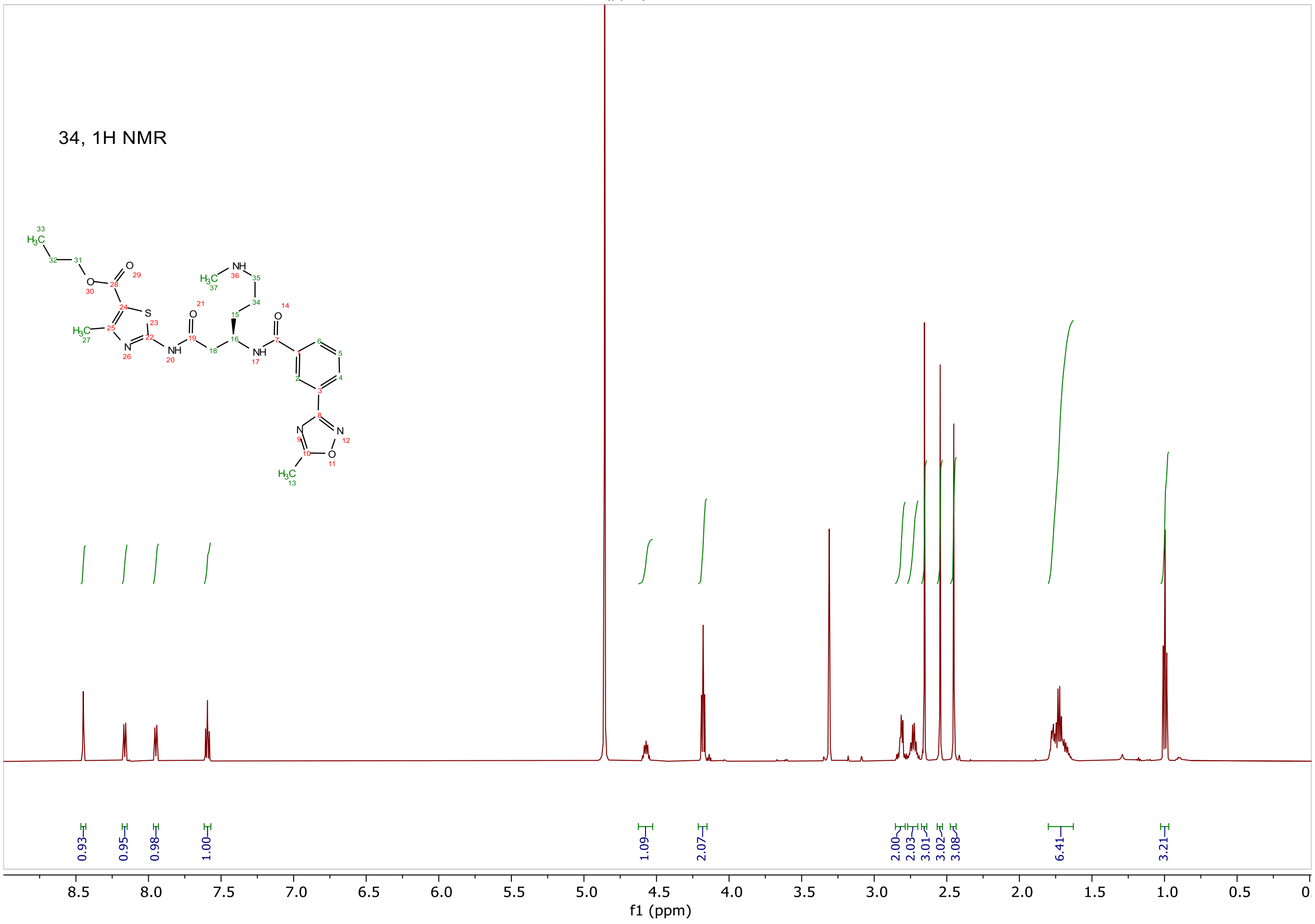
34, 1H NMR
0.93
0.95
0.98
1.00
1.09
2.07
2.00
2.03
3.01
3.02
3.08
6.41
3.21
f1 (ppm)

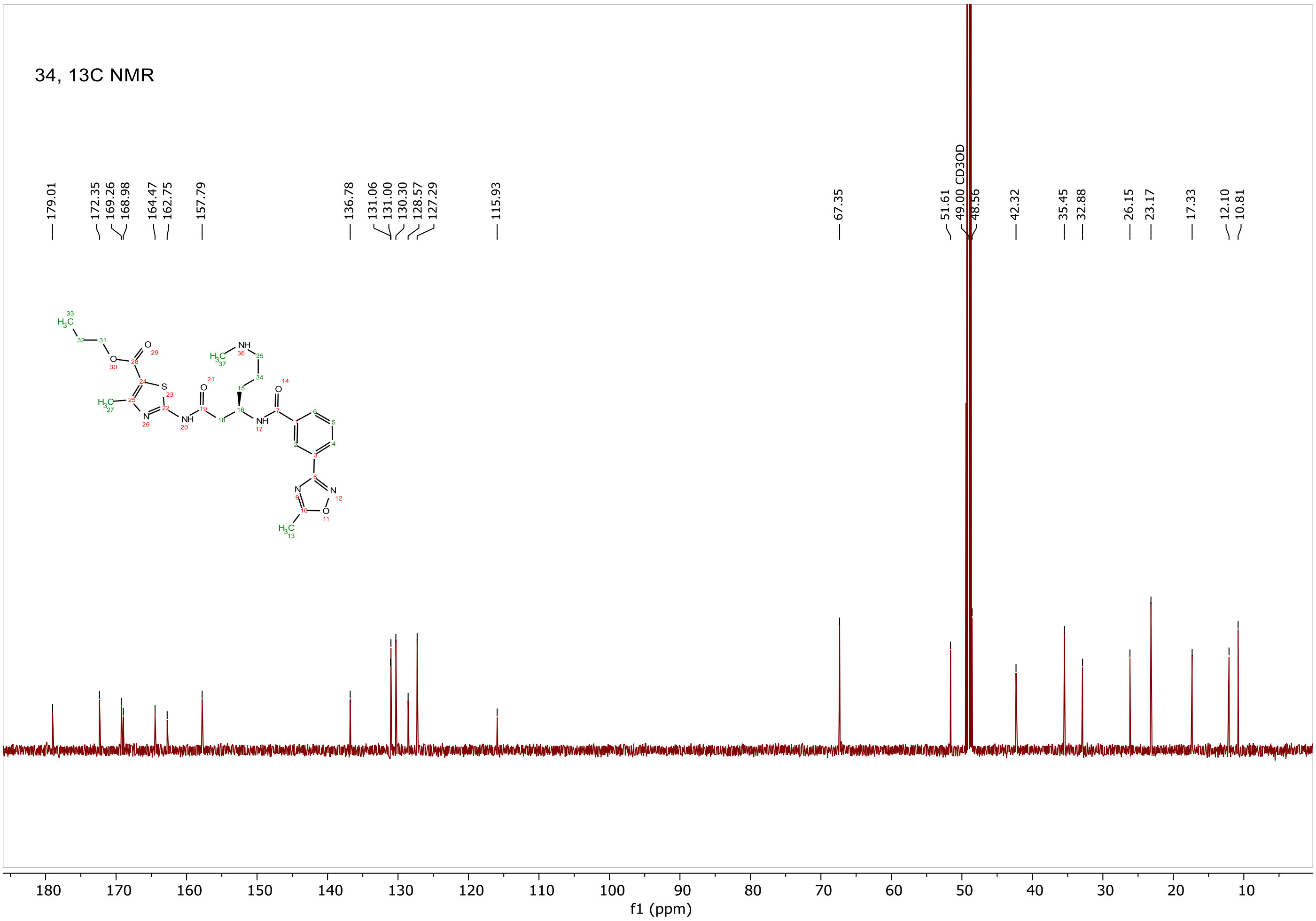
34, 13C NMR
179.01
172.35
169.26
168.98
164.47
162.75
157.79
136.78
131.06
131.00
130.30
128.57
127.29
115.93
67.35
51.61
49.00 CD3OD
48.56
42.32
35.45
32.88
26.15
23.17
17.33
12.10
10.81
f1 (ppm)

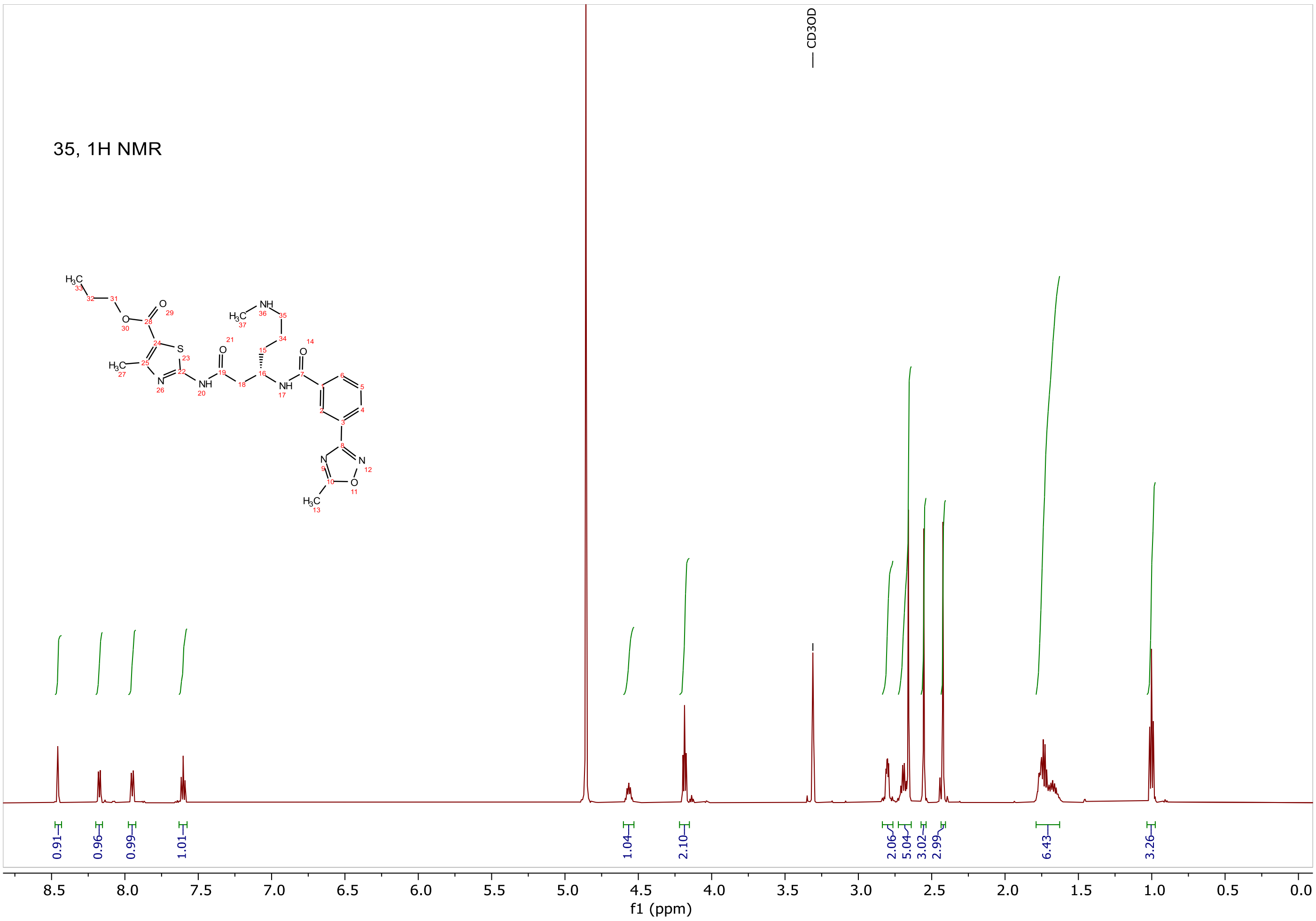
35, 1H NMR
CD3OD
0.91
0.96
0.99
1.01
1.04
2.10
2.06
5.04
3.02
2.99
6.43
3.26
f1 (ppm)

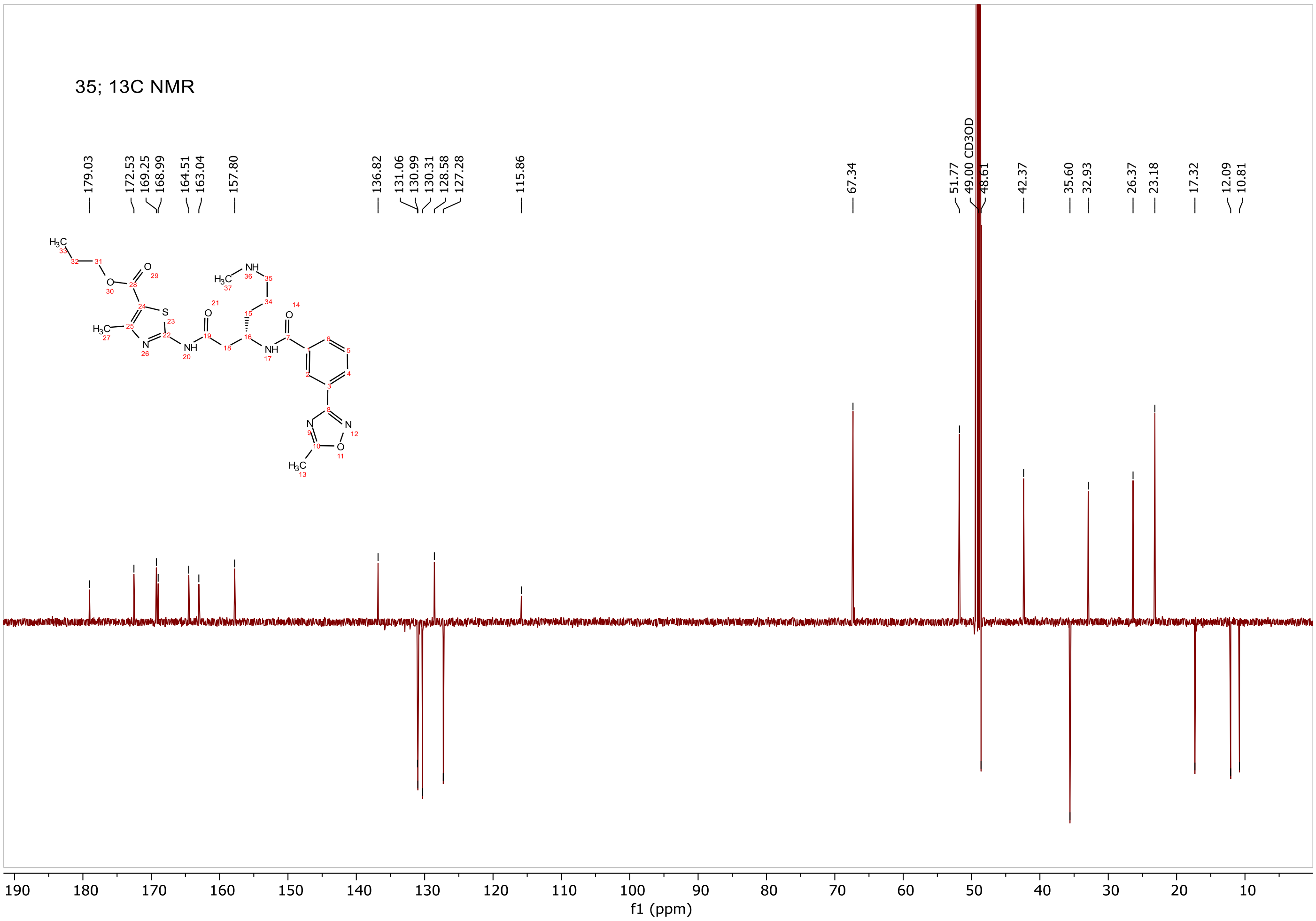
35; 13C NMR
179.03
172.53
169.25
168.99
164.51
163.04
157.80
136.82
131.06
130.99
130.31
128.58
127.28
115.86
67.34
51.77
49.00 CD3OD
48.61
42.37
35.60
32.93
26.37
23.18
17.32
12.09
10.81
f1 (ppm)

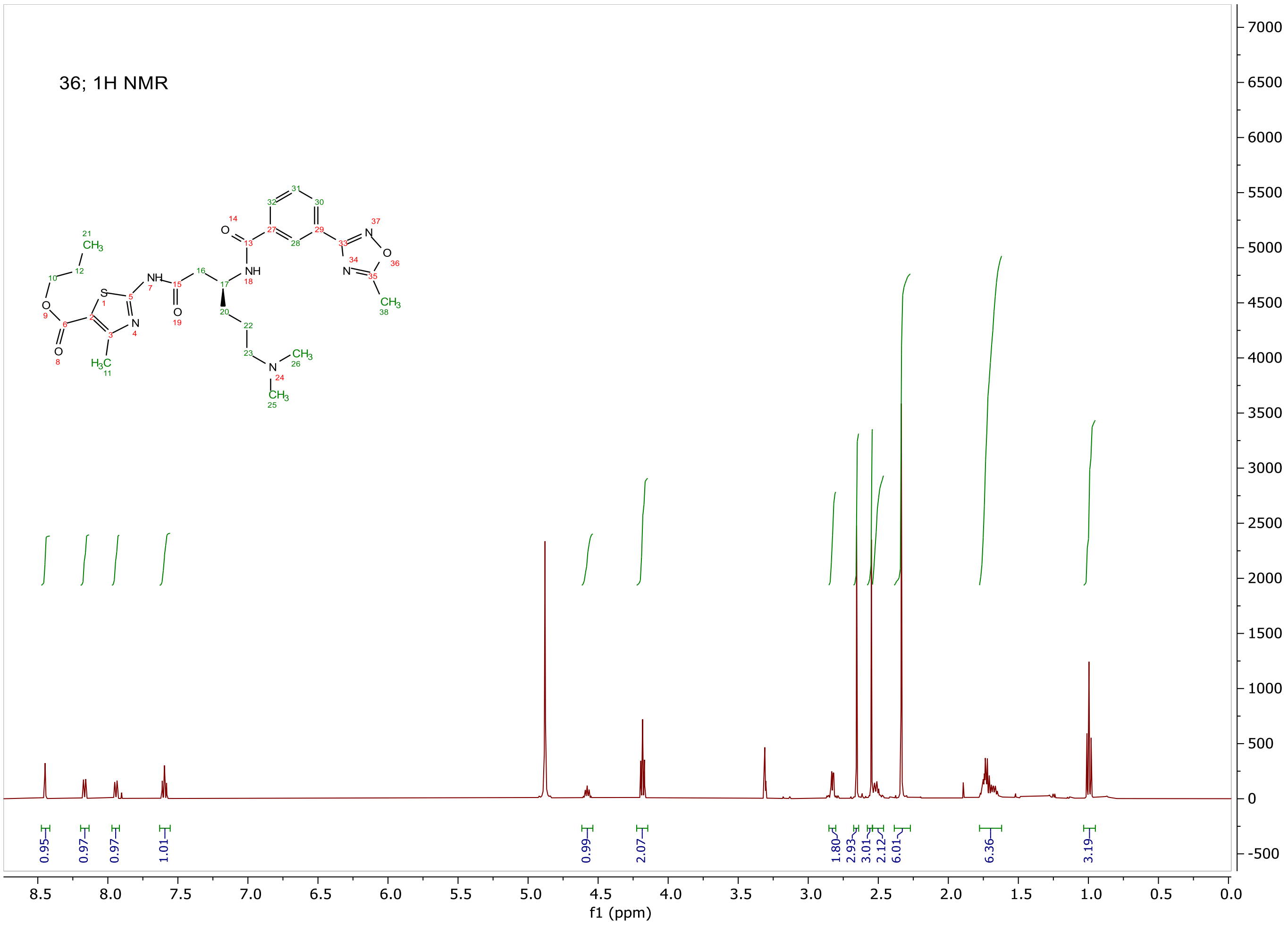
36; 1H NMR
0.95
0.97
0.97
1.01
0.99
2.07
1.80
2.93
3.01
2.12
6.01
6.36
3.19
f1 (ppm)

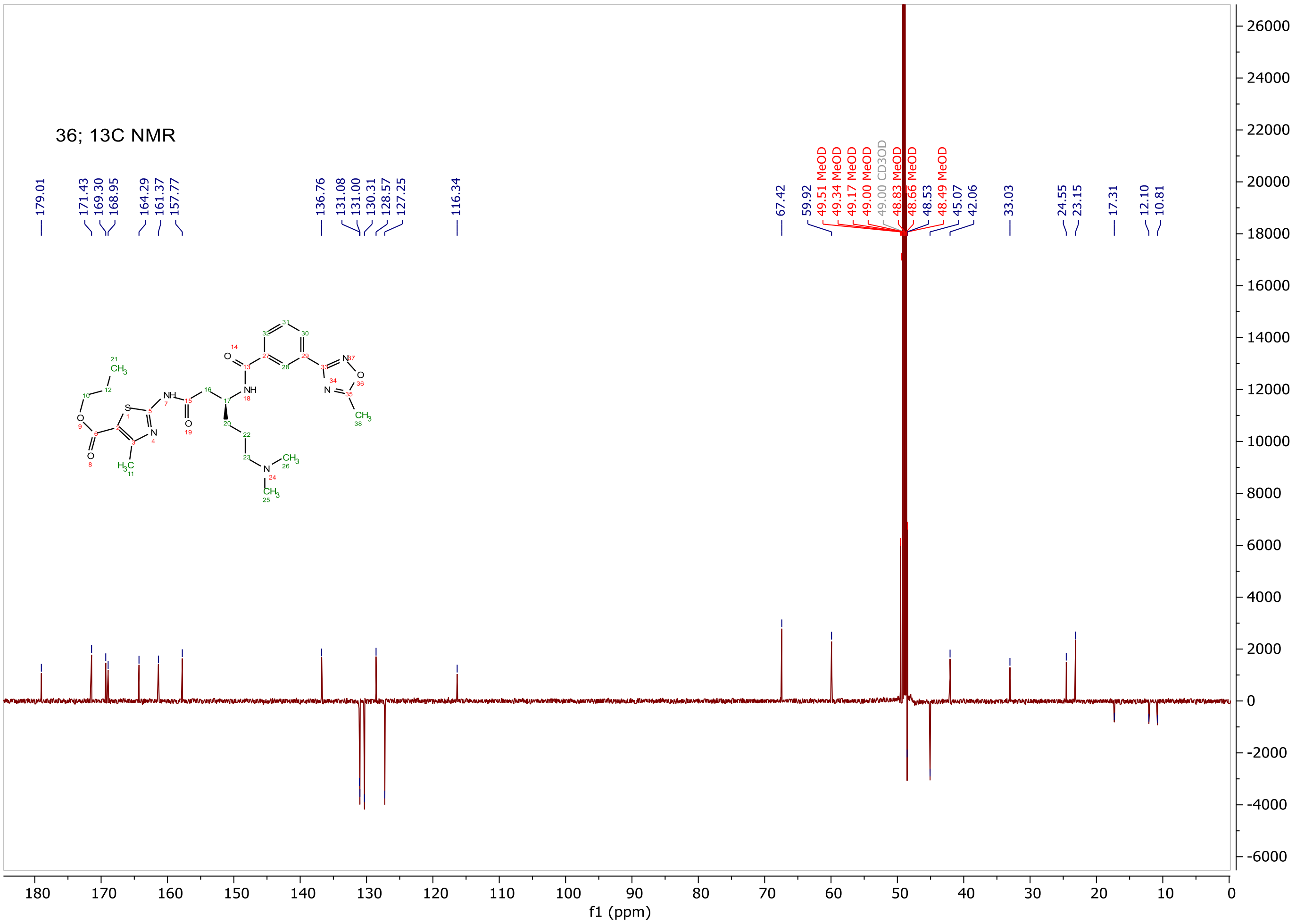
36; 13C NMR
179.01
171.43
169.30
168.95
164.29
161.37
157.77
136.76
131.08
131.00
130.31
128.57
127.25
116.34
67.42
59.92
49.51 MeOD
49.34 MeOD
49.17 MeOD
49.00 MeOD
49.00 CD3OD
48.83 MeOD
48.66 MeOD
48.53
48.49 MeOD
45.07
42.06
33.03
24.55
23.15
17.31
12.10
10.81
f1 (ppm)

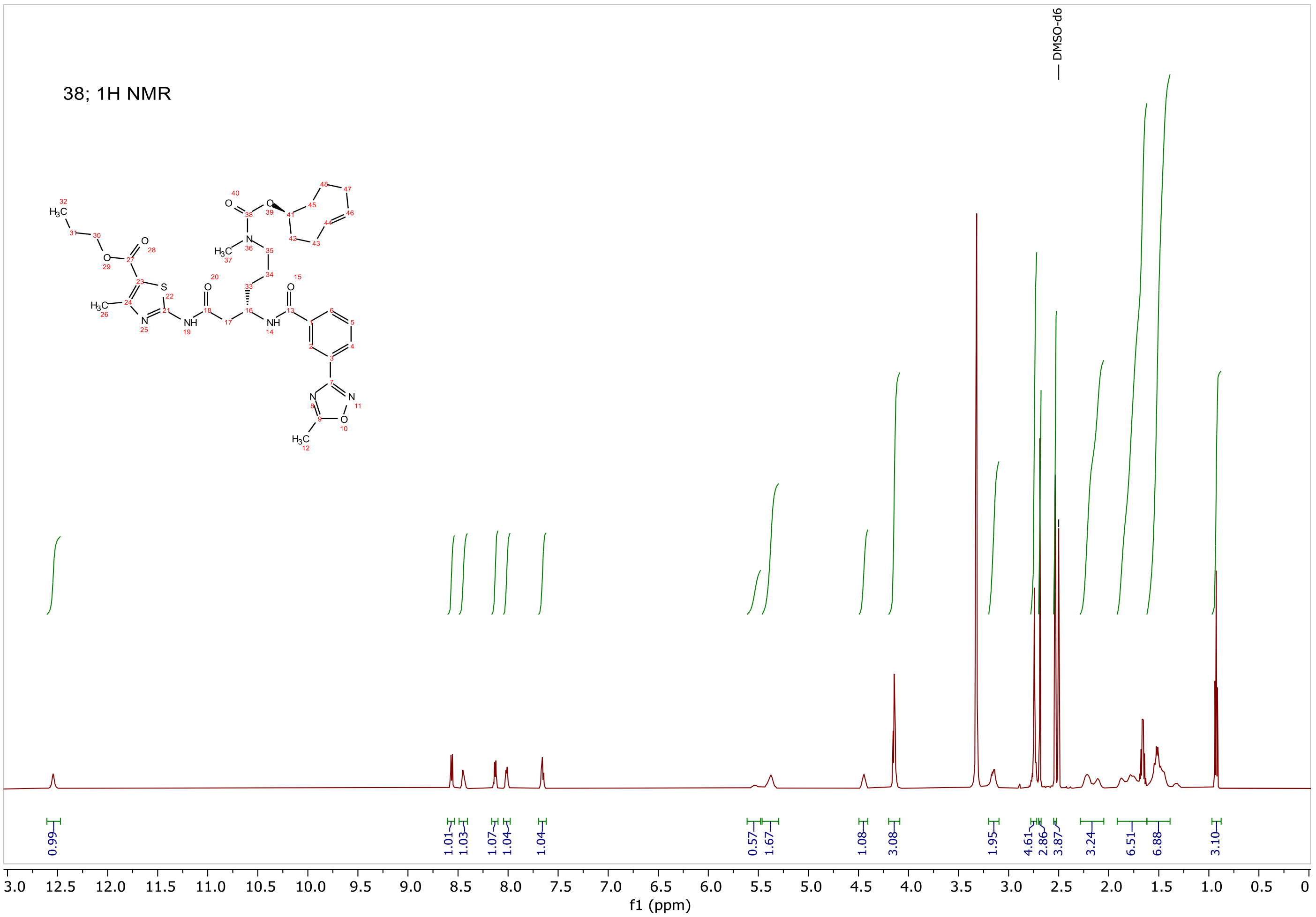
38; 1H NMR
DMSO-d6
0.99
1.01
1.03
1.07
1.04
1.04
0.57
1.67
1.08
3.08
1.95
4.61
2.86
3.87
3.24
6.51
6.88
3.10
f1 (ppm)

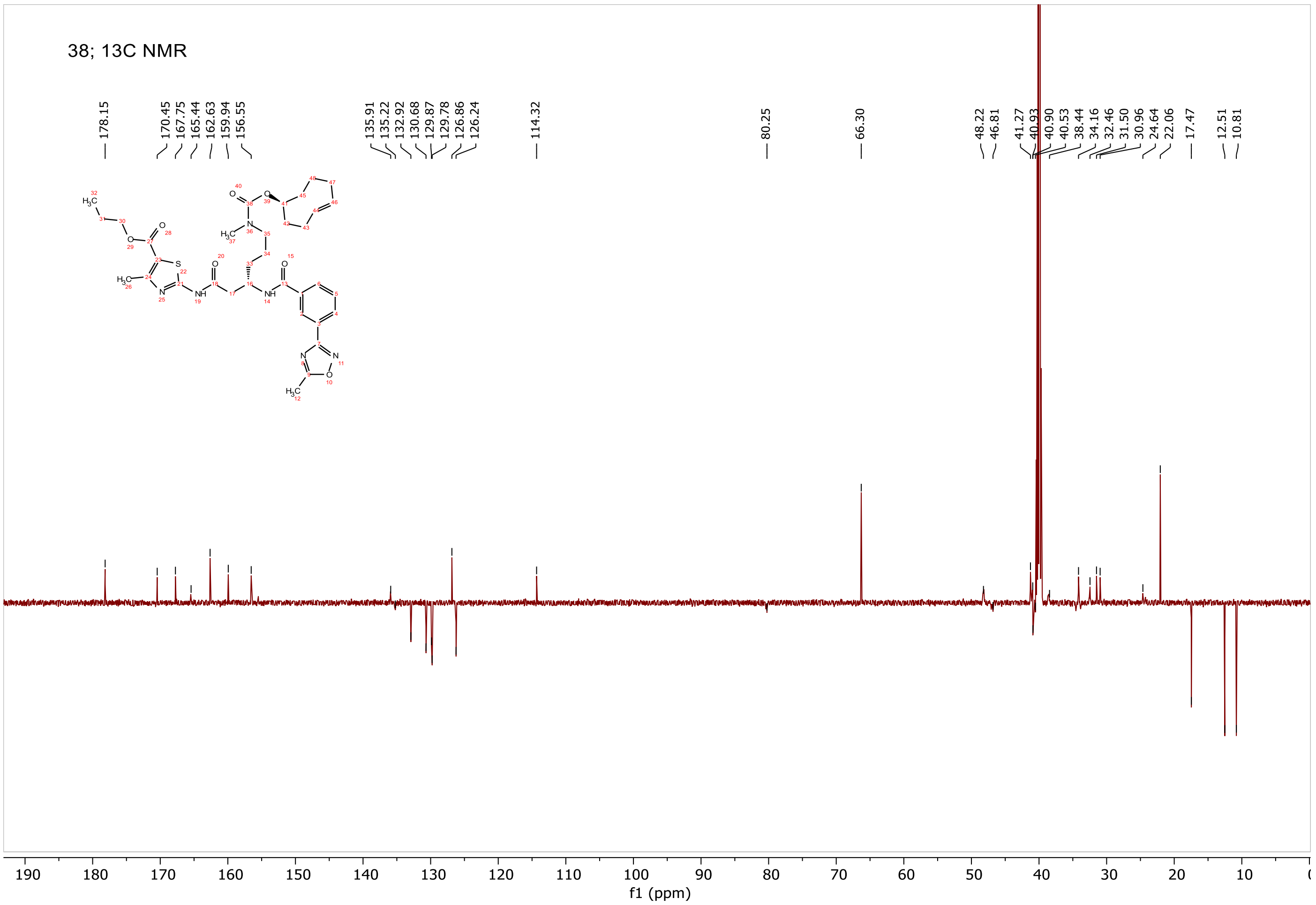
38; 13C NMR
178.15
170.45
167.75
165.44
162.63
159.94
156.55
135.91
135.22
132.92
130.68
129.87
129.78
126.86
126.24
114.32
80.25
66.30
48.22
46.81
41.27
40.93
40.90
40.53
38.44
34.16
32.46
31.50
30.96
24.64
22.06
17.47
12.51
10.81
f1 (ppm)

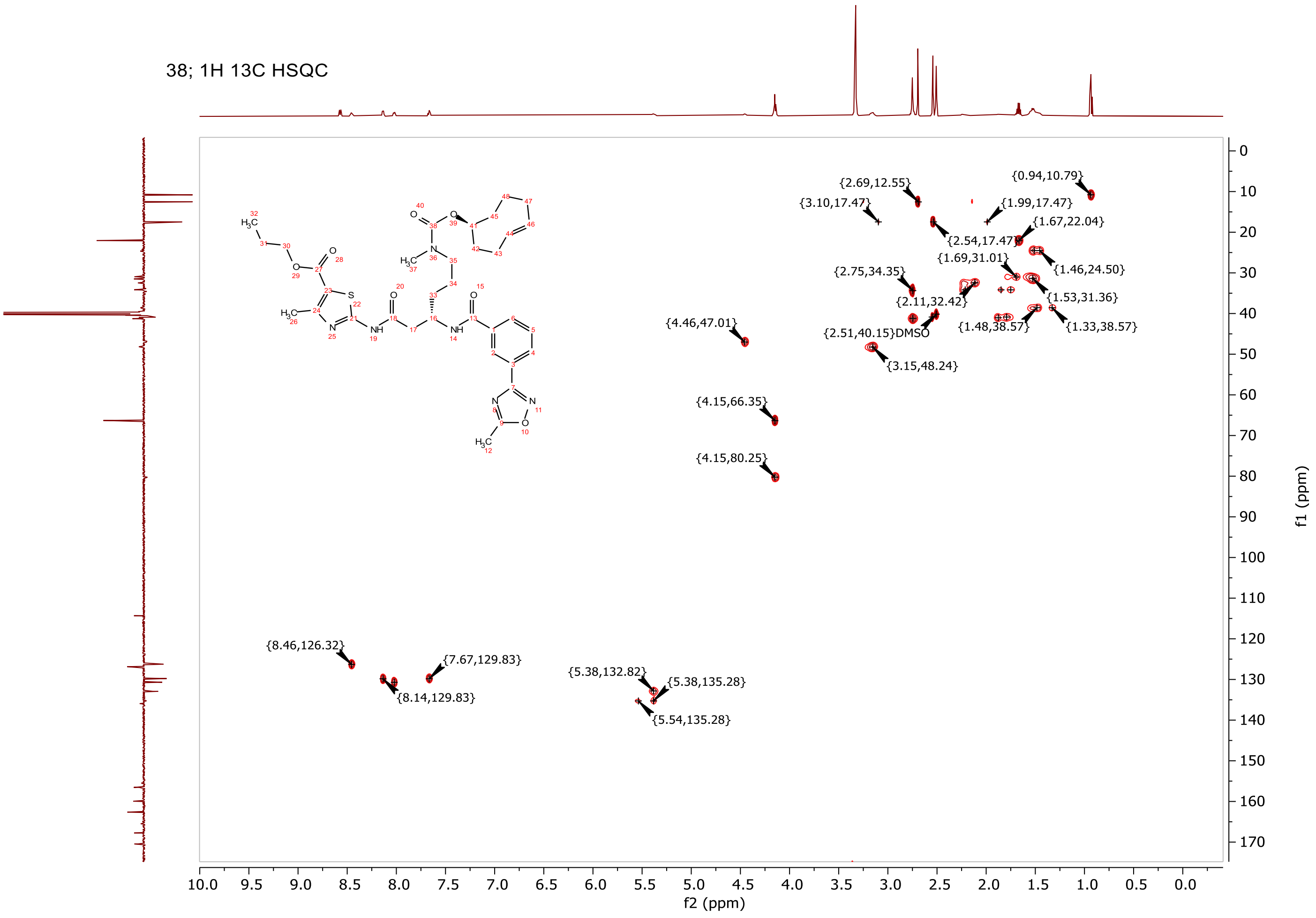
38; 1H 13C HSQC
{2.69,12.55}
{0.94,10.79}
{3.10,17.47}
{1.99,17.47}
{1.67,22.04}
{2.54,17.47}
{1.69,31.01}
{1.46,24.50}
{2.75,34.35}
{1.53,31.36}
{2.11,32.42}
{4.46,47.01}
{1.48,38.57}
{1.33,38.57}
{2.51,40.15}DMSO
{3.15,48.24}
{4.15,66.35}
{4.15,80.25}
{8.46,126.32}
{7.67,129.83}
{5.38,132.82}
{5.38,135.28}
{8.14,129.83}
{5.54,135.28}
f2 (ppm)
f1 (ppm)

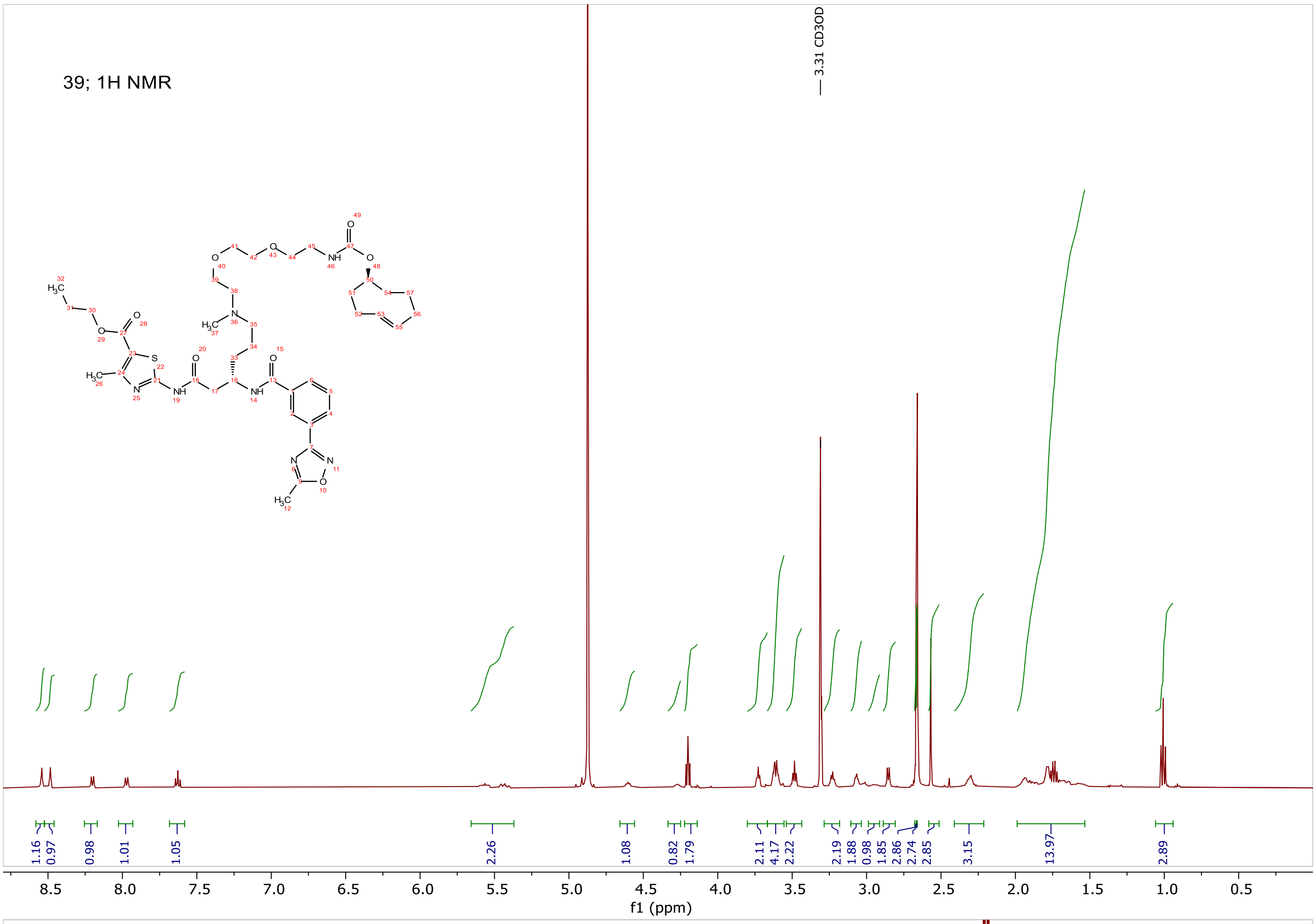
39; 1H NMR
3.31 CD3OD
1.16
0.97
0.98
1.01
1.05
2.26
1.08
0.82
1.79
2.11
4.17
2.22
2.19
1.88
0.98
1.85
2.86
2.74
2.85
3.15
13.97
2.89
f1 (ppm)

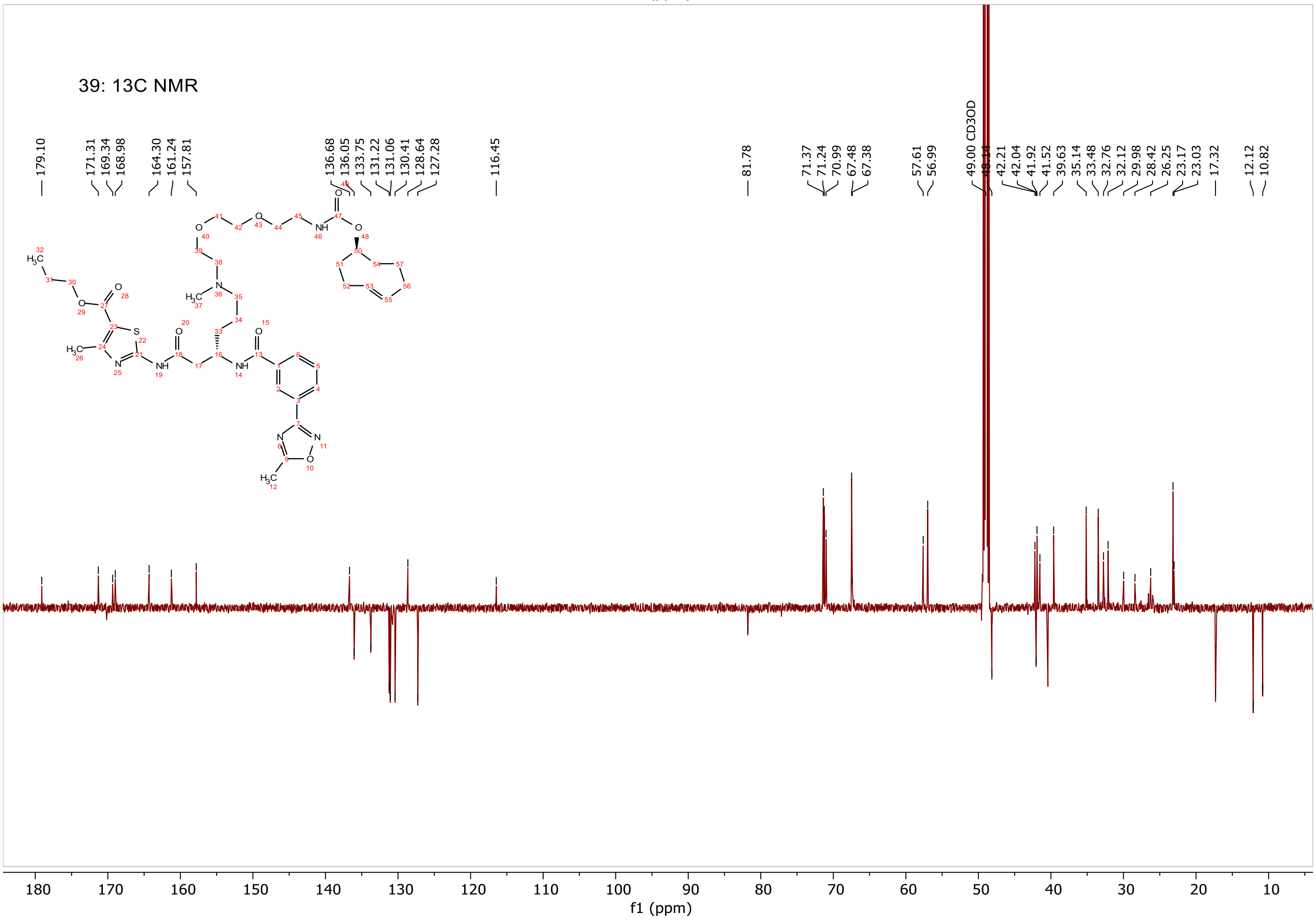
39: 13C NMR
179.10
171.31
169.34
168.98
164.30
161.24
157.81
136.68
136.05
133.75
131.22
131.06
130.41
128.64
127.28
116.45
81.78
71.37
71.24
70.99
67.48
67.38
57.61
56.99
49.00 CD3OD
42.21
42.04
41.92
41.52
39.63
35.14
33.48
32.76
32.12
29.98
28.42
26.25
23.17
23.03
17.32
12.12
10.82
f1 (ppm)

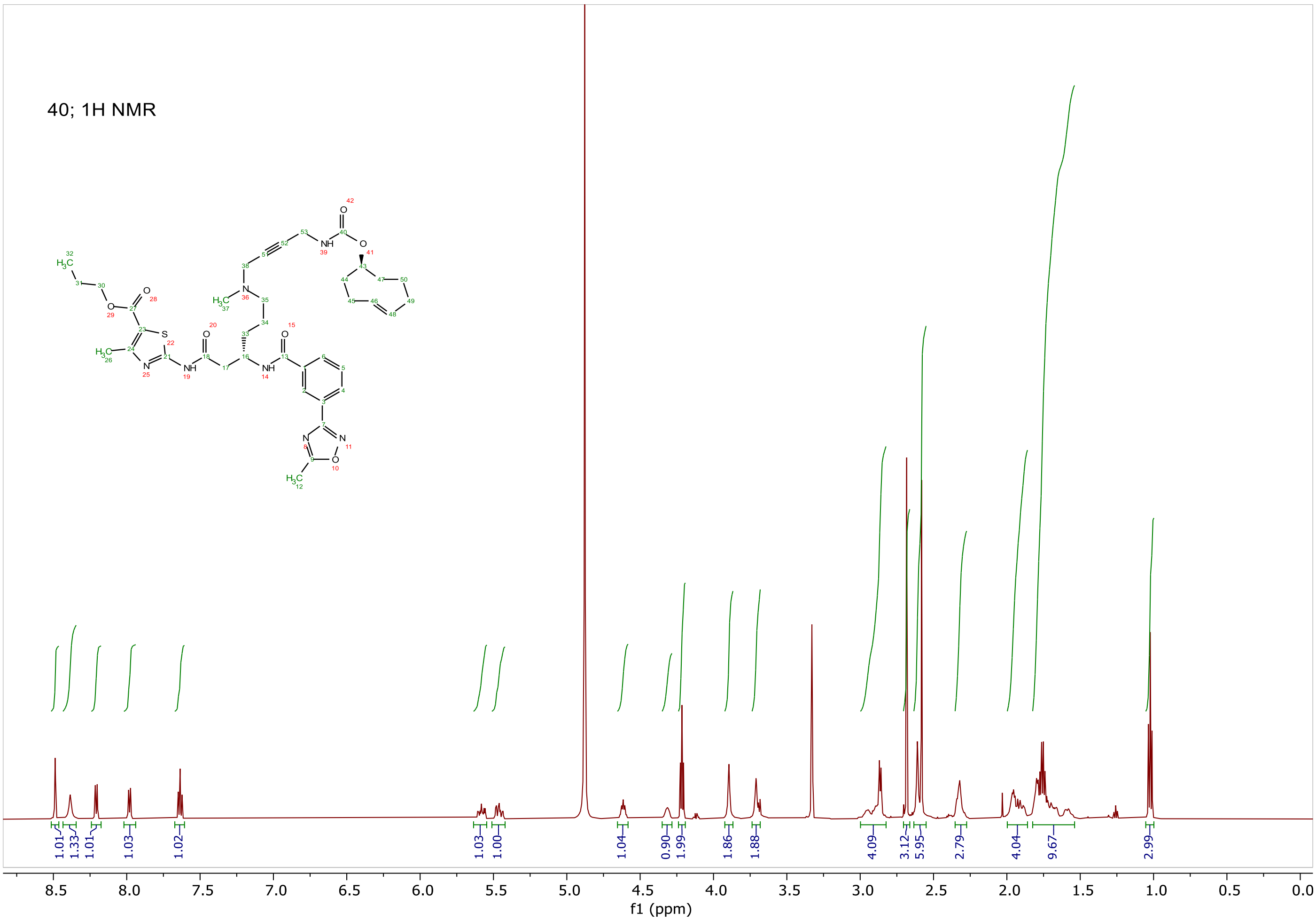
40; 1H NMR
1.01
1.33
1.01
1.03
1.02
1.03
1.00
1.04
0.90
1.99
1.86
1.88
4.09
3.12
5.95
2.79
4.04
9.67
2.99
f1 (ppm)

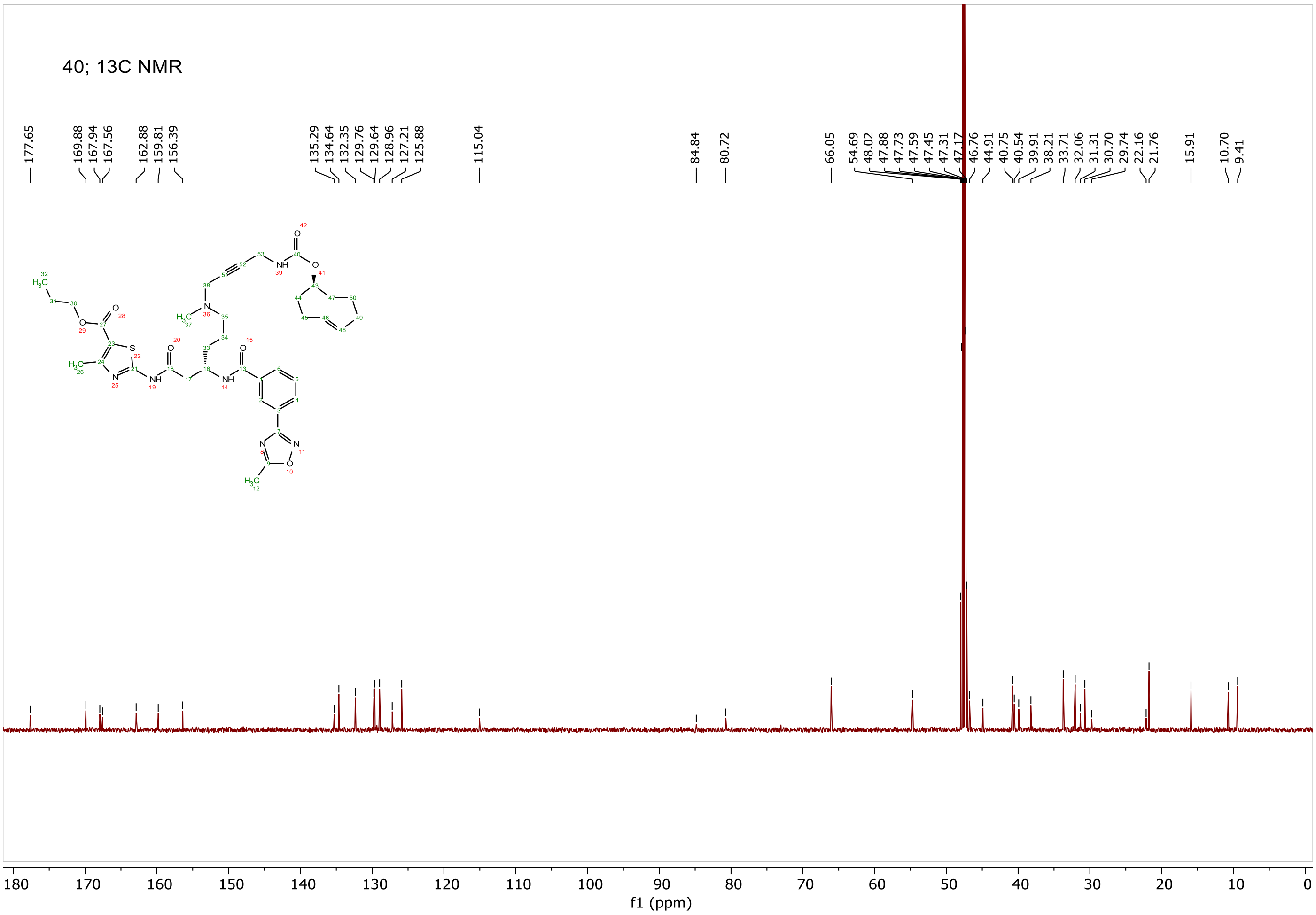
40; 13C NMR
177.65
169.88
167.94
167.56
162.88
159.81
156.39
135.29
134.64
132.35
129.76
129.64
128.96
127.21
125.88
115.04
84.84
80.72
66.05
54.69
48.02
47.88
47.73
47.59
47.45
47.31
47.17
46.76
44.91
40.75
40.54
39.91
38.21
33.71
32.06
31.31
30.70
29.74
22.16
21.76
15.91
10.70
9.41
f1 (ppm)

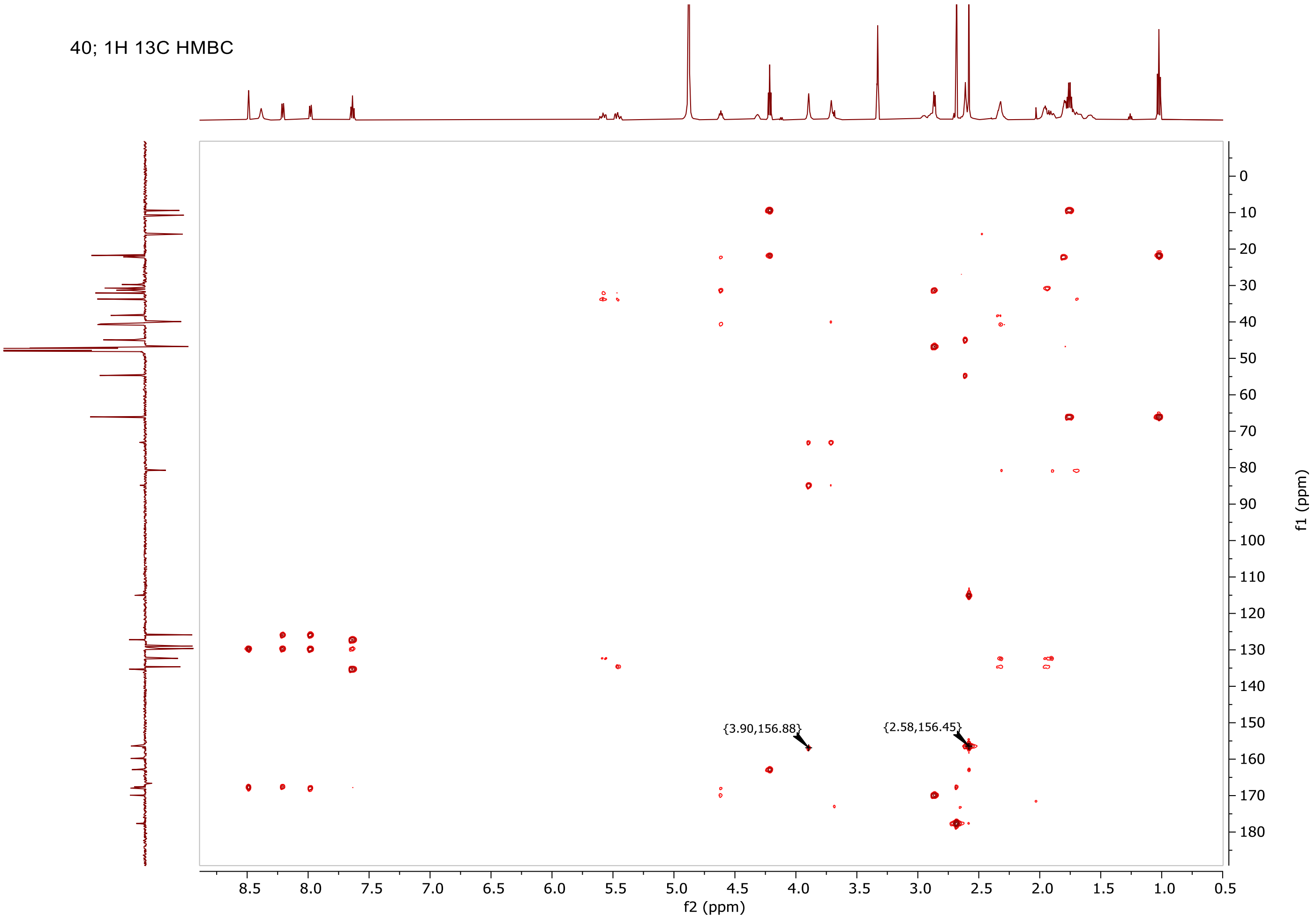

7. Copies of HPLC traces for test compounds **2**, **4-7**, **9-40**

Compound 2

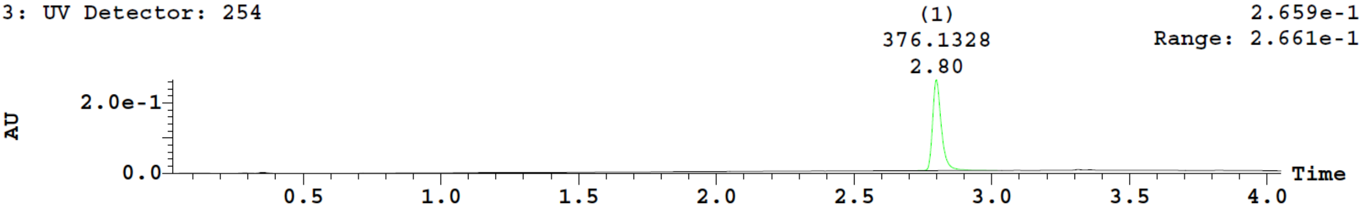

Peak ID	Time	Area %Total
1	2.80	100

Compound 4

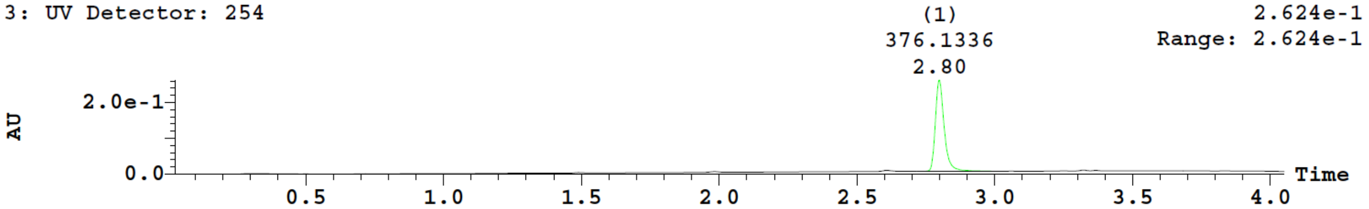

Peak ID	Time	Area %Total
1	2.80	100

Compound 5

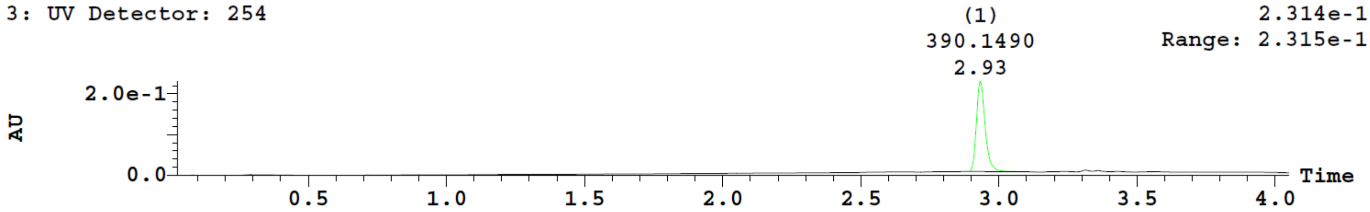

Peak ID	Time	Area %Total
1	2.93	100

Compound 6

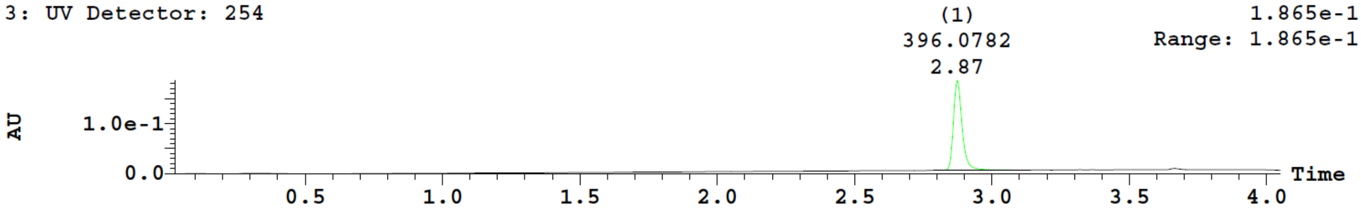

Peak ID	Time	Area %Total
1	2.87	100

Compound 7

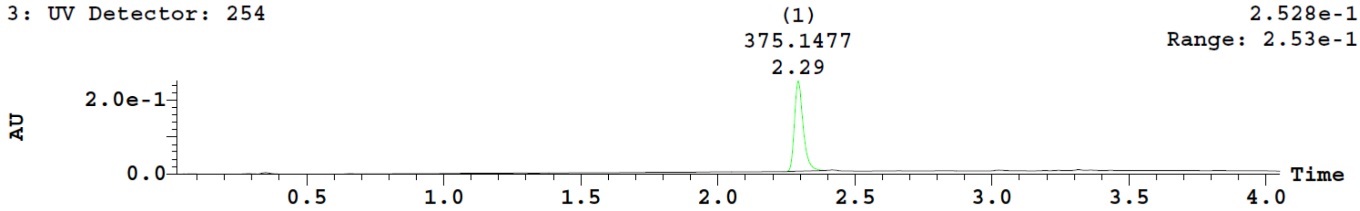

Peak ID	Time	Area %Total
1	2.29	100

Compound 9

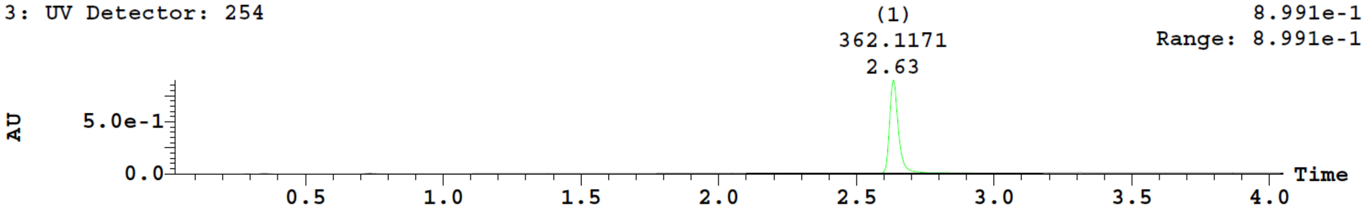

Peak ID	Time	Area %Total
1	2.63	100

Compound 10

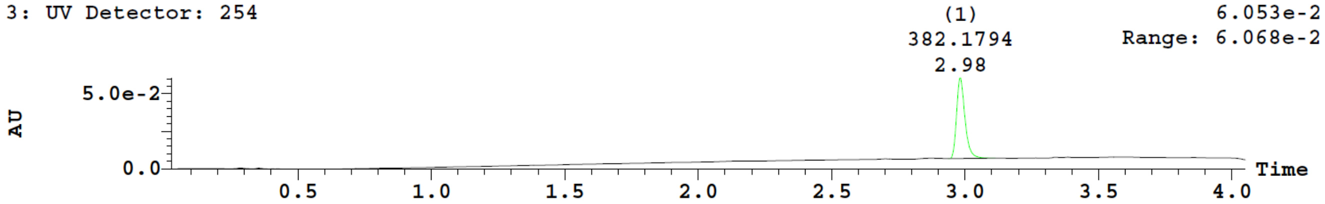

Peak ID	Time	Area %Total
1	2.98	100

Compound 11

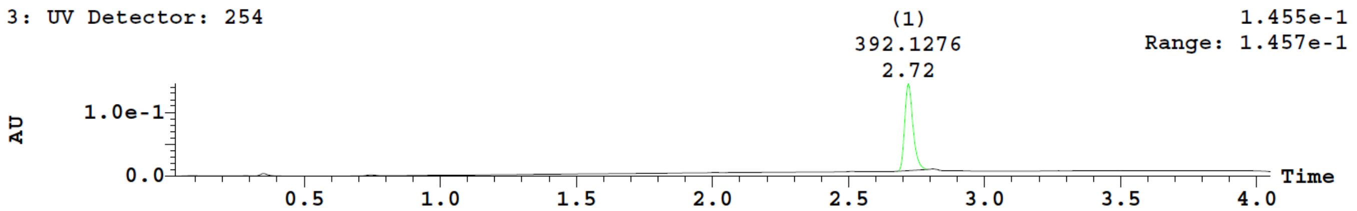

Peak ID	Time	Area %Total
1	2.72	100

Compound 12

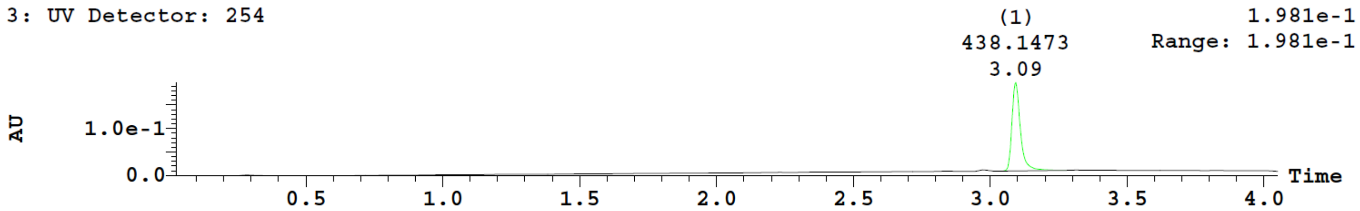

Peak ID	Time	Area %Total
1	3.09	100

Compound 13

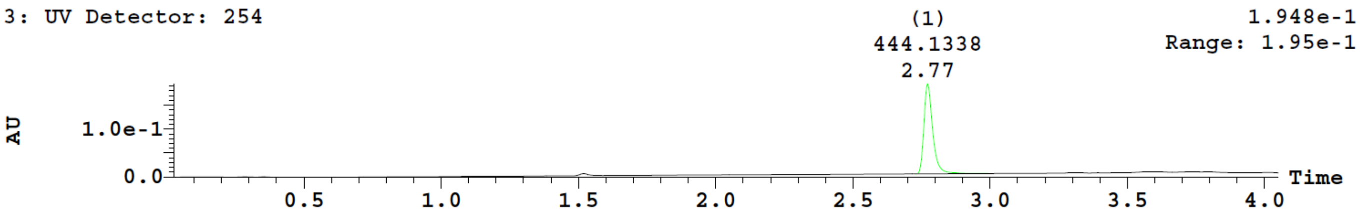

Peak ID	Time	Area %Total
1	2.77	100

Compound 14

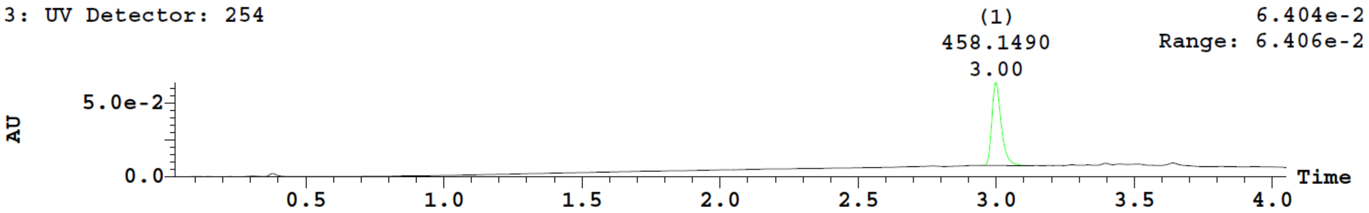

Peak ID	Time	Area %Total
1	3.00	100

Compound 15

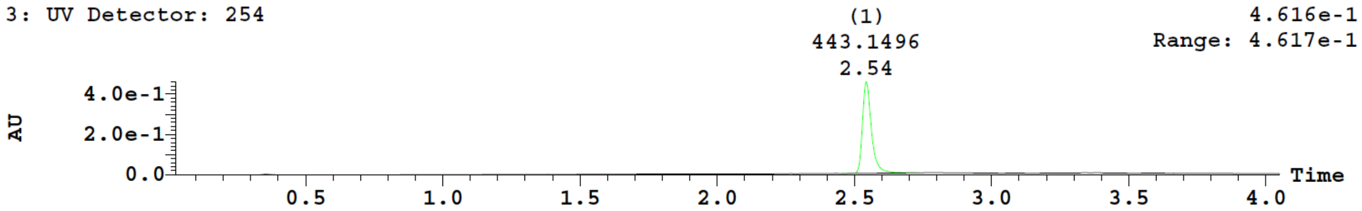

Peak ID	Time	Area %Total
1	2.54	100

Compound 16

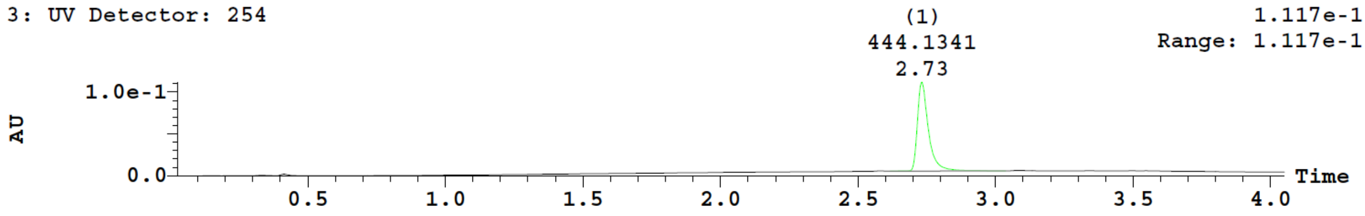

Peak ID	Time	Area %Total
1	2.73	100

Compound 17

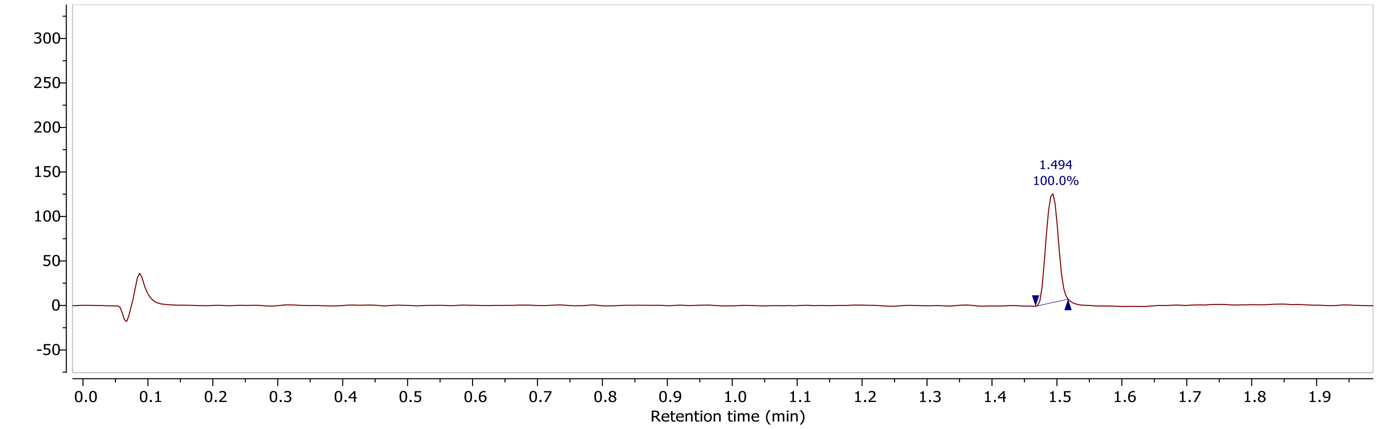

Compound 18

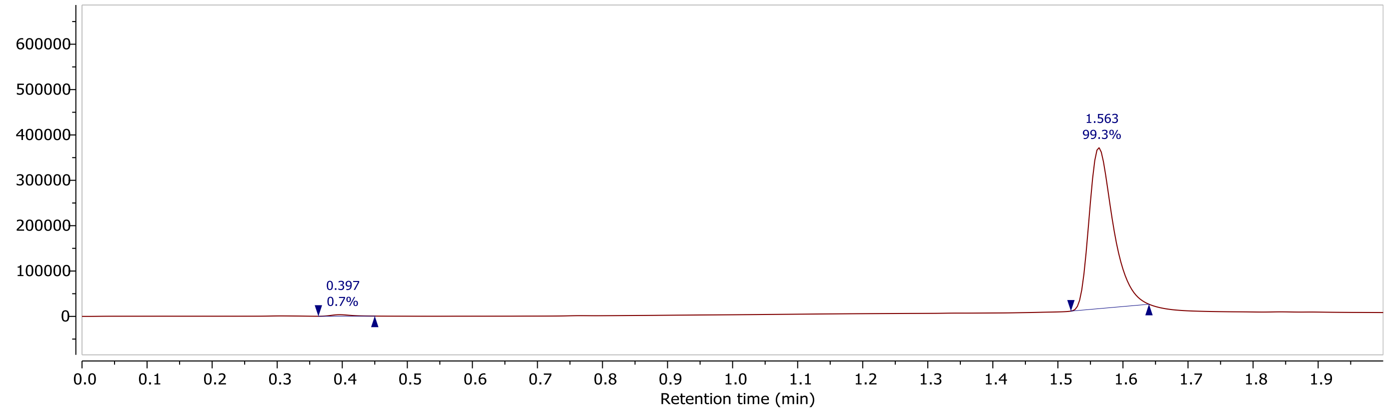

Compound 19

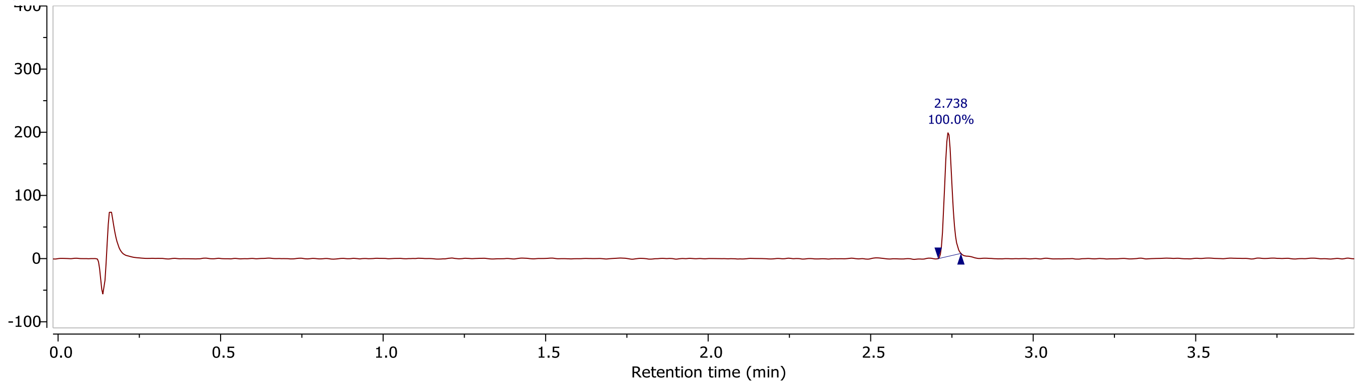

Compound 20

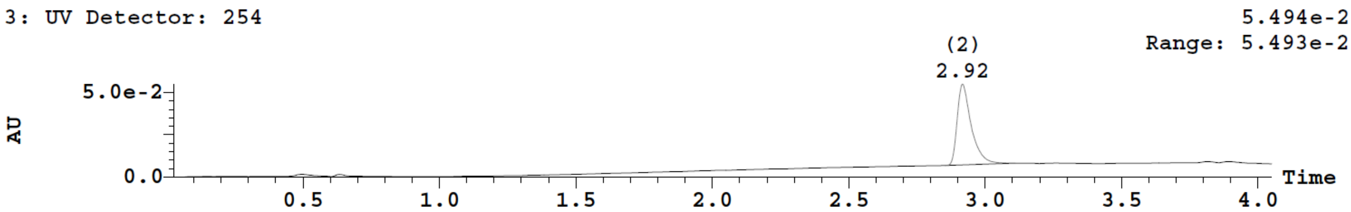

Peak ID	Time	Area %Total
2	2.92	100

Compound 21

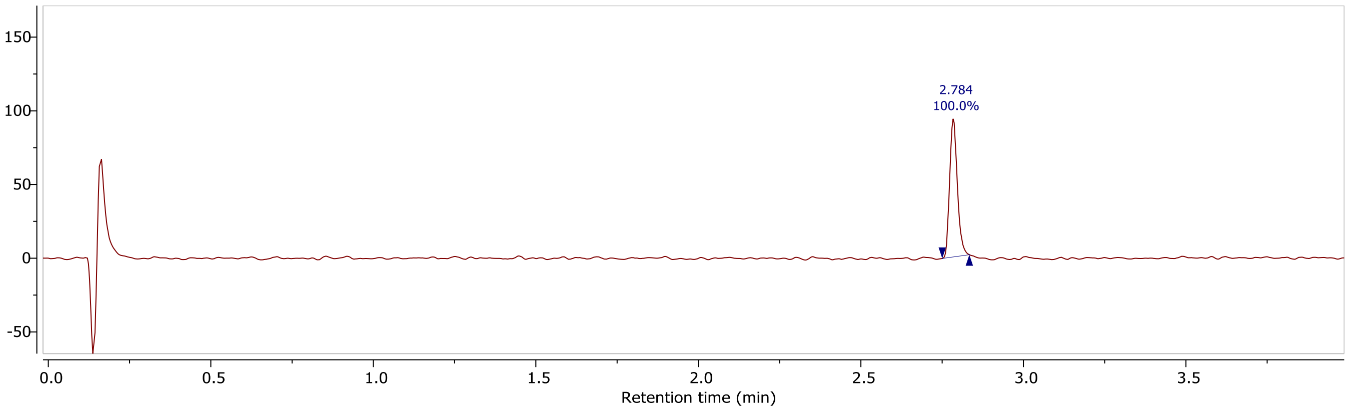

Compound 22

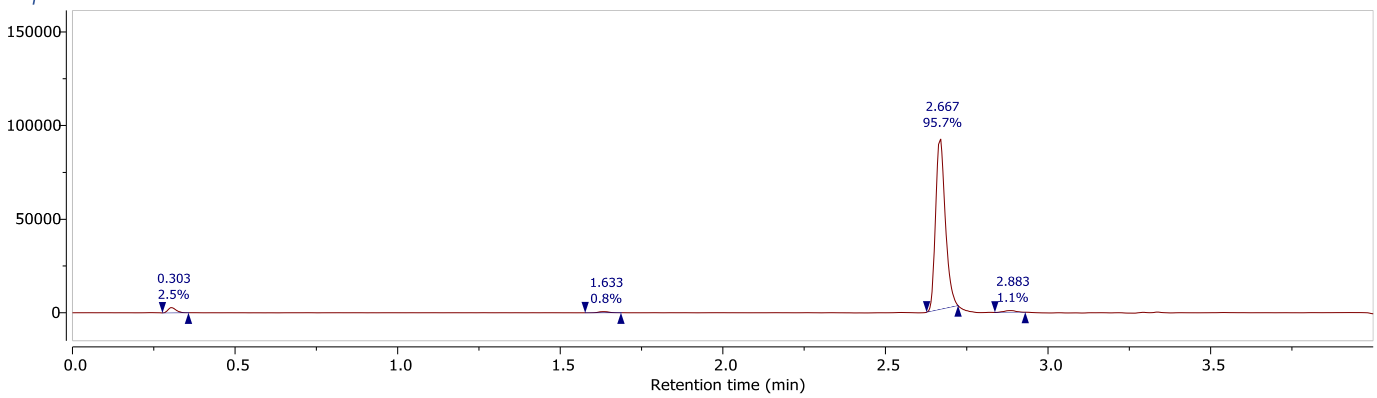

Compound 23

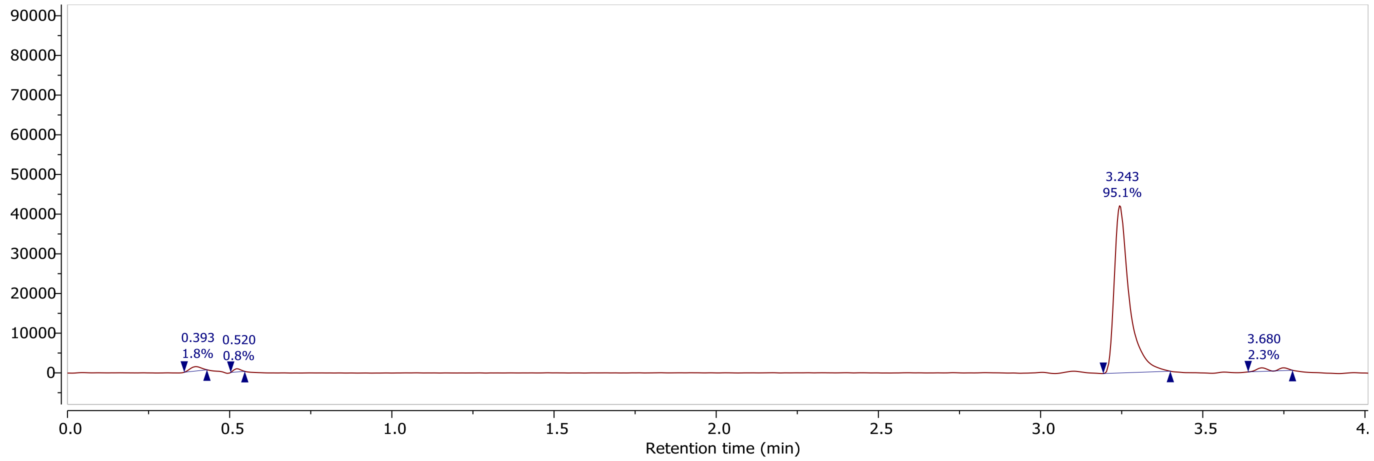

Compound 24

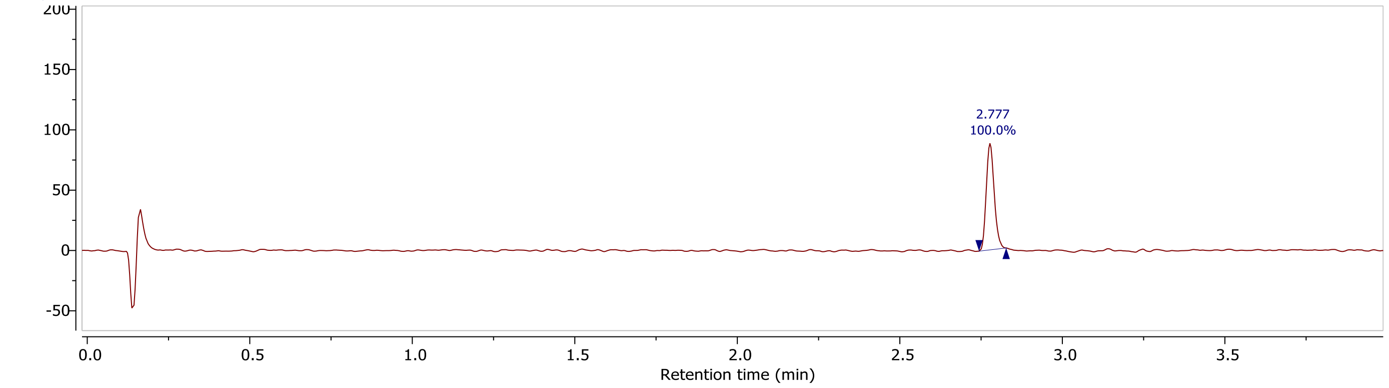

Compound 25

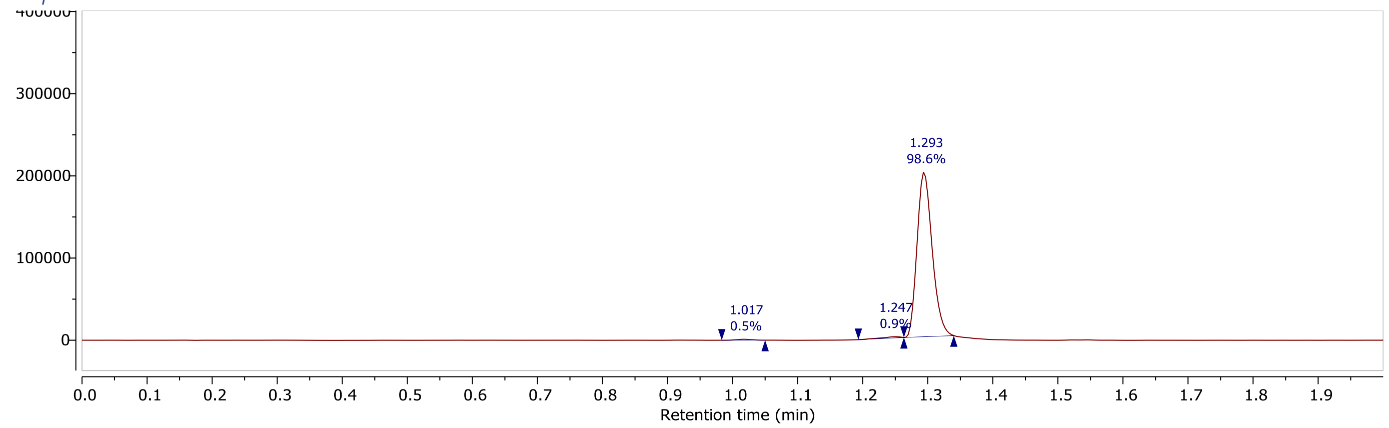

Compound 26

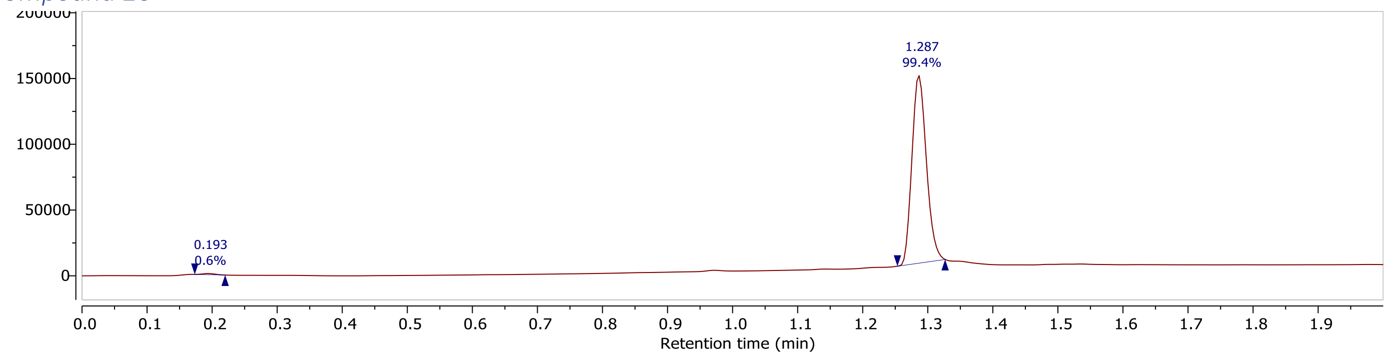

Compound 27

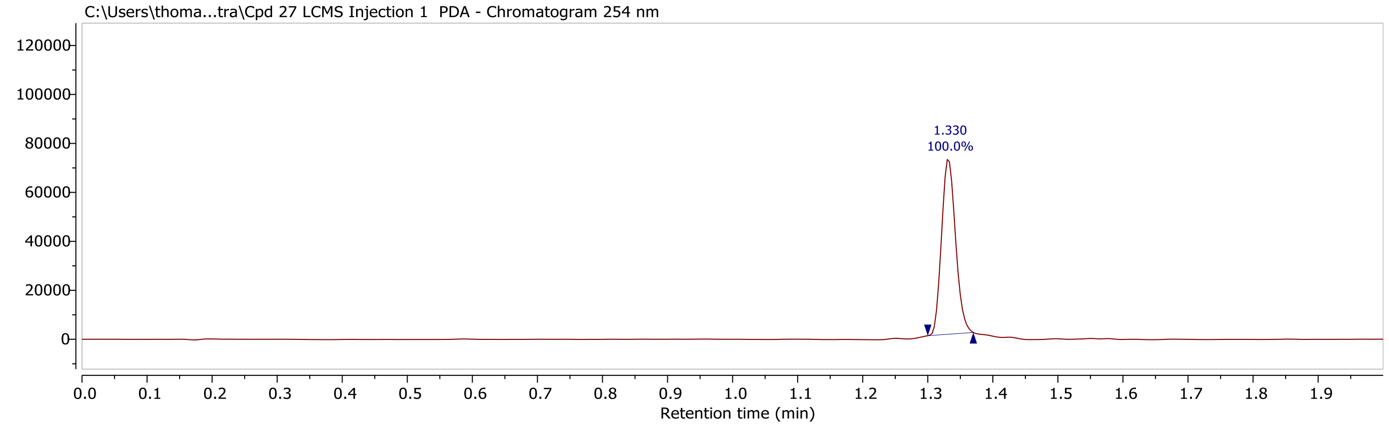

Compound 28

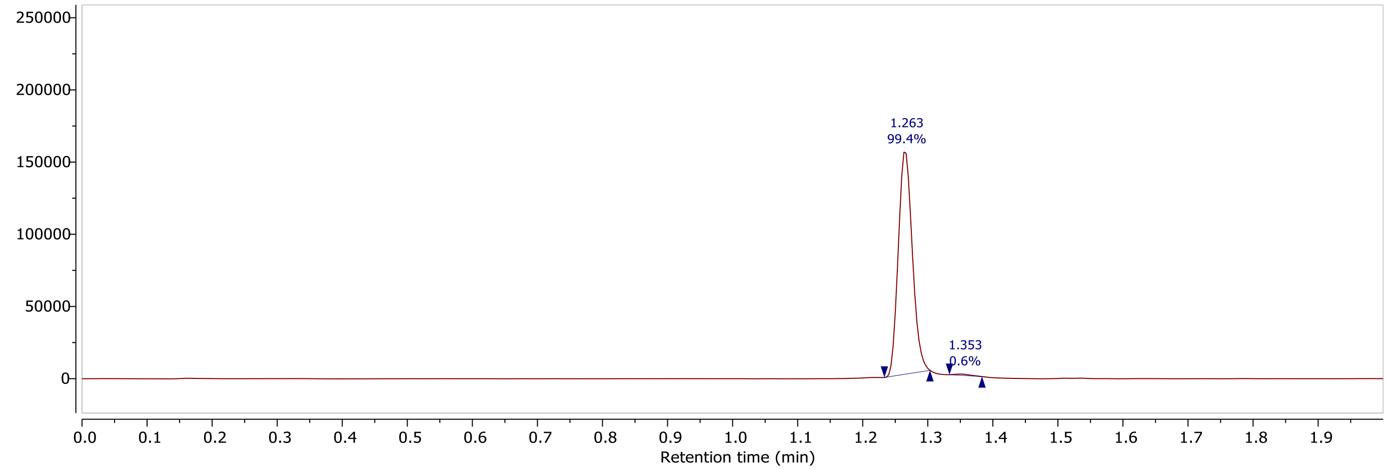

Compound 29

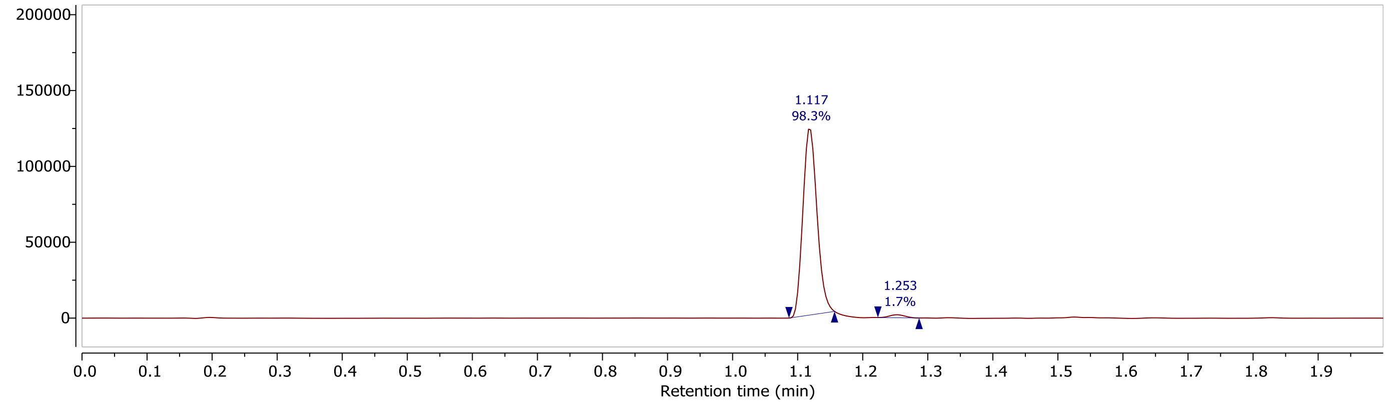

Compound 30

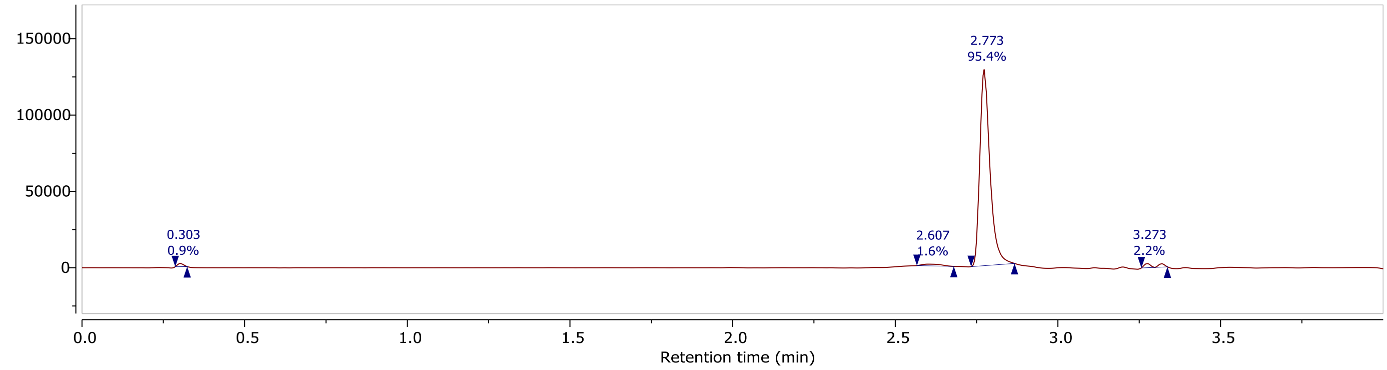

Compound 31

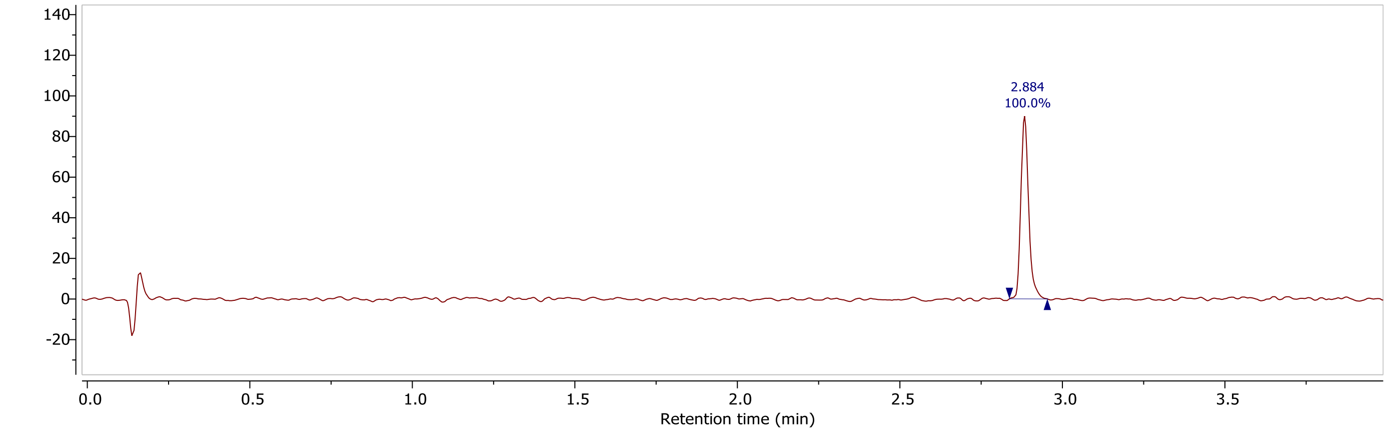

Compound 32

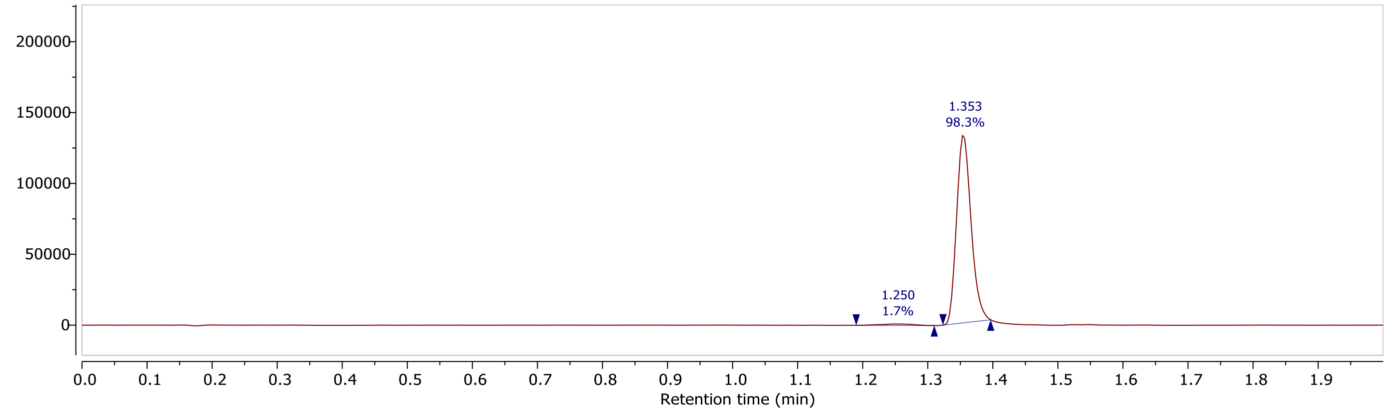

Compound 33

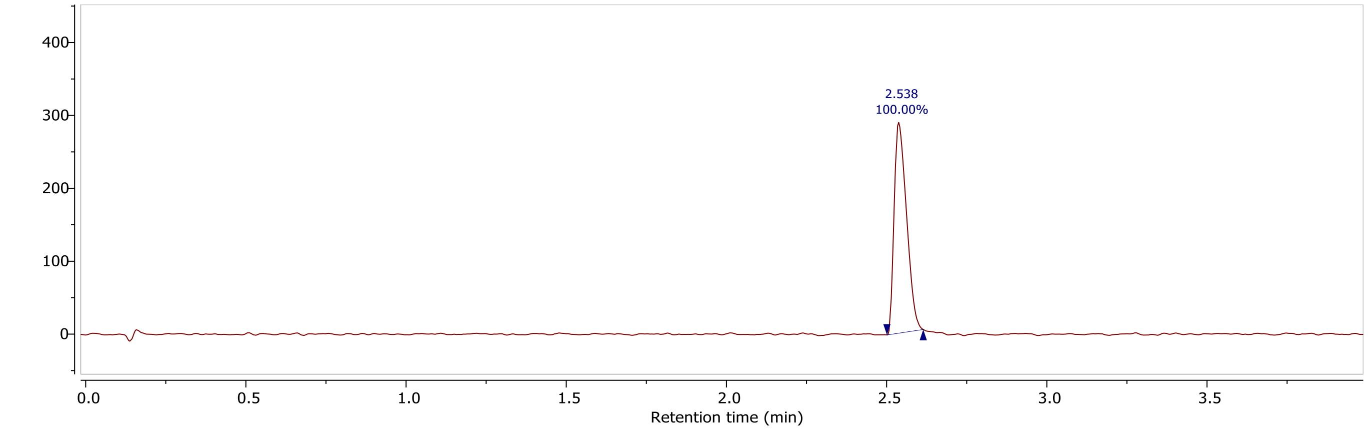

Compound 34

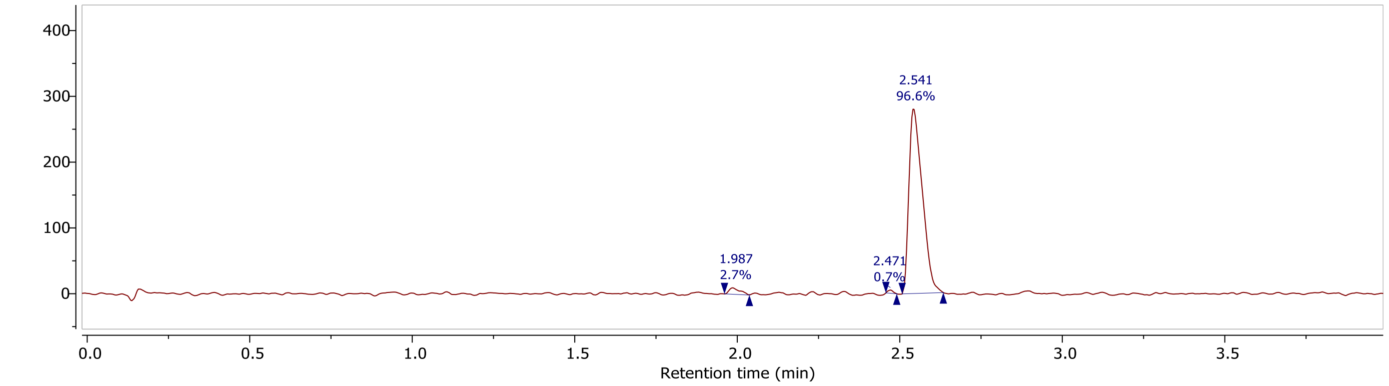

Compound 35

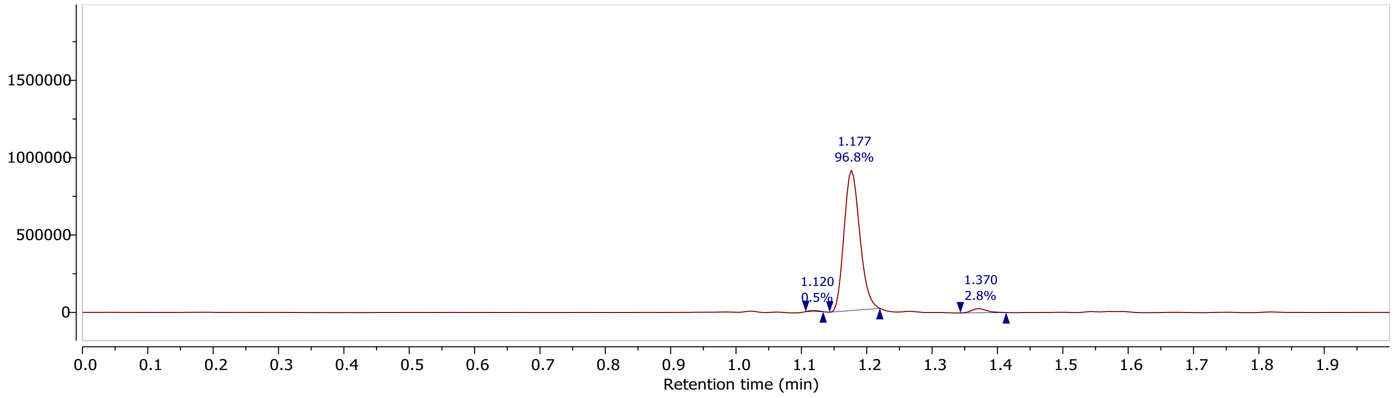

Compound 36

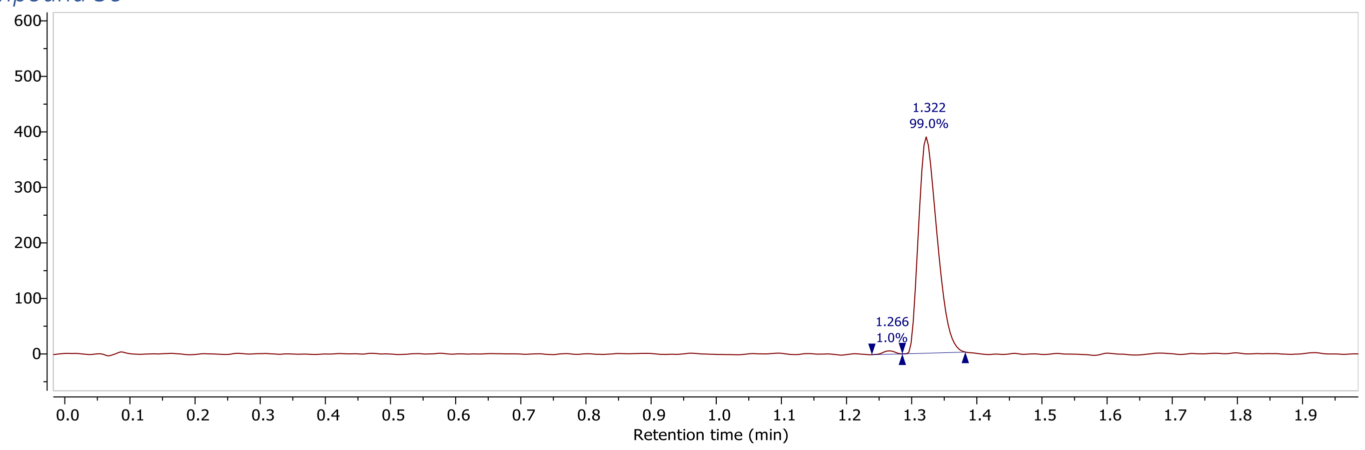

Compound 37

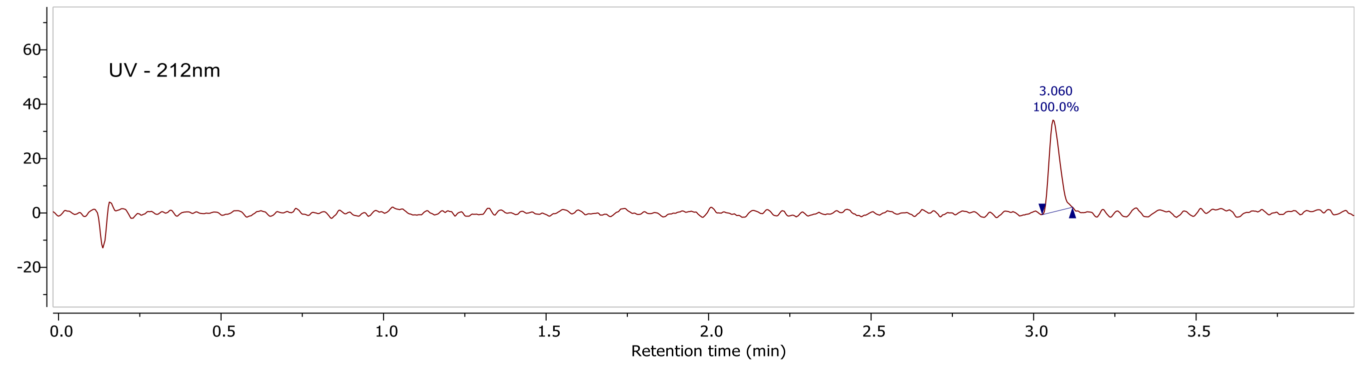

Compound 38

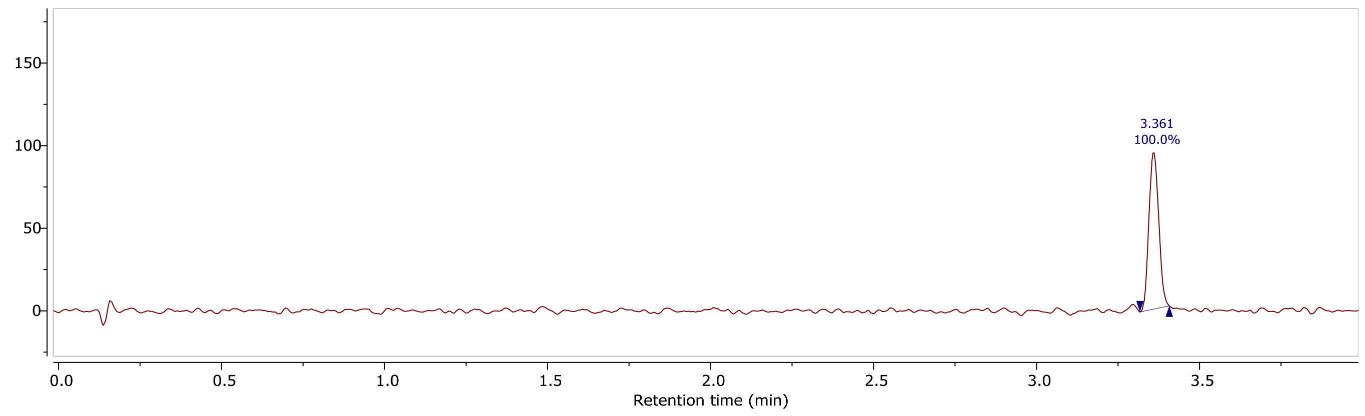

Compound 39

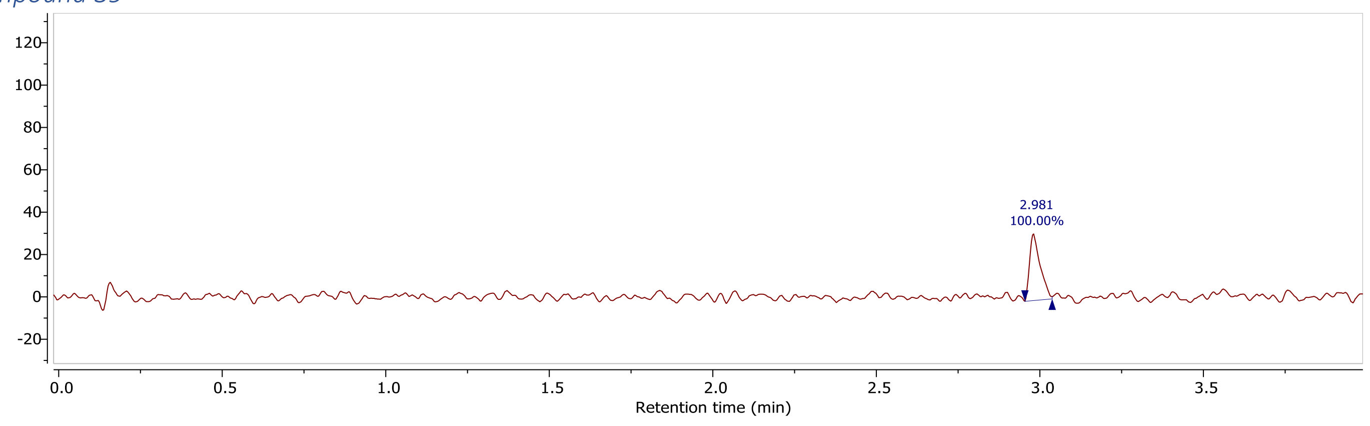

Compound 40

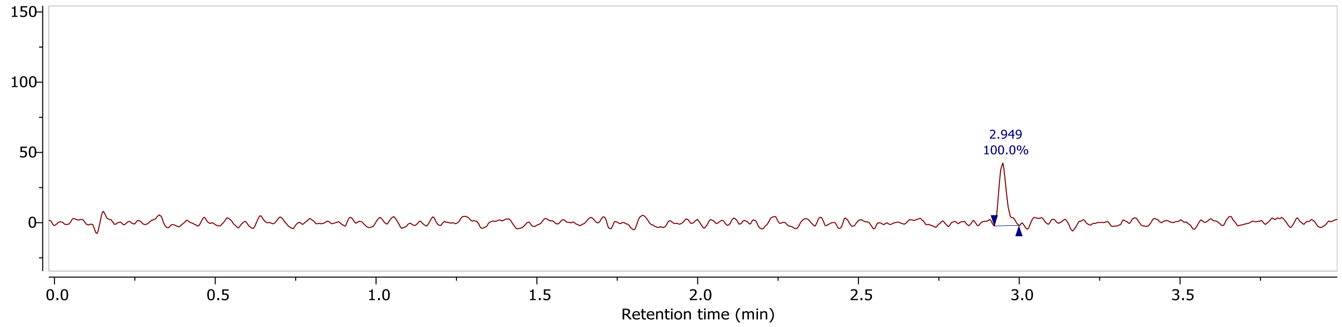

Supplement: Supplementary file 2 — jm2c01591_si_002.pdf [file jm2c01591_si_002.pdf]
